# Construction of a High-Density Genetic Map and Identification of Quantitative Trait Loci Linked to Fruit Quality Traits in Apricots Using Specific-Locus Amplified Fragment Sequencing

**DOI:** 10.3389/fpls.2022.798700

**Published:** 2022-02-14

**Authors:** Qiuping Zhang, Jiacheng Liu, Weisheng Liu, Ning Liu, Yuping Zhang, Ming Xu, Shuo Liu, Xiaoxue Ma, Yujun Zhang

**Affiliations:** Liaoning Institute of Pomology, Yingkou, China

**Keywords:** apricot, high-density linkage map, single nucleotide polymorphisms, quantitative trait locus, fruit quality traits

## Abstract

Improving fruit quality is one of the main tasks in modern commercial apricot breeding. Because of the lack of high-density linkage maps and fine mapping, it is difficult to obtain molecular markers that can assist in breeding for quantitative inheritance of fruit quality traits. In this study, specific-locus amplified fragment sequencing was used to genotype 169 seedlings of F1 apricot (*Prunus armeniaca* L.) progenies derived from crossing “Chuanzhihong” (H) with “Saimaiti” (S). After aligning to the *Prunus armeniaca* reference genome and filtering out low-quality variants, 6,012 high-quality single nucleotide polymorphisms were obtained and employed to construct a genetic map for each parent. The genetic linkage maps showed eight linkage groups of apricot, covering a distance of 809.6 cM in “H” and 1076.4 cM in “S”. The average distance between markers in “H” and “S” was 0.62 and 0.95 cM, respectively. To map quantitative trait loci (QTLs) for fruit quality, we investigated fruit quality traits, including fruit weight (FW), fruit height (FH), fruit lateral width (FL), fruit ventral width (FV), soluble solids content (SSC), and fruit firmness (FF) for all seedlings genotyped in 2018 and 2019. Eleven and nine QTLs linked to fruit quality traits were anchored on the “H” and “S” maps, respectively, and 1,138 putative candidate genes for 16 most significant regions on the corresponding chromosome were identified based on gene annotation. Among them, fruit size contained 648 genes in 11 intervals on the reference genome, SSC contained 372 genes in 3 intervals, and FF contained 117 genes in 2 intervals. Our findings uncovered the genetic basis of apricot fruit quality, and provided candidate genes for further molecular genetic studies on fruit quality and QTL targets for future marker-assisted selection of apricot quality improvement breeding.

## Introduction

Apricots (*Prunus armeniaca*) are among the most favored fruits, given their attractive golden yellow skin and juicy and aromatic fruit flesh (Mehlenbacher et al., 1990). According to the Food and Agriculture Organization of the United Nations (FAO), apricots are commercially grown in semi-arid regions of North China, Central and Near-Eastern Asia, Europe, and America with 4.26 million tons produced worldwide (FAOSTAT, 2019).

Quality traits, such as fruit size, fruit hardness, and solid content, are crucial to apricot consumers and are therefore a priority breeding objective (Liu et al., 2012). Previous studies have considered these traits as quantitative traits. Couranjou (1995) studied 15 F1 progenies issued from six parents over 3 years, and the results showed heritability (*h*^2^) between 0.6 and 0.9 for fruit size and flavor. For firmness, *h*^2^ was slightly lower than 0.6. More recently, *h*^2^ of some fruit traits was estimated for the Minaret × Betinka apricot population (Krska et al., 2009), and the estimated *h*^2^ of fruit weight (FW) was approximately 0.9, whereas *h*^2^ of fruit flavor ranged from 0.6 to 0.9. However, calculated from progeny means, *h*^2^ has often been overestimated and varies widely among different populations. Therefore, the development of marker-assisted selection (MAS) tools with high accuracy and efficiency is necessary for apricot breeding.

Several qualitative traits have been mapped on the genetic map, and their markers can be used in apricot breeding, such as resistance to *plum pox virus* (Lalli et al., 2008) and self-incompatibility (Soriano et al., 2008). Ruiz et al. (2010) performed the first study to identify several quantitative trait loci (QTLs) linked to ripening date, FW, sugars, acidity and firmness (FF) in apricots. More recently, few QTL studies have been reported, such as blooming date (Campoy et al., 2011), soluble solids content (SSC) (Socquest-Juglard et al., 2012), and fruit development period (Salazar et al., 2016). Although QTL analysis has been a hotspot in apricot quality research to date, the QTLs detected previously were always within long genomic regions because of the large gap among the interval loci.

Generally, fine QTLs are dependent on high-density genetic linkage maps. Molecular markers are employed for the construction of molecular genetic maps, and for the development of markers for parental analysis. Before 2016, several genetic maps were constructed using different types of markers, such as amplified fragment length polymorphisms (AFLPs) and simple sequence repeats (SSRs). The first genetic map of apricot was constructed for the F1 population derived from Goldrich × Currot using AFLP, RAPD, RFLP, and SSR markers (Hurtado et al., 2002), and then many maps were constructed with multi-marker (Olukolu et al., 2009; Socquest-Juglard et al., 2012) or a lot of co-markers (Campoy et al., 2011) to increase the marker density. Based on single nucleotide polymorphisms (SNPs) from transcriptome data and SSR marker genotyping, the average distance obtained between markers was 7.59 cM in Bergeron, 7.53 cM in Currot, and 5.6 cM in Goldrich (Salazar et al., 2016). The previously reported total lengths of approximately 500–600 cM were similar to those on the *Prunus* map. The mean densities of markers were approximately 2–4 cM. The highest marker density of 0.92 cM was obtained in G1 for LE3246 × Vestar (Lalli et al., 2008). However, these maps had a low density because of the limited markers and had a large gap between the markers. Because of their high abundance, SNP markers allow us to cover a large extension of the genome and are ideal for genetic mapping (Ball et al., 2010). Specific-locus amplified fragment sequencing (SLAF-seq) is a high-throughput technique widely adopted for the development of SNP markers in different species, such as the genetic map of peaches spanning 1098.79 cM with an average distance of 0.17 cM between adjacent markers (Shi et al., 2020) and the map of tea covering a distance of 3,965 cM with an average inter-locus distance of 1.0 cM (Ma et al., 2015). Zhang et al. (2019) constructed a high-density linkage map using the SLAF markers, and detected several QTLs for pistil abortion.

Several QTLs and candidate genes linked to fruit quality traits in *Prunus* have been described in other species (Aranzana et al., 2019), such as peaches (Dirlewanger et al., 2004; Quilot et al., 2005) and cherries (Wang et al., 2000). Phenotypic data for two consecutive years confirmed a high heritability of fruit size, FF, and FW QTLs were located on a narrow region of LG1 of Ambrunés cherry (Calle et al., 2020). A major QTL for FF, named *qP-FF4.1*, was identified in three sweet cherry populations (Cai et al., 2019), and candidate genes related to plant cell wall modification and various plant hormone signaling pathways were identified, with an expansin gene being the most promising candidate. Eighteen significant QTLs were identified for fruit development time, titratable acidity, and SSC traits on the same narrow region of linkage group 4 in cherry (Calle and Wünsch, 2020). In peaches, QTLs for SSC are dispersed on different linkage groups in different populations. For example, QTLs associated with SSC are located in LG2, LG4, and LG5 (Zeballos et al., 2016) in Venus × Big Top; however, QTLs linked to SSC have been mapped on LG5 in P1908 × Summergrand (Quilot et al., 2005). Only a small number of markers are common to different hybrid populations in peaches (Dirlewanger et al., 2004). Etienne et al. (2002) described sucrose synthase and invertase, which are associated with sugar accumulation. In another population, Nuñez-Lillo et al. (2019) identified four transcription factors, of which one is directly involved in the sugar accumulation process and one is a cell wall remodeling-related gene in the QTL of SSC. In apricots, candidate genes of SSC are involved in D-glucose and D-mannose binding (García-Gómez et al., 2019). Despite the high synteny within the *Prunus* genus, the markers and candidate genes of these quantitative traits are difficult to transfer to other species, such as apricots.

In this study, we constructed a high-density genetic map by SNP markers developed by the SLAF strategy, located several QTL regions associated with fruit quality traits for 2 years in an F1 population, and subsequently identified candidate genes, which could provide new insights into the regulation of fruit quality. Our results will help understand the genetic control of fruit quality traits in apricots, thereby providing a theoretical basis for improving fruit quality via MAS or genetic manipulation.

## Materials and Methods

### Plant Materials

A diploid hybrid population, including 169 seedlings used in this experiment, was generated from a cross between “Chuanzhihong” (H) as the maternal parent and “Saimaiti” (S) as the pollen donor in 2006. We selected two elite cultivars which have different geographical origin and distinguishable quality traits to generate the mapping population. “H,” a traditional native cultivar of Hebei province in China, belongs to the North China geographical group and is the most widely cultivated variety in that area. It shows excellent organoleptic quality: bright appearance, large fruit, high productive and late ripening. While “S” is a dominant cultivar of Xinjiang province in China which belongs to the central Asia geographical group. It was characterized as white and colorless, has no skin pubescence, and its fruit has high SSC, low acidity, small size, and soft flesh. The seedlings and the parental cultivars were planted at a density of 0.5 m × 2 m in the Chinese National Germplasm Repository for Plums and Apricots at the Liaoning Institute of Pomology (Xiongyue, China). The orchard was subjected to conventional field management and pest control methods. At the first fruit harvest in 2011, the plants were 6 years old.

### Fruit Quality Evaluation

The fruits of the seedlings were evaluated in 2018 and 2019. Ten ripening fruits from each genotype in this study were randomly harvested at the optimal ripening stage to evaluate FW, fruit height (FH), fruit lateral width (FL), fruit ventral width (FV), SSC, and FF. In order to reduce the environmental error (such as light), fruits outside the tree crown with sufficient sunlight were only harvested at the optimal timing of maturation based on skin ground color and firmness (Socquest-Juglard et al., 2012). The mean FW was measured using an electronic balance and was recorded in grams. FH, FL, and FV data were obtained using a Vernier caliper. The value of FF was determined in two directions for each fruit using a GY-3 penetrometer. The SSC was measured in°Brix using a digital handheld refractometer.

### Development of Single Nucleotide Polymorphism Markers Using Specific-Locus Amplified Fragment Sequencing

A total of 169 individuals were selected to construct a genetic map. Genomic DNA of the 169 F1 individuals and two parents, “H” and “S,” was extracted from young leaves using a genomic DNA isolation kit (Tiangen, Beijing, China), and processed into SLAF-seq libraries, following a previously described protocol (Sun et al., 2013). Briefly, according to the genome of *Prunus armeniaca*^1^ (Jiang et al., 2019) and the predesigned result of SLAF library, the genomic DNA was digested with two restriction endonucleases, *Hae*III and Hpy166II (New England Biolabs, NEB, United States). Rice (*Oryza sativa* L. *japonica*) was used as control to determine the effectiveness of the restriction digestion scheme. Comparing the 0.24M reads of the control with rice genome, the efficiency of double-end comparison was 88.44%, and only 92.57% of the residual restriction sites were inserted into the sequence reads, and which indicated that the digestion efficiency using these restriction enzyme were normal. The SLAF library was constructed using the SLAF-seq strategy with some modifications, and DNA fragments of the desired length were approximately 264–364 bp. The prepared SLAF libraries were sequenced on an Illumina HiSeq X Ten platform at BMK (Beijing, China) using the PE2500 (paired-end, 125 bp) strategy according to the manufacturer’s recommendations. SLAF labels were developed using clean data after removing the adapters, index sequences, and low-quality reads (quality score < 20e). SOAP software was used to map the clean reads with the terminal 5 bp trimmed onto the *Prunus armeniaca* reference genome. Sequences mapping to the same position were defined as one SLAF locus. The threshold for the definition of the SLAF locus was > 90% sequence identity (Sun et al., 2013).

Single nucleotide polymorphism loci of each SLAF locus were detected between the parents using GATK and samtools. The SLAF loci were filtered using the following standard: (1) excluded the SNP loci had three or more alleles, (2) excluded the lack of SNP genotypes in either parent, (3) the SNPs were supported by 4 or more reads, and (4) excluded the genotypes of the parents were monomorphic.

### Map Construction

Markers used for map construction were removed using the following criteria: (a) the average sequence depths should ≥ 14 in the parents; (b) for each marker, individuals that lacked genotyping data were less than 16 (<10% of the total); (c) the marker with the highest depth in the parent was retained for every 10 kb sequence; (d) all markers were tested using the Chi-square test (*p* < 0.01); and (e) if the genotypes of seedlings among segregation markers were identical, only one marker was retained. All polymorphic SNP loci were genotyped according to parental and offspring SNP alleles. A cross-pollinator population type, a cross between two heterozygous diploid parents, was composed of three segregation types (hk × hk, lm × ll, and nn × np). The segregation ratios of lm × ll and nn × np markers were expected to be 1:1, whereas those of the hk × hk markers were 1:2:1. Two parental genetic linkage maps were generated following a two-way pseudo-testcross strategy for outcrossing species (the cross-pollination population type) using JionMap 4.1 software (Van Ooijen, 2011). A logarithm of the odds (LOD) score of 7.0 was set to distinguish linkage groups. Regression mapping was used as the mapping algorithm, and the genetic distances in centimorgans (cM) were calculated based on Kosambi’s mapping function. MapChart 2.3 was used to draw linkage group figures. The linkage groups were named in accordance with the common chromosome order of the *Prunus* Genus (Dirlewanger et al., 2004).

### Quantitative Trait Loci Analysis

Quantitative trait loci analysis was performed using MapQTL6.01 software (Van Ooijen, 2006) based on the parental maps and phenotype data from 169 seedlings in 2018 and 2019. QTLs were initially detected using multiple-QTL-mapping (MQM), and the phenotypic and genotypic data were analyzed together by first performing a test of 1,000 permutations. The LOD score ≥ 3.0 and phenotypic variance explained (PVE) threshold ≥ 10% were considered as significant QTL interval, but only the LOD threshold of 3.0 was identified as QTL interval of SSC.

### Prediction of Candidate Genes

The physical locations of QTLs on the reference genome were identified by mapping the 20-kb flanking sequences of relevant markers to the genome. Genes within the QTL regions, together with their functional annotation information (see text footnote 1), were employed to identify putative candidate genes for quality trait QTLs in the context of their biological functional relevance to fruit quality.

## Results

### Distribution of Fruit Quality Traits

**FIGURE 1:**
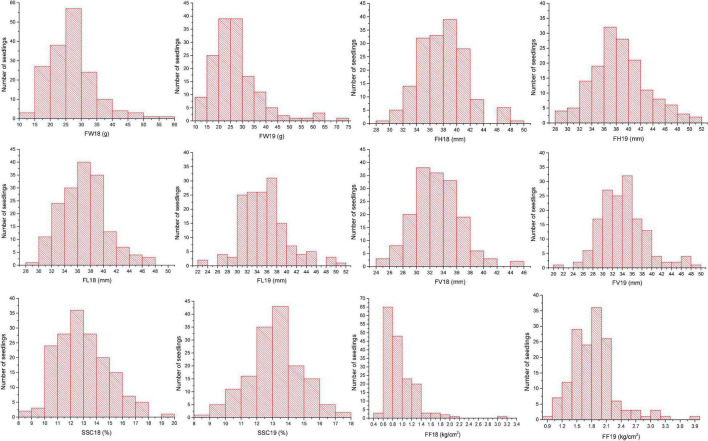
Frequency distribution of phenotyping data for each trait in 2018 and 2019.

Fruit weight is a typical quantitative trait that is controlled by multiple genes. Data on FW were collected in 2018 and 2019. According to the Shapiro-Wilk test, the values of FW varied continuously and belonged a normal distribution (Figure 1). The population means values of FW were 26.53 ± 7.34 g (2018) and 26.98 ± 10.13g (2019), which approximately equaled the value in 2 years (Supplementary Table 1). The population mean values varied with year, indicating the environmental factors had no significant impact. Fruit size traits, FH, FL, and FV, showed transgressive segregation patterns of distribution (Figure 1). From the mean value and variation, these traits were sensitive to environmental conditions.

Soluble solids content was measured in 2018 and 2019. The mean value was 12.92%, whereas the minimum was 8.24%, and the maximum was 19.58% in 2018. The average SSC in 2019 was 13.16%, and the data ranged from 8.46 to 17.66% (Supplementary Table 1). FF showed a typical normal distribution in both 2018 and 2019 (Figure 1); however, the FF in 2019 (1.83 ± 0.44) was higher than that in 2018 (0.94 ± 0.32) (Supplementary Table 1). Therefore, FF could be treated as a quantitative trait in subsequent analyses.

To estimate the correlation in fruit traits, correlation coefficients were calculated using 2 years of sampling data from the seedlings (Table 1). A significant correlation between FW and fruit size traits (FH, FL, and FV) were found in 2018 and 2019, while the correlations between fruit size traits were extremely significant in 2019. The Pearson correlation coefficients of FF were also significantly related to the other measured traits. No significant correlation existed between SSC and other traits, except for FF, in 2019. However, the Pearson correlation for each trait between the 2 years was significant.

**TABLE 1:** The pearson correlation coefficient for different fruit quality traits in 2018 and 2019.

	FW	FH	FL	FV	SSC	FF
Fruit weight (FW)	0.244**	0.879**	0.910**	0.933**	0.094	0.179*
Fruit height (FH)	0.157*	0.330**	0.853**	0.837**	0.007	0.185*
Fruit lateral width (FL)	0.868**	0.815*	0.265**	0.921**	–0.006	0.173*
Fruit ventral width (FV)	0.895*	0.665*	0.774**	0.280**	0.041	0.211**
Soluble solids (SSC)	–0.065	–0.017	–0.023	–0.111	0.274**	0.182*
Firmness (FF)	−0.238**	−0.182*	−0.333**	−0.254**	–0.060	0.200*

The correlation is significant at the 0.05 (*) and 0.01 levels (**). The diagonal italicized line shows the correlation between years. Below the diagonal line, the results correspond to the 2018 correlation while those above the line show the correlation for 2019.

### Marker Development

The raw data of each sample was generated using the SLAF strategy and the Illumina HiSeq 2500 system. The following two steps modified the raw data: first, the index sequences and low-quality terminal sequences were deleted in reads, and then, the low-quality reads, with the length of N bases being more than 10% of the reads, were removed. After filtering, the average Q30s of the samples was approximately 93.22% (minimum 91.61%), indicating the high quality of the data. Finally, 105.11 Gb of sequencing data were generated using the SLAF strategy, including 2.16 Gb of “H,” 2.29 Gb of “S,” and 99.99 Gb of the 169 F1 seedlings. The GC content of the parents was 39.84%, whereas that of the hybrid population was 40.34% (Supplementary Table 2).

Based on the SLAF tags, a high-fidelity SNP dataset was generated according to the *Prunus armerniaca* genome “Chuanzhihong.” Among the 360,704 SLAFs defined, a total of 1,388,415 polymorphic SNPs were obtained in all samples. Among these SNPs, there were 597,089 loci in the “H” parent, 13.37% of which were heterozygous, while there were 592,624 loci in the “S” parent, 14.86% of which were heterozygous (Table 2).

**TABLE 2:** Number of specific-locus amplified fragment (SLAF) tags developed and SNPs statistics.

Sample	SLAF number	Average depth	SNP number	Heterozygous ratio
Chuanzhihong	157,137	45.91	597,089	13.37
Saimaiti	153,421	41.74	592,624	14.86
Offspring	113,154	16.62	41,900	14.00

### Genotyping and Linkage Map Construction

According to the defined filtering criteria, polymorphic SNP tags with low sequence depths, excessive missing data, or Mendelian errors were removed. The 6,012 polymorphic SNP markers were genotyped and successfully classified into four segregation patterns: ef × eg, hk × hk, lm × ll, and nn × nap. The marker codes lm × ll (52.86%) and nn × nap (38.66%) represented markers with one heterozygous parent (providing 1:1 segregation ratios), whereas hk × hk (8.38%) and ef × eg (0.10%) represented markers where both parents were heterozygous (providing 1:2:1 or 1:1:1:1 segregation ratios) (Supplementary Table 3).

**FIGURE 2:**
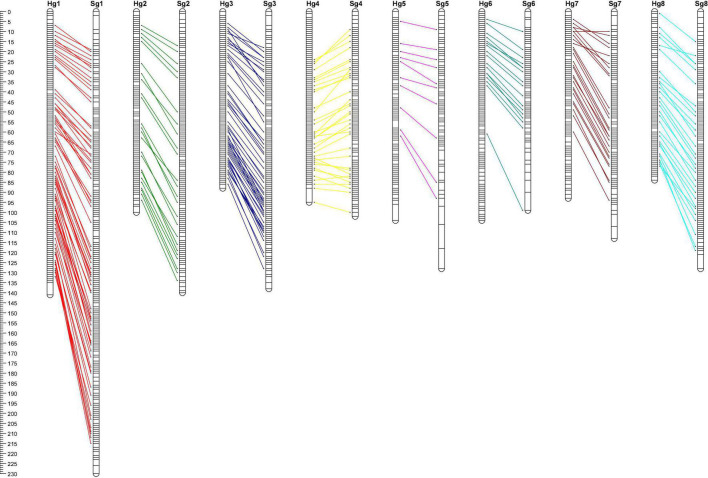
Comparison of genetic map between “Chuanzhihong” (H) and “Saimaiti” (S).

A total of 6,006 SNP markers, excluding the ef × eg pattern, were used to construct the female (“H” parent) and male parental maps (“S” parent) using JionMap4.1 software, respectively. The genetic linkage maps in both parents showed the eight linkage groups of apricot, covering a distance of 809.6 cM in “H” and 1022.7 cM in “S” based on the progeny with a grouping LOD value of 7 (Figure 2). In the “H” map, 1,307 SNP markers were assigned to eight linkage groups (Hg1–Hg8) (Table 3 and Supplementary Table 4), and markers mapped on each linkage group ranged from 99 (Hg5) to 287 (Hg1). The longest chromosome was Hg1 (141.1 cM), whereas the shortest chromosome was Hg8 (84.3 cM). In the “S” map, 1,145 SNP markers were mapped to eight linkage groups (Sg1–Sg8) (Table 3 and Supplementary Table 4), and markers mapped on each linkage group ranged from 79 (Sg5 and Sg6) to 269 (Sg1). The longest and shortest chromosomes were Sg1 (229.7 cM) and Sg6 (98.5 cM), respectively. The average distance obtained between markers was 0.62 cM in “H” and 0.96 cM in “S.” The largest gap in the no marker coverage on this map was located in Hg5 (8.0 cM) and Sg5 (12.7 cM), and only a few intervals were larger than 5 cM. These results indicated that these genetic maps were of high quality with high resolution.

**TABLE 3:** Feature of each linkage group of parents in this study.

Chuanzhihong linkage map						Saimaiti linkage map					
Group	Marker No.	Total distance (cM)	Average distance (cM)	Max. Gap	Gaps < 5 cM%	Group	Marker No.	Total distance (cM)	Average distance (cM)	Max. Gap	Gaps < 5 cM%
Hg1	287	141.1	0.49	5.7	99.65	Sg1	269	229.7	0.86	7.1	99.63
Hg2	132	100.4	0.77	3.8	100.00	Sg2	165	140	0.85	3.1	100.00
Hg3	211	87.7	0.42	2.2	100.00	Sg3	175	138.1	0.79	4.3	100.00
Hg4	182	95.2	0.53	6.9	99.45	Sg4	113	101.5	0.90	2.2	100.00
Hg5	99	104.1	1.06	8.0	98.99	Sg5	79	128	1.64	12.7	96.20
Hg6	145	103.6	0.72	2.2	100.00	Sg6	79	98.5	1.26	8.6	97.47
Hg7	117	93.2	0.80	3.3	100.00	Sg7	115	113	0.99	6.1	98.26
Hg8	134	84.3	0.63	2.6	100.00	Sg8	150	127.6	0.86	6.8	99.33
Total	1,307	809.6	0.62	–	99.76	Total	1,145	1076.4	0.95	–	98.86

We developed two genetic maps using 2,183 SNP markers, which showed a high depth of coverage for the parents (mean of ∼100-fold) and the F1 progeny (mean of ∼27-fold). All the markers on the different linkage groups were aligned to the reference genome sequence (Supplementary Figure 1 and Supplementary Table 5), which indicating that all linkage groups had good linear agreement between the physical chromosomes and genetic maps.

### Identifying Quantitative Trait Loci for Fruit Quality Traits

**FIGURE 3:**
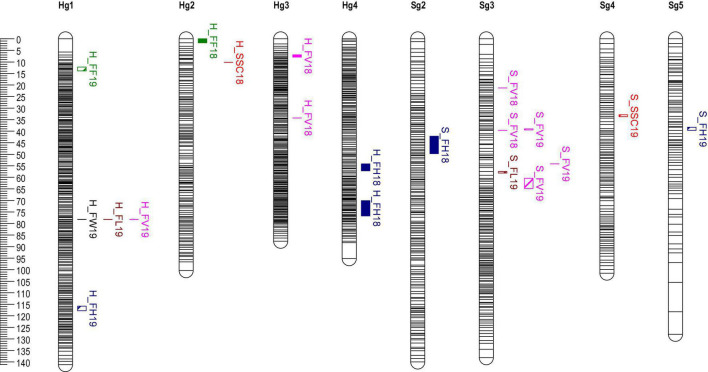
QTLs of fruit quality traits located on “H” and “S” by multiple-QTL-mapping (MQM). QTLs are represented by black bars positioned at the right of each linkage group. QTLs of different trait are drawn by Mapchart software with different RGB colors.

Quantitative trait loci analysis of fruit size, FF, and SSC was performed using MapQTL6.01 with the above SNPs high-density linkage map. A total of 11 significant QTLs were detected using the MQM method in the “H” map. Of the 8 QTLs of fruit size, 1 contributed to FW, 3 to FH, 3 to FV, and 1 to FL (Figure 3 and Table 4). In Hg1, FW19, FL19, and FV19 were clustered in the same interval (78.3 cM). The QTLs related to fruit size were distributed in six intervals of the linkage groups Hg1, 3, and 4. In 2018, the one QTL of SSC was located on 10.2 cM of the Hg2 group, however, the reproducible QTL interval was no significant in 2019 (LOD = 2.55, PVE = 7.40) (Supplementary Table 6). The two QTLs of FF were located on Hg1 and Hg2, whereas they could not be detected repeatedly in different years.

**TABLE 4:** The significant QTLs and regions in reference genome.

Linkage groups	Position (cM)	QTL Loci	Markers	LOD	PVE%	Chr.	Position (Mb)
Hg1	78.3	H_FW19	Marker25196	3.89	11.10	LG2	21.35
Hg1	78.3	H_FL19	Marker25196	3.54	10.10	LG2	21.35
Hg1	78.3	H_FV19	Marker25196	3.81	10.80	LG2	21.35
Hg1	115.9–117.9	H_FH19	Marker33669	3.68	10.50	LG2	4.04
Hg1	115.9–117.9	H_FH19	Marker34090, Marker34145, Marker33411	3.68	10.50	LG2	5.22–5.97
Hg4	54.2–57.2	H_FH18	Marker40697	4.20	10.90	LG3	17.02–18.37
Hg4	70.1–76.7	H_FH18	Marker38162	5.36	13.70	LG3	22.25–22.82
Hg3	6.9–7.9	H_FV18	Marker63202	4.13	10.70	LG4	0.03–1.21
Hg3	6.9–7.9	H_FV18	Marker62528, Marker62787	4.13	10.70	LG4	2.63–3.41
Hg3	34.3	H_FV18	Marker58893	4.13	10.70	LG4	7.30–7.72
Hg2	10.2	H_SSC18	Marker121293	3.31	8.60	LG5	4.85–6.66
Hg2	10.2	H_SSC18	Marker64728	3.31	8.60	LG4	19.71–20.79
Hg1	13.1–14.0	H_FF19	Marker15306, Marker15130, Marker15102, Marker14962	4.13	11.70	LG2	41.54–42.68
Hg2	0–1.7	H_FF18	Marker64041, Marker8120	4.33	11.10	LG5	0.59–0.76
Sg2	42.2–49.7	S_FH18	Marker119270	4.30	11.10	LG3	17.02–18.37
Sg5	38.3–39.7	S_FH19	Marker92443	3.89	11.00	LG7	6.80–7.77
Sg5	38.3–39.7	S_FH19	Marker57966	3.89	11.00	LG7	6.80–7.77
Sg3	54.2	S_FV19	Marker57693	4.12	11.70	LG4	9.44
Sg3	21.3	S_FV18	Marker62942	3.88	10.10	LG4	0.03–1.21
Sg3	60.4–64.8	S_FV19	Marker57207	4.92	13.80	LG4	10.28–11.53
Sg3	39.7	S_FV18	Marker62366	4.17	10.80	LG4	2.63–3.41
Sg3	39.0–39.5	S_FV19	Marker62327, Marker62606	3.98	11.30	LG4	2.63–3.41
Sg3	57.5–58.1	S_FL19	Marker57739, Marker58388	3.73	10.60	LG4	8.92–9.21
Sg4	32.9–33.7	S_SSC19	Marker42337	3.35	9.60	LG3	12.61–14.22

A total of nine QTLs were identified at the genome-wide threshold in the “S” map (Figure 3 and Table 4), eight were identified for fruit size, one for SSC, and none for FF. Most of the QTLs related to fruit size were distributed in six intervals of the linkage groups Sg3. Only one significant QTL interval for SSC was detected on Sg4, however, there was an insignificant QTL locus (Marker65691) on Sg2, which was in the same position as the Hg2 loci.

### Candidate Genes for the Quantitative Trait Loci of Fruit Quality Traits

Seventeen candidate regions related to fruit quality were identified based on the phenotypic QTLs and the physical positions on the *Prunus armeniaca* genome pseudo-chromosomes, and suitable candidate genes were directly searched for those regions.

A significant region related to FW was identified on ∼21.1 Mb in LG3 (Table 4), and 12 candidate genes among the genomic regions were obtained from the reference genome database (Supplementary Table 7). Five regions for FH detected 330 genes, while four regions for FV identified 272 candidate genes. A lot of 34 candidate genes were annotated in one region for FL in LG4 (Supplementary Table 7). These candidate genes related to fruit size and fruit shape were associated with cell cycle control, carbohydrate transport and metabolism, signal transduction, and transcription factors, such as cyclin-dependent kinase gene (PARG13153 and PARG13199), parafibromin gene (PARG10966), Cytochrome P450 gene (PARG12851 and PARG13939), and extracellular region activity (PARG13223). The PARG12738 gene regulates auxin polar transport, and the gene products of PARG11414, PARG12146, PARG12187, and PARG14147 respond to auxin.

For SSC, the three most important intervals were 4.85–6.66 Mb of the LG5, 12.61–14.22 Mb of the LG5, and 19.71–20.79 Mb of the LG4, respectively (Table 4). A total of 438 candidate genes were annotated in these regions, including electron transport and sugar synthesis in the photosynthetic system, intracellular sugar transport, regulatory genes, transcription factors, plant hormones, and so on (Supplementary Table 7). These regions were identified as a series of genes, including the PARG17513, which encodes malate dehydrogenase (NADP+), and PARG15534, which encodes a protein of the subunit of the photosystem I reaction center, and the PARG17684 and PARG17899 genes regulate transmembrane transporter activity.

A total of 117 candidate genes for FF were identified in two intervals. These candidate genes were involved in processes, such as cell metabolism, cell wall composition or degradation, vesicular transport, transcription factors, and signal transduction (Supplementary Table 7). For example, polygalacturonase (PG) gene (PARG16950), beta-glucosidase gene (PARG09228 and PARG09230), and aminocyclopropane carboxylate oxidase gene (PARG12550), which may be related to FF. In addition, the 3-ketoacyl-CoA synthase gene (PARG09079 and PARG09080) contributed to cuticle wax and suborn biosynthesis.

## Discussion

### Single Nucleotide Polymorphism Markers by Specific-Locus Amplified Fragment Sequencing Are an Effective Marker for the Construction of a High-Density Linkage Map in Apricots

Molecular markers, such as AFLPs, RAPDs, RFLPs, and SSRs, have been used for genetic map construction (Hurtado et al., 2002), and then many maps were constructed with multi-marker (Olukolu et al., 2009; Socquest-Juglard et al., 2012) to increase the marker density. Because of the lower availability of AFLP, RAPD, RFLP, and SSR markers, the map density was not high enough and the adjacent marker gaps were larger in previous apricot linkage maps (Lalli et al., 2008). SNPs are the most common genetic variation in the whole genome, and they are very important genetic markers for constructing a high-density genetic map, resulting in a significant decrease in the time and cost of genotyping (Zeballos et al., 2016; Zhang et al., 2020). Recently, some SNP markers have been used to construct genetic maps in apricots to screen for QTLs related to fruit development period-related traits and graft compatibility (Salazar et al., 2016; Pina et al., 2021). However, SNP markers from RNA-seq libraries have substantial limitations in detecting polymorphisms in parents other than the sequencing materials (Salazar et al., 2016). QTL loci for some interesting traits could not be accurately mapped to a smaller region because of limitation markers or loss of genetic information in a large region. Because of large-scale SNP discovery and genotyping, SLAF sequencing technologies have been effectively applied for genetic map construction and QTL detection in apricot (Zhang et al., 2019) and many other plant species (Ma et al., 2015; Shi et al., 2020).

In this study, we constructed two high-density genetic linkage maps using 2,183 SNP markers in the apricot. The “H” and “S” maps spanned 809.6 cM and 1076.4 cM, respectively. The average distance between markers was 0.62 cM in “H” map and 0.95 cM in “S” map. Vilanova et al. (2003) established a map of Lito apricots covering 602 cM, with an average density between pairs of markers of 3.84 cM. Olukolu et al. (2009) published a map of Perfection including 621 AFLP and 34 SSR markers covering the apricot genome of 550.6 cM with an average marker spacing of 0.84 cM; however, the AFLP markers were not evenly spaced across the apricot genome with adjacent marker gaps of up to 5 cM on some linkage groups. The Currot apricot linkage map covered 414.3 cM of the genome with an average interval of 7.53 cM between markers (Salazar et al., 2016). The eight linkage groups of Monqui and Paviot maps were composed of 557 SNPs and 501 SNPs, with genetic distances of 780.2 and 690.4 cM, respectively (Pina et al., 2021). Compared with the published linkage maps, the genetic group constructed in our study covered a longer genetic distance and contained a higher marker density. The differences in the length and density of genetic maps may be related to the genetic distance between the parents, the number of markers, and the population size used in different studies (Zhang et al., 2020). For example, Shi et al. (2020) published a peach map including 7,998 SLAF markers covering the peach genome of 1098.79 cM based on an F1 peach population of 202 individuals. The increased marker number and density could enhance the mapped QTL number and precision. However, in spite of the marker density, were not found important QTLs in this study. Our experimental results showed that SLAF-based SNP markers were highly effective for constructing high-density genetic maps in apricots.

### Quantitative Trait Loci Identification and Candidate Gene Prediction

Apricot consumers care about fruit quality traits, including fruit size, sugar content, and firmness; however, the genetics of fruit quality are not highly developed, which is one of the main bottlenecks for apricot breeders. Most of the evaluated traits showed significant differences between years, suggesting that environmental orchard conditions affect phenology and fruit quality traits, as reported in peaches by Nuñez-Lillo et al. (2019). Other phenotypic studies in apricot showed exceptionally high inter-annual correlations for harvest date and FW (Ruiz et al., 2010; Salazar et al., 2013). Hence, the collection of reliable phenotypic data is a critical step in the identification of genomic markers. Genes and environments have the potential to influence phenotypic variations. Heritability estimates how much variation in a phenotypic trait in a population is caused by genetic variation among individuals in that population. Based on the F1 apricot progeny of “H” × “S,” the heritability coefficients of FW, FF, and SSC were 0.45, 0.65, and 0.93, respectively, in previous studies (Liu et al., 2020). This result showed that the SSC trait is controlled more by genetics, and the FW trait is lower, whereas the FF trait is in the middle for apricots. Using the same population in this study, correlation analysis among different years showed that there was also a significant correlation between the same traits in different years, indicating year-to-year stability of phenotypes.

Environmental factors have a significant influence on the efficiency of trait improvement in conventional breeding. Identifying of genomic regions involved in fruit quality traits offers the opportunity to optimize apricot breeding programs by introducing an early MAS and identifying candidate genes related to these traits (Ruiz et al., 2010).

Fruit size is an important factor in determining the appearance value of fruit in *Prunus*, and many QTL intervals have been mapped on genetic linkage maps. Wang et al. (2000) detected QTLs for fruit size in LG2 and 6 in cherries; recently, Salazar et al. (2020) identified a consistent QTL of FW in a *Prunus salicina* population in the same LG. In peaches, several QTLs for FW have been detected in LG1 and 4 in different years (Eduardo et al., 2010), and Salazar et al. (2013) also described different QTLs in the same linkage groups in apricots. However, the most relevant QTLs were identified in LGs 6 and 7 for two consecutive years in apricots (García-Gómez et al., 2019). Candidate gene searches based on QTL intervals are universal in the current genetic study. In our study, we mapped the QTL intervals of the fruit size traits (FW, FH, FL, and FV) on chr1 (LG2), 3 (LG4), and 4 (LG3) in the apricot, and identified 648 candidate genes for the 11 most significant regions. These genes are involved in cell division or enlargement regulation factors, and auxin response, which was similar to the results for apples (Sun et al., 2015). It is well known that fruit size is determined by the number of cell divisions and cell size. Mesocarp cell number or layers in *Prunus* are believed to be one of the most important factors resulting to the fruit size difference during the early development of fruit. A total of 23 genes of cell number regulator (CNR) subfamily involve in fruit size were identified on G2 and G6 chromosomes in sweet cherry (De Franceschi et al., 2013). We found that four cyclin-dependent kinase genes and a PARG12835 gene, which were reported encoding an E3 ubiquitin ligase that negatively regulates cell division by targeting its substrate(s) for degradation via the ubiquitin-proteasome pathway (Song et al., 2007). Cytochrome P450 (CYP) (PARG12851 and PARG13939) subfamily, termed CYP78A that has been shown to be involved in plant organ growth through increasing cell expansion in the integuments of developing seeds (Fang et al., 2012). The other one, cell volume or size is responsible for fruit enlargement during the late growth stage of fruit. In this study, we detected five genes (PARG12738, PARG11414, PARG12146, PARG12187, and PARG14147) encoding proteins that might regulate fruit elongation by modulating auxin. Previous reports demonstrated that CYP450 possess also played vital roles in the catalysis of early brassinosteroid intermediates to promote cell elongation in rice (Xu et al., 2015). Currently, the families of CNR and CYP genes have been reported to regulate the fruit size (Aranzana et al., 2019). In addition, the annotated parafibromin gene (PARG10966) in this study contributed to a decrease in fruit size by regulating protein-DNA interactions and inhibiting the cell proliferation.

Fruit flavor is dependent on the soluble sugars and acids. SSC is an important index of the internal quality of fresh fruits. Numerous QTLs have been identified on all eight chromosomes for traits and metabolites associated with SSC in *Prunus* (Aranzana et al., 2019); however, many authors have described QTLs for SSC distributed in LG3, 4, and 5 in apricots. The large variability of SSC observed in F1 progeny in apricots allowed the identification of a QTL on LG1 in 2011 and on LG4 in 2012, and the SSC trait was not correlated among years (Socquest-Juglard et al., 2012). Ruiz et al. (2010) placed different QTLs in LG3 and 4, in agreement with Salazar et al. (2013), who identified QTLs in LG3, 4, and 5. The QTL interval for SSC was located on LG4, and SSR4_13182815 was related to the SSC trait in apricots (García-Gómez et al., 2019). QTLs identified for the same traits as those previously described in apricots were often detected in regions comparable to those identified in peaches. This reflects the high homology between the peaches and apricots. In peaches, QTLs for SSC have previously been linked to LG1, 2, 4, 5, and 6 (Zeballos et al., 2016). In this study, a stable QTLs related to SSC in chr2 was detected on the “H” map, and were anchored in a 4.85–6.66 Mb segment of the LG5 in the apricot genome. Etienne et al. (2002) detected a candidate gene/QTL co-location for SSC, which involved a cDNA encoding a sucrose transporter protein. Moreover, they described two proteins associated with sugar accumulation, sucrose synthase and invertase, both of which are involved in sucrose metabolism. However, we identified 372 candidate genes for the SSC trait based on functional gene annotation in apricots, which are involved in electron transport and sugar synthesis in the photosynthetic system, intracellular sugar transport, regulatory genes, transcription factors, plant hormones, and so on.

Fruit firmness determines the long-distance transportation and commercial value of apricot fruits and is affected by multiple factors, such as genetic characteristics and fruit maturity. During fruit ripening, changes in ethylene and the cell wall structure affect FF; therefore, the genes involved in cell wall composition, modification, and ethylene metabolic or signaling pathways were considered to be potential candidate genes for FF. For example, PG and PME are two important cell wall hydrolases that have been widely studied during fruit softening in apricots. This trend has also been observed in other species. *PG1* and *ACO1* are tightly linked to QTLs mapped on LG10 for FF in apples (Chang et al., 2014). *PpYUC11* and Prupe.6G150900.1 were identified as candidate genes for controlling the stony hard phenotype in peaches (Pan et al., 2015). Although FF is also quantitatively inherited in apricots, Wei et al. (2018) identified PaPG as a candidate gene that could be used as a molecular marker for FF. Hou et al. (2019) suggested that PG and PME genes might play crucial roles in apricot softening. Near freezing temperature storage could inhibit the increase in cellulose and beta-galactosidase activity, and delay apricot fruit softening after simulated transport vibration (Cui et al., 2021). In this study, we obtained similar result in that 117 candidate genes were related to this trait, such as PG gene, beta-glucosidase gene and ethylene-responsive transcription factor. In addition, we found that 3-ketoacyl-CoA synthase gene (PARG09079 and PARG09080) might also contribute to FF by increasing the cuticle wax and suborn on the fruit surface.

In this study, we established two high-density genetic maps based on the SLAF-seq method. The female map (“H” map) covered 809.6 cM with an average density of 0.62 cM between pairs of markers, and the male map (“S” map) spanned 1022.7 cM with an average distance of 0.96 cM between adjacent loci. Sixty-two QTLs for fruit quality were mapped on the “H” map, whereas 45 QTLs were located on the “S” map. Thirty-four candidate regions and 1,138 genes controlling fruit quality traits were identified from the corresponding genomic regions. However, it is necessary to further confirm the precise candidate genes using quantitative PCR at critical periods to identify their control of FW, SSC, and FF traits in apricots. Briefly, we concluded that application of SLAF-seq for genetic map construction improved the precision of QTL detection and should be utilized in future molecular selection breeding processes in apricots.

## Data Availability Statement

The data sets presented in this study can be found in online repositories of NCBI. The names of the repository/repositories and accession number(s) can be found below: SRA, PRJNA707359.

## Author Contributions

QZ designed the research, conducted the data analyses, and wrote the manuscript. JL and WL collected the phenotyping data. NL, YPZ, MX, SL, XM, and YJZ participated in the picking and management of test materials. All authors discussed the results and commented on the final manuscript.

## Conflict of Interest

The authors declare that the research was conducted in the absence of any commercial or financial relationships that could be construed as a potential conflict of interest.

## Publisher’s Note

All claims expressed in this article are solely those of the authors and do not necessarily represent those of their affiliated organizations, or those of the publisher, the editors and the reviewers. Any product that may be evaluated in this article, or claim that may be made by its manufacturer, is not guaranteed or endorsed by the publisher.

## Footnotes

1
https://www.rosaceae.org/species/prunus_armeniaca/genome_v1.0
