# Supplementary material for: Construction of a High-Density Genetic Map and Identification of Quantitative Trait Loci Linked to Fruit Quality Traits in Apricots Using Specific-Locus Amplified Fragment Sequencing
Source: Front Plant Sci. 2022 Feb 14;13:798700. doi: 10.3389/fpls.2022.798700 (PMC8882730; doi:10.3389/fpls.2022.798700)
Supplement: Supplementary file 1 [file Table_1.DOCX]

**Supplementary Table 1. An overview of population features of phenotyping data for fruit quality traits**

|  | FW18 | FH18 | FL18 | FV18 | SSC18 | FF18 | FW19 | FH19 | FL19 | FV19 | SSC19 | FF19 |
| --- | --- | --- | --- | --- | --- | --- | --- | --- | --- | --- | --- | --- |
| N | 168 | 168 | 168 | 168 | 169 | 169 | 153 | 153 | 152 | 153 | 153 | 153 |
| Mean | 26.53 | 37.92 | 37 | 33.05 | 12.92 | 0.94 | 26.98 | 38.5 | 35.52 | 34.08 | 13.16 | 1.83 |
| SD | 7.34 | 3.5 | 3.49 | 3.53 | 2.01 | 0.32 | 10.13 | 4.45 | 4.69 | 4.65 | 1.68 | 0.44 |
| Min | 12.8 | 29.74 | 29.23 | 24.02 | 8.24 | 0.59 | 11 | 28.97 | 22.42 | 21.47 | 8.46 | 1 |
| Max | 59.8 | 48.09 | 47.09 | 45.18 | 19.58 | 3.13 | 70.8 | 50.34 | 50.31 | 49.54 | 17.66 | 3.84 |
| Skewness | 1.233 | 0.349 | 0.462 | 0.384 | 0.439 | 2.77 | 1.579 | 0.396 | 0.51 | 0.669 | -0.057 | 1.309 |
| Kurtosis | 3.012 | 0.342 | 0.202 | 0.694 | -0.027 | 12.849 | 3.648 | 0.107 | 1.074 | 1.111 | 0.148 | 3.399 |
| Sig (2-tailed) | 0.067 | 0.077 | 0.164 | 0.222 | 0.670 | 0.000 | 0.003 | 0.547 | 0.052 | 0.242 | 0.516 | 0.035 |
