# Supplementary material for: Construction of a High-Density Genetic Map and Identification of Quantitative Trait Loci Linked to Fruit Quality Traits in Apricots Using Specific-Locus Amplified Fragment Sequencing
Source: Front Plant Sci. 2022 Feb 14;13:798700. doi: 10.3389/fpls.2022.798700 (PMC8882730; doi:10.3389/fpls.2022.798700)
Supplement: Supplementary file 2 [file Table_2.DOCX]

**Supplementary Table 2. Details quality information of the reads for SLAFs in this study**

| Populations | Clean read | Clean base | GC(%) | Q30(%) |
| --- | --- | --- | --- | --- |
| Female Chuanzhihong H | 9,094,217 | 2,289,762,234 | 39.98 | 93.58 |
| Male Saimaiti S | 8,583,531 | 2,160,957,058 | 39.70 | 93.21 |
| CS003 | 2,911,437 | 732,933,164 | 39.30 | 93.26 |
| CS005 | 1,833,201 | 460,604,154 | 39.98 | 92.75 |
| CS006 | 2,620,158 | 659,121,782 | 40.18 | 92.64 |
| CS007 | 1,595,749 | 400,907,822 | 42.10 | 93.59 |
| CS013 | 1,599,662 | 401,445,670 | 41.99 | 93.64 |
| CS014 | 1,907,104 | 479,461,656 | 40.35 | 92.79 |
| CS016 | 1,260,556 | 316,578,506 | 41.40 | 93.34 |
| CS017 | 2,211,823 | 556,115,076 | 39.54 | 92.69 |
| CS019 | 2,374,900 | 597,342,830 | 40.60 | 93.52 |
| CS023 | 2,408,225 | 605,642,630 | 41.69 | 93.28 |
| CS027 | 1,467,979 | 368,534,364 | 41.53 | 93.67 |
| CS028 | 2,683,961 | 675,144,594 | 39.22 | 93.87 |
| CS029 | 2,304,911 | 578,978,872 | 40.06 | 93.62 |
| CS030 | 2,370,818 | 596,736,284 | 39.17 | 92.62 |
| CS032 | 1,803,461 | 453,184,658 | 41.91 | 93.51 |
| CS033 | 2,146,480 | 538,586,946 | 42.54 | 93.06 |
| CS035 | 2,563,305 | 644,888,452 | 39.49 | 93.26 |
| CS037 | 1,804,143 | 453,314,442 | 39.51 | 93.00 |
| CS039 | 2,830,462 | 712,428,750 | 39.53 | 93.13 |
| CS042 | 1,397,137 | 350,491,944 | 41.78 | 93.59 |
| CS043 | 2,059,086 | 518,129,114 | 38.85 | 93.04 |
| CS044 | 1,720,941 | 432,222,816 | 41.49 | 93.54 |
| CS045 | 3,042,573 | 765,923,354 | 39.64 | 93.08 |
| CS046 | 2,931,370 | 737,947,456 | 38.96 | 92.66 |
| CS047 | 2,591,688 | 651,853,918 | 39.57 | 92.77 |
| CS049 | 1,996,802 | 502,049,146 | 41.72 | 93.55 |
| CS052 | 1,964,439 | 493,023,462 | 42.35 | 93.59 |
| CS053 | 3,264,546 | 821,642,224 | 38.98 | 93.15 |
| CS056 | 2,296,608 | 577,652,764 | 39.54 | 93.03 |
| CS061 | 2,699,856 | 679,346,850 | 39.88 | 93.14 |
| CS062 | 1,940,483 | 487,865,852 | 41.62 | 92.56 |
| CS065 | 2,033,369 | 510,634,836 | 41.86 | 92.77 |
| CS068 | 3,043,249 | 765,839,720 | 39.65 | 93.29 |
| CS073 | 1,843,584 | 462,838,442 | 40.70 | 92.62 |
| CS077 | 2,834,352 | 712,860,586 | 40.62 | 93.71 |
| CS078 | 2,821,952 | 710,259,614 | 39.60 | 93.34 |
| CS080 | 1,529,592 | 384,160,242 | 41.12 | 93.11 |
| CS081 | 1,559,872 | 391,866,918 | 41.29 | 93.36 |
| CS087 | 2,213,266 | 556,876,502 | 40.27 | 93.10 |
| CS095 | 3,226,211 | 812,195,500 | 40.31 | 92.81 |
| CS096 | 3,079,242 | 775,012,064 | 40.56 | 93.44 |
| CS097 | 1,823,380 | 458,282,098 | 40.74 | 93.60 |
| CS098 | 1,552,937 | 390,160,114 | 41.03 | 93.90 |
| CS099 | 2,655,202 | 668,662,246 | 39.12 | 92.72 |
| CS102 | 2,478,618 | 623,682,994 | 39.07 | 93.15 |
| CS104 | 3,201,378 | 805,978,432 | 39.57 | 93.54 |
| CS105 | 2,491,311 | 626,975,096 | 39.49 | 93.37 |
| CS106 | 2,497,533 | 628,378,756 | 40.46 | 93.58 |
| CS108 | 2,608,456 | 656,910,364 | 39.59 | 93.27 |
| CS109 | 2,856,920 | 719,182,462 | 39.33 | 92.86 |
| CS110 | 3,074,323 | 773,843,424 | 39.34 | 93.09 |
| CS111 | 3,598,768 | 905,991,206 | 39.30 | 92.99 |
| CS114 | 1,877,472 | 472,204,542 | 41.06 | 93.46 |
| CS116 | 1,403,877 | 352,867,414 | 40.44 | 93.62 |
| CS117 | 2,695,443 | 678,640,342 | 39.42 | 93.54 |
| CS118 | 1,445,779 | 363,596,438 | 41.93 | 94.48 |
| CS119 | 3,119,822 | 785,455,304 | 39.71 | 93.27 |
| CS120 | 2,193,839 | 551,231,012 | 40.97 | 93.60 |
| CS121 | 2,095,457 | 527,038,132 | 40.48 | 93.34 |
| CS123 | 2,496,523 | 628,650,418 | 39.37 | 93.12 |
| CS126 | 2,834,825 | 713,399,162 | 39.37 | 92.78 |
| CS127 | 2,882,467 | 725,300,464 | 39.90 | 92.97 |
| CS130 | 2,135,573 | 536,896,924 | 41.16 | 93.87 |
| CS131 | 1,471,505 | 369,111,182 | 42.23 | 93.36 |
| CS134 | 3,417,887 | 860,494,268 | 39.96 | 93.10 |
| CS135 | 2,594,593 | 653,314,102 | 38.92 | 92.86 |
| CS137 | 2,823,580 | 710,965,586 | 38.62 | 92.19 |
| CS138 | 3,723,082 | 937,518,638 | 39.70 | 93.60 |
| CS139 | 2,265,237 | 569,580,620 | 41.40 | 93.24 |
| CS140 | 2,237,192 | 563,219,824 | 40.81 | 93.15 |
| CS141 | 2,856,338 | 719,046,038 | 39.36 | 93.18 |
| CS144 | 2,999,835 | 755,049,174 | 41.10 | 93.05 |
| CS150 | 2,595,010 | 652,783,214 | 40.68 | 93.27 |
| CS154 | 1,560,004 | 392,738,314 | 39.81 | 92.72 |
| CS155 | 2,013,446 | 506,737,416 | 39.29 | 93.32 |
| CS157 | 2,521,916 | 634,855,718 | 41.21 | 92.90 |
| CS158 | 2,477,065 | 623,522,398 | 41.08 | 93.32 |
| CS161 | 1,094,308 | 274,332,518 | 42.60 | 92.96 |
| CS162 | 2,386,295 | 600,544,684 | 39.50 | 93.33 |
| CS163 | 1,821,414 | 458,079,260 | 40.00 | 92.64 |
| CS164 | 3,626,636 | 912,751,442 | 40.09 | 93.24 |
| CS166 | 1,624,895 | 408,342,906 | 41.35 | 93.40 |
| CS174 | 1,584,320 | 397,935,978 | 41.43 | 93.06 |
| CS175 | 2,460,781 | 619,601,202 | 38.78 | 92.34 |
| CS176 | 1,849,731 | 465,591,590 | 39.10 | 93.09 |
| CS177 | 2,124,655 | 534,115,906 | 40.03 | 93.27 |
| CS179 | 2,155,265 | 542,750,138 | 40.24 | 92.61 |
| CS181 | 1,779,219 | 447,281,636 | 39.05 | 93.39 |
| CS182 | 2,931,645 | 738,120,082 | 39.91 | 92.96 |
| CS185 | 2,476,807 | 623,496,194 | 39.09 | 91.87 |
| CS191 | 1,880,946 | 472,955,756 | 39.66 | 93.37 |
| CS192 | 1,136,416 | 285,133,622 | 42.38 | 93.13 |
| CS193 | 1,258,697 | 315,347,686 | 42.06 | 93.53 |
| CS194 | 3,019,230 | 760,204,798 | 39.86 | 93.62 |
| CS197 | 1,746,918 | 439,153,158 | 40.72 | 93.71 |
| CS200 | 2,554,877 | 643,175,096 | 39.03 | 92.44 |
| CS201 | 2,893,547 | 728,400,378 | 39.97 | 93.51 |
| CS202 | 1,669,861 | 419,774,268 | 42.12 | 93.43 |
| CS203 | 1,634,347 | 410,692,292 | 41.28 | 93.42 |
| CS210 | 1,358,338 | 341,964,444 | 39.14 | 93.42 |
| CS212 | 2,116,105 | 532,324,898 | 39.41 | 93.30 |
| CS213 | 2,578,486 | 649,314,320 | 39.64 | 93.68 |
| CS214 | 2,075,841 | 522,342,724 | 39.05 | 92.85 |
| CS217 | 2,703,969 | 680,658,588 | 39.70 | 93.13 |
| CS218 | 2,657,194 | 668,743,904 | 40.43 | 93.01 |
| CS219 | 2,422,293 | 609,876,060 | 39.43 | 93.04 |
| CS221 | 1,950,500 | 491,110,574 | 38.92 | 93.45 |
| CS226 | 3,638,389 | 915,371,672 | 39.32 | 93.03 |
| CS231 | 1,818,331 | 456,832,626 | 41.50 | 92.45 |
| CS233 | 2,245,782 | 565,433,374 | 40.35 | 93.54 |
| CS234 | 2,674,295 | 673,488,426 | 39.44 | 91.61 |
| CS236 | 2,964,774 | 746,454,688 | 38.93 | 93.23 |
| CS241 | 3,440,875 | 866,473,262 | 39.93 | 93.60 |
| CS243 | 1,403,281 | 352,497,074 | 42.04 | 92.96 |
| CS248 | 2,062,366 | 517,919,950 | 39.15 | 93.56 |
| CS256 | 2,331,308 | 586,270,946 | 40.33 | 93.73 |
| CS257 | 1,195,902 | 299,824,490 | 42.02 | 93.90 |
| CS260 | 1,585,100 | 397,809,602 | 41.11 | 93.34 |
| CS262 | 2,901,460 | 729,961,408 | 41.06 | 93.93 |
| CS263 | 2,614,670 | 658,394,114 | 39.22 | 93.33 |
| CS267 | 2,038,320 | 512,790,664 | 39.00 | 93.01 |
| CS268 | 3,174,552 | 799,351,966 | 39.63 | 93.32 |
| CS271 | 2,004,138 | 503,713,658 | 41.93 | 93.81 |
| CS272 | 1,919,187 | 482,001,072 | 42.14 | 93.59 |
| CS276 | 4,064,758 | 1,023,377,480 | 39.70 | 93.31 |
| CS277 | 3,071,058 | 772,904,232 | 39.26 | 92.76 |
| CS280 | 2,739,140 | 689,758,220 | 39.43 | 93.43 |
| CS281 | 2,579,324 | 648,688,176 | 42.36 | 94.02 |
| CS282 | 2,269,742 | 570,104,686 | 42.03 | 93.66 |
| CS283 | 2,469,304 | 621,152,390 | 41.28 | 93.77 |
| CS284 | 2,775,864 | 698,792,030 | 38.69 | 92.80 |
| CS285 | 2,833,677 | 712,924,492 | 39.04 | 93.12 |
| CS286 | 2,958,533 | 744,798,560 | 39.71 | 93.06 |
| CS287 | 2,463,968 | 620,041,864 | 40.45 | 93.62 |
| CS288 | 1,578,792 | 395,857,092 | 41.98 | 93.50 |
| CS295 | 2,515,749 | 633,429,912 | 39.18 | 93.01 |
| CS300 | 4,702,430 | 1,184,016,248 | 40.18 | 92.83 |
| CS302 | 1,749,954 | 439,140,752 | 41.62 | 93.16 |
| CS303 | 2,458,626 | 618,240,616 | 41.72 | 94.27 |
| CS304 | 2,500,729 | 629,538,914 | 39.32 | 93.25 |
| CS306 | 2,504,234 | 629,784,756 | 39.23 | 93.15 |
| CS307 | 3,031,468 | 762,939,316 | 40.28 | 92.40 |
| CS310 | 1,688,127 | 423,990,822 | 41.53 | 93.36 |
| CS312 | 2,822,404 | 710,490,836 | 39.36 | 92.53 |
| CS313 | 3,344,462 | 841,855,598 | 40.30 | 93.35 |
| CS317 | 2,383,638 | 600,102,018 | 39.27 | 93.43 |
| CS318 | 2,290,979 | 576,078,850 | 39.37 | 92.85 |
| CS320 | 2,937,160 | 739,626,764 | 39.25 | 93.19 |
| CS321 | 1,352,534 | 338,948,646 | 41.76 | 93.55 |
| CS324 | 1,318,366 | 330,817,720 | 41.36 | 93.59 |
| CS325 | 2,191,727 | 551,375,988 | 41.34 | 93.47 |
| CS327 | 3,358,516 | 844,504,478 | 40.75 | 92.37 |
| CS328 | 1,166,650 | 292,271,466 | 41.83 | 92.88 |
| CS329 | 2,222,232 | 558,851,424 | 41.50 | 93.52 |
| CS330 | 1,517,731 | 380,557,826 | 42.22 | 92.79 |
| CS331 | 2,394,015 | 602,338,302 | 39.69 | 92.62 |
| CS333 | 3,287,539 | 827,539,568 | 39.69 | 93.47 |
| CS336 | 2,880,844 | 725,323,594 | 39.37 | 93.34 |
| CS339 | 1,404,505 | 352,899,308 | 41.59 | 93.22 |
| CS342 | 2,249,846 | 564,994,404 | 41.71 | 93.75 |
| CS343 | 2,696,017 | 677,931,686 | 39.82 | 93.40 |
| CS345 | 2,594,123 | 652,808,510 | 39.37 | 93.11 |
| CS346 | 2,165,111 | 544,502,506 | 40.42 | 93.47 |
| CS347 | 2,256,805 | 567,635,694 | 42.13 | 93.50 |
| CS348 | 2,516,436 | 632,809,202 | 40.86 | 93.67 |
| CS349 | 2,885,254 | 725,870,586 | 39.75 | 93.21 |
| CS352 | 2,358,219 | 592,652,438 | 39.64 | 92.86 |
| CS357 | 1,981,659 | 497,756,802 | 40.44 | 92.42 |
| CS371 | 2,005,474 | 503,754,500 | 41.50 | 93.75 |
| Average in F1 progeny | 2,352,007 | 591,641,374 | 40.34 | 93.22 |
| Sum in F1 progeny | 397,489,135 | 99,987,392,178 | 6817.82 | 15753.80 |
