# Supplementary material for: Construction of a High-Density Genetic Map and Identification of Quantitative Trait Loci Linked to Fruit Quality Traits in Apricots Using Specific-Locus Amplified Fragment Sequencing
Source: Front Plant Sci. 2022 Feb 14;13:798700. doi: 10.3389/fpls.2022.798700 (PMC8882730; doi:10.3389/fpls.2022.798700)
Supplement: Supplementary file 3 [file Table_3.DOCX]

**Supplementary Table 3. Details quality information of the reads for SLAFs in this study**

| Type | SNP number | Filtered SNP number |
| --- | --- | --- |
| Total marker | 1,388,415 |  |
| Parent marker lack | 975,321 | - |
| Sequcing depth is less than 4 in parents | 150,613 | - |
| Nopoly marker | 140,340 | - |
| Remain marker | 122,141 | 6,012 |
| aaxbb | 24,465 | - |
| abxcc | 121 | - |
| abxcd | - | - |
| ccxab | 170 | - |
| efxeg | 454 | 6 |
| hkxhk | 14,377 | 504 |
| lmxll | 44,958 | 3,178 |
| nnxnp | 37,596 | 2,324 |
