# Supplementary material for: Construction of a High-Density Genetic Map and Identification of Quantitative Trait Loci Linked to Fruit Quality Traits in Apricots Using Specific-Locus Amplified Fragment Sequencing
Source: Front Plant Sci. 2022 Feb 14;13:798700. doi: 10.3389/fpls.2022.798700 (PMC8882730; doi:10.3389/fpls.2022.798700)
Supplement: Supplementary file 4 [file Table_4.docx]

**Supplementary Table 4. Details genetic distance of mapped markers on linkage maps**

| 'Chuanzhihong' linkage map | | |  | 'Saimaiti' linkage map | | |
| --- | --- | --- | --- | --- | --- | --- |
| SNP marker | Group | Postion |  | SNP marker | Group | Postion |
| Marker15093 | Hg1 | 0.0 |  | Marker35794 | Sg1 | 0.0 |
| Marker14873 | Hg1 | 5.7 |  | Marker36400 | Sg1 | 2.3 |
| Marker14756 | Hg1 | 7.0 |  | Marker35650 | Sg1 | 9.4 |
| Marker17423 | Hg1 | 7.9 |  | Marker35667 | Sg1 | 10.9 |
| Marker14998 | Hg1 | 9.0 |  | Marker35763 | Sg1 | 13.5 |
| Marker16278 | Hg1 | 9.5 |  | Marker35678 | Sg1 | 16.0 |
| Marker15476 | Hg1 | 10.4 |  | Marker35782 | Sg1 | 17.2 |
| Marker15684 | Hg1 | 10.8 |  | Marker35733 | Sg1 | 18.6 |
| Marker116130 | Hg1 | 10.9 |  | Marker36091 | Sg1 | 19.6 |
| Marker14759 | Hg1 | 11.2 |  | Marker35217 | Sg1 | 21.3 |
| Marker15926 | Hg1 | 11.7 |  | Marker35261 | Sg1 | 22.2 |
| Marker15102 | Hg1 | 12.2 |  | Marker36187 | Sg1 | 24.4 |
| Marker15306 | Hg1 | 12.7 |  | Marker36153 | Sg1 | 25.5 |
| Marker15435 | Hg1 | 13.0 |  | Marker34693 | Sg1 | 26.2 |
| Marker15130 | Hg1 | 13.3 |  | Marker36231 | Sg1 | 26.5 |
| Marker14962 | Hg1 | 13.9 |  | Marker35975 | Sg1 | 27.7 |
| Marker16031 | Hg1 | 14.4 |  | Marker34806 | Sg1 | 28.4 |
| Marker15431 | Hg1 | 14.8 |  | Marker35192 | Sg1 | 29.6 |
| Marker16293 | Hg1 | 14.9 |  | Marker34849 | Sg1 | 31.2 |
| Marker15174 | Hg1 | 15.5 |  | Marker34837 | Sg1 | 32.6 |
| Marker16115 | Hg1 | 15.9 |  | Marker34207 | Sg1 | 33.8 |
| Marker15793 | Hg1 | 16.1 |  | Marker34786 | Sg1 | 34.5 |
| Marker15792 | Hg1 | 16.2 |  | Marker34963 | Sg1 | 35.4 |
| Marker15661 | Hg1 | 16.8 |  | Marker34918 | Sg1 | 37.1 |
| Marker15432 | Hg1 | 17.1 |  | Marker34327 | Sg1 | 37.7 |
| Marker16066 | Hg1 | 17.5 |  | Marker33669 | Sg1 | 38.9 |
| Marker16194 | Hg1 | 17.6 |  | Marker34105 | Sg1 | 42.2 |
| Marker16574 | Hg1 | 18.0 |  | Marker33892 | Sg1 | 43.3 |
| Marker16671 | Hg1 | 18.7 |  | Marker33955 | Sg1 | 44.5 |
| Marker16739 | Hg1 | 19.4 |  | Marker33884 | Sg1 | 45.4 |
| Marker17680 | Hg1 | 19.9 |  | Marker32745 | Sg1 | 47.8 |
| Marker17510 | Hg1 | 20.5 |  | Marker32789 | Sg1 | 48.8 |
| Marker17144 | Hg1 | 20.9 |  | Marker32253 | Sg1 | 49.5 |
| Marker16902 | Hg1 | 21.4 |  | Marker32613 | Sg1 | 51.1 |
| Marker17021 | Hg1 | 21.4 |  | Marker32873 | Sg1 | 52.9 |
| Marker16852 | Hg1 | 21.9 |  | Marker32122 | Sg1 | 53.9 |
| Marker17502 | Hg1 | 22.2 |  | Marker32060 | Sg1 | 55.0 |
| Marker17233 | Hg1 | 22.5 |  | Marker32811 | Sg1 | 55.8 |
| Marker16886 | Hg1 | 22.8 |  | Marker31241 | Sg1 | 56.4 |
| Marker17824 | Hg1 | 23.3 |  | Marker30909 | Sg1 | 57.2 |
| Marker18910 | Hg1 | 23.8 |  | Marker32239 | Sg1 | 57.5 |
| Marker17554 | Hg1 | 24.2 |  | Marker31701 | Sg1 | 58.3 |
| Marker19006 | Hg1 | 24.6 |  | Marker31162 | Sg1 | 59.7 |
| Marker75552 | Hg1 | 25.0 |  | Marker30773 | Sg1 | 60.3 |
| Marker19345 | Hg1 | 25.1 |  | Marker30451 | Sg1 | 60.9 |
| Marker18995 | Hg1 | 25.4 |  | Marker30328 | Sg1 | 61.3 |
| Marker75364 | Hg1 | 25.8 |  | Marker30222 | Sg1 | 62.1 |
| Marker18254 | Hg1 | 26.0 |  | Marker30589 | Sg1 | 62.5 |
| Marker17877 | Hg1 | 26.4 |  | Marker30710 | Sg1 | 63.1 |
| Marker118301 | Hg1 | 26.7 |  | Marker30669 | Sg1 | 63.6 |
| Marker19249 | Hg1 | 27.3 |  | Marker30521 | Sg1 | 64.4 |
| Marker75437 | Hg1 | 27.6 |  | Marker30337 | Sg1 | 64.9 |
| Marker75593 | Hg1 | 27.8 |  | Marker30508 | Sg1 | 65.4 |
| Marker19084 | Hg1 | 28.5 |  | Marker30931 | Sg1 | 66.0 |
| Marker19364 | Hg1 | 28.7 |  | Marker31826 | Sg1 | 66.6 |
| Marker19089 | Hg1 | 29.2 |  | Marker30250 | Sg1 | 67.1 |
| Marker19042 | Hg1 | 29.5 |  | Marker30248 | Sg1 | 67.5 |
| Marker19076 | Hg1 | 30.0 |  | Marker30808 | Sg1 | 68.1 |
| Marker18806 | Hg1 | 30.6 |  | Marker63223 | Sg1 | 68.8 |
| Marker19052 | Hg1 | 31.1 |  | Marker31742 | Sg1 | 69.4 |
| Marker18000 | Hg1 | 31.4 |  | Marker27019 | Sg1 | 70.2 |
| Marker18567 | Hg1 | 31.8 |  | Marker27727 | Sg1 | 70.6 |
| Marker18609 | Hg1 | 32.2 |  | Marker29086 | Sg1 | 70.8 |
| Marker18716 | Hg1 | 32.7 |  | Marker28348 | Sg1 | 71.3 |
| Marker18559 | Hg1 | 33.0 |  | Marker28867 | Sg1 | 71.7 |
| Marker18739 | Hg1 | 33.3 |  | Marker30735 | Sg1 | 72.1 |
| Marker120358 | Hg1 | 34.0 |  | Marker30173 | Sg1 | 72.4 |
| Marker117268 | Hg1 | 34.9 |  | Marker30268 | Sg1 | 72.8 |
| Marker18515 | Hg1 | 35.2 |  | Marker30256 | Sg1 | 73.2 |
| Marker18459 | Hg1 | 35.5 |  | Marker28573 | Sg1 | 73.8 |
| Marker19896 | Hg1 | 35.8 |  | Marker29192 | Sg1 | 74.3 |
| Marker19879 | Hg1 | 36.3 |  | Marker29449 | Sg1 | 74.8 |
| Marker19841 | Hg1 | 36.6 |  | Marker28327 | Sg1 | 75.2 |
| Marker19838 | Hg1 | 37.2 |  | Marker27552 | Sg1 | 75.7 |
| Marker19837 | Hg1 | 37.3 |  | Marker120567 | Sg1 | 76.5 |
| Marker20120 | Hg1 | 38.0 |  | Marker29055 | Sg1 | 77.4 |
| Marker19658 | Hg1 | 38.4 |  | Marker112929 | Sg1 | 77.9 |
| Marker20344 | Hg1 | 39.1 |  | Marker112862 | Sg1 | 78.2 |
| Marker20022 | Hg1 | 39.2 |  | Marker29523 | Sg1 | 78.6 |
| Marker19574 | Hg1 | 39.7 |  | Marker29412 | Sg1 | 79.1 |
| Marker20282 | Hg1 | 39.9 |  | Marker28574 | Sg1 | 79.7 |
| Marker20094 | Hg1 | 40.3 |  | Marker29387 | Sg1 | 80.0 |
| Marker19554 | Hg1 | 40.6 |  | Marker28284 | Sg1 | 80.3 |
| Marker20524 | Hg1 | 41.0 |  | Marker29410 | Sg1 | 80.8 |
| Marker20447 | Hg1 | 41.6 |  | Marker27686 | Sg1 | 81.0 |
| Marker20824 | Hg1 | 41.9 |  | Marker26518 | Sg1 | 81.4 |
| Marker20613 | Hg1 | 42.4 |  | Marker29516 | Sg1 | 81.8 |
| Marker21236 | Hg1 | 42.7 |  | Marker27085 | Sg1 | 82.5 |
| Marker20782 | Hg1 | 43.1 |  | Marker85230 | Sg1 | 82.9 |
| Marker20700 | Hg1 | 43.5 |  | Marker27028 | Sg1 | 83.2 |
| Marker21108 | Hg1 | 43.8 |  | Marker27483 | Sg1 | 83.7 |
| Marker20678 | Hg1 | 44.1 |  | Marker85292 | Sg1 | 84.2 |
| Marker21076 | Hg1 | 44.4 |  | Marker27233 | Sg1 | 84.4 |
| Marker20955 | Hg1 | 44.7 |  | Marker26606 | Sg1 | 86.1 |
| Marker20897 | Hg1 | 45.2 |  | Marker26500 | Sg1 | 86.3 |
| Marker21139 | Hg1 | 45.8 |  | Marker27960 | Sg1 | 86.7 |
| Marker20676 | Hg1 | 46.4 |  | Marker26688 | Sg1 | 87.4 |
| Marker20903 | Hg1 | 46.7 |  | Marker28073 | Sg1 | 88.5 |
| Marker20773 | Hg1 | 46.9 |  | Marker26541 | Sg1 | 89.3 |
| Marker21208 | Hg1 | 47.2 |  | Marker26393 | Sg1 | 90.0 |
| Marker21094 | Hg1 | 47.7 |  | Marker26794 | Sg1 | 90.3 |
| Marker21943 | Hg1 | 48.2 |  | Marker26164 | Sg1 | 90.8 |
| Marker21982 | Hg1 | 48.6 |  | Marker26928 | Sg1 | 91.6 |
| Marker22118 | Hg1 | 48.7 |  | Marker26764 | Sg1 | 91.7 |
| Marker21680 | Hg1 | 49.0 |  | Marker26036 | Sg1 | 92.5 |
| Marker21220 | Hg1 | 49.4 |  | Marker26002 | Sg1 | 92.9 |
| Marker21052 | Hg1 | 49.8 |  | Marker25975 | Sg1 | 93.5 |
| Marker21333 | Hg1 | 50.3 |  | Marker25192 | Sg1 | 94.0 |
| Marker21929 | Hg1 | 50.5 |  | Marker25086 | Sg1 | 94.8 |
| Marker21297 | Hg1 | 51.0 |  | Marker26207 | Sg1 | 95.3 |
| Marker21972 | Hg1 | 51.3 |  | Marker24691 | Sg1 | 95.8 |
| Marker22207 | Hg1 | 52.1 |  | Marker24846 | Sg1 | 96.4 |
| Marker23473 | Hg1 | 52.9 |  | Marker26789 | Sg1 | 97.4 |
| Marker21306 | Hg1 | 53.2 |  | Marker119251 | Sg1 | 97.7 |
| Marker21787 | Hg1 | 53.5 |  | Marker25042 | Sg1 | 98.3 |
| Marker21912 | Hg1 | 54.2 |  | Marker24930 | Sg1 | 98.8 |
| Marker22052 | Hg1 | 54.7 |  | Marker24791 | Sg1 | 99.6 |
| Marker23570 | Hg1 | 55.3 |  | Marker23760 | Sg1 | 100.8 |
| Marker23137 | Hg1 | 55.4 |  | Marker24421 | Sg1 | 101.7 |
| Marker22214 | Hg1 | 56.1 |  | Marker116085 | Sg1 | 102.3 |
| Marker23626 | Hg1 | 56.5 |  | Marker24634 | Sg1 | 104.0 |
| Marker22346 | Hg1 | 56.7 |  | Marker24460 | Sg1 | 104.7 |
| Marker23554 | Hg1 | 57.1 |  | Marker24505 | Sg1 | 105.1 |
| Marker22392 | Hg1 | 57.6 |  | Marker24192 | Sg1 | 105.8 |
| Marker22251 | Hg1 | 58.0 |  | Marker24120 | Sg1 | 107.1 |
| Marker22446 | Hg1 | 58.2 |  | Marker110396 | Sg1 | 108.1 |
| Marker23583 | Hg1 | 58.5 |  | Marker23955 | Sg1 | 109.3 |
| Marker23413 | Hg1 | 59.2 |  | Marker110302 | Sg1 | 111.8 |
| Marker22645 | Hg1 | 59.5 |  | Marker110216 | Sg1 | 112.8 |
| Marker22814 | Hg1 | 59.7 |  | Marker108443 | Sg1 | 113.4 |
| Marker23335 | Hg1 | 60.2 |  | Marker108529 | Sg1 | 114.7 |
| Marker22776 | Hg1 | 61.0 |  | Marker108608 | Sg1 | 116.4 |
| Marker22789 | Hg1 | 61.4 |  | Marker108653 | Sg1 | 116.6 |
| Marker23026 | Hg1 | 61.8 |  | Marker22475 | Sg1 | 117.2 |
| Marker22741 | Hg1 | 62.0 |  | Marker23644 | Sg1 | 118.0 |
| Marker22696 | Hg1 | 62.3 |  | Marker23130 | Sg1 | 118.9 |
| Marker22853 | Hg1 | 62.5 |  | Marker108722 | Sg1 | 119.6 |
| Marker23016 | Hg1 | 62.8 |  | Marker22493 | Sg1 | 120.7 |
| Marker108308 | Hg1 | 63.5 |  | Marker108772 | Sg1 | 121.5 |
| Marker22855 | Hg1 | 63.7 |  | Marker22718 | Sg1 | 122.2 |
| Marker108384 | Hg1 | 64.1 |  | Marker22712 | Sg1 | 122.2 |
| Marker22718 | Hg1 | 64.5 |  | Marker22855 | Sg1 | 123.3 |
| Marker108680 | Hg1 | 64.8 |  | Marker22801 | Sg1 | 123.9 |
| Marker23259 | Hg1 | 65.1 |  | Marker23016 | Sg1 | 124.6 |
| Marker22763 | Hg1 | 65.9 |  | Marker22758 | Sg1 | 124.8 |
| Marker22475 | Hg1 | 66.2 |  | Marker23030 | Sg1 | 125.9 |
| Marker22893 | Hg1 | 66.4 |  | Marker23494 | Sg1 | 127.2 |
| Marker23060 | Hg1 | 66.9 |  | Marker23174 | Sg1 | 127.7 |
| Marker22453 | Hg1 | 67.2 |  | Marker23439 | Sg1 | 127.9 |
| Marker110419 | Hg1 | 67.7 |  | Marker23413 | Sg1 | 128.3 |
| Marker23957 | Hg1 | 68.7 |  | Marker22768 | Sg1 | 128.9 |
| Marker23130 | Hg1 | 68.8 |  | Marker23626 | Sg1 | 130.1 |
| Marker24035 | Hg1 | 69.3 |  | Marker22917 | Sg1 | 130.7 |
| Marker25935 | Hg1 | 69.7 |  | Marker22446 | Sg1 | 131.4 |
| Marker25355 | Hg1 | 70.5 |  | Marker22212 | Sg1 | 131.6 |
| Marker24226 | Hg1 | 70.9 |  | Marker22392 | Sg1 | 132.2 |
| Marker83220 | Hg1 | 71.9 |  | Marker23021 | Sg1 | 132.5 |
| Marker24384 | Hg1 | 72.1 |  | Marker22970 | Sg1 | 133.0 |
| Marker25861 | Hg1 | 72.7 |  | Marker23389 | Sg1 | 133.5 |
| Marker25463 | Hg1 | 73.2 |  | Marker23570 | Sg1 | 134.1 |
| Marker24511 | Hg1 | 73.9 |  | Marker22116 | Sg1 | 134.7 |
| Marker24460 | Hg1 | 73.9 |  | Marker22268 | Sg1 | 135.3 |
| Marker25524 | Hg1 | 74.5 |  | Marker22789 | Sg1 | 135.7 |
| Marker108227 | Hg1 | 75.0 |  | Marker23592 | Sg1 | 136.5 |
| Marker25336 | Hg1 | 75.8 |  | Marker23575 | Sg1 | 137.5 |
| Marker25975 | Hg1 | 76.5 |  | Marker23629 | Sg1 | 138.1 |
| Marker25609 | Hg1 | 76.5 |  | Marker21949 | Sg1 | 138.9 |
| Marker26020 | Hg1 | 77.0 |  | Marker21982 | Sg1 | 139.4 |
| Marker26928 | Hg1 | 77.2 |  | Marker22042 | Sg1 | 139.9 |
| Marker28434 | Hg1 | 77.5 |  | Marker21929 | Sg1 | 140.2 |
| Marker25196 | Hg1 | 78.2 |  | Marker21926 | Sg1 | 140.8 |
| Marker26789 | Hg1 | 78.5 |  | Marker22177 | Sg1 | 141.9 |
| Marker85738 | Hg1 | 79.0 |  | Marker21702 | Sg1 | 143.4 |
| Marker26754 | Hg1 | 79.3 |  | Marker21488 | Sg1 | 144.4 |
| Marker25161 | Hg1 | 79.8 |  | Marker21575 | Sg1 | 145.2 |
| Marker26207 | Hg1 | 80.0 |  | Marker21181 | Sg1 | 146.7 |
| Marker25082 | Hg1 | 80.5 |  | Marker21306 | Sg1 | 147.9 |
| Marker26217 | Hg1 | 81.0 |  | Marker21409 | Sg1 | 148.5 |
| Marker27727 | Hg1 | 81.4 |  | Marker20547 | Sg1 | 149.5 |
| Marker25241 | Hg1 | 81.8 |  | Marker20824 | Sg1 | 149.8 |
| Marker29619 | Hg1 | 82.2 |  | Marker21328 | Sg1 | 150.2 |
| Marker26314 | Hg1 | 83.1 |  | Marker21132 | Sg1 | 151.2 |
| Marker112862 | Hg1 | 83.3 |  | Marker20782 | Sg1 | 151.6 |
| Marker27518 | Hg1 | 83.6 |  | Marker20951 | Sg1 | 152.1 |
| Marker26555 | Hg1 | 84.2 |  | Marker20741 | Sg1 | 152.5 |
| Marker27747 | Hg1 | 84.8 |  | Marker21220 | Sg1 | 152.9 |
| Marker29410 | Hg1 | 84.9 |  | Marker20344 | Sg1 | 153.7 |
| Marker29081 | Hg1 | 85.3 |  | Marker20339 | Sg1 | 154.1 |
| Marker28327 | Hg1 | 85.9 |  | Marker20282 | Sg1 | 154.2 |
| Marker27243 | Hg1 | 86.2 |  | Marker120364 | Sg1 | 154.7 |
| Marker85285 | Hg1 | 86.3 |  | Marker20278 | Sg1 | 155.3 |
| Marker28584 | Hg1 | 86.8 |  | Marker20120 | Sg1 | 156.1 |
| Marker28532 | Hg1 | 87.1 |  | Marker19789 | Sg1 | 156.3 |
| Marker28573 | Hg1 | 87.4 |  | Marker20379 | Sg1 | 156.7 |
| Marker28050 | Hg1 | 87.9 |  | Marker20201 | Sg1 | 157.2 |
| Marker27028 | Hg1 | 88.2 |  | Marker21107 | Sg1 | 157.5 |
| Marker29767 | Hg1 | 88.8 |  | Marker21089 | Sg1 | 157.8 |
| Marker26518 | Hg1 | 89.3 |  | Marker20692 | Sg1 | 159.0 |
| Marker30154 | Hg1 | 90.1 |  | Marker20678 | Sg1 | 159.4 |
| Marker30299 | Hg1 | 90.5 |  | Marker21062 | Sg1 | 160.0 |
| Marker30214 | Hg1 | 91.0 |  | Marker20903 | Sg1 | 160.5 |
| Marker30250 | Hg1 | 91.7 |  | Marker19556 | Sg1 | 160.9 |
| Marker30386 | Hg1 | 91.9 |  | Marker20053 | Sg1 | 162.2 |
| Marker30145 | Hg1 | 92.6 |  | Marker20018 | Sg1 | 162.3 |
| Marker30735 | Hg1 | 92.8 |  | Marker20804 | Sg1 | 162.8 |
| Marker29945 | Hg1 | 92.9 |  | Marker19052 | Sg1 | 163.5 |
| Marker30521 | Hg1 | 93.5 |  | Marker19687 | Sg1 | 163.9 |
| Marker30266 | Hg1 | 93.8 |  | Marker19604 | Sg1 | 164.5 |
| Marker30694 | Hg1 | 94.5 |  | Marker19838 | Sg1 | 164.7 |
| Marker30508 | Hg1 | 94.9 |  | Marker18380 | Sg1 | 165.5 |
| Marker30808 | Hg1 | 95.6 |  | Marker19554 | Sg1 | 166.0 |
| Marker30886 | Hg1 | 95.7 |  | Marker20095 | Sg1 | 166.5 |
| Marker56177 | Hg1 | 96.5 |  | Marker20452 | Sg1 | 167.7 |
| Marker30990 | Hg1 | 97.0 |  | Marker18515 | Sg1 | 168.8 |
| Marker30222 | Hg1 | 97.8 |  | Marker19885 | Sg1 | 169.2 |
| Marker31254 | Hg1 | 98.2 |  | Marker18784 | Sg1 | 171.0 |
| Marker31124 | Hg1 | 99.3 |  | Marker18716 | Sg1 | 172.2 |
| Marker31065 | Hg1 | 100.1 |  | Marker19055 | Sg1 | 174.3 |
| Marker30909 | Hg1 | 100.5 |  | Marker19038 | Sg1 | 175.1 |
| Marker31903 | Hg1 | 101.9 |  | Marker19093 | Sg1 | 175.9 |
| Marker32239 | Hg1 | 102.3 |  | Marker19084 | Sg1 | 176.7 |
| Marker31781 | Hg1 | 102.8 |  | Marker19352 | Sg1 | 177.5 |
| Marker32797 | Hg1 | 103.4 |  | Marker75268 | Sg1 | 178.0 |
| Marker31723 | Hg1 | 104.1 |  | Marker75552 | Sg1 | 178.6 |
| Marker32064 | Hg1 | 104.8 |  | Marker18995 | Sg1 | 179.5 |
| Marker32272 | Hg1 | 105.6 |  | Marker75570 | Sg1 | 180.4 |
| Marker32055 | Hg1 | 106.0 |  | Marker18314 | Sg1 | 181.8 |
| Marker32770 | Hg1 | 106.7 |  | Marker17919 | Sg1 | 183.9 |
| Marker32035 | Hg1 | 107.1 |  | Marker18194 | Sg1 | 185.5 |
| Marker32537 | Hg1 | 107.5 |  | Marker17510 | Sg1 | 186.7 |
| Marker32426 | Hg1 | 108.1 |  | Marker18124 | Sg1 | 187.6 |
| Marker32362 | Hg1 | 108.8 |  | Marker17505 | Sg1 | 190.1 |
| Marker33075 | Hg1 | 109.2 |  | Marker17021 | Sg1 | 190.6 |
| Marker111940 | Hg1 | 109.6 |  | Marker17159 | Sg1 | 191.0 |
| Marker32902 | Hg1 | 110.0 |  | Marker17643 | Sg1 | 191.7 |
| Marker33220 | Hg1 | 110.4 |  | Marker17706 | Sg1 | 193.3 |
| Marker33171 | Hg1 | 111.2 |  | Marker17107 | Sg1 | 194.8 |
| Marker32939 | Hg1 | 112.1 |  | Marker16278 | Sg1 | 196.2 |
| Marker33297 | Hg1 | 112.7 |  | Marker16981 | Sg1 | 196.8 |
| Marker33892 | Hg1 | 113.0 |  | Marker16889 | Sg1 | 197.6 |
| Marker33353 | Hg1 | 113.2 |  | Marker16618 | Sg1 | 198.6 |
| Marker32962 | Hg1 | 113.8 |  | Marker16294 | Sg1 | 199.7 |
| Marker33955 | Hg1 | 114.2 |  | Marker16886 | Sg1 | 200.5 |
| Marker34057 | Hg1 | 114.6 |  | Marker16864 | Sg1 | 200.8 |
| Marker33499 | Hg1 | 115.0 |  | Marker16277 | Sg1 | 202.1 |
| Marker33411 | Hg1 | 115.8 |  | Marker15926 | Sg1 | 202.5 |
| Marker34090 | Hg1 | 116.2 |  | Marker16699 | Sg1 | 203.1 |
| Marker34145 | Hg1 | 116.9 |  | Marker16204 | Sg1 | 205.1 |
| Marker33669 | Hg1 | 117.3 |  | Marker16142 | Sg1 | 205.8 |
| Marker34918 | Hg1 | 117.8 |  | Marker16066 | Sg1 | 206.8 |
| Marker33883 | Hg1 | 118.2 |  | Marker15973 | Sg1 | 207.7 |
| Marker33733 | Hg1 | 118.9 |  | Marker15684 | Sg1 | 208.5 |
| Marker34786 | Hg1 | 119.4 |  | Marker15669 | Sg1 | 209.5 |
| Marker34279 | Hg1 | 119.7 |  | Marker15793 | Sg1 | 210.0 |
| Marker34979 | Hg1 | 120.4 |  | Marker15884 | Sg1 | 210.7 |
| Marker34837 | Hg1 | 121.1 |  | Marker15426 | Sg1 | 211.9 |
| Marker33822 | Hg1 | 121.4 |  | Marker15431 | Sg1 | 212.4 |
| Marker33712 | Hg1 | 121.7 |  | Marker14757 | Sg1 | 212.6 |
| Marker35172 | Hg1 | 122.4 |  | Marker15237 | Sg1 | 214.1 |
| Marker35192 | Hg1 | 122.5 |  | Marker15435 | Sg1 | 214.5 |
| Marker33943 | Hg1 | 123.1 |  | Marker15342 | Sg1 | 215.3 |
| Marker36231 | Hg1 | 123.8 |  | Marker14785 | Sg1 | 217.2 |
| Marker34754 | Hg1 | 124.0 |  | Marker15003 | Sg1 | 218.4 |
| Marker36204 | Hg1 | 124.5 |  | Marker15100 | Sg1 | 219.3 |
| Marker36153 | Hg1 | 124.8 |  | Marker14886 | Sg1 | 220.5 |
| Marker35034 | Hg1 | 125.3 |  | Marker15116 | Sg1 | 222.3 |
| Marker35918 | Hg1 | 125.8 |  | Marker15398 | Sg1 | 222.8 |
| Marker36187 | Hg1 | 126.1 |  | Marker14963 | Sg1 | 225.8 |
| Marker35480 | Hg1 | 126.7 |  | Marker14924 | Sg1 | 229.7 |
| Marker35655 | Hg1 | 127.3 |  | Marker8128 | Sg2 | 0.0 |
| Marker35975 | Hg1 | 127.4 |  | Marker8106 | Sg2 | 1.2 |
| Marker35770 | Hg1 | 128.4 |  | Marker120505 | Sg2 | 4.3 |
| Marker35668 | Hg1 | 129.0 |  | Marker65356 | Sg2 | 5.8 |
| Marker35261 | Hg1 | 129.4 |  | Marker63733 | Sg2 | 7.7 |
| Marker35730 | Hg1 | 129.6 |  | Marker64016 | Sg2 | 9.6 |
| Marker36718 | Hg1 | 130.1 |  | Marker64043 | Sg2 | 10.0 |
| Marker35310 | Hg1 | 130.6 |  | Marker63782 | Sg2 | 11.2 |
| Marker35733 | Hg1 | 131.5 |  | Marker64200 | Sg2 | 12.7 |
| Marker35789 | Hg1 | 131.9 |  | Marker64275 | Sg2 | 13.8 |
| Marker35064 | Hg1 | 133.0 |  | Marker63491 | Sg2 | 15.3 |
| Marker36323 | Hg1 | 133.6 |  | Marker63659 | Sg2 | 16.1 |
| Marker36091 | Hg1 | 134.0 |  | Marker63679 | Sg2 | 16.7 |
| Marker35619 | Hg1 | 135.3 |  | Marker64492 | Sg2 | 18.0 |
| Marker35420 | Hg1 | 137.1 |  | Marker64114 | Sg2 | 19.5 |
| Marker36929 | Hg1 | 138.7 |  | Marker121978 | Sg2 | 21.2 |
| Marker34945 | Hg1 | 139.7 |  | Marker65459 | Sg2 | 22.4 |
| Marker36799 | Hg1 | 141.1 |  | Marker121492 | Sg2 | 23.6 |
| Marker8120 | Hg2 | 0.0 |  | Marker65179 | Sg2 | 24.4 |
| Marker64041 | Hg2 | 1.7 |  | Marker119529 | Sg2 | 24.8 |
| Marker63686 | Hg2 | 3.2 |  | Marker64728 | Sg2 | 25.6 |
| Marker63731 | Hg2 | 4.2 |  | Marker64936 | Sg2 | 26.2 |
| Marker64634 | Hg2 | 5.1 |  | Marker65691 | Sg2 | 27.1 |
| Marker64393 | Hg2 | 5.9 |  | Marker64770 | Sg2 | 27.6 |
| Marker65427 | Hg2 | 6.9 |  | Marker65724 | Sg2 | 28.9 |
| Marker63679 | Hg2 | 7.3 |  | Marker66921 | Sg2 | 29.7 |
| Marker65153 | Hg2 | 8.4 |  | Marker65953 | Sg2 | 30.0 |
| Marker64114 | Hg2 | 8.6 |  | Marker64824 | Sg2 | 30.7 |
| Marker64985 | Hg2 | 9.0 |  | Marker65914 | Sg2 | 31.0 |
| Marker121293 | Hg2 | 10.2 |  | Marker65775 | Sg2 | 32.1 |
| Marker64728 | Hg2 | 10.6 |  | Marker121490 | Sg2 | 32.5 |
| Marker64741 | Hg2 | 11.3 |  | Marker67152 | Sg2 | 33.5 |
| Marker65953 | Hg2 | 12.9 |  | Marker67235 | Sg2 | 33.9 |
| Marker65696 | Hg2 | 13.2 |  | Marker66588 | Sg2 | 34.6 |
| Marker66701 | Hg2 | 13.8 |  | Marker89057 | Sg2 | 35.4 |
| Marker121490 | Hg2 | 14.6 |  | Marker66709 | Sg2 | 36.4 |
| Marker65743 | Hg2 | 15.4 |  | Marker67472 | Sg2 | 37.0 |
| Marker66828 | Hg2 | 16.1 |  | Marker67755 | Sg2 | 37.3 |
| Marker66142 | Hg2 | 16.5 |  | Marker67904 | Sg2 | 37.9 |
| Marker67691 | Hg2 | 17.9 |  | Marker17757 | Sg2 | 38.8 |
| Marker66436 | Hg2 | 18.3 |  | Marker66469 | Sg2 | 39.6 |
| Marker68245 | Hg2 | 19.3 |  | Marker66367 | Sg2 | 40.1 |
| Marker67963 | Hg2 | 20.2 |  | Marker115470 | Sg2 | 40.5 |
| Marker68600 | Hg2 | 21.3 |  | Marker67640 | Sg2 | 41.4 |
| Marker67936 | Hg2 | 22.4 |  | Marker68014 | Sg2 | 42.4 |
| Marker69780 | Hg2 | 23.2 |  | Marker69783 | Sg2 | 43.4 |
| Marker115367 | Hg2 | 23.9 |  | Marker68686 | Sg2 | 43.8 |
| Marker69380 | Hg2 | 25.1 |  | Marker26430 | Sg2 | 44.7 |
| Marker68821 | Hg2 | 25.5 |  | Marker67950 | Sg2 | 44.9 |
| Marker68841 | Hg2 | 26.1 |  | Marker68385 | Sg2 | 45.6 |
| Marker68722 | Hg2 | 26.4 |  | Marker68128 | Sg2 | 46.3 |
| Marker69069 | Hg2 | 27.0 |  | Marker68747 | Sg2 | 47.2 |
| Marker69000 | Hg2 | 27.8 |  | Marker68498 | Sg2 | 48.0 |
| Marker70461 | Hg2 | 28.5 |  | Marker16360 | Sg2 | 48.4 |
| Marker70318 | Hg2 | 29.4 |  | Marker16405 | Sg2 | 49.1 |
| Marker70592 | Hg2 | 30.0 |  | Marker68841 | Sg2 | 49.7 |
| Marker70656 | Hg2 | 30.8 |  | Marker68883 | Sg2 | 50.3 |
| Marker70810 | Hg2 | 31.2 |  | Marker69023 | Sg2 | 50.6 |
| Marker70777 | Hg2 | 31.3 |  | Marker68721 | Sg2 | 51.3 |
| Marker70821 | Hg2 | 32.2 |  | Marker96358 | Sg2 | 52.0 |
| Marker70573 | Hg2 | 32.7 |  | Marker69465 | Sg2 | 53.1 |
| Marker70252 | Hg2 | 33.2 |  | Marker69079 | Sg2 | 53.5 |
| Marker69509 | Hg2 | 33.7 |  | Marker69349 | Sg2 | 54.0 |
| Marker70940 | Hg2 | 34.6 |  | Marker120891 | Sg2 | 54.8 |
| Marker70914 | Hg2 | 35.4 |  | Marker69665 | Sg2 | 55.9 |
| Marker71937 | Hg2 | 37.0 |  | Marker69509 | Sg2 | 56.4 |
| Marker71125 | Hg2 | 37.7 |  | Marker70164 | Sg2 | 57.2 |
| Marker71106 | Hg2 | 38.5 |  | Marker69898 | Sg2 | 57.8 |
| Marker71325 | Hg2 | 39.0 |  | Marker70040 | Sg2 | 58.6 |
| Marker71139 | Hg2 | 39.8 |  | Marker70392 | Sg2 | 59.4 |
| Marker65646 | Hg2 | 40.2 |  | Marker70789 | Sg2 | 60.2 |
| Marker72029 | Hg2 | 40.9 |  | Marker70810 | Sg2 | 60.8 |
| Marker72007 | Hg2 | 41.3 |  | Marker70751 | Sg2 | 61.1 |
| Marker71907 | Hg2 | 41.9 |  | Marker70860 | Sg2 | 61.8 |
| Marker71568 | Hg2 | 42.3 |  | Marker121705 | Sg2 | 62.4 |
| Marker71544 | Hg2 | 42.8 |  | Marker70924 | Sg2 | 62.9 |
| Marker71948 | Hg2 | 43.2 |  | Marker70963 | Sg2 | 63.7 |
| Marker71606 | Hg2 | 43.6 |  | Marker70587 | Sg2 | 64.6 |
| Marker71844 | Hg2 | 44.6 |  | Marker71113 | Sg2 | 65.3 |
| Marker71749 | Hg2 | 45.1 |  | Marker71076 | Sg2 | 66.1 |
| Marker71817 | Hg2 | 46.1 |  | Marker71130 | Sg2 | 66.6 |
| Marker71791 | Hg2 | 46.6 |  | Marker71395 | Sg2 | 67.8 |
| Marker72174 | Hg2 | 48.2 |  | Marker72029 | Sg2 | 68.3 |
| Marker114288 | Hg2 | 49.6 |  | Marker71932 | Sg2 | 69.1 |
| Marker72435 | Hg2 | 50.5 |  | Marker71955 | Sg2 | 69.5 |
| Marker72335 | Hg2 | 51.2 |  | Marker71945 | Sg2 | 70.2 |
| Marker72634 | Hg2 | 52.0 |  | Marker71948 | Sg2 | 71.0 |
| Marker73124 | Hg2 | 53.9 |  | Marker71565 | Sg2 | 71.8 |
| Marker73046 | Hg2 | 54.9 |  | Marker71569 | Sg2 | 72.4 |
| Marker72948 | Hg2 | 55.5 |  | Marker71622 | Sg2 | 73.6 |
| Marker73107 | Hg2 | 55.8 |  | Marker71835 | Sg2 | 74.6 |
| Marker72973 | Hg2 | 56.5 |  | Marker71864 | Sg2 | 75.0 |
| Marker72806 | Hg2 | 57.2 |  | Marker71765 | Sg2 | 75.3 |
| Marker73201 | Hg2 | 57.7 |  | Marker71808 | Sg2 | 76.1 |
| Marker73512 | Hg2 | 58.2 |  | Marker72312 | Sg2 | 77.3 |
| Marker73188 | Hg2 | 58.6 |  | Marker121382 | Sg2 | 79.0 |
| Marker73486 | Hg2 | 59.2 |  | Marker72629 | Sg2 | 79.5 |
| Marker73483 | Hg2 | 59.8 |  | Marker73159 | Sg2 | 80.0 |
| Marker73266 | Hg2 | 60.4 |  | Marker72379 | Sg2 | 80.8 |
| Marker72769 | Hg2 | 61.4 |  | Marker72964 | Sg2 | 81.6 |
| Marker73476 | Hg2 | 62.0 |  | Marker72825 | Sg2 | 82.2 |
| Marker73264 | Hg2 | 62.2 |  | Marker72794 | Sg2 | 83.0 |
| Marker72949 | Hg2 | 62.5 |  | Marker72949 | Sg2 | 83.6 |
| Marker73401 | Hg2 | 62.9 |  | Marker114337 | Sg2 | 84.1 |
| Marker72745 | Hg2 | 63.9 |  | Marker111825 | Sg2 | 85.2 |
| Marker72677 | Hg2 | 64.5 |  | Marker73036 | Sg2 | 86.2 |
| Marker72835 | Hg2 | 65.5 |  | Marker72506 | Sg2 | 86.5 |
| Marker73551 | Hg2 | 65.8 |  | Marker73107 | Sg2 | 87.1 |
| Marker73660 | Hg2 | 66.6 |  | Marker72847 | Sg2 | 87.9 |
| Marker74370 | Hg2 | 67.9 |  | Marker73198 | Sg2 | 88.6 |
| Marker74189 | Hg2 | 68.9 |  | Marker89707 | Sg2 | 89.3 |
| Marker74292 | Hg2 | 69.4 |  | Marker111750 | Sg2 | 89.7 |
| Marker74922 | Hg2 | 70.0 |  | Marker73236 | Sg2 | 90.9 |
| Marker74105 | Hg2 | 70.5 |  | Marker73508 | Sg2 | 91.6 |
| Marker73853 | Hg2 | 71.5 |  | Marker73483 | Sg2 | 92.2 |
| Marker74615 | Hg2 | 72.2 |  | Marker73336 | Sg2 | 92.7 |
| Marker74590 | Hg2 | 72.6 |  | Marker72758 | Sg2 | 93.8 |
| Marker75014 | Hg2 | 73.3 |  | Marker73201 | Sg2 | 94.9 |
| Marker74859 | Hg2 | 74.3 |  | Marker73311 | Sg2 | 95.4 |
| Marker74522 | Hg2 | 75.1 |  | Marker73265 | Sg2 | 95.9 |
| Marker74841 | Hg2 | 76.0 |  | Marker73468 | Sg2 | 98.0 |
| Marker74974 | Hg2 | 76.9 |  | Marker73481 | Sg2 | 98.7 |
| Marker74669 | Hg2 | 77.3 |  | Marker74823 | Sg2 | 100.0 |
| Marker75205 | Hg2 | 78.0 |  | Marker74576 | Sg2 | 100.9 |
| Marker76268 | Hg2 | 78.7 |  | Marker74615 | Sg2 | 101.6 |
| Marker75838 | Hg2 | 79.1 |  | Marker74606 | Sg2 | 102.2 |
| Marker75145 | Hg2 | 79.7 |  | Marker74858 | Sg2 | 103.4 |
| Marker75846 | Hg2 | 80.3 |  | Marker74663 | Sg2 | 104.2 |
| Marker75742 | Hg2 | 80.9 |  | Marker74929 | Sg2 | 105.3 |
| Marker75646 | Hg2 | 81.5 |  | Marker74922 | Sg2 | 106.4 |
| Marker75658 | Hg2 | 82.2 |  | Marker75750 | Sg2 | 107.0 |
| Marker76203 | Hg2 | 82.9 |  | Marker75636 | Sg2 | 107.9 |
| Marker75614 | Hg2 | 83.4 |  | Marker75721 | Sg2 | 109.4 |
| Marker76228 | Hg2 | 84.3 |  | Marker75597 | Sg2 | 110.4 |
| Marker75790 | Hg2 | 85.0 |  | Marker75659 | Sg2 | 112.0 |
| Marker75834 | Hg2 | 86.2 |  | Marker76203 | Sg2 | 112.4 |
| Marker75788 | Hg2 | 86.7 |  | Marker76301 | Sg2 | 113.5 |
| Marker75760 | Hg2 | 87.5 |  | Marker76128 | Sg2 | 115.0 |
| Marker76868 | Hg2 | 87.9 |  | Marker75846 | Sg2 | 116.1 |
| Marker76659 | Hg2 | 89.0 |  | Marker75844 | Sg2 | 116.6 |
| Marker76745 | Hg2 | 89.6 |  | Marker76261 | Sg2 | 116.9 |
| Marker75774 | Hg2 | 90.3 |  | Marker76061 | Sg2 | 117.7 |
| Marker76722 | Hg2 | 91.2 |  | Marker75988 | Sg2 | 118.2 |
| Marker76436 | Hg2 | 92.5 |  | Marker75838 | Sg2 | 118.5 |
| Marker76629 | Hg2 | 93.1 |  | Marker75928 | Sg2 | 119.5 |
| Marker76500 | Hg2 | 93.8 |  | Marker75832 | Sg2 | 120.1 |
| Marker76501 | Hg2 | 94.3 |  | Marker75790 | Sg2 | 120.8 |
| Marker76721 | Hg2 | 95.6 |  | Marker75787 | Sg2 | 122.2 |
| Marker76386 | Hg2 | 96.6 |  | Marker75760 | Sg2 | 123.0 |
| Marker76598 | Hg2 | 100.4 |  | Marker76884 | Sg2 | 123.5 |
| Marker63284 | Hg3 | 0.0 |  | Marker76850 | Sg2 | 124.4 |
| Marker63263 | Hg3 | 2.2 |  | Marker76821 | Sg2 | 125.1 |
| Marker61119 | Hg3 | 3.2 |  | Marker76784 | Sg2 | 126.3 |
| Marker62859 | Hg3 | 4.0 |  | Marker76660 | Sg2 | 127.5 |
| Marker61269 | Hg3 | 5.0 |  | Marker76659 | Sg2 | 127.9 |
| Marker61140 | Hg3 | 5.8 |  | Marker76700 | Sg2 | 128.5 |
| Marker63169 | Hg3 | 6.2 |  | Marker76722 | Sg2 | 129.8 |
| Marker62787 | Hg3 | 6.9 |  | Marker76639 | Sg2 | 130.4 |
| Marker62528 | Hg3 | 7.5 |  | Marker76418 | Sg2 | 131.8 |
| Marker63202 | Hg3 | 7.9 |  | Marker76500 | Sg2 | 133.6 |
| Marker61612 | Hg3 | 8.3 |  | Marker76454 | Sg2 | 134.1 |
| Marker62408 | Hg3 | 8.7 |  | Marker63443 | Sg2 | 135.1 |
| Marker61884 | Hg3 | 9.2 |  | Marker76394 | Sg2 | 137.1 |
| Marker61871 | Hg3 | 9.5 |  | Marker76565 | Sg2 | 138.5 |
| Marker61339 | Hg3 | 9.9 |  | Marker76373 | Sg2 | 140.0 |
| Marker61547 | Hg3 | 10.1 |  | Marker63215 | Sg3 | 0.0 |
| Marker62969 | Hg3 | 10.5 |  | Marker63271 | Sg3 | 2.4 |
| Marker63121 | Hg3 | 10.8 |  | Marker61117 | Sg3 | 6.7 |
| Marker61104 | Hg3 | 11.1 |  | Marker61138 | Sg3 | 8.7 |
| Marker61134 | Hg3 | 11.7 |  | Marker62796 | Sg3 | 10.1 |
| Marker62943 | Hg3 | 11.9 |  | Marker62951 | Sg3 | 11.6 |
| Marker61573 | Hg3 | 12.2 |  | Marker63181 | Sg3 | 12.6 |
| Marker61699 | Hg3 | 12.5 |  | Marker63033 | Sg3 | 14.1 |
| Marker61732 | Hg3 | 13.0 |  | Marker61125 | Sg3 | 15.5 |
| Marker61872 | Hg3 | 13.6 |  | Marker63149 | Sg3 | 16.0 |
| Marker61907 | Hg3 | 13.7 |  | Marker61164 | Sg3 | 17.5 |
| Marker60678 | Hg3 | 14.1 |  | Marker63121 | Sg3 | 17.8 |
| Marker61535 | Hg3 | 14.5 |  | Marker61651 | Sg3 | 18.7 |
| Marker61365 | Hg3 | 14.7 |  | Marker61515 | Sg3 | 19.0 |
| Marker62016 | Hg3 | 15.1 |  | Marker63169 | Sg3 | 19.6 |
| Marker61407 | Hg3 | 15.5 |  | Marker61323 | Sg3 | 20.3 |
| Marker62318 | Hg3 | 16.0 |  | Marker62942 | Sg3 | 21.2 |
| Marker61991 | Hg3 | 16.3 |  | Marker63234 | Sg3 | 22.0 |
| Marker62018 | Hg3 | 16.7 |  | Marker61547 | Sg3 | 22.5 |
| Marker62000 | Hg3 | 16.9 |  | Marker61612 | Sg3 | 23.1 |
| Marker62008 | Hg3 | 17.1 |  | Marker61555 | Sg3 | 24.3 |
| Marker62067 | Hg3 | 17.5 |  | Marker61342 | Sg3 | 25.0 |
| Marker62358 | Hg3 | 17.9 |  | Marker61991 | Sg3 | 25.3 |
| Marker62541 | Hg3 | 18.4 |  | Marker61391 | Sg3 | 26.0 |
| Marker62343 | Hg3 | 18.6 |  | Marker61876 | Sg3 | 26.8 |
| Marker62104 | Hg3 | 18.7 |  | Marker61872 | Sg3 | 27.0 |
| Marker62284 | Hg3 | 19.2 |  | Marker61856 | Sg3 | 27.8 |
| Marker62206 | Hg3 | 19.6 |  | Marker62000 | Sg3 | 28.9 |
| Marker62614 | Hg3 | 20.1 |  | Marker61895 | Sg3 | 29.2 |
| Marker60573 | Hg3 | 20.3 |  | Marker62016 | Sg3 | 30.2 |
| Marker62606 | Hg3 | 20.8 |  | Marker61912 | Sg3 | 31.2 |
| Marker60723 | Hg3 | 21.0 |  | Marker62055 | Sg3 | 31.9 |
| Marker14305 | Hg3 | 21.2 |  | Marker62197 | Sg3 | 32.5 |
| Marker60637 | Hg3 | 21.6 |  | Marker61995 | Sg3 | 33.4 |
| Marker62786 | Hg3 | 21.8 |  | Marker62343 | Sg3 | 33.7 |
| Marker60985 | Hg3 | 22.0 |  | Marker61973 | Sg3 | 34.8 |
| Marker72316 | Hg3 | 22.3 |  | Marker61699 | Sg3 | 35.4 |
| Marker122061 | Hg3 | 22.6 |  | Marker62098 | Sg3 | 35.7 |
| Marker60832 | Hg3 | 23.0 |  | Marker62408 | Sg3 | 36.8 |
| Marker60915 | Hg3 | 23.4 |  | Marker62258 | Sg3 | 37.5 |
| Marker60332 | Hg3 | 24.0 |  | Marker62315 | Sg3 | 38.2 |
| Marker60421 | Hg3 | 24.2 |  | Marker62327 | Sg3 | 39.0 |
| Marker60682 | Hg3 | 24.6 |  | Marker62606 | Sg3 | 39.5 |
| Marker60268 | Hg3 | 24.9 |  | Marker62366 | Sg3 | 39.7 |
| Marker60180 | Hg3 | 25.7 |  | Marker62539 | Sg3 | 41.1 |
| Marker60115 | Hg3 | 26.3 |  | Marker62786 | Sg3 | 41.7 |
| Marker59874 | Hg3 | 26.9 |  | Marker62491 | Sg3 | 42.7 |
| Marker60082 | Hg3 | 27.3 |  | Marker62692 | Sg3 | 43.8 |
| Marker59201 | Hg3 | 27.8 |  | Marker60682 | Sg3 | 45.5 |
| Marker59614 | Hg3 | 28.4 |  | Marker62620 | Sg3 | 45.8 |
| Marker59089 | Hg3 | 28.8 |  | Marker60710 | Sg3 | 47.4 |
| Marker59992 | Hg3 | 29.1 |  | Marker60803 | Sg3 | 48.7 |
| Marker59025 | Hg3 | 29.5 |  | Marker60530 | Sg3 | 49.9 |
| Marker59568 | Hg3 | 29.9 |  | Marker60659 | Sg3 | 50.6 |
| Marker59745 | Hg3 | 30.2 |  | Marker60598 | Sg3 | 51.3 |
| Marker59544 | Hg3 | 30.5 |  | Marker60907 | Sg3 | 51.6 |
| Marker59427 | Hg3 | 31.0 |  | Marker59544 | Sg3 | 52.4 |
| Marker59314 | Hg3 | 31.4 |  | Marker57693 | Sg3 | 54.2 |
| Marker58664 | Hg3 | 32.1 |  | Marker60915 | Sg3 | 55.0 |
| Marker57891 | Hg3 | 32.5 |  | Marker59611 | Sg3 | 55.8 |
| Marker58989 | Hg3 | 33.0 |  | Marker58388 | Sg3 | 57.5 |
| Marker58834 | Hg3 | 33.5 |  | Marker57739 | Sg3 | 59.1 |
| Marker58893 | Hg3 | 34.3 |  | Marker116994 | Sg3 | 60.4 |
| Marker58591 | Hg3 | 34.6 |  | Marker72717 | Sg3 | 61.1 |
| Marker57834 | Hg3 | 34.8 |  | Marker56644 | Sg3 | 61.7 |
| Marker58476 | Hg3 | 35.4 |  | Marker113721 | Sg3 | 62.7 |
| Marker58412 | Hg3 | 35.8 |  | Marker57207 | Sg3 | 63.8 |
| Marker57746 | Hg3 | 36.3 |  | Marker115632 | Sg3 | 64.5 |
| Marker58342 | Hg3 | 37.1 |  | Marker57055 | Sg3 | 64.8 |
| Marker116994 | Hg3 | 37.4 |  | Marker57191 | Sg3 | 65.5 |
| Marker120775 | Hg3 | 37.8 |  | Marker57591 | Sg3 | 65.9 |
| Marker57572 | Hg3 | 38.3 |  | Marker56955 | Sg3 | 66.5 |
| Marker112330 | Hg3 | 38.7 |  | Marker57186 | Sg3 | 67.0 |
| Marker113632 | Hg3 | 39.3 |  | Marker55787 | Sg3 | 67.8 |
| Marker57416 | Hg3 | 39.8 |  | Marker56126 | Sg3 | 68.5 |
| Marker57191 | Hg3 | 40.3 |  | Marker55253 | Sg3 | 68.8 |
| Marker57638 | Hg3 | 40.8 |  | Marker55474 | Sg3 | 69.6 |
| Marker57591 | Hg3 | 41.0 |  | Marker56723 | Sg3 | 69.9 |
| Marker57595 | Hg3 | 41.1 |  | Marker54592 | Sg3 | 70.5 |
| Marker57194 | Hg3 | 42.1 |  | Marker56348 | Sg3 | 70.7 |
| Marker54465 | Hg3 | 42.6 |  | Marker28707 | Sg3 | 71.5 |
| Marker57322 | Hg3 | 43.2 |  | Marker56200 | Sg3 | 71.8 |
| Marker57223 | Hg3 | 43.5 |  | Marker55551 | Sg3 | 72.7 |
| Marker57207 | Hg3 | 43.7 |  | Marker54526 | Sg3 | 73.5 |
| Marker57180 | Hg3 | 44.0 |  | Marker54531 | Sg3 | 74.2 |
| Marker56822 | Hg3 | 44.2 |  | Marker113245 | Sg3 | 74.5 |
| Marker56723 | Hg3 | 44.7 |  | Marker54475 | Sg3 | 75.0 |
| Marker55758 | Hg3 | 45.3 |  | Marker54359 | Sg3 | 76.1 |
| Marker57090 | Hg3 | 45.8 |  | Marker54112 | Sg3 | 76.5 |
| Marker55787 | Hg3 | 46.1 |  | Marker54048 | Sg3 | 76.9 |
| Marker55843 | Hg3 | 46.2 |  | Marker54280 | Sg3 | 77.7 |
| Marker56348 | Hg3 | 46.7 |  | Marker54040 | Sg3 | 78.0 |
| Marker57020 | Hg3 | 47.0 |  | Marker46732 | Sg3 | 78.5 |
| Marker55561 | Hg3 | 47.6 |  | Marker53705 | Sg3 | 79.2 |
| Marker55462 | Hg3 | 48.3 |  | Marker53861 | Sg3 | 80.1 |
| Marker54789 | Hg3 | 48.7 |  | Marker110001 | Sg3 | 80.5 |
| Marker55260 | Hg3 | 49.1 |  | Marker53600 | Sg3 | 81.1 |
| Marker55341 | Hg3 | 49.5 |  | Marker53540 | Sg3 | 81.3 |
| Marker56545 | Hg3 | 49.9 |  | Marker53619 | Sg3 | 81.9 |
| Marker113245 | Hg3 | 50.2 |  | Marker53698 | Sg3 | 82.6 |
| Marker56071 | Hg3 | 50.4 |  | Marker53702 | Sg3 | 82.7 |
| Marker113347 | Hg3 | 50.9 |  | Marker4954 | Sg3 | 83.2 |
| Marker42507 | Hg3 | 51.2 |  | Marker53790 | Sg3 | 83.9 |
| Marker113267 | Hg3 | 51.5 |  | Marker53503 | Sg3 | 84.3 |
| Marker56237 | Hg3 | 51.9 |  | Marker53192 | Sg3 | 85.4 |
| Marker55536 | Hg3 | 52.4 |  | Marker53269 | Sg3 | 85.5 |
| Marker55969 | Hg3 | 52.8 |  | Marker109800 | Sg3 | 86.5 |
| Marker54830 | Hg3 | 53.2 |  | Marker53140 | Sg3 | 87.2 |
| Marker54675 | Hg3 | 53.4 |  | Marker53093 | Sg3 | 87.8 |
| Marker56022 | Hg3 | 53.8 |  | Marker53027 | Sg3 | 88.2 |
| Marker54491 | Hg3 | 54.3 |  | Marker52981 | Sg3 | 89.3 |
| Marker54040 | Hg3 | 54.6 |  | Marker52959 | Sg3 | 89.8 |
| Marker55101 | Hg3 | 54.9 |  | Marker52553 | Sg3 | 90.6 |
| Marker113263 | Hg3 | 55.2 |  | Marker52879 | Sg3 | 90.9 |
| Marker53934 | Hg3 | 55.8 |  | Marker52926 | Sg3 | 91.5 |
| Marker54044 | Hg3 | 56.2 |  | Marker52807 | Sg3 | 92.1 |
| Marker54338 | Hg3 | 56.8 |  | Marker52274 | Sg3 | 92.8 |
| Marker54199 | Hg3 | 57.1 |  | Marker52599 | Sg3 | 93.4 |
| Marker53702 | Hg3 | 57.3 |  | Marker52336 | Sg3 | 93.9 |
| Marker53885 | Hg3 | 57.8 |  | Marker52402 | Sg3 | 94.4 |
| Marker53619 | Hg3 | 58.2 |  | Marker52353 | Sg3 | 94.6 |
| Marker53672 | Hg3 | 58.5 |  | Marker52261 | Sg3 | 95.1 |
| Marker53841 | Hg3 | 59.0 |  | Marker113092 | Sg3 | 96.0 |
| Marker52353 | Hg3 | 59.4 |  | Marker52221 | Sg3 | 96.2 |
| Marker53661 | Hg3 | 59.6 |  | Marker52152 | Sg3 | 96.8 |
| Marker53192 | Hg3 | 60.1 |  | Marker52309 | Sg3 | 97.2 |
| Marker53543 | Hg3 | 60.4 |  | Marker52344 | Sg3 | 97.8 |
| Marker110006 | Hg3 | 60.8 |  | Marker52279 | Sg3 | 98.1 |
| Marker109780 | Hg3 | 61.4 |  | Marker52778 | Sg3 | 98.5 |
| Marker109858 | Hg3 | 61.8 |  | Marker52008 | Sg3 | 99.0 |
| Marker53255 | Hg3 | 62.1 |  | Marker52128 | Sg3 | 99.6 |
| Marker52778 | Hg3 | 62.3 |  | Marker52427 | Sg3 | 100.1 |
| Marker53452 | Hg3 | 62.6 |  | Marker51636 | Sg3 | 100.6 |
| Marker53027 | Hg3 | 63.0 |  | Marker52055 | Sg3 | 101.0 |
| Marker53509 | Hg3 | 63.3 |  | Marker114583 | Sg3 | 101.5 |
| Marker52553 | Hg3 | 63.9 |  | Marker52003 | Sg3 | 101.9 |
| Marker53118 | Hg3 | 64.2 |  | Marker51422 | Sg3 | 102.4 |
| Marker52891 | Hg3 | 64.6 |  | Marker51625 | Sg3 | 102.8 |
| Marker52427 | Hg3 | 64.8 |  | Marker51775 | Sg3 | 103.8 |
| Marker52967 | Hg3 | 64.9 |  | Marker51712 | Sg3 | 104.6 |
| Marker52959 | Hg3 | 65.3 |  | Marker51054 | Sg3 | 105.0 |
| Marker53075 | Hg3 | 65.6 |  | Marker51431 | Sg3 | 106.2 |
| Marker52796 | Hg3 | 65.8 |  | Marker51299 | Sg3 | 106.7 |
| Marker52274 | Hg3 | 66.1 |  | Marker51533 | Sg3 | 107.2 |
| Marker52653 | Hg3 | 66.4 |  | Marker50479 | Sg3 | 107.7 |
| Marker52828 | Hg3 | 66.9 |  | Marker51316 | Sg3 | 108.4 |
| Marker52336 | Hg3 | 67.2 |  | Marker51433 | Sg3 | 108.9 |
| Marker52285 | Hg3 | 67.7 |  | Marker50232 | Sg3 | 109.5 |
| Marker52261 | Hg3 | 68.1 |  | Marker51348 | Sg3 | 110.0 |
| Marker52273 | Hg3 | 68.2 |  | Marker51270 | Sg3 | 110.1 |
| Marker113092 | Hg3 | 68.7 |  | Marker51131 | Sg3 | 110.5 |
| Marker52148 | Hg3 | 68.9 |  | Marker51257 | Sg3 | 111.6 |
| Marker52182 | Hg3 | 69.3 |  | Marker51238 | Sg3 | 112.1 |
| Marker52152 | Hg3 | 69.5 |  | Marker51028 | Sg3 | 112.9 |
| Marker51923 | Hg3 | 69.8 |  | Marker51212 | Sg3 | 114.1 |
| Marker52072 | Hg3 | 70.1 |  | Marker116909 | Sg3 | 114.3 |
| Marker51633 | Hg3 | 70.5 |  | Marker50795 | Sg3 | 114.9 |
| Marker52008 | Hg3 | 70.9 |  | Marker50381 | Sg3 | 116.0 |
| Marker51327 | Hg3 | 71.0 |  | Marker50466 | Sg3 | 116.3 |
| Marker51215 | Hg3 | 71.4 |  | Marker51045 | Sg3 | 117.5 |
| Marker51065 | Hg3 | 71.8 |  | Marker50498 | Sg3 | 118.8 |
| Marker51170 | Hg3 | 72.2 |  | Marker50039 | Sg3 | 119.5 |
| Marker51054 | Hg3 | 72.5 |  | Marker50702 | Sg3 | 120.2 |
| Marker50249 | Hg3 | 72.8 |  | Marker50176 | Sg3 | 121.5 |
| Marker51536 | Hg3 | 73.3 |  | Marker49806 | Sg3 | 122.2 |
| Marker51422 | Hg3 | 73.4 |  | Marker50571 | Sg3 | 123.1 |
| Marker51785 | Hg3 | 73.8 |  | Marker106231 | Sg3 | 124.1 |
| Marker51257 | Hg3 | 73.8 |  | Marker49947 | Sg3 | 125.0 |
| Marker51286 | Hg3 | 74.0 |  | Marker50111 | Sg3 | 126.4 |
| Marker51636 | Hg3 | 74.1 |  | Marker49555 | Sg3 | 127.6 |
| Marker51036 | Hg3 | 74.2 |  | Marker50036 | Sg3 | 128.9 |
| Marker50812 | Hg3 | 74.7 |  | Marker45541 | Sg3 | 130.7 |
| Marker51348 | Hg3 | 75.0 |  | Marker49778 | Sg3 | 131.9 |
| Marker51720 | Hg3 | 75.2 |  | Marker49707 | Sg3 | 134.5 |
| Marker51521 | Hg3 | 75.7 |  | Marker49549 | Sg3 | 138.1 |
| Marker50378 | Hg3 | 76.1 |  | Marker44694 | Sg4 | 0.0 |
| Marker50479 | Hg3 | 76.3 |  | Marker44402 | Sg4 | 2.0 |
| Marker51212 | Hg3 | 76.9 |  | Marker43910 | Sg4 | 4.4 |
| Marker50586 | Hg3 | 77.2 |  | Marker52116 | Sg4 | 6.1 |
| Marker50202 | Hg3 | 77.4 |  | Marker43886 | Sg4 | 8.0 |
| Marker51448 | Hg3 | 78.0 |  | Marker43812 | Sg4 | 9.1 |
| Marker52077 | Hg3 | 78.4 |  | Marker44571 | Sg4 | 9.9 |
| Marker50232 | Hg3 | 78.7 |  | Marker44036 | Sg4 | 10.6 |
| Marker51637 | Hg3 | 79.0 |  | Marker44180 | Sg4 | 11.8 |
| Marker51433 | Hg3 | 79.3 |  | Marker43883 | Sg4 | 12.4 |
| Marker51360 | Hg3 | 79.7 |  | Marker44289 | Sg4 | 13.4 |
| Marker51299 | Hg3 | 79.9 |  | Marker112668 | Sg4 | 14.3 |
| Marker50125 | Hg3 | 80.6 |  | Marker112525 | Sg4 | 15.4 |
| Marker49806 | Hg3 | 81.3 |  | Marker44220 | Sg4 | 15.6 |
| Marker49726 | Hg3 | 81.8 |  | Marker112442 | Sg4 | 17.1 |
| Marker49785 | Hg3 | 82.8 |  | Marker43509 | Sg4 | 18.3 |
| Marker50039 | Hg3 | 83.4 |  | Marker43184 | Sg4 | 18.9 |
| Marker50021 | Hg3 | 84.2 |  | Marker43545 | Sg4 | 19.8 |
| Marker49633 | Hg3 | 85.3 |  | Marker43236 | Sg4 | 20.5 |
| Marker49555 | Hg3 | 86.3 |  | Marker43428 | Sg4 | 20.9 |
| Marker52014 | Hg3 | 87.7 |  | Marker43129 | Sg4 | 22.2 |
| Marker46522 | Hg4 | 0.0 |  | Marker43101 | Sg4 | 23.8 |
| Marker93800 | Hg4 | 0.5 |  | Marker42927 | Sg4 | 24.3 |
| Marker48222 | Hg4 | 1.1 |  | Marker43169 | Sg4 | 25.0 |
| Marker49235 | Hg4 | 2.5 |  | Marker42898 | Sg4 | 25.8 |
| Marker49419 | Hg4 | 3.6 |  | Marker42957 | Sg4 | 26.6 |
| Marker48082 | Hg4 | 4.0 |  | Marker43043 | Sg4 | 27.5 |
| Marker29774 | Hg4 | 4.6 |  | Marker42925 | Sg4 | 27.7 |
| Marker49039 | Hg4 | 5.2 |  | Marker41040 | Sg4 | 28.9 |
| Marker47656 | Hg4 | 5.9 |  | Marker42741 | Sg4 | 29.7 |
| Marker49174 | Hg4 | 6.4 |  | Marker42836 | Sg4 | 30.5 |
| Marker45080 | Hg4 | 7.0 |  | Marker42299 | Sg4 | 31.4 |
| Marker45624 | Hg4 | 7.5 |  | Marker42775 | Sg4 | 32.1 |
| Marker49324 | Hg4 | 8.4 |  | Marker42337 | Sg4 | 32.9 |
| Marker116764 | Hg4 | 9.0 |  | Marker41859 | Sg4 | 33.7 |
| Marker49361 | Hg4 | 9.3 |  | Marker41643 | Sg4 | 34.4 |
| Marker48037 | Hg4 | 10.0 |  | Marker41074 | Sg4 | 35.9 |
| Marker47459 | Hg4 | 10.5 |  | Marker40981 | Sg4 | 37.1 |
| Marker60206 | Hg4 | 11.1 |  | Marker41285 | Sg4 | 38.9 |
| Marker47978 | Hg4 | 11.4 |  | Marker41355 | Sg4 | 39.8 |
| Marker48131 | Hg4 | 11.5 |  | Marker41558 | Sg4 | 40.3 |
| Marker4488 | Hg4 | 12.1 |  | Marker41340 | Sg4 | 40.8 |
| Marker46937 | Hg4 | 12.9 |  | Marker41351 | Sg4 | 41.4 |
| Marker45697 | Hg4 | 13.3 |  | Marker119270 | Sg4 | 42.2 |
| Marker45797 | Hg4 | 13.7 |  | Marker40475 | Sg4 | 43.5 |
| Marker44933 | Hg4 | 13.9 |  | Marker40397 | Sg4 | 44.2 |
| Marker46832 | Hg4 | 14.4 |  | Marker40455 | Sg4 | 45.0 |
| Marker48545 | Hg4 | 15.0 |  | Marker40697 | Sg4 | 45.5 |
| Marker46457 | Hg4 | 15.5 |  | Marker40472 | Sg4 | 46.2 |
| Marker46250 | Hg4 | 15.8 |  | Marker40712 | Sg4 | 47.0 |
| Marker48594 | Hg4 | 16.1 |  | Marker40276 | Sg4 | 47.9 |
| Marker48323 | Hg4 | 16.5 |  | Marker39996 | Sg4 | 49.0 |
| Marker46387 | Hg4 | 16.8 |  | Marker39938 | Sg4 | 49.4 |
| Marker45293 | Hg4 | 17.2 |  | Marker40274 | Sg4 | 50.0 |
| Marker46856 | Hg4 | 17.5 |  | Marker40074 | Sg4 | 50.6 |
| Marker24860 | Hg4 | 17.9 |  | Marker40371 | Sg4 | 51.4 |
| Marker44610 | Hg4 | 18.4 |  | Marker40171 | Sg4 | 51.8 |
| Marker48703 | Hg4 | 18.7 |  | Marker39689 | Sg4 | 52.7 |
| Marker47795 | Hg4 | 18.8 |  | Marker39444 | Sg4 | 53.6 |
| Marker47892 | Hg4 | 19.4 |  | Marker40426 | Sg4 | 54.0 |
| Marker44692 | Hg4 | 19.9 |  | Marker39268 | Sg4 | 54.8 |
| Marker44779 | Hg4 | 20.1 |  | Marker39791 | Sg4 | 55.6 |
| Marker45024 | Hg4 | 20.6 |  | Marker39572 | Sg4 | 56.0 |
| Marker47186 | Hg4 | 21.3 |  | Marker39343 | Sg4 | 56.9 |
| Marker47399 | Hg4 | 21.7 |  | Marker40108 | Sg4 | 57.8 |
| Marker119672 | Hg4 | 22.1 |  | Marker39211 | Sg4 | 58.5 |
| Marker44560 | Hg4 | 22.7 |  | Marker39549 | Sg4 | 59.4 |
| Marker45472 | Hg4 | 23.0 |  | Marker40181 | Sg4 | 60.1 |
| Marker112525 | Hg4 | 23.6 |  | Marker39153 | Sg4 | 60.7 |
| Marker112712 | Hg4 | 23.8 |  | Marker39173 | Sg4 | 61.3 |
| Marker44241 | Hg4 | 24.4 |  | Marker39080 | Sg4 | 61.9 |
| Marker43883 | Hg4 | 24.7 |  | Marker38929 | Sg4 | 62.9 |
| Marker44139 | Hg4 | 25.2 |  | Marker39076 | Sg4 | 63.5 |
| Marker44206 | Hg4 | 25.4 |  | Marker39116 | Sg4 | 64.4 |
| Marker44037 | Hg4 | 25.7 |  | Marker39002 | Sg4 | 65.6 |
| Marker43812 | Hg4 | 26.2 |  | Marker38610 | Sg4 | 66.4 |
| Marker43722 | Hg4 | 26.7 |  | Marker38726 | Sg4 | 68.1 |
| Marker43817 | Hg4 | 27.6 |  | Marker38847 | Sg4 | 68.6 |
| Marker112595 | Hg4 | 27.9 |  | Marker38856 | Sg4 | 69.0 |
| Marker43531 | Hg4 | 28.8 |  | Marker38706 | Sg4 | 70.1 |
| Marker43590 | Hg4 | 29.2 |  | Marker37945 | Sg4 | 72.3 |
| Marker43509 | Hg4 | 29.3 |  | Marker38005 | Sg4 | 73.7 |
| Marker112476 | Hg4 | 29.7 |  | Marker37832 | Sg4 | 74.8 |
| Marker43420 | Hg4 | 30.4 |  | Marker37792 | Sg4 | 76.0 |
| Marker43436 | Hg4 | 31.1 |  | Marker37884 | Sg4 | 76.8 |
| Marker43344 | Hg4 | 31.9 |  | Marker37320 | Sg4 | 77.6 |
| Marker43314 | Hg4 | 32.2 |  | Marker37721 | Sg4 | 78.5 |
| Marker42927 | Hg4 | 32.7 |  | Marker37630 | Sg4 | 79.3 |
| Marker42979 | Hg4 | 33.1 |  | Marker37628 | Sg4 | 80.1 |
| Marker43126 | Hg4 | 33.6 |  | Marker37284 | Sg4 | 80.9 |
| Marker43169 | Hg4 | 33.8 |  | Marker37600 | Sg4 | 81.3 |
| Marker42901 | Hg4 | 34.1 |  | Marker37259 | Sg4 | 81.9 |
| Marker42864 | Hg4 | 34.7 |  | Marker37636 | Sg4 | 82.5 |
| Marker43043 | Hg4 | 35.1 |  | Marker37389 | Sg4 | 82.8 |
| Marker42782 | Hg4 | 35.6 |  | Marker37562 | Sg4 | 83.9 |
| Marker42753 | Hg4 | 36.1 |  | Marker37429 | Sg4 | 84.8 |
| Marker118377 | Hg4 | 36.4 |  | Marker37421 | Sg4 | 84.9 |
| Marker121810 | Hg4 | 37.1 |  | Marker47553 | Sg4 | 85.8 |
| Marker42836 | Hg4 | 37.4 |  | Marker37315 | Sg4 | 86.1 |
| Marker43186 | Hg4 | 37.9 |  | Marker37330 | Sg4 | 87.1 |
| Marker43073 | Hg4 | 38.4 |  | Marker47598 | Sg4 | 87.7 |
| Marker42337 | Hg4 | 39.1 |  | Marker37390 | Sg4 | 88.6 |
| Marker42196 | Hg4 | 39.3 |  | Marker37044 | Sg4 | 89.5 |
| Marker42385 | Hg4 | 39.9 |  | Marker37365 | Sg4 | 90.3 |
| Marker42775 | Hg4 | 40.4 |  | Marker47614 | Sg4 | 91.0 |
| Marker42119 | Hg4 | 40.9 |  | Marker37155 | Sg4 | 91.9 |
| Marker42596 | Hg4 | 41.8 |  | Marker37017 | Sg4 | 92.9 |
| Marker42494 | Hg4 | 42.5 |  | Marker47572 | Sg4 | 94.1 |
| Marker42583 | Hg4 | 43.0 |  | Marker47533 | Sg4 | 95.9 |
| Marker42262 | Hg4 | 43.5 |  | Marker37063 | Sg4 | 96.9 |
| Marker42419 | Hg4 | 44.2 |  | Marker37195 | Sg4 | 98.2 |
| Marker42717 | Hg4 | 45.0 |  | Marker37078 | Sg4 | 99.9 |
| Marker42049 | Hg4 | 45.7 |  | Marker37107 | Sg4 | 101.5 |
| Marker41923 | Hg4 | 46.7 |  | Marker73796 | Sg5 | 0.0 |
| Marker41839 | Hg4 | 47.4 |  | Marker89556 | Sg5 | 2.0 |
| Marker41558 | Hg4 | 47.9 |  | Marker90342 | Sg5 | 3.5 |
| Marker41679 | Hg4 | 48.5 |  | Marker89612 | Sg5 | 6.2 |
| Marker40952 | Hg4 | 49.4 |  | Marker117179 | Sg5 | 7.8 |
| Marker40915 | Hg4 | 49.9 |  | Marker89567 | Sg5 | 8.9 |
| Marker41040 | Hg4 | 50.3 |  | Marker90026 | Sg5 | 9.2 |
| Marker41545 | Hg4 | 50.7 |  | Marker90155 | Sg5 | 10.6 |
| Marker41076 | Hg4 | 51.1 |  | Marker89848 | Sg5 | 11.4 |
| Marker41021 | Hg4 | 52.1 |  | Marker60840 | Sg5 | 12.6 |
| Marker40827 | Hg4 | 52.5 |  | Marker90414 | Sg5 | 13.1 |
| Marker41172 | Hg4 | 53.2 |  | Marker90494 | Sg5 | 14.0 |
| Marker41281 | Hg4 | 53.6 |  | Marker117235 | Sg5 | 14.6 |
| Marker40697 | Hg4 | 54.2 |  | Marker90997 | Sg5 | 16.0 |
| Marker41344 | Hg4 | 54.8 |  | Marker91411 | Sg5 | 18.1 |
| Marker41353 | Hg4 | 55.4 |  | Marker91167 | Sg5 | 18.3 |
| Marker40712 | Hg4 | 56.0 |  | Marker90876 | Sg5 | 18.6 |
| Marker41358 | Hg4 | 56.3 |  | Marker91059 | Sg5 | 19.0 |
| Marker40713 | Hg4 | 57.2 |  | Marker90900 | Sg5 | 19.7 |
| Marker40798 | Hg4 | 57.7 |  | Marker90581 | Sg5 | 20.5 |
| Marker40703 | Hg4 | 58.1 |  | Marker91313 | Sg5 | 21.7 |
| Marker40666 | Hg4 | 59.0 |  | Marker91253 | Sg5 | 22.3 |
| Marker40426 | Hg4 | 59.5 |  | Marker91394 | Sg5 | 23.5 |
| Marker40603 | Hg4 | 59.8 |  | Marker91463 | Sg5 | 24.9 |
| Marker40381 | Hg4 | 60.4 |  | Marker91850 | Sg5 | 26.6 |
| Marker40475 | Hg4 | 60.6 |  | Marker91603 | Sg5 | 27.3 |
| Marker40555 | Hg4 | 61.1 |  | Marker91602 | Sg5 | 27.8 |
| Marker40181 | Hg4 | 61.5 |  | Marker91862 | Sg5 | 28.5 |
| Marker40198 | Hg4 | 61.8 |  | Marker91911 | Sg5 | 29.6 |
| Marker40438 | Hg4 | 62.3 |  | Marker92073 | Sg5 | 30.4 |
| Marker40466 | Hg4 | 62.8 |  | Marker92034 | Sg5 | 31.3 |
| Marker40274 | Hg4 | 63.0 |  | Marker92165 | Sg5 | 31.8 |
| Marker40371 | Hg4 | 63.4 |  | Marker58276 | Sg5 | 33.6 |
| Marker40313 | Hg4 | 63.6 |  | Marker115189 | Sg5 | 34.7 |
| Marker40152 | Hg4 | 64.2 |  | Marker58252 | Sg5 | 35.9 |
| Marker39959 | Hg4 | 64.7 |  | Marker92275 | Sg5 | 36.1 |
| Marker40087 | Hg4 | 65.2 |  | Marker92465 | Sg5 | 37.0 |
| Marker39997 | Hg4 | 65.4 |  | Marker92341 | Sg5 | 37.9 |
| Marker40108 | Hg4 | 65.6 |  | Marker57966 | Sg5 | 38.3 |
| Marker121768 | Hg4 | 66.0 |  | Marker92443 | Sg5 | 39.7 |
| Marker39888 | Hg4 | 66.6 |  | Marker93013 | Sg5 | 41.1 |
| Marker39494 | Hg4 | 66.8 |  | Marker93342 | Sg5 | 42.3 |
| Marker39658 | Hg4 | 67.4 |  | Marker93623 | Sg5 | 43.7 |
| Marker39572 | Hg4 | 67.7 |  | Marker92813 | Sg5 | 44.1 |
| Marker39275 | Hg4 | 67.9 |  | Marker93038 | Sg5 | 44.8 |
| Marker39751 | Hg4 | 68.4 |  | Marker93086 | Sg5 | 45.6 |
| Marker38633 | Hg4 | 68.9 |  | Marker92834 | Sg5 | 46.1 |
| Marker38977 | Hg4 | 69.2 |  | Marker93410 | Sg5 | 46.3 |
| Marker39204 | Hg4 | 69.4 |  | Marker92725 | Sg5 | 47.1 |
| Marker39153 | Hg4 | 69.6 |  | Marker93316 | Sg5 | 48.1 |
| Marker39099 | Hg4 | 69.7 |  | Marker93209 | Sg5 | 49.2 |
| Marker39007 | Hg4 | 70.1 |  | Marker93437 | Sg5 | 50.3 |
| Marker39160 | Hg4 | 70.6 |  | Marker93551 | Sg5 | 51.3 |
| Marker39247 | Hg4 | 71.1 |  | Marker93449 | Sg5 | 52.5 |
| Marker39077 | Hg4 | 71.6 |  | Marker93520 | Sg5 | 53.5 |
| Marker38035 | Hg4 | 72.0 |  | Marker94132 | Sg5 | 54.8 |
| Marker38726 | Hg4 | 72.2 |  | Marker94681 | Sg5 | 56.7 |
| Marker38535 | Hg4 | 72.6 |  | Marker94821 | Sg5 | 58.8 |
| Marker38474 | Hg4 | 73.2 |  | Marker94409 | Sg5 | 60.0 |
| Marker37284 | Hg4 | 73.3 |  | Marker94592 | Sg5 | 61.9 |
| Marker38757 | Hg4 | 73.7 |  | Marker93668 | Sg5 | 62.9 |
| Marker38141 | Hg4 | 74.0 |  | Marker94768 | Sg5 | 63.8 |
| Marker37945 | Hg4 | 74.3 |  | Marker94794 | Sg5 | 64.6 |
| Marker38109 | Hg4 | 74.5 |  | Marker94960 | Sg5 | 65.7 |
| Marker37630 | Hg4 | 75.2 |  | Marker95019 | Sg5 | 67.1 |
| Marker38162 | Hg4 | 75.4 |  | Marker94849 | Sg5 | 69.2 |
| Marker37320 | Hg4 | 76.0 |  | Marker95123 | Sg5 | 73.8 |
| Marker38259 | Hg4 | 76.4 |  | Marker95514 | Sg5 | 76.0 |
| Marker37909 | Hg4 | 76.7 |  | Marker95392 | Sg5 | 77.2 |
| Marker37855 | Hg4 | 77.1 |  | Marker96724 | Sg5 | 80.3 |
| Marker37163 | Hg4 | 77.7 |  | Marker95572 | Sg5 | 83.7 |
| Marker37421 | Hg4 | 78.0 |  | Marker96722 | Sg5 | 85.2 |
| Marker37279 | Hg4 | 78.2 |  | Marker96590 | Sg5 | 88.8 |
| Marker37389 | Hg4 | 78.8 |  | Marker96680 | Sg5 | 91.0 |
| Marker37363 | Hg4 | 79.0 |  | Marker96639 | Sg5 | 92.7 |
| Marker37138 | Hg4 | 79.3 |  | Marker96474 | Sg5 | 96.9 |
| Marker37382 | Hg4 | 80.0 |  | Marker96394 | Sg5 | 105.5 |
| Marker37529 | Hg4 | 80.6 |  | Marker98335 | Sg5 | 118.2 |
| Marker37313 | Hg4 | 81.4 |  | Marker98292 | Sg5 | 128.0 |
| Marker47598 | Hg4 | 82.4 |  | Marker78 | Sg6 | 0.0 |
| Marker47582 | Hg4 | 82.6 |  | Marker293 | Sg6 | 2.7 |
| Marker37229 | Hg4 | 83.1 |  | Marker267 | Sg6 | 4.2 |
| Marker37259 | Hg4 | 83.6 |  | Marker109397 | Sg6 | 7.7 |
| Marker38365 | Hg4 | 84.3 |  | Marker1516 | Sg6 | 8.2 |
| Marker37267 | Hg4 | 85.2 |  | Marker109495 | Sg6 | 9.4 |
| Marker47553 | Hg4 | 85.7 |  | Marker1408 | Sg6 | 9.9 |
| Marker47537 | Hg4 | 86.4 |  | Marker109561 | Sg6 | 10.6 |
| Marker37110 | Hg4 | 87.7 |  | Marker368 | Sg6 | 12.1 |
| Marker37044 | Hg4 | 88.3 |  | Marker1056 | Sg6 | 13.0 |
| Marker37078 | Hg4 | 95.2 |  | Marker1417 | Sg6 | 14.0 |
| Marker89718 | Hg5 | 0.0 |  | Marker828 | Sg6 | 14.7 |
| Marker117178 | Hg5 | 2.9 |  | Marker993 | Sg6 | 15.4 |
| Marker89629 | Hg5 | 4.2 |  | Marker710 | Sg6 | 16.1 |
| Marker89567 | Hg5 | 5.4 |  | Marker686 | Sg6 | 17.6 |
| Marker73798 | Hg5 | 5.8 |  | Marker885 | Sg6 | 18.2 |
| Marker89551 | Hg5 | 6.7 |  | Marker39 | Sg6 | 19.3 |
| Marker49228 | Hg5 | 7.5 |  | Marker484 | Sg6 | 20.4 |
| Marker89985 | Hg5 | 9.0 |  | Marker503 | Sg6 | 20.9 |
| Marker89822 | Hg5 | 10.8 |  | Marker518 | Sg6 | 21.5 |
| Marker89990 | Hg5 | 11.5 |  | Marker533 | Sg6 | 22.3 |
| Marker22829 | Hg5 | 12.7 |  | Marker109403 | Sg6 | 22.7 |
| Marker89915 | Hg5 | 14.1 |  | Marker282 | Sg6 | 23.2 |
| Marker91330 | Hg5 | 15.0 |  | Marker1345 | Sg6 | 23.6 |
| Marker90785 | Hg5 | 15.8 |  | Marker555 | Sg6 | 25.3 |
| Marker90876 | Hg5 | 16.2 |  | Marker782 | Sg6 | 25.6 |
| Marker91372 | Hg5 | 17.4 |  | Marker1935 | Sg6 | 26.5 |
| Marker91270 | Hg5 | 18.3 |  | Marker617 | Sg6 | 27.6 |
| Marker90877 | Hg5 | 19.2 |  | Marker538 | Sg6 | 27.6 |
| Marker91394 | Hg5 | 19.9 |  | Marker522 | Sg6 | 28.3 |
| Marker91562 | Hg5 | 20.3 |  | Marker2000 | Sg6 | 29.3 |
| Marker91609 | Hg5 | 21.6 |  | Marker613 | Sg6 | 30.3 |
| Marker91909 | Hg5 | 22.5 |  | Marker2395 | Sg6 | 31.2 |
| Marker91602 | Hg5 | 23.0 |  | Marker1627 | Sg6 | 31.9 |
| Marker92071 | Hg5 | 24.4 |  | Marker1805 | Sg6 | 32.5 |
| Marker58252 | Hg5 | 25.0 |  | Marker1705 | Sg6 | 33.4 |
| Marker92117 | Hg5 | 25.5 |  | Marker2106 | Sg6 | 34.0 |
| Marker58058 | Hg5 | 26.4 |  | Marker1650 | Sg6 | 34.5 |
| Marker58102 | Hg5 | 27.1 |  | Marker1823 | Sg6 | 35.1 |
| Marker57911 | Hg5 | 27.8 |  | Marker1821 | Sg6 | 35.3 |
| Marker58201 | Hg5 | 28.6 |  | Marker1717 | Sg6 | 36.1 |
| Marker92945 | Hg5 | 29.4 |  | Marker1613 | Sg6 | 36.5 |
| Marker113111 | Hg5 | 29.9 |  | Marker3187 | Sg6 | 37.0 |
| Marker92305 | Hg5 | 30.6 |  | Marker3056 | Sg6 | 38.0 |
| Marker92344 | Hg5 | 31.1 |  | Marker2433 | Sg6 | 39.6 |
| Marker92609 | Hg5 | 32.1 |  | Marker2995 | Sg6 | 40.4 |
| Marker92797 | Hg5 | 32.6 |  | Marker2933 | Sg6 | 41.1 |
| Marker92341 | Hg5 | 33.0 |  | Marker2834 | Sg6 | 42.0 |
| Marker92814 | Hg5 | 33.2 |  | Marker2727 | Sg6 | 42.3 |
| Marker92488 | Hg5 | 33.8 |  | Marker3521 | Sg6 | 43.0 |
| Marker92458 | Hg5 | 34.4 |  | Marker2526 | Sg6 | 44.6 |
| Marker93066 | Hg5 | 35.5 |  | Marker3409 | Sg6 | 45.8 |
| Marker93123 | Hg5 | 36.3 |  | Marker3410 | Sg6 | 46.6 |
| Marker93410 | Hg5 | 36.8 |  | Marker3363 | Sg6 | 47.2 |
| Marker93339 | Hg5 | 37.2 |  | Marker3215 | Sg6 | 48.2 |
| Marker93344 | Hg5 | 37.8 |  | Marker3661 | Sg6 | 49.1 |
| Marker93274 | Hg5 | 38.5 |  | Marker3555 | Sg6 | 49.9 |
| Marker93497 | Hg5 | 39.4 |  | Marker3695 | Sg6 | 50.8 |
| Marker93443 | Hg5 | 40.5 |  | Marker3599 | Sg6 | 52.4 |
| Marker93415 | Hg5 | 41.4 |  | Marker117899 | Sg6 | 52.8 |
| Marker93539 | Hg5 | 42.7 |  | Marker3462 | Sg6 | 53.2 |
| Marker93587 | Hg5 | 43.4 |  | Marker3683 | Sg6 | 54.9 |
| Marker93700 | Hg5 | 44.0 |  | Marker4267 | Sg6 | 55.3 |
| Marker93762 | Hg5 | 45.2 |  | Marker3785 | Sg6 | 55.7 |
| Marker94219 | Hg5 | 46.3 |  | Marker3699 | Sg6 | 56.3 |
| Marker93663 | Hg5 | 47.0 |  | Marker4208 | Sg6 | 58.3 |
| Marker93668 | Hg5 | 47.7 |  | Marker3824 | Sg6 | 59.5 |
| Marker94640 | Hg5 | 48.2 |  | Marker4092 | Sg6 | 60.6 |
| Marker94950 | Hg5 | 49.4 |  | Marker4171 | Sg6 | 62.5 |
| Marker94800 | Hg5 | 50.2 |  | Marker117893 | Sg6 | 63.7 |
| Marker94972 | Hg5 | 51.1 |  | Marker3999 | Sg6 | 64.8 |
| Marker94843 | Hg5 | 52.3 |  | Marker4299 | Sg6 | 67.5 |
| Marker94738 | Hg5 | 52.9 |  | Marker6104 | Sg6 | 70.2 |
| Marker94375 | Hg5 | 54.2 |  | Marker5683 | Sg6 | 73.6 |
| Marker96629 | Hg5 | 58.0 |  | Marker6022 | Sg6 | 75.9 |
| Marker96722 | Hg5 | 58.7 |  | Marker7505 | Sg6 | 80.1 |
| Marker96437 | Hg5 | 59.9 |  | Marker7835 | Sg6 | 83.6 |
| Marker96324 | Hg5 | 60.8 |  | Marker8175 | Sg6 | 89.9 |
| Marker96639 | Hg5 | 61.7 |  | Marker9652 | Sg6 | 98.5 |
| Marker96241 | Hg5 | 61.9 |  | Marker99125 | Sg7 | 0.0 |
| Marker96201 | Hg5 | 62.8 |  | Marker98707 | Sg7 | 1.6 |
| Marker96125 | Hg5 | 63.5 |  | Marker111148 | Sg7 | 6.6 |
| Marker95988 | Hg5 | 64.3 |  | Marker98587 | Sg7 | 8.5 |
| Marker96613 | Hg5 | 66.3 |  | Marker99166 | Sg7 | 9.0 |
| Marker97182 | Hg5 | 67.1 |  | Marker98720 | Sg7 | 10.4 |
| Marker97133 | Hg5 | 68.1 |  | Marker99007 | Sg7 | 11.5 |
| Marker96362 | Hg5 | 69.3 |  | Marker98653 | Sg7 | 12.3 |
| Marker96070 | Hg5 | 70.6 |  | Marker98765 | Sg7 | 13.0 |
| Marker97376 | Hg5 | 71.7 |  | Marker99388 | Sg7 | 14.1 |
| Marker97539 | Hg5 | 73.2 |  | Marker49215 | Sg7 | 15.5 |
| Marker110846 | Hg5 | 73.6 |  | Marker111574 | Sg7 | 16.1 |
| Marker97692 | Hg5 | 75.6 |  | Marker111540 | Sg7 | 16.7 |
| Marker97632 | Hg5 | 76.6 |  | Marker111548 | Sg7 | 17.5 |
| Marker96917 | Hg5 | 78.0 |  | Marker111553 | Sg7 | 17.8 |
| Marker97012 | Hg5 | 78.8 |  | Marker111581 | Sg7 | 18.5 |
| Marker39848 | Hg5 | 79.6 |  | Marker111719 | Sg7 | 19.4 |
| Marker97774 | Hg5 | 80.9 |  | Marker100031 | Sg7 | 20.5 |
| Marker97285 | Hg5 | 82.1 |  | Marker99492 | Sg7 | 21.7 |
| Marker97808 | Hg5 | 83.1 |  | Marker99990 | Sg7 | 22.1 |
| Marker97906 | Hg5 | 83.9 |  | Marker99604 | Sg7 | 22.9 |
| Marker97834 | Hg5 | 84.9 |  | Marker110988 | Sg7 | 23.9 |
| Marker97830 | Hg5 | 86.0 |  | Marker99587 | Sg7 | 24.6 |
| Marker98239 | Hg5 | 87.8 |  | Marker99315 | Sg7 | 25.4 |
| Marker98081 | Hg5 | 88.7 |  | Marker114669 | Sg7 | 25.8 |
| Marker98188 | Hg5 | 89.9 |  | Marker3180 | Sg7 | 26.3 |
| Marker97985 | Hg5 | 91.1 |  | Marker99776 | Sg7 | 27.3 |
| Marker98450 | Hg5 | 93.3 |  | Marker99577 | Sg7 | 28.1 |
| Marker98304 | Hg5 | 94.4 |  | Marker100163 | Sg7 | 28.8 |
| Marker98014 | Hg5 | 96.1 |  | Marker114640 | Sg7 | 30.0 |
| Marker98376 | Hg5 | 104.1 |  | Marker111028 | Sg7 | 30.8 |
| Marker1344 | Hg6 | 0.0 |  | Marker100309 | Sg7 | 31.7 |
| Marker79 | Hg6 | 1.0 |  | Marker99792 | Sg7 | 32.0 |
| Marker62 | Hg6 | 2.1 |  | Marker100458 | Sg7 | 33.0 |
| Marker624 | Hg6 | 3.0 |  | Marker108066 | Sg7 | 33.7 |
| Marker1408 | Hg6 | 3.8 |  | Marker100820 | Sg7 | 34.3 |
| Marker109395 | Hg6 | 4.0 |  | Marker100658 | Sg7 | 35.6 |
| Marker1394 | Hg6 | 4.6 |  | Marker100936 | Sg7 | 36.4 |
| Marker336 | Hg6 | 5.3 |  | Marker101675 | Sg7 | 36.9 |
| Marker39 | Hg6 | 5.5 |  | Marker108121 | Sg7 | 38.0 |
| Marker1258 | Hg6 | 5.9 |  | Marker101770 | Sg7 | 39.1 |
| Marker1154 | Hg6 | 6.4 |  | Marker31638 | Sg7 | 40.0 |
| Marker707 | Hg6 | 7.1 |  | Marker120140 | Sg7 | 40.6 |
| Marker1531 | Hg6 | 7.9 |  | Marker115019 | Sg7 | 41.5 |
| Marker1508 | Hg6 | 7.9 |  | Marker101033 | Sg7 | 41.9 |
| Marker1037 | Hg6 | 8.7 |  | Marker117035 | Sg7 | 43.2 |
| Marker109449 | Hg6 | 9.3 |  | Marker101167 | Sg7 | 43.8 |
| Marker1410 | Hg6 | 9.9 |  | Marker101231 | Sg7 | 45.4 |
| Marker378 | Hg6 | 10.4 |  | Marker112276 | Sg7 | 46.1 |
| Marker109403 | Hg6 | 10.6 |  | Marker101419 | Sg7 | 46.8 |
| Marker282 | Hg6 | 11.0 |  | Marker112263 | Sg7 | 48.1 |
| Marker902 | Hg6 | 11.5 |  | Marker112174 | Sg7 | 48.5 |
| Marker682 | Hg6 | 12.1 |  | Marker112137 | Sg7 | 49.6 |
| Marker540 | Hg6 | 12.6 |  | Marker111975 | Sg7 | 50.1 |
| Marker538 | Hg6 | 12.8 |  | Marker100547 | Sg7 | 50.7 |
| Marker529 | Hg6 | 13.4 |  | Marker25776 | Sg7 | 51.5 |
| Marker487 | Hg6 | 13.8 |  | Marker25725 | Sg7 | 52.7 |
| Marker2035 | Hg6 | 14.1 |  | Marker25760 | Sg7 | 53.1 |
| Marker522 | Hg6 | 14.5 |  | Marker102186 | Sg7 | 54.6 |
| Marker507 | Hg6 | 15.2 |  | Marker102209 | Sg7 | 55.6 |
| Marker1019 | Hg6 | 15.8 |  | Marker102119 | Sg7 | 55.8 |
| Marker782 | Hg6 | 16.2 |  | Marker101963 | Sg7 | 57.5 |
| Marker464 | Hg6 | 16.4 |  | Marker102028 | Sg7 | 57.7 |
| Marker809 | Hg6 | 16.9 |  | Marker101896 | Sg7 | 58.4 |
| Marker613 | Hg6 | 17.2 |  | Marker101954 | Sg7 | 59.1 |
| Marker849 | Hg6 | 17.4 |  | Marker102773 | Sg7 | 59.6 |
| Marker1985 | Hg6 | 17.9 |  | Marker101837 | Sg7 | 60.6 |
| Marker1618 | Hg6 | 18.5 |  | Marker108836 | Sg7 | 61.3 |
| Marker1737 | Hg6 | 18.9 |  | Marker102596 | Sg7 | 61.7 |
| Marker1916 | Hg6 | 19.3 |  | Marker108894 | Sg7 | 62.1 |
| Marker1821 | Hg6 | 19.5 |  | Marker108908 | Sg7 | 63.0 |
| Marker1845 | Hg6 | 19.8 |  | Marker102736 | Sg7 | 63.5 |
| Marker1650 | Hg6 | 20.4 |  | Marker102775 | Sg7 | 64.1 |
| Marker2265 | Hg6 | 20.6 |  | Marker103737 | Sg7 | 64.6 |
| Marker2325 | Hg6 | 21.3 |  | Marker103319 | Sg7 | 64.9 |
| Marker1613 | Hg6 | 21.5 |  | Marker109229 | Sg7 | 65.7 |
| Marker2178 | Hg6 | 21.9 |  | Marker102797 | Sg7 | 66.4 |
| Marker2396 | Hg6 | 22.5 |  | Marker109084 | Sg7 | 66.9 |
| Marker1682 | Hg6 | 23.1 |  | Marker109078 | Sg7 | 67.5 |
| Marker2448 | Hg6 | 23.8 |  | Marker109139 | Sg7 | 68.3 |
| Marker3088 | Hg6 | 24.6 |  | Marker109252 | Sg7 | 69.6 |
| Marker2684 | Hg6 | 24.9 |  | Marker103498 | Sg7 | 70.2 |
| Marker2727 | Hg6 | 25.5 |  | Marker103406 | Sg7 | 70.7 |
| Marker2947 | Hg6 | 25.6 |  | Marker103491 | Sg7 | 71.4 |
| Marker3053 | Hg6 | 26.1 |  | Marker103555 | Sg7 | 71.9 |
| Marker2708 | Hg6 | 26.8 |  | Marker103573 | Sg7 | 72.0 |
| Marker2776 | Hg6 | 27.4 |  | Marker103814 | Sg7 | 73.0 |
| Marker2896 | Hg6 | 27.9 |  | Marker104027 | Sg7 | 73.4 |
| Marker3410 | Hg6 | 28.1 |  | Marker103951 | Sg7 | 74.2 |
| Marker2649 | Hg6 | 28.4 |  | Marker104056 | Sg7 | 75.0 |
| Marker3392 | Hg6 | 28.9 |  | Marker104436 | Sg7 | 75.8 |
| Marker3464 | Hg6 | 29.4 |  | Marker103991 | Sg7 | 76.7 |
| Marker117881 | Hg6 | 30.0 |  | Marker104347 | Sg7 | 77.2 |
| Marker4300 | Hg6 | 30.1 |  | Marker104482 | Sg7 | 77.9 |
| Marker3661 | Hg6 | 30.6 |  | Marker104199 | Sg7 | 78.9 |
| Marker3924 | Hg6 | 30.9 |  | Marker104363 | Sg7 | 79.5 |
| Marker2470 | Hg6 | 31.2 |  | Marker104716 | Sg7 | 80.7 |
| Marker117899 | Hg6 | 31.4 |  | Marker105195 | Sg7 | 82.6 |
| Marker3497 | Hg6 | 31.7 |  | Marker105074 | Sg7 | 83.4 |
| Marker3293 | Hg6 | 32.6 |  | Marker105427 | Sg7 | 84.0 |
| Marker3695 | Hg6 | 33.0 |  | Marker104970 | Sg7 | 84.6 |
| Marker4342 | Hg6 | 33.5 |  | Marker104774 | Sg7 | 85.4 |
| Marker4228 | Hg6 | 34.0 |  | Marker105120 | Sg7 | 86.2 |
| Marker4267 | Hg6 | 34.6 |  | Marker105288 | Sg7 | 87.4 |
| Marker4149 | Hg6 | 34.8 |  | Marker105458 | Sg7 | 88.3 |
| Marker4016 | Hg6 | 35.3 |  | Marker105854 | Sg7 | 90.5 |
| Marker3588 | Hg6 | 35.7 |  | Marker105516 | Sg7 | 91.3 |
| Marker4420 | Hg6 | 36.2 |  | Marker105757 | Sg7 | 92.7 |
| Marker5216 | Hg6 | 37.0 |  | Marker105707 | Sg7 | 93.5 |
| Marker4208 | Hg6 | 37.2 |  | Marker105420 | Sg7 | 94.6 |
| Marker4609 | Hg6 | 37.6 |  | Marker105883 | Sg7 | 96.4 |
| Marker4490 | Hg6 | 38.3 |  | Marker105812 | Sg7 | 99.1 |
| Marker4876 | Hg6 | 39.2 |  | Marker105497 | Sg7 | 101.2 |
| Marker4934 | Hg6 | 39.9 |  | Marker106322 | Sg7 | 107.3 |
| Marker4762 | Hg6 | 40.6 |  | Marker106072 | Sg7 | 113.0 |
| Marker5526 | Hg6 | 41.2 |  | Marker77323 | Sg8 | 0.0 |
| Marker4974 | Hg6 | 41.5 |  | Marker77577 | Sg8 | 3.1 |
| Marker5421 | Hg6 | 42.1 |  | Marker77281 | Sg8 | 6.1 |
| Marker5644 | Hg6 | 43.1 |  | Marker77237 | Sg8 | 8.3 |
| Marker5722 | Hg6 | 44.1 |  | Marker77503 | Sg8 | 10.0 |
| Marker5877 | Hg6 | 45.1 |  | Marker77199 | Sg8 | 11.6 |
| Marker6254 | Hg6 | 46.3 |  | Marker76993 | Sg8 | 12.4 |
| Marker6616 | Hg6 | 46.9 |  | Marker77831 | Sg8 | 14.0 |
| Marker6374 | Hg6 | 47.6 |  | Marker77253 | Sg8 | 14.7 |
| Marker6103 | Hg6 | 48.7 |  | Marker78232 | Sg8 | 15.7 |
| Marker6106 | Hg6 | 49.2 |  | Marker77859 | Sg8 | 17.0 |
| Marker6889 | Hg6 | 50.3 |  | Marker79518 | Sg8 | 19.3 |
| Marker6358 | Hg6 | 50.8 |  | Marker78139 | Sg8 | 20.1 |
| Marker112844 | Hg6 | 51.7 |  | Marker78030 | Sg8 | 20.9 |
| Marker7529 | Hg6 | 52.3 |  | Marker77896 | Sg8 | 21.3 |
| Marker112735 | Hg6 | 52.6 |  | Marker77085 | Sg8 | 22.2 |
| Marker121666 | Hg6 | 53.6 |  | Marker78300 | Sg8 | 22.7 |
| Marker7697 | Hg6 | 54.9 |  | Marker77966 | Sg8 | 24.2 |
| Marker8576 | Hg6 | 55.5 |  | Marker78081 | Sg8 | 25.1 |
| Marker44209 | Hg6 | 56.7 |  | Marker79510 | Sg8 | 25.5 |
| Marker9030 | Hg6 | 57.4 |  | Marker78064 | Sg8 | 26.4 |
| Marker8729 | Hg6 | 58.6 |  | Marker79441 | Sg8 | 27.5 |
| Marker9548 | Hg6 | 59.2 |  | Marker79227 | Sg8 | 28.5 |
| Marker9814 | Hg6 | 60.0 |  | Marker78116 | Sg8 | 29.0 |
| Marker9652 | Hg6 | 60.8 |  | Marker79496 | Sg8 | 30.0 |
| Marker9519 | Hg6 | 61.4 |  | Marker79283 | Sg8 | 30.7 |
| Marker9243 | Hg6 | 62.5 |  | Marker79133 | Sg8 | 31.5 |
| Marker9984 | Hg6 | 63.0 |  | Marker79378 | Sg8 | 32.0 |
| Marker9674 | Hg6 | 64.1 |  | Marker78823 | Sg8 | 33.3 |
| Marker110661 | Hg6 | 66.2 |  | Marker78971 | Sg8 | 34.1 |
| Marker10537 | Hg6 | 67.6 |  | Marker65526 | Sg8 | 35.2 |
| Marker10025 | Hg6 | 68.3 |  | Marker79062 | Sg8 | 35.8 |
| Marker110516 | Hg6 | 69.5 |  | Marker79233 | Sg8 | 36.2 |
| Marker11769 | Hg6 | 70.5 |  | Marker78492 | Sg8 | 37.0 |
| Marker11719 | Hg6 | 71.7 |  | Marker78553 | Sg8 | 38.4 |
| Marker11537 | Hg6 | 72.9 |  | Marker78761 | Sg8 | 39.4 |
| Marker10963 | Hg6 | 73.6 |  | Marker78346 | Sg8 | 41.0 |
| Marker11109 | Hg6 | 74.9 |  | Marker78381 | Sg8 | 41.9 |
| Marker11860 | Hg6 | 76.0 |  | Marker114432 | Sg8 | 42.8 |
| Marker11296 | Hg6 | 76.9 |  | Marker78587 | Sg8 | 43.2 |
| Marker11421 | Hg6 | 78.2 |  | Marker79992 | Sg8 | 44.8 |
| Marker12195 | Hg6 | 80.1 |  | Marker79901 | Sg8 | 45.7 |
| Marker13312 | Hg6 | 81.7 |  | Marker79845 | Sg8 | 46.9 |
| Marker12986 | Hg6 | 83.7 |  | Marker80315 | Sg8 | 47.3 |
| Marker13676 | Hg6 | 85.8 |  | Marker79776 | Sg8 | 47.7 |
| Marker14193 | Hg6 | 86.8 |  | Marker79684 | Sg8 | 48.6 |
| Marker14271 | Hg6 | 87.8 |  | Marker79637 | Sg8 | 49.1 |
| Marker12710 | Hg6 | 88.9 |  | Marker80142 | Sg8 | 50.0 |
| Marker13416 | Hg6 | 89.6 |  | Marker80260 | Sg8 | 50.8 |
| Marker12853 | Hg6 | 90.9 |  | Marker80450 | Sg8 | 51.7 |
| Marker13868 | Hg6 | 92.2 |  | Marker79652 | Sg8 | 52.1 |
| Marker14326 | Hg6 | 93.1 |  | Marker80160 | Sg8 | 52.3 |
| Marker13785 | Hg6 | 94.0 |  | Marker80362 | Sg8 | 52.7 |
| Marker13162 | Hg6 | 94.6 |  | Marker81045 | Sg8 | 53.7 |
| Marker14708 | Hg6 | 96.8 |  | Marker80965 | Sg8 | 54.7 |
| Marker14651 | Hg6 | 97.9 |  | Marker80159 | Sg8 | 55.5 |
| Marker14382 | Hg6 | 98.8 |  | Marker80231 | Sg8 | 55.7 |
| Marker14057 | Hg6 | 100.6 |  | Marker80168 | Sg8 | 56.2 |
| Marker14107 | Hg6 | 101.9 |  | Marker81244 | Sg8 | 57.4 |
| Marker14452 | Hg6 | 102.5 |  | Marker80789 | Sg8 | 58.2 |
| Marker13598 | Hg6 | 103.6 |  | Marker81631 | Sg8 | 58.6 |
| Marker111166 | Hg7 | 0.0 |  | Marker81120 | Sg8 | 59.2 |
| Marker99584 | Hg7 | 1.9 |  | Marker81743 | Sg8 | 59.6 |
| Marker110898 | Hg7 | 3.1 |  | Marker81823 | Sg8 | 60.6 |
| Marker99608 | Hg7 | 3.8 |  | Marker80915 | Sg8 | 61.1 |
| Marker111574 | Hg7 | 4.3 |  | Marker81322 | Sg8 | 61.6 |
| Marker46782 | Hg7 | 4.7 |  | Marker81611 | Sg8 | 62.8 |
| Marker111551 | Hg7 | 5.6 |  | Marker82238 | Sg8 | 64.1 |
| Marker111548 | Hg7 | 5.8 |  | Marker82200 | Sg8 | 64.4 |
| Marker111413 | Hg7 | 6.1 |  | Marker81945 | Sg8 | 66.1 |
| Marker99025 | Hg7 | 6.8 |  | Marker82015 | Sg8 | 66.7 |
| Marker99141 | Hg7 | 7.3 |  | Marker82284 | Sg8 | 66.9 |
| Marker99577 | Hg7 | 7.7 |  | Marker81594 | Sg8 | 67.3 |
| Marker111635 | Hg7 | 8.0 |  | Marker82278 | Sg8 | 67.8 |
| Marker98653 | Hg7 | 8.2 |  | Marker82441 | Sg8 | 68.5 |
| Marker99236 | Hg7 | 8.7 |  | Marker82672 | Sg8 | 69.3 |
| Marker99527 | Hg7 | 9.4 |  | Marker83430 | Sg8 | 69.9 |
| Marker111028 | Hg7 | 9.6 |  | Marker82590 | Sg8 | 70.6 |
| Marker98738 | Hg7 | 10.1 |  | Marker82058 | Sg8 | 71.1 |
| Marker98720 | Hg7 | 10.3 |  | Marker82942 | Sg8 | 71.5 |
| Marker99312 | Hg7 | 10.7 |  | Marker83517 | Sg8 | 72.5 |
| Marker119289 | Hg7 | 11.5 |  | Marker82731 | Sg8 | 73.0 |
| Marker99792 | Hg7 | 11.8 |  | Marker83553 | Sg8 | 74.1 |
| Marker100162 | Hg7 | 12.0 |  | Marker83691 | Sg8 | 74.8 |
| Marker115233 | Hg7 | 12.7 |  | Marker83506 | Sg8 | 75.8 |
| Marker100605 | Hg7 | 13.2 |  | Marker83778 | Sg8 | 76.1 |
| Marker100029 | Hg7 | 13.5 |  | Marker83602 | Sg8 | 76.5 |
| Marker114669 | Hg7 | 14.1 |  | Marker83321 | Sg8 | 77.3 |
| Marker100320 | Hg7 | 14.5 |  | Marker82895 | Sg8 | 77.8 |
| Marker101347 | Hg7 | 15.2 |  | Marker83847 | Sg8 | 78.2 |
| Marker99713 | Hg7 | 15.4 |  | Marker84242 | Sg8 | 79.2 |
| Marker100098 | Hg7 | 15.8 |  | Marker84285 | Sg8 | 79.6 |
| Marker99990 | Hg7 | 16.1 |  | Marker85874 | Sg8 | 80.0 |
| Marker110971 | Hg7 | 16.5 |  | Marker85933 | Sg8 | 80.3 |
| Marker102154 | Hg7 | 16.8 |  | Marker84028 | Sg8 | 80.7 |
| Marker112263 | Hg7 | 17.6 |  | Marker83109 | Sg8 | 81.1 |
| Marker100674 | Hg7 | 17.7 |  | Marker86560 | Sg8 | 81.8 |
| Marker101351 | Hg7 | 18.1 |  | Marker86755 | Sg8 | 82.2 |
| Marker118402 | Hg7 | 18.8 |  | Marker55620 | Sg8 | 82.7 |
| Marker100547 | Hg7 | 19.1 |  | Marker85037 | Sg8 | 83.1 |
| Marker102205 | Hg7 | 19.5 |  | Marker83340 | Sg8 | 83.5 |
| Marker101418 | Hg7 | 20.4 |  | Marker86252 | Sg8 | 83.8 |
| Marker25760 | Hg7 | 20.9 |  | Marker86212 | Sg8 | 84.2 |
| Marker111962 | Hg7 | 21.5 |  | Marker84563 | Sg8 | 84.7 |
| Marker102060 | Hg7 | 21.9 |  | Marker84919 | Sg8 | 85.1 |
| Marker101540 | Hg7 | 22.3 |  | Marker84477 | Sg8 | 85.6 |
| Marker102021 | Hg7 | 23.6 |  | Marker84472 | Sg8 | 85.8 |
| Marker102529 | Hg7 | 24.7 |  | Marker83249 | Sg8 | 86.5 |
| Marker102028 | Hg7 | 25.4 |  | Marker84782 | Sg8 | 87.1 |
| Marker101867 | Hg7 | 26.0 |  | Marker85651 | Sg8 | 87.3 |
| Marker101954 | Hg7 | 26.5 |  | Marker85413 | Sg8 | 87.8 |
| Marker102341 | Hg7 | 27.1 |  | Marker83949 | Sg8 | 88.3 |
| Marker102419 | Hg7 | 27.8 |  | Marker83189 | Sg8 | 88.6 |
| Marker102596 | Hg7 | 28.3 |  | Marker84365 | Sg8 | 89.0 |
| Marker103162 | Hg7 | 28.7 |  | Marker86562 | Sg8 | 90.0 |
| Marker103020 | Hg7 | 29.8 |  | Marker84876 | Sg8 | 90.2 |
| Marker102905 | Hg7 | 30.4 |  | Marker86295 | Sg8 | 90.9 |
| Marker109053 | Hg7 | 31.2 |  | Marker86976 | Sg8 | 91.5 |
| Marker102775 | Hg7 | 31.5 |  | Marker86700 | Sg8 | 91.8 |
| Marker102678 | Hg7 | 32.0 |  | Marker119175 | Sg8 | 92.7 |
| Marker108872 | Hg7 | 32.4 |  | Marker87121 | Sg8 | 93.1 |
| Marker108894 | Hg7 | 32.9 |  | Marker87599 | Sg8 | 93.3 |
| Marker109250 | Hg7 | 33.4 |  | Marker87062 | Sg8 | 94.1 |
| Marker103361 | Hg7 | 33.7 |  | Marker86808 | Sg8 | 94.6 |
| Marker109084 | Hg7 | 34.5 |  | Marker84378 | Sg8 | 95.5 |
| Marker103514 | Hg7 | 35.5 |  | Marker86508 | Sg8 | 97.0 |
| Marker103713 | Hg7 | 36.2 |  | Marker87584 | Sg8 | 97.6 |
| Marker103859 | Hg7 | 37.0 |  | Marker109597 | Sg8 | 97.9 |
| Marker103498 | Hg7 | 37.5 |  | Marker87449 | Sg8 | 99.0 |
| Marker103946 | Hg7 | 37.6 |  | Marker88070 | Sg8 | 100.0 |
| Marker103591 | Hg7 | 38.1 |  | Marker88126 | Sg8 | 100.9 |
| Marker103766 | Hg7 | 38.9 |  | Marker89495 | Sg8 | 102.2 |
| Marker103555 | Hg7 | 39.3 |  | Marker109573 | Sg8 | 102.6 |
| Marker103981 | Hg7 | 40.4 |  | Marker88429 | Sg8 | 103.6 |
| Marker104027 | Hg7 | 41.1 |  | Marker88525 | Sg8 | 104.1 |
| Marker104076 | Hg7 | 41.4 |  | Marker109625 | Sg8 | 105.0 |
| Marker104096 | Hg7 | 42.4 |  | Marker87616 | Sg8 | 105.8 |
| Marker104885 | Hg7 | 43.5 |  | Marker88331 | Sg8 | 106.8 |
| Marker104733 | Hg7 | 44.6 |  | Marker88734 | Sg8 | 107.9 |
| Marker104436 | Hg7 | 44.6 |  | Marker88730 | Sg8 | 109.3 |
| Marker104665 | Hg7 | 45.6 |  | Marker115509 | Sg8 | 110.4 |
| Marker104347 | Hg7 | 46.2 |  | Marker89080 | Sg8 | 111.2 |
| Marker104227 | Hg7 | 46.8 |  | Marker113837 | Sg8 | 111.7 |
| Marker104774 | Hg7 | 48.5 |  | Marker88798 | Sg8 | 113.1 |
| Marker105088 | Hg7 | 49.7 |  | Marker89212 | Sg8 | 114.8 |
| Marker105258 | Hg7 | 50.9 |  | Marker88996 | Sg8 | 116.8 |
| Marker105427 | Hg7 | 52.2 |  | Marker89353 | Sg8 | 117.6 |
| Marker105307 | Hg7 | 53.1 |  | Marker88942 | Sg8 | 119.0 |
| Marker105471 | Hg7 | 55.3 |  | Marker88929 | Sg8 | 120.8 |
| Marker105519 | Hg7 | 56.2 |  | Marker89246 | Sg8 | 127.6 |
| Marker105832 | Hg7 | 56.8 |  |  |  |  |
| Marker105857 | Hg7 | 57.6 |  |  |  |  |
| Marker105498 | Hg7 | 58.5 |  |  |  |  |
| Marker105707 | Hg7 | 59.5 |  |  |  |  |
| Marker105632 | Hg7 | 60.0 |  |  |  |  |
| Marker105907 | Hg7 | 61.1 |  |  |  |  |
| Marker106007 | Hg7 | 62.1 |  |  |  |  |
| Marker106570 | Hg7 | 65.4 |  |  |  |  |
| Marker106683 | Hg7 | 66.6 |  |  |  |  |
| Marker106630 | Hg7 | 67.8 |  |  |  |  |
| Marker107074 | Hg7 | 68.7 |  |  |  |  |
| Marker107001 | Hg7 | 69.5 |  |  |  |  |
| Marker106409 | Hg7 | 70.6 |  |  |  |  |
| Marker107111 | Hg7 | 72.3 |  |  |  |  |
| Marker107040 | Hg7 | 74.2 |  |  |  |  |
| Marker107226 | Hg7 | 75.6 |  |  |  |  |
| Marker107541 | Hg7 | 76.6 |  |  |  |  |
| Marker107476 | Hg7 | 77.8 |  |  |  |  |
| Marker107591 | Hg7 | 79.2 |  |  |  |  |
| Marker107273 | Hg7 | 80.4 |  |  |  |  |
| Marker107342 | Hg7 | 82.4 |  |  |  |  |
| Marker107738 | Hg7 | 83.3 |  |  |  |  |
| Marker107662 | Hg7 | 84.4 |  |  |  |  |
| Marker107890 | Hg7 | 86.0 |  |  |  |  |
| Marker119972 | Hg7 | 87.8 |  |  |  |  |
| Marker107985 | Hg7 | 88.9 |  |  |  |  |
| Marker108013 | Hg7 | 91.2 |  |  |  |  |
| Marker107388 | Hg7 | 93.2 |  |  |  |  |
| Marker77308 | Hg8 | 0.0 |  |  |  |  |
| Marker77253 | Hg8 | 1.2 |  |  |  |  |
| Marker77900 | Hg8 | 2.5 |  |  |  |  |
| Marker78310 | Hg8 | 3.6 |  |  |  |  |
| Marker77211 | Hg8 | 5.0 |  |  |  |  |
| Marker78175 | Hg8 | 6.1 |  |  |  |  |
| Marker77858 | Hg8 | 7.5 |  |  |  |  |
| Marker78064 | Hg8 | 8.4 |  |  |  |  |
| Marker78255 | Hg8 | 9.6 |  |  |  |  |
| Marker77861 | Hg8 | 10.0 |  |  |  |  |
| Marker79510 | Hg8 | 10.5 |  |  |  |  |
| Marker76920 | Hg8 | 11.2 |  |  |  |  |
| Marker77276 | Hg8 | 11.5 |  |  |  |  |
| Marker77450 | Hg8 | 11.9 |  |  |  |  |
| Marker77138 | Hg8 | 12.7 |  |  |  |  |
| Marker78116 | Hg8 | 13.0 |  |  |  |  |
| Marker77350 | Hg8 | 13.6 |  |  |  |  |
| Marker79476 | Hg8 | 15.1 |  |  |  |  |
| Marker79537 | Hg8 | 15.8 |  |  |  |  |
| Marker77085 | Hg8 | 16.5 |  |  |  |  |
| Marker79371 | Hg8 | 16.9 |  |  |  |  |
| Marker79398 | Hg8 | 17.9 |  |  |  |  |
| Marker79233 | Hg8 | 18.8 |  |  |  |  |
| Marker78612 | Hg8 | 20.8 |  |  |  |  |
| Marker78932 | Hg8 | 21.5 |  |  |  |  |
| Marker78797 | Hg8 | 22.5 |  |  |  |  |
| Marker78586 | Hg8 | 23.4 |  |  |  |  |
| Marker78417 | Hg8 | 24.5 |  |  |  |  |
| Marker78520 | Hg8 | 25.1 |  |  |  |  |
| Marker78349 | Hg8 | 26.1 |  |  |  |  |
| Marker81030 | Hg8 | 26.7 |  |  |  |  |
| Marker114505 | Hg8 | 27.9 |  |  |  |  |
| Marker80096 | Hg8 | 28.4 |  |  |  |  |
| Marker80042 | Hg8 | 29.2 |  |  |  |  |
| Marker80313 | Hg8 | 29.8 |  |  |  |  |
| Marker80142 | Hg8 | 30.2 |  |  |  |  |
| Marker80137 | Hg8 | 30.7 |  |  |  |  |
| Marker80487 | Hg8 | 31.1 |  |  |  |  |
| Marker80111 | Hg8 | 31.7 |  |  |  |  |
| Marker80674 | Hg8 | 32.3 |  |  |  |  |
| Marker80315 | Hg8 | 32.5 |  |  |  |  |
| Marker80718 | Hg8 | 33.0 |  |  |  |  |
| Marker79681 | Hg8 | 33.5 |  |  |  |  |
| Marker114440 | Hg8 | 33.9 |  |  |  |  |
| Marker80160 | Hg8 | 34.8 |  |  |  |  |
| Marker80157 | Hg8 | 35.1 |  |  |  |  |
| Marker80920 | Hg8 | 35.8 |  |  |  |  |
| Marker81322 | Hg8 | 36.3 |  |  |  |  |
| Marker81130 | Hg8 | 36.6 |  |  |  |  |
| Marker81383 | Hg8 | 37.0 |  |  |  |  |
| Marker80231 | Hg8 | 37.7 |  |  |  |  |
| Marker81800 | Hg8 | 38.2 |  |  |  |  |
| Marker81478 | Hg8 | 39.0 |  |  |  |  |
| Marker81689 | Hg8 | 39.4 |  |  |  |  |
| Marker81631 | Hg8 | 40.3 |  |  |  |  |
| Marker81597 | Hg8 | 40.6 |  |  |  |  |
| Marker82193 | Hg8 | 41.5 |  |  |  |  |
| Marker82236 | Hg8 | 42.1 |  |  |  |  |
| Marker81594 | Hg8 | 42.7 |  |  |  |  |
| Marker81553 | Hg8 | 43.0 |  |  |  |  |
| Marker82341 | Hg8 | 43.6 |  |  |  |  |
| Marker82373 | Hg8 | 44.3 |  |  |  |  |
| Marker82019 | Hg8 | 44.8 |  |  |  |  |
| Marker82058 | Hg8 | 45.1 |  |  |  |  |
| Marker82707 | Hg8 | 45.6 |  |  |  |  |
| Marker82405 | Hg8 | 46.4 |  |  |  |  |
| Marker82731 | Hg8 | 46.8 |  |  |  |  |
| Marker82476 | Hg8 | 47.4 |  |  |  |  |
| Marker115636 | Hg8 | 47.6 |  |  |  |  |
| Marker82436 | Hg8 | 48.2 |  |  |  |  |
| Marker82953 | Hg8 | 49.0 |  |  |  |  |
| Marker82895 | Hg8 | 49.7 |  |  |  |  |
| Marker83004 | Hg8 | 50.3 |  |  |  |  |
| Marker117993 | Hg8 | 50.7 |  |  |  |  |
| Marker83082 | Hg8 | 51.6 |  |  |  |  |
| Marker83506 | Hg8 | 52.1 |  |  |  |  |
| Marker83948 | Hg8 | 52.6 |  |  |  |  |
| Marker83438 | Hg8 | 53.3 |  |  |  |  |
| Marker83615 | Hg8 | 53.8 |  |  |  |  |
| Marker84247 | Hg8 | 54.5 |  |  |  |  |
| Marker83340 | Hg8 | 55.2 |  |  |  |  |
| Marker84370 | Hg8 | 55.3 |  |  |  |  |
| Marker83748 | Hg8 | 56.1 |  |  |  |  |
| Marker84336 | Hg8 | 57.0 |  |  |  |  |
| Marker84476 | Hg8 | 58.4 |  |  |  |  |
| Marker85933 | Hg8 | 59.5 |  |  |  |  |
| Marker85954 | Hg8 | 60.4 |  |  |  |  |
| Marker84830 | Hg8 | 61.2 |  |  |  |  |
| Marker84782 | Hg8 | 61.5 |  |  |  |  |
| Marker86840 | Hg8 | 62.4 |  |  |  |  |
| Marker87483 | Hg8 | 63.3 |  |  |  |  |
| Marker86510 | Hg8 | 63.6 |  |  |  |  |
| Marker86303 | Hg8 | 64.1 |  |  |  |  |
| Marker80823 | Hg8 | 64.8 |  |  |  |  |
| Marker86562 | Hg8 | 65.1 |  |  |  |  |
| Marker86247 | Hg8 | 65.4 |  |  |  |  |
| Marker8388 | Hg8 | 65.8 |  |  |  |  |
| Marker86508 | Hg8 | 66.0 |  |  |  |  |
| Marker87156 | Hg8 | 66.2 |  |  |  |  |
| Marker87087 | Hg8 | 66.9 |  |  |  |  |
| Marker87599 | Hg8 | 67.0 |  |  |  |  |
| Marker70248 | Hg8 | 67.2 |  |  |  |  |
| Marker86967 | Hg8 | 67.6 |  |  |  |  |
| Marker86484 | Hg8 | 68.1 |  |  |  |  |
| Marker118691 | Hg8 | 68.2 |  |  |  |  |
| Marker86753 | Hg8 | 68.7 |  |  |  |  |
| Marker85824 | Hg8 | 69.1 |  |  |  |  |
| Marker109747 | Hg8 | 69.6 |  |  |  |  |
| Marker88195 | Hg8 | 70.0 |  |  |  |  |
| Marker73521 | Hg8 | 70.6 |  |  |  |  |
| Marker88126 | Hg8 | 71.0 |  |  |  |  |
| Marker89007 | Hg8 | 71.4 |  |  |  |  |
| Marker70708 | Hg8 | 71.9 |  |  |  |  |
| Marker89080 | Hg8 | 72.2 |  |  |  |  |
| Marker89250 | Hg8 | 72.3 |  |  |  |  |
| Marker73843 | Hg8 | 73.1 |  |  |  |  |
| Marker21908 | Hg8 | 73.5 |  |  |  |  |
| Marker88942 | Hg8 | 73.8 |  |  |  |  |
| Marker87860 | Hg8 | 74.1 |  |  |  |  |
| Marker89314 | Hg8 | 74.6 |  |  |  |  |
| Marker88525 | Hg8 | 74.9 |  |  |  |  |
| Marker88520 | Hg8 | 75.3 |  |  |  |  |
| Marker88996 | Hg8 | 75.7 |  |  |  |  |
| Marker88715 | Hg8 | 76.0 |  |  |  |  |
| Marker88733 | Hg8 | 76.4 |  |  |  |  |
| Marker109577 | Hg8 | 76.8 |  |  |  |  |
| Marker109597 | Hg8 | 77.3 |  |  |  |  |
| Marker89178 | Hg8 | 77.6 |  |  |  |  |
| Marker88264 | Hg8 | 78.2 |  |  |  |  |
| Marker87963 | Hg8 | 78.7 |  |  |  |  |
| Marker86289 | Hg8 | 79.7 |  |  |  |  |
| Marker109610 | Hg8 | 80.6 |  |  |  |  |
| Marker88813 | Hg8 | 81.7 |  |  |  |  |
| Marker88978 | Hg8 | 84.3 |  |  |  |  |
