# Supplementary material for: Construction of a High-Density Genetic Map and Identification of Quantitative Trait Loci Linked to Fruit Quality Traits in Apricots Using Specific-Locus Amplified Fragment Sequencing
Source: Front Plant Sci. 2022 Feb 14;13:798700. doi: 10.3389/fpls.2022.798700 (PMC8882730; doi:10.3389/fpls.2022.798700)
Supplement: Supplementary file 5 [file Table_5.DOCX]

**Supplementary Table 5. Details physical location of mapped markers in apricot reference genome**

| SNP Markers | Chromosome Number | Position | Start | End |
| --- | --- | --- | --- | --- |
| Marker368 | LG1 | 18362 | 18312 | 18412 |
| Marker358 | LG1 | 53484 | 53434 | 53534 |
| Marker352 | LG1 | 74034 | 73984 | 74084 |
| Marker336 | LG1 | 100789 | 100739 | 100839 |
| Marker325 | LG1 | 138972 | 138922 | 139022 |
| Marker315 | LG1 | 152858 | 152808 | 152908 |
| Marker305 | LG1 | 167253 | 167203 | 167303 |
| Marker293 | LG1 | 169810 | 169760 | 169860 |
| Marker79 | LG1 | 209751 | 209701 | 209801 |
| Marker78 | LG1 | 211226 | 211176 | 211276 |
| Marker65 | LG1 | 230237 | 230187 | 230287 |
| Marker64 | LG1 | 235926 | 235876 | 235976 |
| Marker62 | LG1 | 244201 | 244151 | 244251 |
| Marker55 | LG1 | 262758 | 262708 | 262808 |
| Marker43 | LG1 | 296645 | 296595 | 296695 |
| Marker39 | LG1 | 305131 | 305081 | 305181 |
| Marker38 | LG1 | 306724 | 306674 | 306774 |
| Marker32 | LG1 | 324329 | 324279 | 324379 |
| Marker30 | LG1 | 325594 | 325544 | 325644 |
| Marker14 | LG1 | 340912 | 340862 | 340962 |
| Marker373 | LG1 | 473955 | 473905 | 474005 |
| Marker378 | LG1 | 480648 | 480598 | 480698 |
| Marker267 | LG1 | 489514 | 489464 | 489564 |
| Marker282 | LG1 | 509084 | 509034 | 509134 |
| Marker109568 | LG1 | 554797 | 554747 | 554847 |
| Marker109561 | LG1 | 564734 | 564684 | 564784 |
| Marker109540 | LG1 | 614240 | 614190 | 614290 |
| Marker109495 | LG1 | 727699 | 727649 | 727749 |
| Marker109484 | LG1 | 738494 | 738444 | 738544 |
| Marker109478 | LG1 | 750088 | 750038 | 750138 |
| Marker109458 | LG1 | 760856 | 760806 | 760906 |
| Marker109449 | LG1 | 772558 | 772508 | 772608 |
| Marker109438 | LG1 | 785897 | 785847 | 785947 |
| Marker109403 | LG1 | 824474 | 824424 | 824524 |
| Marker109401 | LG1 | 850662 | 850612 | 850712 |
| Marker109397 | LG1 | 862243 | 862193 | 862293 |
| Marker109395 | LG1 | 867831 | 867781 | 867881 |
| Marker109369 | LG1 | 949709 | 949659 | 949759 |
| Marker109366 | LG1 | 951243 | 951193 | 951293 |
| Marker1394 | LG1 | 1036654 | 1036604 | 1036704 |
| Marker1408 | LG1 | 1064104 | 1064054 | 1064154 |
| Marker1410 | LG1 | 1071212 | 1071162 | 1071262 |
| Marker1417 | LG1 | 1075730 | 1075680 | 1075780 |
| Marker1484 | LG1 | 1178596 | 1178546 | 1178646 |
| Marker1531 | LG1 | 1257468 | 1257418 | 1257518 |
| Marker1516 | LG1 | 1277919 | 1277869 | 1277969 |
| Marker1512 | LG1 | 1290401 | 1290351 | 1290451 |
| Marker1508 | LG1 | 1295789 | 1295739 | 1295839 |
| Marker1497 | LG1 | 1314714 | 1314664 | 1314764 |
| Marker103591 | LG1 | 1320455 | 1320405 | 1320505 |
| Marker1037 | LG1 | 1333465 | 1333415 | 1333515 |
| Marker1039 | LG1 | 1337422 | 1337372 | 1337472 |
| Marker1051 | LG1 | 1365986 | 1365936 | 1366036 |
| Marker1053 | LG1 | 1366257 | 1366207 | 1366307 |
| Marker1054 | LG1 | 1367552 | 1367502 | 1367602 |
| Marker1056 | LG1 | 1379867 | 1379817 | 1379917 |
| Marker1058 | LG1 | 1381933 | 1381883 | 1381983 |
| Marker1061 | LG1 | 1413466 | 1413416 | 1413516 |
| Marker1076 | LG1 | 1462371 | 1462321 | 1462421 |
| Marker1077 | LG1 | 1462664 | 1462614 | 1462714 |
| Marker1093 | LG1 | 1501280 | 1501230 | 1501330 |
| Marker1125 | LG1 | 1552268 | 1552218 | 1552318 |
| Marker1135 | LG1 | 1583757 | 1583707 | 1583807 |
| Marker1154 | LG1 | 1598195 | 1598145 | 1598245 |
| Marker1167 | LG1 | 1603160 | 1603110 | 1603210 |
| Marker1170 | LG1 | 1603385 | 1603335 | 1603435 |
| Marker1172 | LG1 | 1605519 | 1605469 | 1605569 |
| Marker1184 | LG1 | 1613094 | 1613044 | 1613144 |
| Marker1185 | LG1 | 1617105 | 1617055 | 1617155 |
| Marker1209 | LG1 | 1651498 | 1651448 | 1651548 |
| Marker1225 | LG1 | 1657122 | 1657072 | 1657172 |
| Marker1258 | LG1 | 1701487 | 1701437 | 1701537 |
| Marker1290 | LG1 | 1758803 | 1758753 | 1758853 |
| Marker1294 | LG1 | 1762527 | 1762477 | 1762577 |
| Marker1326 | LG1 | 1862196 | 1862146 | 1862246 |
| Marker1343 | LG1 | 1889433 | 1889383 | 1889483 |
| Marker1344 | LG1 | 1894902 | 1894852 | 1894952 |
| Marker1345 | LG1 | 1898873 | 1898823 | 1898923 |
| Marker1350 | LG1 | 1899100 | 1899050 | 1899150 |
| Marker1361 | LG1 | 1927205 | 1927155 | 1927255 |
| Marker1364 | LG1 | 1928826 | 1928776 | 1928876 |
| Marker902 | LG1 | 2042308 | 2042258 | 2042358 |
| Marker944 | LG1 | 2135880 | 2135830 | 2135930 |
| Marker949 | LG1 | 2149349 | 2149299 | 2149399 |
| Marker993 | LG1 | 2195159 | 2195109 | 2195209 |
| Marker994 | LG1 | 2195390 | 2195340 | 2195440 |
| Marker1013 | LG1 | 2219696 | 2219646 | 2219746 |
| Marker1019 | LG1 | 2225354 | 2225304 | 2225404 |
| Marker676 | LG1 | 2276845 | 2276795 | 2276895 |
| Marker683 | LG1 | 2310056 | 2310006 | 2310106 |
| Marker686 | LG1 | 2314077 | 2314027 | 2314127 |
| Marker707 | LG1 | 2353683 | 2353633 | 2353733 |
| Marker710 | LG1 | 2365193 | 2365143 | 2365243 |
| Marker719 | LG1 | 2387459 | 2387409 | 2387509 |
| Marker727 | LG1 | 2389827 | 2389777 | 2389877 |
| Marker735 | LG1 | 2393684 | 2393634 | 2393734 |
| Marker760 | LG1 | 2452191 | 2452141 | 2452241 |
| Marker782 | LG1 | 2477720 | 2477670 | 2477770 |
| Marker787 | LG1 | 2483519 | 2483469 | 2483569 |
| Marker796 | LG1 | 2494734 | 2494684 | 2494784 |
| Marker809 | LG1 | 2506815 | 2506765 | 2506865 |
| Marker822 | LG1 | 2527966 | 2527916 | 2528016 |
| Marker828 | LG1 | 2533456 | 2533406 | 2533506 |
| Marker849 | LG1 | 2558477 | 2558427 | 2558527 |
| Marker863 | LG1 | 2609513 | 2609463 | 2609563 |
| Marker885 | LG1 | 2635803 | 2635753 | 2635853 |
| Marker382 | LG1 | 2659673 | 2659623 | 2659723 |
| Marker383 | LG1 | 2661844 | 2661794 | 2661894 |
| Marker388 | LG1 | 2670213 | 2670163 | 2670263 |
| Marker389 | LG1 | 2670424 | 2670374 | 2670474 |
| Marker407 | LG1 | 2694788 | 2694738 | 2694838 |
| Marker415 | LG1 | 2720738 | 2720688 | 2720788 |
| Marker431 | LG1 | 2738364 | 2738314 | 2738414 |
| Marker464 | LG1 | 2774173 | 2774123 | 2774223 |
| Marker484 | LG1 | 2796608 | 2796558 | 2796658 |
| Marker487 | LG1 | 2797484 | 2797434 | 2797534 |
| Marker503 | LG1 | 2811279 | 2811229 | 2811329 |
| Marker507 | LG1 | 2824133 | 2824083 | 2824183 |
| Marker518 | LG1 | 2838489 | 2838439 | 2838539 |
| Marker522 | LG1 | 2838903 | 2838853 | 2838953 |
| Marker529 | LG1 | 2840431 | 2840381 | 2840481 |
| Marker533 | LG1 | 2849630 | 2849580 | 2849680 |
| Marker538 | LG1 | 2853852 | 2853802 | 2853902 |
| Marker540 | LG1 | 2858021 | 2857971 | 2858071 |
| Marker555 | LG1 | 2898250 | 2898200 | 2898300 |
| Marker564 | LG1 | 2914372 | 2914322 | 2914422 |
| Marker565 | LG1 | 2915564 | 2915514 | 2915614 |
| Marker566 | LG1 | 2916182 | 2916132 | 2916232 |
| Marker567 | LG1 | 2916455 | 2916405 | 2916505 |
| Marker582 | LG1 | 2924895 | 2924845 | 2924945 |
| Marker611 | LG1 | 2952023 | 2951973 | 2952073 |
| Marker613 | LG1 | 2952033 | 2951983 | 2952083 |
| Marker617 | LG1 | 2953696 | 2953646 | 2953746 |
| Marker624 | LG1 | 2957016 | 2956966 | 2957066 |
| Marker1717 | LG1 | 3039268 | 3039218 | 3039318 |
| Marker1728 | LG1 | 3069711 | 3069661 | 3069761 |
| Marker1729 | LG1 | 3075424 | 3075374 | 3075474 |
| Marker1737 | LG1 | 3086180 | 3086130 | 3086230 |
| Marker1765 | LG1 | 3120452 | 3120402 | 3120502 |
| Marker1766 | LG1 | 3122874 | 3122824 | 3122924 |
| Marker1791 | LG1 | 3160845 | 3160795 | 3160895 |
| Marker1800 | LG1 | 3167128 | 3167078 | 3167178 |
| Marker1805 | LG1 | 3174877 | 3174827 | 3174927 |
| Marker1821 | LG1 | 3197983 | 3197933 | 3198033 |
| Marker1823 | LG1 | 3199532 | 3199482 | 3199582 |
| Marker1843 | LG1 | 3231348 | 3231298 | 3231398 |
| Marker1845 | LG1 | 3232513 | 3232463 | 3232563 |
| Marker1849 | LG1 | 3235397 | 3235347 | 3235447 |
| Marker1906 | LG1 | 3343744 | 3343694 | 3343794 |
| Marker1915 | LG1 | 3360154 | 3360104 | 3360204 |
| Marker1916 | LG1 | 3361606 | 3361556 | 3361656 |
| Marker1917 | LG1 | 3361775 | 3361725 | 3361825 |
| Marker1935 | LG1 | 3374346 | 3374296 | 3374396 |
| Marker1965 | LG1 | 3417043 | 3416993 | 3417093 |
| Marker1968 | LG1 | 3417255 | 3417205 | 3417305 |
| Marker1984 | LG1 | 3441381 | 3441331 | 3441431 |
| Marker1985 | LG1 | 3454508 | 3454458 | 3454558 |
| Marker1989 | LG1 | 3462004 | 3461954 | 3462054 |
| Marker2000 | LG1 | 3490285 | 3490235 | 3490335 |
| Marker2008 | LG1 | 3503565 | 3503515 | 3503615 |
| Marker2010 | LG1 | 3503588 | 3503538 | 3503638 |
| Marker2014 | LG1 | 3506289 | 3506239 | 3506339 |
| Marker2035 | LG1 | 3534393 | 3534343 | 3534443 |
| Marker2038 | LG1 | 3534948 | 3534898 | 3534998 |
| Marker63071 | LG1 | 3629138 | 3629088 | 3629188 |
| Marker1705 | LG1 | 3690638 | 3690588 | 3690688 |
| Marker1682 | LG1 | 3748693 | 3748643 | 3748743 |
| Marker1651 | LG1 | 3772607 | 3772557 | 3772657 |
| Marker1650 | LG1 | 3772846 | 3772796 | 3772896 |
| Marker1627 | LG1 | 3802752 | 3802702 | 3802802 |
| Marker1625 | LG1 | 3802826 | 3802776 | 3802876 |
| Marker1618 | LG1 | 3812202 | 3812152 | 3812252 |
| Marker1616 | LG1 | 3829266 | 3829216 | 3829316 |
| Marker1613 | LG1 | 3853528 | 3853478 | 3853578 |
| Marker1611 | LG1 | 3853988 | 3853938 | 3854038 |
| Marker1603 | LG1 | 3869118 | 3869068 | 3869168 |
| Marker1569 | LG1 | 3878556 | 3878506 | 3878606 |
| Marker1582 | LG1 | 3905052 | 3905002 | 3905102 |
| Marker1592 | LG1 | 3921159 | 3921109 | 3921209 |
| Marker1597 | LG1 | 3927678 | 3927628 | 3927728 |
| Marker2106 | LG1 | 3949690 | 3949640 | 3949740 |
| Marker2139 | LG1 | 4007939 | 4007889 | 4007989 |
| Marker2174 | LG1 | 4052654 | 4052604 | 4052704 |
| Marker2176 | LG1 | 4055703 | 4055653 | 4055753 |
| Marker2178 | LG1 | 4057033 | 4056983 | 4057083 |
| Marker2180 | LG1 | 4060613 | 4060563 | 4060663 |
| Marker2225 | LG1 | 4126763 | 4126713 | 4126813 |
| Marker2265 | LG1 | 4170140 | 4170090 | 4170190 |
| Marker2281 | LG1 | 4199351 | 4199301 | 4199401 |
| Marker2286 | LG1 | 4210845 | 4210795 | 4210895 |
| Marker2307 | LG1 | 4246421 | 4246371 | 4246471 |
| Marker2316 | LG1 | 4257286 | 4257236 | 4257336 |
| Marker2319 | LG1 | 4259500 | 4259450 | 4259550 |
| Marker2325 | LG1 | 4279162 | 4279112 | 4279212 |
| Marker2372 | LG1 | 4323774 | 4323724 | 4323824 |
| Marker2376 | LG1 | 4345452 | 4345402 | 4345502 |
| Marker2395 | LG1 | 4406628 | 4406578 | 4406678 |
| Marker2396 | LG1 | 4406710 | 4406660 | 4406760 |
| Marker2412 | LG1 | 4414956 | 4414906 | 4415006 |
| Marker2418 | LG1 | 4432570 | 4432520 | 4432620 |
| Marker2433 | LG1 | 4504447 | 4504397 | 4504497 |
| Marker2448 | LG1 | 4528557 | 4528507 | 4528607 |
| Marker3197 | LG1 | 4585961 | 4585911 | 4586011 |
| Marker3195 | LG1 | 4587026 | 4586976 | 4587076 |
| Marker3191 | LG1 | 4587245 | 4587195 | 4587295 |
| Marker3187 | LG1 | 4594033 | 4593983 | 4594083 |
| Marker3180 | LG1 | 4610460 | 4610410 | 4610510 |
| Marker3088 | LG1 | 4714240 | 4714190 | 4714290 |
| Marker3087 | LG1 | 4714762 | 4714712 | 4714812 |
| Marker3056 | LG1 | 4788742 | 4788692 | 4788792 |
| Marker3054 | LG1 | 4790000 | 4789950 | 4790050 |
| Marker3053 | LG1 | 4790235 | 4790185 | 4790285 |
| Marker3038 | LG1 | 4818508 | 4818458 | 4818558 |
| Marker2995 | LG1 | 4887613 | 4887563 | 4887663 |
| Marker2986 | LG1 | 4887834 | 4887784 | 4887884 |
| Marker2947 | LG1 | 4895390 | 4895340 | 4895440 |
| Marker2933 | LG1 | 4918582 | 4918532 | 4918632 |
| Marker2927 | LG1 | 4923427 | 4923377 | 4923477 |
| Marker2925 | LG1 | 4926549 | 4926499 | 4926599 |
| Marker2920 | LG1 | 4932921 | 4932871 | 4932971 |
| Marker2896 | LG1 | 4968484 | 4968434 | 4968534 |
| Marker2879 | LG1 | 4993675 | 4993625 | 4993725 |
| Marker2874 | LG1 | 5019307 | 5019257 | 5019357 |
| Marker2834 | LG1 | 5080536 | 5080486 | 5080586 |
| Marker2830 | LG1 | 5080574 | 5080524 | 5080624 |
| Marker2819 | LG1 | 5086271 | 5086221 | 5086321 |
| Marker2795 | LG1 | 5121403 | 5121353 | 5121453 |
| Marker2793 | LG1 | 5123847 | 5123797 | 5123897 |
| Marker2792 | LG1 | 5123911 | 5123861 | 5123961 |
| Marker2776 | LG1 | 5141920 | 5141870 | 5141970 |
| Marker2769 | LG1 | 5144153 | 5144103 | 5144203 |
| Marker2766 | LG1 | 5150446 | 5150396 | 5150496 |
| Marker56914 | LG1 | 5164422 | 5164372 | 5164472 |
| Marker2727 | LG1 | 5212021 | 5211971 | 5212071 |
| Marker2725 | LG1 | 5215351 | 5215301 | 5215401 |
| Marker2708 | LG1 | 5234618 | 5234568 | 5234668 |
| Marker2701 | LG1 | 5246157 | 5246107 | 5246207 |
| Marker2684 | LG1 | 5461760 | 5461710 | 5461810 |
| Marker106231 | LG1 | 5498528 | 5498478 | 5498578 |
| Marker2649 | LG1 | 5507518 | 5507468 | 5507568 |
| Marker2580 | LG1 | 5562779 | 5562729 | 5562829 |
| Marker2526 | LG1 | 5622164 | 5622114 | 5622214 |
| Marker2504 | LG1 | 5653680 | 5653630 | 5653730 |
| Marker2503 | LG1 | 5656270 | 5656220 | 5656320 |
| Marker2470 | LG1 | 5699916 | 5699866 | 5699966 |
| Marker3521 | LG1 | 5740300 | 5740250 | 5740350 |
| Marker3515 | LG1 | 5750472 | 5750422 | 5750522 |
| Marker3514 | LG1 | 5756996 | 5756946 | 5757046 |
| Marker3500 | LG1 | 5761076 | 5761026 | 5761126 |
| Marker3497 | LG1 | 5764803 | 5764753 | 5764853 |
| Marker3474 | LG1 | 5849202 | 5849152 | 5849252 |
| Marker3464 | LG1 | 5883316 | 5883266 | 5883366 |
| Marker3462 | LG1 | 5893175 | 5893125 | 5893225 |
| Marker3445 | LG1 | 5911957 | 5911907 | 5912007 |
| Marker3433 | LG1 | 5918904 | 5918854 | 5918954 |
| Marker3416 | LG1 | 5945407 | 5945357 | 5945457 |
| Marker3410 | LG1 | 5950054 | 5950004 | 5950104 |
| Marker3409 | LG1 | 5952040 | 5951990 | 5952090 |
| Marker3404 | LG1 | 5964082 | 5964032 | 5964132 |
| Marker3399 | LG1 | 5978566 | 5978516 | 5978616 |
| Marker3392 | LG1 | 5997528 | 5997478 | 5997578 |
| Marker3373 | LG1 | 6023249 | 6023199 | 6023299 |
| Marker3372 | LG1 | 6023489 | 6023439 | 6023539 |
| Marker3369 | LG1 | 6028940 | 6028890 | 6028990 |
| Marker3363 | LG1 | 6029480 | 6029430 | 6029530 |
| Marker3351 | LG1 | 6041322 | 6041272 | 6041372 |
| Marker3343 | LG1 | 6057996 | 6057946 | 6058046 |
| Marker3293 | LG1 | 6144589 | 6144539 | 6144639 |
| Marker3289 | LG1 | 6149668 | 6149618 | 6149718 |
| Marker3271 | LG1 | 6170469 | 6170419 | 6170519 |
| Marker3264 | LG1 | 6180669 | 6180619 | 6180719 |
| Marker3215 | LG1 | 6222614 | 6222564 | 6222664 |
| Marker3547 | LG1 | 6245248 | 6245198 | 6245298 |
| Marker3549 | LG1 | 6249964 | 6249914 | 6250014 |
| Marker3558 | LG1 | 6276752 | 6276702 | 6276802 |
| Marker3563 | LG1 | 6277487 | 6277437 | 6277537 |
| Marker3575 | LG1 | 6303966 | 6303916 | 6304016 |
| Marker3577 | LG1 | 6304232 | 6304182 | 6304282 |
| Marker3578 | LG1 | 6311193 | 6311143 | 6311243 |
| Marker3588 | LG1 | 6337268 | 6337218 | 6337318 |
| Marker3595 | LG1 | 6348334 | 6348284 | 6348384 |
| Marker3599 | LG1 | 6350847 | 6350797 | 6350897 |
| Marker3601 | LG1 | 6357632 | 6357582 | 6357682 |
| Marker3608 | LG1 | 6364121 | 6364071 | 6364171 |
| Marker3632 | LG1 | 6380106 | 6380056 | 6380156 |
| Marker3661 | LG1 | 6408983 | 6408933 | 6409033 |
| Marker3678 | LG1 | 6439660 | 6439610 | 6439710 |
| Marker3683 | LG1 | 6451829 | 6451779 | 6451879 |
| Marker3695 | LG1 | 6483610 | 6483560 | 6483660 |
| Marker3699 | LG1 | 6483990 | 6483940 | 6484040 |
| Marker3718 | LG1 | 6497102 | 6497052 | 6497152 |
| Marker3731 | LG1 | 6499881 | 6499831 | 6499931 |
| Marker3753 | LG1 | 6571987 | 6571937 | 6572037 |
| Marker3756 | LG1 | 6572123 | 6572073 | 6572173 |
| Marker3773 | LG1 | 6578691 | 6578641 | 6578741 |
| Marker3785 | LG1 | 6616907 | 6616857 | 6616957 |
| Marker117899 | LG1 | 6679960 | 6679910 | 6680010 |
| Marker117893 | LG1 | 6687562 | 6687512 | 6687612 |
| Marker117881 | LG1 | 6709160 | 6709110 | 6709210 |
| Marker3812 | LG1 | 6794797 | 6794747 | 6794847 |
| Marker3815 | LG1 | 6797110 | 6797060 | 6797160 |
| Marker3824 | LG1 | 6835564 | 6835514 | 6835614 |
| Marker3924 | LG1 | 6971041 | 6970991 | 6971091 |
| Marker3925 | LG1 | 6971248 | 6971198 | 6971298 |
| Marker3959 | LG1 | 6997064 | 6997014 | 6997114 |
| Marker3961 | LG1 | 6997695 | 6997645 | 6997745 |
| Marker3972 | LG1 | 7022767 | 7022717 | 7022817 |
| Marker3979 | LG1 | 7043767 | 7043717 | 7043817 |
| Marker3999 | LG1 | 7061589 | 7061539 | 7061639 |
| Marker4002 | LG1 | 7061851 | 7061801 | 7061901 |
| Marker4016 | LG1 | 7072025 | 7071975 | 7072075 |
| Marker4074 | LG1 | 7121944 | 7121894 | 7121994 |
| Marker121593 | LG1 | 7129523 | 7129473 | 7129573 |
| Marker4113 | LG1 | 7172043 | 7171993 | 7172093 |
| Marker4144 | LG1 | 7200781 | 7200731 | 7200831 |
| Marker4149 | LG1 | 7201048 | 7200998 | 7201098 |
| Marker4171 | LG1 | 7222435 | 7222385 | 7222485 |
| Marker4208 | LG1 | 7282775 | 7282725 | 7282825 |
| Marker4228 | LG1 | 7359312 | 7359262 | 7359362 |
| Marker4234 | LG1 | 7372078 | 7372028 | 7372128 |
| Marker4267 | LG1 | 7411436 | 7411386 | 7411486 |
| Marker4293 | LG1 | 7499891 | 7499841 | 7499941 |
| Marker4299 | LG1 | 7508434 | 7508384 | 7508484 |
| Marker4300 | LG1 | 7508980 | 7508930 | 7509030 |
| Marker4321 | LG1 | 7588878 | 7588828 | 7588928 |
| Marker4327 | LG1 | 7598573 | 7598523 | 7598623 |
| Marker4092 | LG1 | 7662424 | 7662374 | 7662474 |
| Marker4342 | LG1 | 7685948 | 7685898 | 7685998 |
| Marker4359 | LG1 | 7724286 | 7724236 | 7724336 |
| Marker4387 | LG1 | 7755109 | 7755059 | 7755159 |
| Marker4399 | LG1 | 7767744 | 7767694 | 7767794 |
| Marker4400 | LG1 | 7768013 | 7767963 | 7768063 |
| Marker4417 | LG1 | 7798920 | 7798870 | 7798970 |
| Marker4418 | LG1 | 7809047 | 7808997 | 7809097 |
| Marker4420 | LG1 | 7809304 | 7809254 | 7809354 |
| Marker4445 | LG1 | 7856228 | 7856178 | 7856278 |
| Marker4451 | LG1 | 7884503 | 7884453 | 7884553 |
| Marker4488 | LG1 | 7971364 | 7971314 | 7971414 |
| Marker4490 | LG1 | 7976448 | 7976398 | 7976498 |
| Marker4500 | LG1 | 8004830 | 8004780 | 8004880 |
| Marker4508 | LG1 | 8034061 | 8034011 | 8034111 |
| Marker4520 | LG1 | 8051018 | 8050968 | 8051068 |
| Marker4524 | LG1 | 8059761 | 8059711 | 8059811 |
| Marker4529 | LG1 | 8091049 | 8090999 | 8091099 |
| Marker4567 | LG1 | 8176782 | 8176732 | 8176832 |
| Marker4609 | LG1 | 8256133 | 8256083 | 8256183 |
| Marker4630 | LG1 | 8285658 | 8285608 | 8285708 |
| Marker4635 | LG1 | 8285902 | 8285852 | 8285952 |
| Marker4660 | LG1 | 8324059 | 8324009 | 8324109 |
| Marker4661 | LG1 | 8324240 | 8324190 | 8324290 |
| Marker4664 | LG1 | 8327188 | 8327138 | 8327238 |
| Marker4670 | LG1 | 8333061 | 8333011 | 8333111 |
| Marker4791 | LG1 | 8564532 | 8564482 | 8564582 |
| Marker4762 | LG1 | 8589719 | 8589669 | 8589769 |
| Marker5526 | LG1 | 8752079 | 8752029 | 8752129 |
| Marker5722 | LG1 | 8967252 | 8967202 | 8967302 |
| Marker4974 | LG1 | 9327422 | 9327372 | 9327472 |
| Marker4967 | LG1 | 9351521 | 9351471 | 9351571 |
| Marker5324 | LG1 | 9373257 | 9373207 | 9373307 |
| Marker5303 | LG1 | 9391767 | 9391717 | 9391817 |
| Marker5216 | LG1 | 9581521 | 9581471 | 9581571 |
| Marker4954 | LG1 | 9744011 | 9743961 | 9744061 |
| Marker4934 | LG1 | 9753601 | 9753551 | 9753651 |
| Marker4915 | LG1 | 9823302 | 9823252 | 9823352 |
| Marker4888 | LG1 | 9880807 | 9880757 | 9880857 |
| Marker4876 | LG1 | 9919224 | 9919174 | 9919274 |
| Marker4870 | LG1 | 9926286 | 9926236 | 9926336 |
| Marker4861 | LG1 | 9929943 | 9929893 | 9929993 |
| Marker5644 | LG1 | 10470651 | 10470601 | 10470701 |
| Marker5636 | LG1 | 10473339 | 10473289 | 10473389 |
| Marker5421 | LG1 | 10722918 | 10722868 | 10722968 |
| Marker5683 | LG1 | 10894686 | 10894636 | 10894736 |
| Marker5877 | LG1 | 10925984 | 10925934 | 10926034 |
| Marker6012 | LG1 | 11091791 | 11091741 | 11091841 |
| Marker6022 | LG1 | 11111093 | 11111043 | 11111143 |
| Marker6103 | LG1 | 11204495 | 11204445 | 11204545 |
| Marker5804 | LG1 | 11260294 | 11260244 | 11260344 |
| Marker5791 | LG1 | 11276606 | 11276556 | 11276656 |
| Marker5779 | LG1 | 11291265 | 11291215 | 11291315 |
| Marker5768 | LG1 | 11296056 | 11296006 | 11296106 |
| Marker5745 | LG1 | 11364652 | 11364602 | 11364702 |
| Marker6104 | LG1 | 11368897 | 11368847 | 11368947 |
| Marker6106 | LG1 | 11374720 | 11374670 | 11374770 |
| Marker6151 | LG1 | 11410102 | 11410052 | 11410152 |
| Marker6249 | LG1 | 11501880 | 11501830 | 11501930 |
| Marker6254 | LG1 | 11511669 | 11511619 | 11511719 |
| Marker6265 | LG1 | 11523468 | 11523418 | 11523518 |
| Marker6275 | LG1 | 11540564 | 11540514 | 11540614 |
| Marker6281 | LG1 | 11540865 | 11540815 | 11540915 |
| Marker6358 | LG1 | 11633763 | 11633713 | 11633813 |
| Marker6374 | LG1 | 11748368 | 11748318 | 11748418 |
| Marker7505 | LG1 | 11886557 | 11886507 | 11886607 |
| Marker7508 | LG1 | 11888749 | 11888699 | 11888799 |
| Marker7529 | LG1 | 11894934 | 11894884 | 11894984 |
| Marker7394 | LG1 | 12150127 | 12150077 | 12150177 |
| Marker7317 | LG1 | 12219438 | 12219388 | 12219488 |
| Marker7312 | LG1 | 12220852 | 12220802 | 12220902 |
| Marker7184 | LG1 | 12404183 | 12404133 | 12404233 |
| Marker7164 | LG1 | 12418051 | 12418001 | 12418101 |
| Marker112721 | LG1 | 12516388 | 12516338 | 12516438 |
| Marker112735 | LG1 | 12517810 | 12517760 | 12517860 |
| Marker112742 | LG1 | 12522696 | 12522646 | 12522746 |
| Marker112747 | LG1 | 12535844 | 12535794 | 12535894 |
| Marker112844 | LG1 | 12617956 | 12617906 | 12618006 |
| Marker6872 | LG1 | 12692435 | 12692385 | 12692485 |
| Marker6889 | LG1 | 12728313 | 12728263 | 12728363 |
| Marker6906 | LG1 | 12758109 | 12758059 | 12758159 |
| Marker6913 | LG1 | 12763417 | 12763367 | 12763467 |
| Marker6915 | LG1 | 12763790 | 12763740 | 12763840 |
| Marker6933 | LG1 | 12793709 | 12793659 | 12793759 |
| Marker6939 | LG1 | 12797904 | 12797854 | 12797954 |
| Marker118691 | LG1 | 12891552 | 12891502 | 12891602 |
| Marker118685 | LG1 | 12892125 | 12892075 | 12892175 |
| Marker7700 | LG1 | 13131339 | 13131289 | 13131389 |
| Marker7697 | LG1 | 13135485 | 13135435 | 13135535 |
| Marker7656 | LG1 | 13242813 | 13242763 | 13242863 |
| Marker9814 | LG1 | 13287507 | 13287457 | 13287557 |
| Marker9823 | LG1 | 13302606 | 13302556 | 13302656 |
| Marker114234 | LG1 | 13331752 | 13331702 | 13331802 |
| Marker114232 | LG1 | 13332910 | 13332860 | 13332960 |
| Marker114198 | LG1 | 13352816 | 13352766 | 13352866 |
| Marker682 | LG1 | 13406883 | 13406833 | 13406933 |
| Marker114121 | LG1 | 13428755 | 13428705 | 13428805 |
| Marker121666 | LG1 | 13874548 | 13874498 | 13874598 |
| Marker54526 | LG1 | 14256109 | 14256059 | 14256159 |
| Marker8576 | LG1 | 14327828 | 14327778 | 14327878 |
| Marker8729 | LG1 | 14807094 | 14807044 | 14807144 |
| Marker8788 | LG1 | 14876702 | 14876652 | 14876752 |
| Marker9030 | LG1 | 15211864 | 15211814 | 15211914 |
| Marker9050 | LG1 | 15257488 | 15257438 | 15257538 |
| Marker9069 | LG1 | 15273194 | 15273144 | 15273244 |
| Marker9077 | LG1 | 15281248 | 15281198 | 15281298 |
| Marker44209 | LG1 | 15353346 | 15353296 | 15353396 |
| Marker9192 | LG1 | 15435159 | 15435109 | 15435209 |
| Marker9200 | LG1 | 15451916 | 15451866 | 15451966 |
| Marker9243 | LG1 | 15535708 | 15535658 | 15535758 |
| Marker9255 | LG1 | 15541310 | 15541260 | 15541360 |
| Marker9500 | LG1 | 15808096 | 15808046 | 15808146 |
| Marker9501 | LG1 | 15808318 | 15808268 | 15808368 |
| Marker9519 | LG1 | 15828223 | 15828173 | 15828273 |
| Marker89707 | LG1 | 15855280 | 15855230 | 15855330 |
| Marker9548 | LG1 | 15872733 | 15872683 | 15872783 |
| Marker9561 | LG1 | 15887304 | 15887254 | 15887354 |
| Marker9566 | LG1 | 15905078 | 15905028 | 15905128 |
| Marker57880 | LG1 | 15932832 | 15932782 | 15932882 |
| Marker9585 | LG1 | 15952223 | 15952173 | 15952273 |
| Marker9595 | LG1 | 15972519 | 15972469 | 15972569 |
| Marker9597 | LG1 | 15972772 | 15972722 | 15972822 |
| Marker9652 | LG1 | 16060426 | 16060376 | 16060476 |
| Marker9674 | LG1 | 16090389 | 16090339 | 16090439 |
| Marker9679 | LG1 | 16094753 | 16094703 | 16094803 |
| Marker9681 | LG1 | 16097756 | 16097706 | 16097806 |
| Marker9701 | LG1 | 16136535 | 16136485 | 16136585 |
| Marker9984 | LG1 | 16227445 | 16227395 | 16227495 |
| Marker9956 | LG1 | 16247189 | 16247139 | 16247239 |
| Marker9928 | LG1 | 16269977 | 16269927 | 16270027 |
| Marker9920 | LG1 | 16276258 | 16276208 | 16276308 |
| Marker9919 | LG1 | 16276512 | 16276462 | 16276562 |
| Marker9474 | LG1 | 16317060 | 16317010 | 16317110 |
| Marker9472 | LG1 | 16328173 | 16328123 | 16328223 |
| Marker10025 | LG1 | 16448661 | 16448611 | 16448711 |
| Marker10049 | LG1 | 16501864 | 16501814 | 16501914 |
| Marker10058 | LG1 | 16504280 | 16504230 | 16504330 |
| Marker10093 | LG1 | 16560874 | 16560824 | 16560924 |
| Marker10456 | LG1 | 17257586 | 17257536 | 17257636 |
| Marker10460 | LG1 | 17259589 | 17259539 | 17259639 |
| Marker10537 | LG1 | 17343711 | 17343661 | 17343761 |
| Marker110739 | LG1 | 17370262 | 17370212 | 17370312 |
| Marker110661 | LG1 | 17462057 | 17462007 | 17462107 |
| Marker110653 | LG1 | 17473861 | 17473811 | 17473911 |
| Marker110652 | LG1 | 17473990 | 17473940 | 17474040 |
| Marker110647 | LG1 | 17482683 | 17482633 | 17482733 |
| Marker110516 | LG1 | 17756394 | 17756344 | 17756444 |
| Marker110497 | LG1 | 17766676 | 17766626 | 17766726 |
| Marker10565 | LG1 | 18028763 | 18028713 | 18028813 |
| Marker11861 | LG1 | 18165750 | 18165700 | 18165800 |
| Marker11860 | LG1 | 18165938 | 18165888 | 18165988 |
| Marker11804 | LG1 | 18243971 | 18243921 | 18244021 |
| Marker11790 | LG1 | 18255100 | 18255050 | 18255150 |
| Marker11769 | LG1 | 18320675 | 18320625 | 18320725 |
| Marker11719 | LG1 | 18425050 | 18425000 | 18425100 |
| Marker11718 | LG1 | 18425287 | 18425237 | 18425337 |
| Marker11717 | LG1 | 18426221 | 18426171 | 18426271 |
| Marker11713 | LG1 | 18426465 | 18426415 | 18426515 |
| Marker11557 | LG1 | 18471959 | 18471909 | 18472009 |
| Marker11563 | LG1 | 18473942 | 18473892 | 18473992 |
| Marker11606 | LG1 | 18563290 | 18563240 | 18563340 |
| Marker11673 | LG1 | 18629423 | 18629373 | 18629473 |
| Marker11537 | LG1 | 18727928 | 18727878 | 18727978 |
| Marker11515 | LG1 | 18764485 | 18764435 | 18764535 |
| Marker11421 | LG1 | 18885493 | 18885443 | 18885543 |
| Marker11410 | LG1 | 18894540 | 18894490 | 18894590 |
| Marker11404 | LG1 | 18894712 | 18894662 | 18894762 |
| Marker11386 | LG1 | 18945814 | 18945764 | 18945864 |
| Marker11296 | LG1 | 19060592 | 19060542 | 19060642 |
| Marker11293 | LG1 | 19061205 | 19061155 | 19061255 |
| Marker11272 | LG1 | 19075782 | 19075732 | 19075832 |
| Marker11271 | LG1 | 19090353 | 19090303 | 19090403 |
| Marker11266 | LG1 | 19092836 | 19092786 | 19092886 |
| Marker11250 | LG1 | 19117396 | 19117346 | 19117446 |
| Marker11235 | LG1 | 19134987 | 19134937 | 19135037 |
| Marker11219 | LG1 | 19149719 | 19149669 | 19149769 |
| Marker11216 | LG1 | 19150928 | 19150878 | 19150978 |
| Marker11186 | LG1 | 19192044 | 19191994 | 19192094 |
| Marker11185 | LG1 | 19192960 | 19192910 | 19193010 |
| Marker11174 | LG1 | 19198102 | 19198052 | 19198152 |
| Marker11161 | LG1 | 19217724 | 19217674 | 19217774 |
| Marker11109 | LG1 | 19242254 | 19242204 | 19242304 |
| Marker11095 | LG1 | 19249514 | 19249464 | 19249564 |
| Marker11063 | LG1 | 19276702 | 19276652 | 19276752 |
| Marker11024 | LG1 | 19286497 | 19286447 | 19286547 |
| Marker11020 | LG1 | 19291807 | 19291757 | 19291857 |
| Marker10989 | LG1 | 19320672 | 19320622 | 19320722 |
| Marker10963 | LG1 | 19394130 | 19394080 | 19394180 |
| Marker10937 | LG1 | 19421815 | 19421765 | 19421865 |
| Marker7835 | LG1 | 19700515 | 19700465 | 19700565 |
| Marker7836 | LG1 | 19700721 | 19700671 | 19700771 |
| Marker12195 | LG1 | 20698215 | 20698165 | 20698265 |
| Marker13361 | LG1 | 20715508 | 20715458 | 20715558 |
| Marker13353 | LG1 | 20749202 | 20749152 | 20749252 |
| Marker13352 | LG1 | 20749355 | 20749305 | 20749405 |
| Marker13346 | LG1 | 20757213 | 20757163 | 20757263 |
| Marker13330 | LG1 | 20777012 | 20776962 | 20777062 |
| Marker13312 | LG1 | 20821847 | 20821797 | 20821897 |
| Marker13310 | LG1 | 20824890 | 20824840 | 20824940 |
| Marker13305 | LG1 | 20843506 | 20843456 | 20843556 |
| Marker13287 | LG1 | 20872818 | 20872768 | 20872868 |
| Marker13276 | LG1 | 20875472 | 20875422 | 20875522 |
| Marker13253 | LG1 | 20900265 | 20900215 | 20900315 |
| Marker13250 | LG1 | 20902385 | 20902335 | 20902435 |
| Marker13245 | LG1 | 20917019 | 20916969 | 20917069 |
| Marker13162 | LG1 | 21031918 | 21031868 | 21031968 |
| Marker13107 | LG1 | 21071382 | 21071332 | 21071432 |
| Marker13058 | LG1 | 21098792 | 21098742 | 21098842 |
| Marker12986 | LG1 | 21205420 | 21205370 | 21205470 |
| Marker12958 | LG1 | 21237537 | 21237487 | 21237587 |
| Marker12924 | LG1 | 21300979 | 21300929 | 21301029 |
| Marker12916 | LG1 | 21305303 | 21305253 | 21305353 |
| Marker61059 | LG1 | 21342158 | 21342108 | 21342208 |
| Marker12877 | LG1 | 21404735 | 21404685 | 21404785 |
| Marker12853 | LG1 | 21427420 | 21427370 | 21427470 |
| Marker12799 | LG1 | 21480339 | 21480289 | 21480389 |
| Marker12710 | LG1 | 21584837 | 21584787 | 21584887 |
| Marker12694 | LG1 | 21617301 | 21617251 | 21617351 |
| Marker12681 | LG1 | 21649752 | 21649702 | 21649802 |
| Marker12672 | LG1 | 21651057 | 21651007 | 21651107 |
| Marker12671 | LG1 | 21651467 | 21651417 | 21651517 |
| Marker12668 | LG1 | 21654901 | 21654851 | 21654951 |
| Marker12582 | LG1 | 21719839 | 21719789 | 21719889 |
| Marker13487 | LG1 | 21810099 | 21810049 | 21810149 |
| Marker13480 | LG1 | 21818609 | 21818559 | 21818659 |
| Marker13477 | LG1 | 21833445 | 21833395 | 21833495 |
| Marker13476 | LG1 | 21834865 | 21834815 | 21834915 |
| Marker13416 | LG1 | 21882201 | 21882151 | 21882251 |
| Marker13404 | LG1 | 21902448 | 21902398 | 21902498 |
| Marker13399 | LG1 | 21926177 | 21926127 | 21926227 |
| Marker13397 | LG1 | 21927466 | 21927416 | 21927516 |
| Marker13510 | LG1 | 22012160 | 22012110 | 22012210 |
| Marker13529 | LG1 | 22042567 | 22042517 | 22042617 |
| Marker13539 | LG1 | 22057301 | 22057251 | 22057351 |
| Marker12598 | LG1 | 22095390 | 22095340 | 22095440 |
| Marker13598 | LG1 | 22171624 | 22171574 | 22171674 |
| Marker13602 | LG1 | 22171957 | 22171907 | 22172007 |
| Marker13620 | LG1 | 22195407 | 22195357 | 22195457 |
| Marker13641 | LG1 | 22226845 | 22226795 | 22226895 |
| Marker13651 | LG1 | 22248874 | 22248824 | 22248924 |
| Marker13654 | LG1 | 22252025 | 22251975 | 22252075 |
| Marker13669 | LG1 | 22263848 | 22263798 | 22263898 |
| Marker13674 | LG1 | 22270985 | 22270935 | 22271035 |
| Marker13676 | LG1 | 22283360 | 22283310 | 22283410 |
| Marker13709 | LG1 | 22319267 | 22319217 | 22319317 |
| Marker13725 | LG1 | 22334215 | 22334165 | 22334265 |
| Marker13768 | LG1 | 22353028 | 22352978 | 22353078 |
| Marker13772 | LG1 | 22366227 | 22366177 | 22366277 |
| Marker13776 | LG1 | 22370711 | 22370661 | 22370761 |
| Marker13785 | LG1 | 22384090 | 22384040 | 22384140 |
| Marker13868 | LG1 | 22568732 | 22568682 | 22568782 |
| Marker13900 | LG1 | 22622335 | 22622285 | 22622385 |
| Marker13905 | LG1 | 22626130 | 22626080 | 22626180 |
| Marker13906 | LG1 | 22634838 | 22634788 | 22634888 |
| Marker14031 | LG1 | 22934403 | 22934353 | 22934453 |
| Marker14057 | LG1 | 22956594 | 22956544 | 22956644 |
| Marker14080 | LG1 | 23020379 | 23020329 | 23020429 |
| Marker14082 | LG1 | 23020532 | 23020482 | 23020582 |
| Marker14100 | LG1 | 23050735 | 23050685 | 23050785 |
| Marker14107 | LG1 | 23061653 | 23061603 | 23061703 |
| Marker14149 | LG1 | 23125781 | 23125731 | 23125831 |
| Marker14156 | LG1 | 23128416 | 23128366 | 23128466 |
| Marker14158 | LG1 | 23136293 | 23136243 | 23136343 |
| Marker14160 | LG1 | 23136519 | 23136469 | 23136569 |
| Marker14172 | LG1 | 23150189 | 23150139 | 23150239 |
| Marker14193 | LG1 | 23212911 | 23212861 | 23212961 |
| Marker14271 | LG1 | 23353669 | 23353619 | 23353719 |
| Marker14305 | LG1 | 23374339 | 23374289 | 23374389 |
| Marker14316 | LG1 | 23388780 | 23388730 | 23388830 |
| Marker14326 | LG1 | 23417929 | 23417879 | 23417979 |
| Marker14334 | LG1 | 23486955 | 23486905 | 23487005 |
| Marker14337 | LG1 | 23491202 | 23491152 | 23491252 |
| Marker14339 | LG1 | 23494280 | 23494230 | 23494330 |
| Marker14346 | LG1 | 23506165 | 23506115 | 23506215 |
| Marker14354 | LG1 | 23519031 | 23518981 | 23519081 |
| Marker14355 | LG1 | 23532211 | 23532161 | 23532261 |
| Marker14382 | LG1 | 23591382 | 23591332 | 23591432 |
| Marker14450 | LG1 | 23729792 | 23729742 | 23729842 |
| Marker14452 | LG1 | 23729963 | 23729913 | 23730013 |
| Marker14454 | LG1 | 23730800 | 23730750 | 23730850 |
| Marker14476 | LG1 | 23773046 | 23772996 | 23773096 |
| Marker14496 | LG1 | 23786751 | 23786701 | 23786801 |
| Marker14503 | LG1 | 23789678 | 23789628 | 23789728 |
| Marker14505 | LG1 | 23801338 | 23801288 | 23801388 |
| Marker14514 | LG1 | 23815755 | 23815705 | 23815805 |
| Marker14522 | LG1 | 23834839 | 23834789 | 23834889 |
| Marker14537 | LG1 | 23868611 | 23868561 | 23868661 |
| Marker14717 | LG1 | 23924360 | 23924310 | 23924410 |
| Marker14715 | LG1 | 23936171 | 23936121 | 23936221 |
| Marker14713 | LG1 | 23948383 | 23948333 | 23948433 |
| Marker14708 | LG1 | 23964887 | 23964837 | 23964937 |
| Marker14669 | LG1 | 24044377 | 24044327 | 24044427 |
| Marker14666 | LG1 | 24061094 | 24061044 | 24061144 |
| Marker14659 | LG1 | 24062858 | 24062808 | 24062908 |
| Marker14657 | LG1 | 24064289 | 24064239 | 24064339 |
| Marker14651 | LG1 | 24082288 | 24082238 | 24082338 |
| Marker14630 | LG1 | 24111058 | 24111008 | 24111108 |
| Marker14626 | LG1 | 24115914 | 24115864 | 24115964 |
| Marker14624 | LG1 | 24117680 | 24117630 | 24117730 |
| Marker14602 | LG1 | 24145159 | 24145109 | 24145209 |
| Marker36718 | LG2 | 41139 | 41089 | 41189 |
| Marker36783 | LG2 | 130755 | 130705 | 130805 |
| Marker36799 | LG2 | 170760 | 170710 | 170810 |
| Marker36834 | LG2 | 253392 | 253342 | 253442 |
| Marker36911 | LG2 | 381524 | 381474 | 381574 |
| Marker36929 | LG2 | 402186 | 402136 | 402236 |
| Marker36953 | LG2 | 416318 | 416268 | 416368 |
| Marker36960 | LG2 | 419604 | 419554 | 419654 |
| Marker36323 | LG2 | 533049 | 532999 | 533099 |
| Marker36335 | LG2 | 540725 | 540675 | 540775 |
| Marker36342 | LG2 | 546586 | 546536 | 546636 |
| Marker36354 | LG2 | 572678 | 572628 | 572728 |
| Marker36392 | LG2 | 662322 | 662272 | 662372 |
| Marker36394 | LG2 | 668968 | 668918 | 669018 |
| Marker36400 | LG2 | 680795 | 680745 | 680845 |
| Marker35789 | LG2 | 883867 | 883817 | 883917 |
| Marker35782 | LG2 | 899662 | 899612 | 899712 |
| Marker35781 | LG2 | 904221 | 904171 | 904271 |
| Marker35770 | LG2 | 962671 | 962621 | 962721 |
| Marker35763 | LG2 | 986625 | 986575 | 986675 |
| Marker35733 | LG2 | 1085404 | 1085354 | 1085454 |
| Marker35730 | LG2 | 1094903 | 1094853 | 1094953 |
| Marker35712 | LG2 | 1116705 | 1116655 | 1116755 |
| Marker35678 | LG2 | 1155896 | 1155846 | 1155946 |
| Marker35668 | LG2 | 1170617 | 1170567 | 1170667 |
| Marker35667 | LG2 | 1170646 | 1170596 | 1170696 |
| Marker35655 | LG2 | 1197484 | 1197434 | 1197534 |
| Marker35650 | LG2 | 1213025 | 1212975 | 1213075 |
| Marker35648 | LG2 | 1217545 | 1217495 | 1217595 |
| Marker35637 | LG2 | 1233709 | 1233659 | 1233759 |
| Marker35619 | LG2 | 1256932 | 1256882 | 1256982 |
| Marker35617 | LG2 | 1257128 | 1257078 | 1257178 |
| Marker35615 | LG2 | 1260197 | 1260147 | 1260247 |
| Marker35553 | LG2 | 1332710 | 1332660 | 1332760 |
| Marker35535 | LG2 | 1345608 | 1345558 | 1345658 |
| Marker35533 | LG2 | 1345775 | 1345725 | 1345825 |
| Marker35531 | LG2 | 1350475 | 1350425 | 1350525 |
| Marker35480 | LG2 | 1399812 | 1399762 | 1399862 |
| Marker35450 | LG2 | 1460843 | 1460793 | 1460893 |
| Marker35443 | LG2 | 1468019 | 1467969 | 1468069 |
| Marker35424 | LG2 | 1513742 | 1513692 | 1513792 |
| Marker35420 | LG2 | 1519039 | 1518989 | 1519089 |
| Marker35310 | LG2 | 1735606 | 1735556 | 1735656 |
| Marker35301 | LG2 | 1743516 | 1743466 | 1743566 |
| Marker35299 | LG2 | 1748792 | 1748742 | 1748842 |
| Marker35261 | LG2 | 1805221 | 1805171 | 1805271 |
| Marker35034 | LG2 | 1880145 | 1880095 | 1880195 |
| Marker36291 | LG2 | 1963461 | 1963411 | 1963511 |
| Marker36290 | LG2 | 1963674 | 1963624 | 1963724 |
| Marker36261 | LG2 | 2001232 | 2001182 | 2001282 |
| Marker36241 | LG2 | 2026887 | 2026837 | 2026937 |
| Marker36231 | LG2 | 2042461 | 2042411 | 2042511 |
| Marker36227 | LG2 | 2054375 | 2054325 | 2054425 |
| Marker36204 | LG2 | 2070364 | 2070314 | 2070414 |
| Marker36200 | LG2 | 2077448 | 2077398 | 2077498 |
| Marker36187 | LG2 | 2090195 | 2090145 | 2090245 |
| Marker36185 | LG2 | 2090531 | 2090481 | 2090581 |
| Marker36178 | LG2 | 2152602 | 2152552 | 2152652 |
| Marker36153 | LG2 | 2200418 | 2200368 | 2200468 |
| Marker36152 | LG2 | 2200644 | 2200594 | 2200694 |
| Marker36091 | LG2 | 2383585 | 2383535 | 2383635 |
| Marker36007 | LG2 | 2535166 | 2535116 | 2535216 |
| Marker56177 | LG2 | 2622689 | 2622639 | 2622739 |
| Marker35975 | LG2 | 2687525 | 2687475 | 2687575 |
| Marker35932 | LG2 | 2764913 | 2764863 | 2764963 |
| Marker35918 | LG2 | 2808145 | 2808095 | 2808195 |
| Marker35883 | LG2 | 2877027 | 2876977 | 2877077 |
| Marker35064 | LG2 | 2971816 | 2971766 | 2971866 |
| Marker35126 | LG2 | 3043325 | 3043275 | 3043375 |
| Marker35166 | LG2 | 3104383 | 3104333 | 3104433 |
| Marker35172 | LG2 | 3105670 | 3105620 | 3105720 |
| Marker35192 | LG2 | 3132745 | 3132695 | 3132795 |
| Marker35197 | LG2 | 3138108 | 3138058 | 3138158 |
| Marker35217 | LG2 | 3162306 | 3162256 | 3162356 |
| Marker34612 | LG2 | 3289422 | 3289372 | 3289472 |
| Marker34614 | LG2 | 3297050 | 3297000 | 3297100 |
| Marker34693 | LG2 | 3434195 | 3434145 | 3434245 |
| Marker34744 | LG2 | 3509085 | 3509035 | 3509135 |
| Marker34754 | LG2 | 3536171 | 3536121 | 3536221 |
| Marker34758 | LG2 | 3536383 | 3536333 | 3536433 |
| Marker34786 | LG2 | 3547585 | 3547535 | 3547635 |
| Marker34806 | LG2 | 3582163 | 3582113 | 3582213 |
| Marker34822 | LG2 | 3585305 | 3585255 | 3585355 |
| Marker34837 | LG2 | 3603461 | 3603411 | 3603511 |
| Marker34849 | LG2 | 3615799 | 3615749 | 3615849 |
| Marker34891 | LG2 | 3679599 | 3679549 | 3679649 |
| Marker34918 | LG2 | 3711111 | 3711061 | 3711161 |
| Marker34945 | LG2 | 3746324 | 3746274 | 3746374 |
| Marker34963 | LG2 | 3775424 | 3775374 | 3775474 |
| Marker34965 | LG2 | 3775632 | 3775582 | 3775682 |
| Marker34979 | LG2 | 3790246 | 3790196 | 3790296 |
| Marker34984 | LG2 | 3804554 | 3804504 | 3804604 |
| Marker42262 | LG2 | 3853742 | 3853692 | 3853792 |
| Marker33681 | LG2 | 3916320 | 3916270 | 3916370 |
| Marker33683 | LG2 | 3917659 | 3917609 | 3917709 |
| Marker33687 | LG2 | 3931704 | 3931654 | 3931754 |
| Marker33712 | LG2 | 3944672 | 3944622 | 3944722 |
| Marker33717 | LG2 | 3954392 | 3954342 | 3954442 |
| Marker33722 | LG2 | 3961459 | 3961409 | 3961509 |
| Marker33726 | LG2 | 3967035 | 3966985 | 3967085 |
| Marker33669 | LG2 | 4039375 | 4039325 | 4039425 |
| Marker33647 | LG2 | 4062025 | 4061975 | 4062075 |
| Marker33602 | LG2 | 4114362 | 4114312 | 4114412 |
| Marker34365 | LG2 | 4185789 | 4185739 | 4185839 |
| Marker34327 | LG2 | 4236749 | 4236699 | 4236799 |
| Marker34283 | LG2 | 4277306 | 4277256 | 4277356 |
| Marker34279 | LG2 | 4281681 | 4281631 | 4281731 |
| Marker86206 | LG2 | 4326520 | 4326470 | 4326570 |
| Marker34254 | LG2 | 4340009 | 4339959 | 4340059 |
| Marker34219 | LG2 | 4392648 | 4392598 | 4392698 |
| Marker34207 | LG2 | 4414390 | 4414340 | 4414440 |
| Marker34057 | LG2 | 4633117 | 4633067 | 4633167 |
| Marker34033 | LG2 | 4662046 | 4661996 | 4662096 |
| Marker34028 | LG2 | 4664548 | 4664498 | 4664598 |
| Marker34002 | LG2 | 4699553 | 4699503 | 4699603 |
| Marker33991 | LG2 | 4708218 | 4708168 | 4708268 |
| Marker33968 | LG2 | 4724810 | 4724760 | 4724860 |
| Marker33958 | LG2 | 4727499 | 4727449 | 4727549 |
| Marker33955 | LG2 | 4755180 | 4755130 | 4755230 |
| Marker33943 | LG2 | 4784472 | 4784422 | 4784522 |
| Marker33921 | LG2 | 4829180 | 4829130 | 4829230 |
| Marker33917 | LG2 | 4834321 | 4834271 | 4834371 |
| Marker33892 | LG2 | 4857730 | 4857680 | 4857780 |
| Marker33884 | LG2 | 4881478 | 4881428 | 4881528 |
| Marker33883 | LG2 | 4882232 | 4882182 | 4882282 |
| Marker33874 | LG2 | 4901358 | 4901308 | 4901408 |
| Marker33866 | LG2 | 4912374 | 4912324 | 4912424 |
| Marker33882 | LG2 | 4914804 | 4914754 | 4914854 |
| Marker33881 | LG2 | 4915081 | 4915031 | 4915131 |
| Marker33853 | LG2 | 4933875 | 4933825 | 4933925 |
| Marker33841 | LG2 | 4961832 | 4961782 | 4961882 |
| Marker33822 | LG2 | 5002467 | 5002417 | 5002517 |
| Marker33779 | LG2 | 5020402 | 5020352 | 5020452 |
| Marker33774 | LG2 | 5027636 | 5027586 | 5027686 |
| Marker33773 | LG2 | 5028830 | 5028780 | 5028880 |
| Marker33765 | LG2 | 5047418 | 5047368 | 5047468 |
| Marker33737 | LG2 | 5094932 | 5094882 | 5094982 |
| Marker33733 | LG2 | 5105094 | 5105044 | 5105144 |
| Marker34123 | LG2 | 5162278 | 5162228 | 5162328 |
| Marker34105 | LG2 | 5200295 | 5200245 | 5200345 |
| Marker34090 | LG2 | 5219624 | 5219574 | 5219674 |
| Marker33538 | LG2 | 5290567 | 5290517 | 5290617 |
| Marker33499 | LG2 | 5381083 | 5381033 | 5381133 |
| Marker33497 | LG2 | 5385554 | 5385504 | 5385604 |
| Marker34145 | LG2 | 5571557 | 5571507 | 5571607 |
| Marker33353 | LG2 | 5737153 | 5737103 | 5737203 |
| Marker33362 | LG2 | 5759627 | 5759577 | 5759677 |
| Marker33363 | LG2 | 5762136 | 5762086 | 5762186 |
| Marker33411 | LG2 | 5973974 | 5973924 | 5974024 |
| Marker33423 | LG2 | 5989327 | 5989277 | 5989377 |
| Marker33433 | LG2 | 6010271 | 6010221 | 6010321 |
| Marker33442 | LG2 | 6036420 | 6036370 | 6036470 |
| Marker33448 | LG2 | 6041886 | 6041836 | 6041936 |
| Marker33159 | LG2 | 6067283 | 6067233 | 6067333 |
| Marker33171 | LG2 | 6113103 | 6113053 | 6113153 |
| Marker33220 | LG2 | 6217442 | 6217392 | 6217492 |
| Marker33222 | LG2 | 6243038 | 6242988 | 6243088 |
| Marker33225 | LG2 | 6249000 | 6248950 | 6249050 |
| Marker32902 | LG2 | 6327238 | 6327188 | 6327288 |
| Marker35795 | LG2 | 6345497 | 6345447 | 6345547 |
| Marker32923 | LG2 | 6353273 | 6353223 | 6353323 |
| Marker32926 | LG2 | 6387334 | 6387284 | 6387384 |
| Marker32930 | LG2 | 6400431 | 6400381 | 6400481 |
| Marker32939 | LG2 | 6437854 | 6437804 | 6437904 |
| Marker33297 | LG2 | 6548264 | 6548214 | 6548314 |
| Marker33284 | LG2 | 6596284 | 6596234 | 6596334 |
| Marker32962 | LG2 | 6684654 | 6684604 | 6684704 |
| Marker32997 | LG2 | 6730024 | 6729974 | 6730074 |
| Marker33066 | LG2 | 6837538 | 6837488 | 6837588 |
| Marker33075 | LG2 | 6839324 | 6839274 | 6839374 |
| Marker33077 | LG2 | 6839402 | 6839352 | 6839452 |
| Marker8175 | LG2 | 7205136 | 7205086 | 7205186 |
| Marker33213 | LG2 | 7219972 | 7219922 | 7220022 |
| Marker8357 | LG2 | 7392592 | 7392542 | 7392642 |
| Marker8359 | LG2 | 7393063 | 7393013 | 7393113 |
| Marker8362 | LG2 | 7393308 | 7393258 | 7393358 |
| Marker8388 | LG2 | 7467953 | 7467903 | 7468003 |
| Marker8390 | LG2 | 7478995 | 7478945 | 7479045 |
| Marker8435 | LG2 | 7499373 | 7499323 | 7499423 |
| Marker30154 | LG2 | 7712226 | 7712176 | 7712276 |
| Marker30167 | LG2 | 7754848 | 7754798 | 7754898 |
| Marker30169 | LG2 | 7764155 | 7764105 | 7764205 |
| Marker30173 | LG2 | 7798088 | 7798038 | 7798138 |
| Marker30177 | LG2 | 7808692 | 7808642 | 7808742 |
| Marker30248 | LG2 | 7958338 | 7958288 | 7958388 |
| Marker30250 | LG2 | 7962788 | 7962738 | 7962838 |
| Marker30256 | LG2 | 7969051 | 7969001 | 7969101 |
| Marker30266 | LG2 | 7972820 | 7972770 | 7972870 |
| Marker30268 | LG2 | 7973381 | 7973331 | 7973431 |
| Marker30273 | LG2 | 7995981 | 7995931 | 7996031 |
| Marker30287 | LG2 | 8053192 | 8053142 | 8053242 |
| Marker30299 | LG2 | 8094285 | 8094235 | 8094335 |
| Marker30328 | LG2 | 8096092 | 8096042 | 8096142 |
| Marker32055 | LG2 | 8118550 | 8118500 | 8118600 |
| Marker32060 | LG2 | 8120759 | 8120709 | 8120809 |
| Marker32064 | LG2 | 8125830 | 8125780 | 8125880 |
| Marker32068 | LG2 | 8152320 | 8152270 | 8152370 |
| Marker32075 | LG2 | 8155770 | 8155720 | 8155820 |
| Marker32107 | LG2 | 8228452 | 8228402 | 8228502 |
| Marker32116 | LG2 | 8246140 | 8246090 | 8246190 |
| Marker32122 | LG2 | 8252926 | 8252876 | 8252976 |
| Marker32174 | LG2 | 8368840 | 8368790 | 8368890 |
| Marker32207 | LG2 | 8397035 | 8396985 | 8397085 |
| Marker32210 | LG2 | 8406022 | 8405972 | 8406072 |
| Marker32220 | LG2 | 8413917 | 8413867 | 8413967 |
| Marker32239 | LG2 | 8433282 | 8433232 | 8433332 |
| Marker32253 | LG2 | 8440620 | 8440570 | 8440670 |
| Marker32266 | LG2 | 8445670 | 8445620 | 8445720 |
| Marker32269 | LG2 | 8451846 | 8451796 | 8451896 |
| Marker32272 | LG2 | 8455642 | 8455592 | 8455692 |
| Marker32278 | LG2 | 8464978 | 8464928 | 8465028 |
| Marker32282 | LG2 | 8469183 | 8469133 | 8469233 |
| Marker32286 | LG2 | 8500178 | 8500128 | 8500228 |
| Marker32309 | LG2 | 8526751 | 8526701 | 8526801 |
| Marker32325 | LG2 | 8536088 | 8536038 | 8536138 |
| Marker32893 | LG2 | 8624083 | 8624033 | 8624133 |
| Marker32892 | LG2 | 8624278 | 8624228 | 8624328 |
| Marker32880 | LG2 | 8651362 | 8651312 | 8651412 |
| Marker32873 | LG2 | 8655666 | 8655616 | 8655716 |
| Marker32834 | LG2 | 8711016 | 8710966 | 8711066 |
| Marker32830 | LG2 | 8715356 | 8715306 | 8715406 |
| Marker32829 | LG2 | 8716409 | 8716359 | 8716459 |
| Marker32824 | LG2 | 8739632 | 8739582 | 8739682 |
| Marker32816 | LG2 | 8749756 | 8749706 | 8749806 |
| Marker32811 | LG2 | 8781036 | 8780986 | 8781086 |
| Marker32797 | LG2 | 8790476 | 8790426 | 8790526 |
| Marker32789 | LG2 | 8808175 | 8808125 | 8808225 |
| Marker32770 | LG2 | 8842265 | 8842215 | 8842315 |
| Marker32745 | LG2 | 8903049 | 8902999 | 8903099 |
| Marker32741 | LG2 | 8905704 | 8905654 | 8905754 |
| Marker32709 | LG2 | 8954006 | 8953956 | 8954056 |
| Marker32703 | LG2 | 8967997 | 8967947 | 8968047 |
| Marker32047 | LG2 | 8998929 | 8998879 | 8998979 |
| Marker32035 | LG2 | 9024240 | 9024190 | 9024290 |
| Marker32010 | LG2 | 9053246 | 9053196 | 9053296 |
| Marker32009 | LG2 | 9053500 | 9053450 | 9053550 |
| Marker31906 | LG2 | 9129780 | 9129730 | 9129830 |
| Marker31903 | LG2 | 9137016 | 9136966 | 9137066 |
| Marker31899 | LG2 | 9140579 | 9140529 | 9140629 |
| Marker31887 | LG2 | 9145698 | 9145648 | 9145748 |
| Marker31886 | LG2 | 9150461 | 9150411 | 9150511 |
| Marker31876 | LG2 | 9182513 | 9182463 | 9182563 |
| Marker31861 | LG2 | 9196015 | 9195965 | 9196065 |
| Marker31856 | LG2 | 9216026 | 9215976 | 9216076 |
| Marker31854 | LG2 | 9231651 | 9231601 | 9231701 |
| Marker31826 | LG2 | 9256332 | 9256282 | 9256382 |
| Marker31799 | LG2 | 9279707 | 9279657 | 9279757 |
| Marker31781 | LG2 | 9306543 | 9306493 | 9306593 |
| Marker31742 | LG2 | 9333683 | 9333633 | 9333733 |
| Marker31728 | LG2 | 9352926 | 9352876 | 9352976 |
| Marker31723 | LG2 | 9368158 | 9368108 | 9368208 |
| Marker31717 | LG2 | 9383987 | 9383937 | 9384037 |
| Marker31704 | LG2 | 9412171 | 9412121 | 9412221 |
| Marker31701 | LG2 | 9427359 | 9427309 | 9427409 |
| Marker31698 | LG2 | 9427602 | 9427552 | 9427652 |
| Marker31697 | LG2 | 9429553 | 9429503 | 9429603 |
| Marker31638 | LG2 | 9720253 | 9720203 | 9720303 |
| Marker31637 | LG2 | 9720256 | 9720206 | 9720306 |
| Marker28867 | LG2 | 10047835 | 10047785 | 10047885 |
| Marker28866 | LG2 | 10047898 | 10047848 | 10047948 |
| Marker28806 | LG2 | 10092776 | 10092726 | 10092826 |
| Marker120567 | LG2 | 10272428 | 10272378 | 10272478 |
| Marker28543 | LG2 | 10285032 | 10284982 | 10285082 |
| Marker28545 | LG2 | 10289717 | 10289667 | 10289767 |
| Marker28573 | LG2 | 10325331 | 10325281 | 10325381 |
| Marker28574 | LG2 | 10330138 | 10330088 | 10330188 |
| Marker28582 | LG2 | 10342216 | 10342166 | 10342266 |
| Marker28583 | LG2 | 10342494 | 10342444 | 10342544 |
| Marker28584 | LG2 | 10343542 | 10343492 | 10343592 |
| Marker85738 | LG2 | 10504730 | 10504680 | 10504780 |
| Marker119073 | LG2 | 10573006 | 10572956 | 10573056 |
| Marker119072 | LG2 | 10573035 | 10572985 | 10573085 |
| Marker27686 | LG2 | 10599464 | 10599414 | 10599514 |
| Marker27690 | LG2 | 10600054 | 10600004 | 10600104 |
| Marker27708 | LG2 | 10619385 | 10619335 | 10619435 |
| Marker27727 | LG2 | 10634228 | 10634178 | 10634278 |
| Marker27747 | LG2 | 10656370 | 10656320 | 10656420 |
| Marker27607 | LG2 | 10706074 | 10706024 | 10706124 |
| Marker27552 | LG2 | 10766181 | 10766131 | 10766231 |
| Marker27549 | LG2 | 10766267 | 10766217 | 10766317 |
| Marker27547 | LG2 | 10785168 | 10785118 | 10785218 |
| Marker27544 | LG2 | 10786516 | 10786466 | 10786566 |
| Marker27518 | LG2 | 10838346 | 10838296 | 10838396 |
| Marker27483 | LG2 | 10885423 | 10885373 | 10885473 |
| Marker27480 | LG2 | 10885638 | 10885588 | 10885688 |
| Marker27401 | LG2 | 10970959 | 10970909 | 10971009 |
| Marker85230 | LG2 | 11079287 | 11079237 | 11079337 |
| Marker85280 | LG2 | 11137871 | 11137821 | 11137921 |
| Marker85285 | LG2 | 11166692 | 11166642 | 11166742 |
| Marker85292 | LG2 | 11172198 | 11172148 | 11172248 |
| Marker6739 | LG2 | 11172224 | 11172174 | 11172274 |
| Marker85295 | LG2 | 11172405 | 11172355 | 11172455 |
| Marker26976 | LG2 | 11226189 | 11226139 | 11226239 |
| Marker27002 | LG2 | 11271421 | 11271371 | 11271471 |
| Marker27019 | LG2 | 11293094 | 11293044 | 11293144 |
| Marker47186 | LG2 | 11348856 | 11348806 | 11348906 |
| Marker27028 | LG2 | 11598465 | 11598415 | 11598515 |
| Marker27038 | LG2 | 11599265 | 11599215 | 11599315 |
| Marker27085 | LG2 | 11658668 | 11658618 | 11658718 |
| Marker27095 | LG2 | 11663588 | 11663538 | 11663638 |
| Marker27103 | LG2 | 11663798 | 11663748 | 11663848 |
| Marker27108 | LG2 | 11669989 | 11669939 | 11670039 |
| Marker27113 | LG2 | 11679991 | 11679941 | 11680041 |
| Marker27154 | LG2 | 11709067 | 11709017 | 11709117 |
| Marker27164 | LG2 | 11732020 | 11731970 | 11732070 |
| Marker27233 | LG2 | 11771080 | 11771030 | 11771130 |
| Marker27243 | LG2 | 11773522 | 11773472 | 11773572 |
| Marker27250 | LG2 | 11785572 | 11785522 | 11785622 |
| Marker27398 | LG2 | 11815041 | 11814991 | 11815091 |
| Marker27395 | LG2 | 11815998 | 11815948 | 11816048 |
| Marker27374 | LG2 | 11823565 | 11823515 | 11823615 |
| Marker27365 | LG2 | 11828210 | 11828160 | 11828260 |
| Marker27363 | LG2 | 11828395 | 11828345 | 11828445 |
| Marker27362 | LG2 | 11834541 | 11834491 | 11834591 |
| Marker27331 | LG2 | 11843313 | 11843263 | 11843363 |
| Marker26606 | LG2 | 12135428 | 12135378 | 12135478 |
| Marker26555 | LG2 | 12184796 | 12184746 | 12184846 |
| Marker26545 | LG2 | 12218053 | 12218003 | 12218103 |
| Marker26541 | LG2 | 12240132 | 12240082 | 12240182 |
| Marker26518 | LG2 | 12288562 | 12288512 | 12288612 |
| Marker26500 | LG2 | 12322165 | 12322115 | 12322215 |
| Marker26497 | LG2 | 12323280 | 12323230 | 12323330 |
| Marker26464 | LG2 | 12378191 | 12378141 | 12378241 |
| Marker26400 | LG2 | 12472142 | 12472092 | 12472192 |
| Marker26393 | LG2 | 12516485 | 12516435 | 12516535 |
| Marker26314 | LG2 | 12639150 | 12639100 | 12639200 |
| Marker26312 | LG2 | 12639311 | 12639261 | 12639361 |
| Marker26310 | LG2 | 12643288 | 12643238 | 12643338 |
| Marker26271 | LG2 | 12666049 | 12665999 | 12666099 |
| Marker26233 | LG2 | 12715942 | 12715892 | 12715992 |
| Marker120065 | LG2 | 12723839 | 12723789 | 12723889 |
| Marker26928 | LG2 | 12774845 | 12774795 | 12774895 |
| Marker27794 | LG2 | 12854075 | 12854025 | 12854125 |
| Marker27914 | LG2 | 12871871 | 12871821 | 12871921 |
| Marker27960 | LG2 | 12918424 | 12918374 | 12918474 |
| Marker27961 | LG2 | 12919415 | 12919365 | 12919465 |
| Marker27966 | LG2 | 12921505 | 12921455 | 12921555 |
| Marker27982 | LG2 | 12930801 | 12930751 | 12930851 |
| Marker27990 | LG2 | 12945018 | 12944968 | 12945068 |
| Marker28017 | LG2 | 12973592 | 12973542 | 12973642 |
| Marker28020 | LG2 | 12986918 | 12986868 | 12986968 |
| Marker28024 | LG2 | 13007855 | 13007805 | 13007905 |
| Marker28029 | LG2 | 13016533 | 13016483 | 13016583 |
| Marker28050 | LG2 | 13034585 | 13034535 | 13034635 |
| Marker112200 | LG2 | 13104241 | 13104191 | 13104291 |
| Marker28073 | LG2 | 13116511 | 13116461 | 13116561 |
| Marker28076 | LG2 | 13120435 | 13120385 | 13120485 |
| Marker28082 | LG2 | 13135162 | 13135112 | 13135212 |
| Marker28226 | LG2 | 13188081 | 13188031 | 13188131 |
| Marker8545 | LG2 | 13204015 | 13203965 | 13204065 |
| Marker28532 | LG2 | 13569749 | 13569699 | 13569799 |
| Marker28420 | LG2 | 13664936 | 13664886 | 13664986 |
| Marker28434 | LG2 | 13689535 | 13689485 | 13689585 |
| Marker28435 | LG2 | 13689569 | 13689519 | 13689619 |
| Marker85787 | LG2 | 13750770 | 13750720 | 13750820 |
| Marker28284 | LG2 | 13893502 | 13893452 | 13893552 |
| Marker28294 | LG2 | 13932460 | 13932410 | 13932510 |
| Marker28315 | LG2 | 13962674 | 13962624 | 13962724 |
| Marker28327 | LG2 | 13989454 | 13989404 | 13989504 |
| Marker28335 | LG2 | 13994376 | 13994326 | 13994426 |
| Marker28348 | LG2 | 14030825 | 14030775 | 14030875 |
| Marker29139 | LG2 | 14137809 | 14137759 | 14137859 |
| Marker29124 | LG2 | 14158965 | 14158915 | 14159015 |
| Marker29081 | LG2 | 14222231 | 14222181 | 14222281 |
| Marker29055 | LG2 | 14248591 | 14248541 | 14248641 |
| Marker29007 | LG2 | 14295407 | 14295357 | 14295457 |
| Marker29000 | LG2 | 14324895 | 14324845 | 14324945 |
| Marker1273 | LG2 | 14350590 | 14350540 | 14350640 |
| Marker73521 | LG2 | 14472226 | 14472176 | 14472276 |
| Marker115509 | LG2 | 14504457 | 14504407 | 14504507 |
| Marker29192 | LG2 | 14514310 | 14514260 | 14514360 |
| Marker29226 | LG2 | 14538254 | 14538204 | 14538304 |
| Marker29325 | LG2 | 14675383 | 14675333 | 14675433 |
| Marker29387 | LG2 | 14798212 | 14798162 | 14798262 |
| Marker29389 | LG2 | 14811690 | 14811640 | 14811740 |
| Marker29410 | LG2 | 14884913 | 14884863 | 14884963 |
| Marker29411 | LG2 | 14890908 | 14890858 | 14890958 |
| Marker29412 | LG2 | 14893323 | 14893273 | 14893373 |
| Marker29424 | LG2 | 14924986 | 14924936 | 14925036 |
| Marker29427 | LG2 | 14926450 | 14926400 | 14926500 |
| Marker29428 | LG2 | 14929821 | 14929771 | 14929871 |
| Marker29430 | LG2 | 14935483 | 14935433 | 14935533 |
| Marker29433 | LG2 | 14935796 | 14935746 | 14935846 |
| Marker29436 | LG2 | 14939216 | 14939166 | 14939266 |
| Marker29438 | LG2 | 14939250 | 14939200 | 14939300 |
| Marker29441 | LG2 | 14945623 | 14945573 | 14945673 |
| Marker29449 | LG2 | 14996494 | 14996444 | 14996544 |
| Marker29459 | LG2 | 15010947 | 15010897 | 15010997 |
| Marker29460 | LG2 | 15013613 | 15013563 | 15013663 |
| Marker29463 | LG2 | 15024029 | 15023979 | 15024079 |
| Marker29493 | LG2 | 15132474 | 15132424 | 15132524 |
| Marker29497 | LG2 | 15149377 | 15149327 | 15149427 |
| Marker29523 | LG2 | 15192652 | 15192602 | 15192702 |
| Marker112856 | LG2 | 15240488 | 15240438 | 15240538 |
| Marker112862 | LG2 | 15259378 | 15259328 | 15259428 |
| Marker112929 | LG2 | 15325877 | 15325827 | 15325927 |
| Marker112932 | LG2 | 15326107 | 15326057 | 15326157 |
| Marker112934 | LG2 | 15329235 | 15329185 | 15329285 |
| Marker49215 | LG2 | 15469689 | 15469639 | 15469739 |
| Marker29619 | LG2 | 15549273 | 15549223 | 15549323 |
| Marker29616 | LG2 | 15556473 | 15556423 | 15556523 |
| Marker29774 | LG2 | 16229012 | 16228962 | 16229062 |
| Marker29767 | LG2 | 16239549 | 16239499 | 16239599 |
| Marker29945 | LG2 | 16776550 | 16776500 | 16776600 |
| Marker29959 | LG2 | 16800536 | 16800486 | 16800586 |
| Marker29964 | LG2 | 16816967 | 16816917 | 16817017 |
| Marker29968 | LG2 | 16819598 | 16819548 | 16819648 |
| Marker29973 | LG2 | 16823290 | 16823240 | 16823340 |
| Marker29981 | LG2 | 16850496 | 16850446 | 16850546 |
| Marker29982 | LG2 | 16850734 | 16850684 | 16850784 |
| Marker30145 | LG2 | 17167039 | 17166989 | 17167089 |
| Marker31162 | LG2 | 17227007 | 17226957 | 17227057 |
| Marker31170 | LG2 | 17233492 | 17233442 | 17233542 |
| Marker31241 | LG2 | 17333892 | 17333842 | 17333942 |
| Marker31254 | LG2 | 17366095 | 17366045 | 17366145 |
| Marker31065 | LG2 | 17617509 | 17617459 | 17617559 |
| Marker31050 | LG2 | 17648453 | 17648403 | 17648503 |
| Marker31006 | LG2 | 17738803 | 17738753 | 17738853 |
| Marker30990 | LG2 | 17791743 | 17791693 | 17791793 |
| Marker30973 | LG2 | 17808035 | 17807985 | 17808085 |
| Marker30962 | LG2 | 17815381 | 17815331 | 17815431 |
| Marker30959 | LG2 | 17830236 | 17830186 | 17830286 |
| Marker30945 | LG2 | 17870654 | 17870604 | 17870704 |
| Marker30939 | LG2 | 17871864 | 17871814 | 17871914 |
| Marker30935 | LG2 | 17872949 | 17872899 | 17872999 |
| Marker30931 | LG2 | 17877644 | 17877594 | 17877694 |
| Marker30909 | LG2 | 17909603 | 17909553 | 17909653 |
| Marker30886 | LG2 | 17940724 | 17940674 | 17940774 |
| Marker30824 | LG2 | 17969038 | 17968988 | 17969088 |
| Marker28707 | LG2 | 18005579 | 18005529 | 18005629 |
| Marker30808 | LG2 | 18034158 | 18034108 | 18034208 |
| Marker30804 | LG2 | 18039606 | 18039556 | 18039656 |
| Marker30790 | LG2 | 18045014 | 18044964 | 18045064 |
| Marker30774 | LG2 | 18066263 | 18066213 | 18066313 |
| Marker30773 | LG2 | 18068085 | 18068035 | 18068135 |
| Marker30753 | LG2 | 18133571 | 18133521 | 18133621 |
| Marker30752 | LG2 | 18133755 | 18133705 | 18133805 |
| Marker30749 | LG2 | 18135502 | 18135452 | 18135552 |
| Marker30744 | LG2 | 18147178 | 18147128 | 18147228 |
| Marker30735 | LG2 | 18162430 | 18162380 | 18162480 |
| Marker30728 | LG2 | 18224902 | 18224852 | 18224952 |
| Marker30711 | LG2 | 18254949 | 18254899 | 18254999 |
| Marker30710 | LG2 | 18255180 | 18255130 | 18255230 |
| Marker30694 | LG2 | 18296160 | 18296110 | 18296210 |
| Marker30678 | LG2 | 18321131 | 18321081 | 18321181 |
| Marker30672 | LG2 | 18327234 | 18327184 | 18327284 |
| Marker30669 | LG2 | 18331202 | 18331152 | 18331252 |
| Marker30666 | LG2 | 18337878 | 18337828 | 18337928 |
| Marker30612 | LG2 | 18406365 | 18406315 | 18406415 |
| Marker30609 | LG2 | 18408861 | 18408811 | 18408911 |
| Marker30589 | LG2 | 18436171 | 18436121 | 18436221 |
| Marker30580 | LG2 | 18460896 | 18460846 | 18460946 |
| Marker30511 | LG2 | 18531778 | 18531728 | 18531828 |
| Marker30510 | LG2 | 18536185 | 18536135 | 18536235 |
| Marker30508 | LG2 | 18536925 | 18536875 | 18536975 |
| Marker31124 | LG2 | 18553442 | 18553392 | 18553492 |
| Marker30451 | LG2 | 18627879 | 18627829 | 18627929 |
| Marker30432 | LG2 | 18657295 | 18657245 | 18657345 |
| Marker30409 | LG2 | 18684818 | 18684768 | 18684868 |
| Marker30399 | LG2 | 18711904 | 18711854 | 18711954 |
| Marker30386 | LG2 | 18737020 | 18736970 | 18737070 |
| Marker30356 | LG2 | 18757417 | 18757367 | 18757467 |
| Marker30222 | LG2 | 18768386 | 18768336 | 18768436 |
| Marker30214 | LG2 | 18778837 | 18778787 | 18778887 |
| Marker30226 | LG2 | 18806634 | 18806584 | 18806684 |
| Marker30228 | LG2 | 18806791 | 18806741 | 18806841 |
| Marker30346 | LG2 | 18837827 | 18837777 | 18837877 |
| Marker30337 | LG2 | 18847363 | 18847313 | 18847413 |
| Marker30336 | LG2 | 18859186 | 18859136 | 18859236 |
| Marker30334 | LG2 | 18859434 | 18859384 | 18859484 |
| Marker41839 | LG2 | 18868894 | 18868844 | 18868944 |
| Marker41840 | LG2 | 18874598 | 18874548 | 18874648 |
| Marker41852 | LG2 | 18901049 | 18900999 | 18901099 |
| Marker41859 | LG2 | 18913726 | 18913676 | 18913776 |
| Marker41894 | LG2 | 18940352 | 18940302 | 18940402 |
| Marker41903 | LG2 | 18965335 | 18965285 | 18965385 |
| Marker26878 | LG2 | 19165864 | 19165814 | 19165914 |
| Marker26868 | LG2 | 19179450 | 19179400 | 19179500 |
| Marker26794 | LG2 | 19277932 | 19277882 | 19277982 |
| Marker26793 | LG2 | 19278022 | 19277972 | 19278072 |
| Marker26791 | LG2 | 19293085 | 19293035 | 19293135 |
| Marker26790 | LG2 | 19293493 | 19293443 | 19293543 |
| Marker26789 | LG2 | 19293717 | 19293667 | 19293767 |
| Marker26765 | LG2 | 19344407 | 19344357 | 19344457 |
| Marker26764 | LG2 | 19347086 | 19347036 | 19347136 |
| Marker26754 | LG2 | 19366510 | 19366460 | 19366560 |
| Marker26733 | LG2 | 19388766 | 19388716 | 19388816 |
| Marker26731 | LG2 | 19389667 | 19389617 | 19389717 |
| Marker119289 | LG2 | 19419573 | 19419523 | 19419623 |
| Marker26691 | LG2 | 19443953 | 19443903 | 19444003 |
| Marker26688 | LG2 | 19450043 | 19449993 | 19450093 |
| Marker26686 | LG2 | 19450306 | 19450256 | 19450356 |
| Marker26217 | LG2 | 19490631 | 19490581 | 19490681 |
| Marker26207 | LG2 | 19494620 | 19494570 | 19494670 |
| Marker25935 | LG2 | 19632053 | 19632003 | 19632103 |
| Marker25944 | LG2 | 19642503 | 19642453 | 19642553 |
| Marker25958 | LG2 | 19683273 | 19683223 | 19683323 |
| Marker25965 | LG2 | 19689689 | 19689639 | 19689739 |
| Marker25975 | LG2 | 19698726 | 19698676 | 19698776 |
| Marker25999 | LG2 | 19761765 | 19761715 | 19761815 |
| Marker26002 | LG2 | 19764445 | 19764395 | 19764495 |
| Marker26020 | LG2 | 19791090 | 19791040 | 19791140 |
| Marker26022 | LG2 | 19791303 | 19791253 | 19791353 |
| Marker26036 | LG2 | 19826205 | 19826155 | 19826255 |
| Marker26050 | LG2 | 19839923 | 19839873 | 19839973 |
| Marker26053 | LG2 | 19841621 | 19841571 | 19841671 |
| Marker26128 | LG2 | 19893690 | 19893640 | 19893740 |
| Marker26135 | LG2 | 19910948 | 19910898 | 19910998 |
| Marker26164 | LG2 | 19989098 | 19989048 | 19989148 |
| Marker26175 | LG2 | 20012085 | 20012035 | 20012135 |
| Marker25861 | LG2 | 20147670 | 20147620 | 20147720 |
| Marker25856 | LG2 | 20147892 | 20147842 | 20147942 |
| Marker25834 | LG2 | 20164325 | 20164275 | 20164375 |
| Marker25832 | LG2 | 20170177 | 20170127 | 20170227 |
| Marker25643 | LG2 | 20189160 | 20189110 | 20189210 |
| Marker25612 | LG2 | 20222149 | 20222099 | 20222199 |
| Marker25609 | LG2 | 20335973 | 20335923 | 20336023 |
| Marker25551 | LG2 | 20424808 | 20424758 | 20424858 |
| Marker25524 | LG2 | 20494770 | 20494720 | 20494820 |
| Marker25523 | LG2 | 20496487 | 20496437 | 20496537 |
| Marker25520 | LG2 | 20501698 | 20501648 | 20501748 |
| Marker25515 | LG2 | 20512276 | 20512226 | 20512326 |
| Marker25485 | LG2 | 20565572 | 20565522 | 20565622 |
| Marker25472 | LG2 | 20579470 | 20579420 | 20579520 |
| Marker25470 | LG2 | 20579970 | 20579920 | 20580020 |
| Marker25463 | LG2 | 20618284 | 20618234 | 20618334 |
| Marker25462 | LG2 | 20628066 | 20628016 | 20628116 |
| Marker25414 | LG2 | 20740643 | 20740593 | 20740693 |
| Marker25355 | LG2 | 20967778 | 20967728 | 20967828 |
| Marker25336 | LG2 | 21116198 | 21116148 | 21116248 |
| Marker25297 | LG2 | 21157722 | 21157672 | 21157772 |
| Marker25281 | LG2 | 21171300 | 21171250 | 21171350 |
| Marker25260 | LG2 | 21225589 | 21225539 | 21225639 |
| Marker25252 | LG2 | 21232204 | 21232154 | 21232254 |
| Marker25241 | LG2 | 21240674 | 21240624 | 21240724 |
| Marker25212 | LG2 | 21281983 | 21281933 | 21282033 |
| Marker25206 | LG2 | 21302059 | 21302009 | 21302109 |
| Marker25200 | LG2 | 21343381 | 21343331 | 21343431 |
| Marker25196 | LG2 | 21355006 | 21354956 | 21355056 |
| Marker25195 | LG2 | 21355547 | 21355497 | 21355597 |
| Marker25194 | LG2 | 21360952 | 21360902 | 21361002 |
| Marker25192 | LG2 | 21371243 | 21371193 | 21371293 |
| Marker25176 | LG2 | 21378020 | 21377970 | 21378070 |
| Marker25161 | LG2 | 21424955 | 21424905 | 21425005 |
| Marker25148 | LG2 | 21438138 | 21438088 | 21438188 |
| Marker25139 | LG2 | 21470610 | 21470560 | 21470660 |
| Marker25138 | LG2 | 21474119 | 21474069 | 21474169 |
| Marker25126 | LG2 | 21484268 | 21484218 | 21484318 |
| Marker25125 | LG2 | 21490878 | 21490828 | 21490928 |
| Marker25086 | LG2 | 21554201 | 21554151 | 21554251 |
| Marker25082 | LG2 | 21562238 | 21562188 | 21562288 |
| Marker25076 | LG2 | 21568396 | 21568346 | 21568446 |
| Marker25058 | LG2 | 21590726 | 21590676 | 21590776 |
| Marker25047 | LG2 | 21612774 | 21612724 | 21612824 |
| Marker25046 | LG2 | 21613043 | 21612993 | 21613093 |
| Marker25042 | LG2 | 21628476 | 21628426 | 21628526 |
| Marker25032 | LG2 | 21647442 | 21647392 | 21647492 |
| Marker31189 | LG2 | 21664178 | 21664128 | 21664228 |
| Marker24983 | LG2 | 21718734 | 21718684 | 21718784 |
| Marker24978 | LG2 | 21724949 | 21724899 | 21724999 |
| Marker119250 | LG2 | 21759258 | 21759208 | 21759308 |
| Marker119251 | LG2 | 21763448 | 21763398 | 21763498 |
| Marker24969 | LG2 | 21772720 | 21772670 | 21772770 |
| Marker24959 | LG2 | 21797391 | 21797341 | 21797441 |
| Marker24956 | LG2 | 21800222 | 21800172 | 21800272 |
| Marker24930 | LG2 | 21884723 | 21884673 | 21884773 |
| Marker24918 | LG2 | 21905605 | 21905555 | 21905655 |
| Marker24905 | LG2 | 21923898 | 21923848 | 21923948 |
| Marker24854 | LG2 | 22022586 | 22022536 | 22022636 |
| Marker24846 | LG2 | 22027562 | 22027512 | 22027612 |
| Marker24835 | LG2 | 22049068 | 22049018 | 22049118 |
| Marker24791 | LG2 | 22173991 | 22173941 | 22174041 |
| Marker24773 | LG2 | 22232217 | 22232167 | 22232267 |
| Marker24766 | LG2 | 22249931 | 22249881 | 22249981 |
| Marker24738 | LG2 | 22289718 | 22289668 | 22289768 |
| Marker24737 | LG2 | 22294472 | 22294422 | 22294522 |
| Marker24721 | LG2 | 22306368 | 22306318 | 22306418 |
| Marker24691 | LG2 | 22343222 | 22343172 | 22343272 |
| Marker117779 | LG2 | 22367018 | 22366968 | 22367068 |
| Marker117786 | LG2 | 22369434 | 22369384 | 22369484 |
| Marker117794 | LG2 | 22383999 | 22383949 | 22384049 |
| Marker117796 | LG2 | 22384184 | 22384134 | 22384234 |
| Marker117827 | LG2 | 22435413 | 22435363 | 22435463 |
| Marker119190 | LG2 | 22472781 | 22472731 | 22472831 |
| Marker117550 | LG2 | 22492383 | 22492333 | 22492433 |
| Marker116085 | LG2 | 22633247 | 22633197 | 22633297 |
| Marker23723 | LG2 | 22670968 | 22670918 | 22671018 |
| Marker23740 | LG2 | 22689178 | 22689128 | 22689228 |
| Marker23741 | LG2 | 22690053 | 22690003 | 22690103 |
| Marker23742 | LG2 | 22692153 | 22692103 | 22692203 |
| Marker23744 | LG2 | 22697188 | 22697138 | 22697238 |
| Marker23753 | LG2 | 22707924 | 22707874 | 22707974 |
| Marker23759 | LG2 | 22717299 | 22717249 | 22717349 |
| Marker23760 | LG2 | 22738143 | 22738093 | 22738193 |
| Marker23775 | LG2 | 22779620 | 22779570 | 22779670 |
| Marker23776 | LG2 | 22779780 | 22779730 | 22779830 |
| Marker24668 | LG2 | 22832977 | 22832927 | 22833027 |
| Marker97660 | LG2 | 22845551 | 22845501 | 22845601 |
| Marker24654 | LG2 | 22867366 | 22867316 | 22867416 |
| Marker24640 | LG2 | 22909879 | 22909829 | 22909929 |
| Marker24634 | LG2 | 22922345 | 22922295 | 22922395 |
| Marker24622 | LG2 | 22939473 | 22939423 | 22939523 |
| Marker24608 | LG2 | 22954640 | 22954590 | 22954690 |
| Marker24597 | LG2 | 22963777 | 22963727 | 22963827 |
| Marker24511 | LG2 | 23019260 | 23019210 | 23019310 |
| Marker24505 | LG2 | 23048176 | 23048126 | 23048226 |
| Marker24460 | LG2 | 23110167 | 23110117 | 23110217 |
| Marker24276 | LG2 | 23154980 | 23154930 | 23155030 |
| Marker24421 | LG2 | 23203080 | 23203030 | 23203130 |
| Marker24415 | LG2 | 23206757 | 23206707 | 23206807 |
| Marker24384 | LG2 | 23219711 | 23219661 | 23219761 |
| Marker24291 | LG2 | 23299153 | 23299103 | 23299203 |
| Marker24226 | LG2 | 23415838 | 23415788 | 23415888 |
| Marker24221 | LG2 | 23441690 | 23441640 | 23441740 |
| Marker24198 | LG2 | 23475132 | 23475082 | 23475182 |
| Marker24192 | LG2 | 23494966 | 23494916 | 23495016 |
| Marker24153 | LG2 | 23567434 | 23567384 | 23567484 |
| Marker24152 | LG2 | 23574405 | 23574355 | 23574455 |
| Marker24120 | LG2 | 23616553 | 23616503 | 23616603 |
| Marker110428 | LG2 | 23848599 | 23848549 | 23848649 |
| Marker110427 | LG2 | 23848804 | 23848754 | 23848854 |
| Marker110419 | LG2 | 23862405 | 23862355 | 23862455 |
| Marker110396 | LG2 | 23900152 | 23900102 | 23900202 |
| Marker110390 | LG2 | 23908915 | 23908865 | 23908965 |
| Marker110318 | LG2 | 23993187 | 23993137 | 23993237 |
| Marker110314 | LG2 | 24003194 | 24003144 | 24003244 |
| Marker110313 | LG2 | 24004773 | 24004723 | 24004823 |
| Marker110302 | LG2 | 24021990 | 24021940 | 24022040 |
| Marker110300 | LG2 | 24030360 | 24030310 | 24030410 |
| Marker110276 | LG2 | 24115492 | 24115442 | 24115542 |
| Marker110216 | LG2 | 24302907 | 24302857 | 24302957 |
| Marker24035 | LG2 | 24408211 | 24408161 | 24408261 |
| Marker23980 | LG2 | 24489611 | 24489561 | 24489661 |
| Marker23966 | LG2 | 24516218 | 24516168 | 24516268 |
| Marker23957 | LG2 | 24539054 | 24539004 | 24539104 |
| Marker23955 | LG2 | 24541093 | 24541043 | 24541143 |
| Marker23946 | LG2 | 24561755 | 24561705 | 24561805 |
| Marker23941 | LG2 | 24567699 | 24567649 | 24567749 |
| Marker23907 | LG2 | 24636263 | 24636213 | 24636313 |
| Marker23906 | LG2 | 24637365 | 24637315 | 24637415 |
| Marker108227 | LG2 | 24907960 | 24907910 | 24908010 |
| Marker108233 | LG2 | 24913516 | 24913466 | 24913566 |
| Marker108234 | LG2 | 24913565 | 24913515 | 24913615 |
| Marker24052 | LG2 | 25060763 | 25060713 | 25060813 |
| Marker108308 | LG2 | 25112735 | 25112685 | 25112785 |
| Marker108313 | LG2 | 25125518 | 25125468 | 25125568 |
| Marker108325 | LG2 | 25135027 | 25134977 | 25135077 |
| Marker108327 | LG2 | 25150850 | 25150800 | 25150900 |
| Marker108355 | LG2 | 25197187 | 25197137 | 25197237 |
| Marker108357 | LG2 | 25206594 | 25206544 | 25206644 |
| Marker108384 | LG2 | 25285542 | 25285492 | 25285592 |
| Marker122049 | LG2 | 25362876 | 25362826 | 25362926 |
| Marker108433 | LG2 | 25388442 | 25388392 | 25388492 |
| Marker108443 | LG2 | 25415464 | 25415414 | 25415514 |
| Marker108468 | LG2 | 25479578 | 25479528 | 25479628 |
| Marker108470 | LG2 | 25480786 | 25480736 | 25480836 |
| Marker108482 | LG2 | 25523776 | 25523726 | 25523826 |
| Marker108524 | LG2 | 25673829 | 25673779 | 25673879 |
| Marker108529 | LG2 | 25699737 | 25699687 | 25699787 |
| Marker108608 | LG2 | 25893316 | 25893266 | 25893366 |
| Marker108621 | LG2 | 25952244 | 25952194 | 25952294 |
| Marker108653 | LG2 | 26028750 | 26028700 | 26028800 |
| Marker108680 | LG2 | 26079875 | 26079825 | 26079925 |
| Marker108695 | LG2 | 26098322 | 26098272 | 26098372 |
| Marker108705 | LG2 | 26113617 | 26113567 | 26113667 |
| Marker108722 | LG2 | 26115144 | 26115094 | 26115194 |
| Marker108757 | LG2 | 26194800 | 26194750 | 26194850 |
| Marker108772 | LG2 | 26242807 | 26242757 | 26242857 |
| Marker108777 | LG2 | 26259191 | 26259141 | 26259241 |
| Marker108782 | LG2 | 26276443 | 26276393 | 26276493 |
| Marker108786 | LG2 | 26305814 | 26305764 | 26305864 |
| Marker23644 | LG2 | 26417044 | 26416994 | 26417094 |
| Marker68320 | LG2 | 26438176 | 26438126 | 26438226 |
| Marker23648 | LG2 | 26445424 | 26445374 | 26445474 |
| Marker23655 | LG2 | 26451456 | 26451406 | 26451506 |
| Marker23662 | LG2 | 26560731 | 26560681 | 26560781 |
| Marker23671 | LG2 | 26588083 | 26588033 | 26588133 |
| Marker22453 | LG2 | 26758409 | 26758359 | 26758459 |
| Marker22475 | LG2 | 26809117 | 26809067 | 26809167 |
| Marker22486 | LG2 | 26821968 | 26821918 | 26822018 |
| Marker22493 | LG2 | 26833852 | 26833802 | 26833902 |
| Marker22500 | LG2 | 26849318 | 26849268 | 26849368 |
| Marker22557 | LG2 | 26930157 | 26930107 | 26930207 |
| Marker22561 | LG2 | 26941245 | 26941195 | 26941295 |
| Marker22580 | LG2 | 27012341 | 27012291 | 27012391 |
| Marker22586 | LG2 | 27040529 | 27040479 | 27040579 |
| Marker22645 | LG2 | 27139457 | 27139407 | 27139507 |
| Marker22657 | LG2 | 27166465 | 27166415 | 27166515 |
| Marker22672 | LG2 | 27185666 | 27185616 | 27185716 |
| Marker22696 | LG2 | 27247469 | 27247419 | 27247519 |
| Marker22701 | LG2 | 27253285 | 27253235 | 27253335 |
| Marker22704 | LG2 | 27257586 | 27257536 | 27257636 |
| Marker22706 | LG2 | 27269380 | 27269330 | 27269430 |
| Marker22712 | LG2 | 27280856 | 27280806 | 27280906 |
| Marker22718 | LG2 | 27290845 | 27290795 | 27290895 |
| Marker22719 | LG2 | 27291102 | 27291052 | 27291152 |
| Marker22725 | LG2 | 27312524 | 27312474 | 27312574 |
| Marker22741 | LG2 | 27348615 | 27348565 | 27348665 |
| Marker22743 | LG2 | 27348859 | 27348809 | 27348909 |
| Marker22758 | LG2 | 27362940 | 27362890 | 27362990 |
| Marker22763 | LG2 | 27364176 | 27364126 | 27364226 |
| Marker22768 | LG2 | 27366796 | 27366746 | 27366846 |
| Marker22776 | LG2 | 27378329 | 27378279 | 27378379 |
| Marker22783 | LG2 | 27403262 | 27403212 | 27403312 |
| Marker22789 | LG2 | 27405673 | 27405623 | 27405723 |
| Marker22793 | LG2 | 27426173 | 27426123 | 27426223 |
| Marker22801 | LG2 | 27453994 | 27453944 | 27454044 |
| Marker22814 | LG2 | 27475142 | 27475092 | 27475192 |
| Marker22829 | LG2 | 27521371 | 27521321 | 27521421 |
| Marker22853 | LG2 | 27559738 | 27559688 | 27559788 |
| Marker22855 | LG2 | 27562728 | 27562678 | 27562778 |
| Marker22878 | LG2 | 27603245 | 27603195 | 27603295 |
| Marker22893 | LG2 | 27653469 | 27653419 | 27653519 |
| Marker22908 | LG2 | 27670568 | 27670518 | 27670618 |
| Marker22917 | LG2 | 27688621 | 27688571 | 27688671 |
| Marker22937 | LG2 | 27734971 | 27734921 | 27735021 |
| Marker22965 | LG2 | 27780476 | 27780426 | 27780526 |
| Marker22968 | LG2 | 27787067 | 27787017 | 27787117 |
| Marker22970 | LG2 | 27793292 | 27793242 | 27793342 |
| Marker22976 | LG2 | 27803391 | 27803341 | 27803441 |
| Marker22978 | LG2 | 27807022 | 27806972 | 27807072 |
| Marker22979 | LG2 | 27808896 | 27808846 | 27808946 |
| Marker22981 | LG2 | 27814680 | 27814630 | 27814730 |
| Marker22987 | LG2 | 27831326 | 27831276 | 27831376 |
| Marker22989 | LG2 | 27843625 | 27843575 | 27843675 |
| Marker23016 | LG2 | 27931017 | 27930967 | 27931067 |
| Marker23021 | LG2 | 27947945 | 27947895 | 27947995 |
| Marker23023 | LG2 | 27953569 | 27953519 | 27953619 |
| Marker23026 | LG2 | 27957409 | 27957359 | 27957459 |
| Marker23030 | LG2 | 27974229 | 27974179 | 27974279 |
| Marker23033 | LG2 | 27977125 | 27977075 | 27977175 |
| Marker23038 | LG2 | 27989875 | 27989825 | 27989925 |
| Marker23060 | LG2 | 28014329 | 28014279 | 28014379 |
| Marker23065 | LG2 | 28023388 | 28023338 | 28023438 |
| Marker23073 | LG2 | 28035402 | 28035352 | 28035452 |
| Marker23084 | LG2 | 28075932 | 28075882 | 28075982 |
| Marker23090 | LG2 | 28101323 | 28101273 | 28101373 |
| Marker23093 | LG2 | 28101525 | 28101475 | 28101575 |
| Marker23100 | LG2 | 28106303 | 28106253 | 28106353 |
| Marker23130 | LG2 | 28129529 | 28129479 | 28129579 |
| Marker23137 | LG2 | 28141193 | 28141143 | 28141243 |
| Marker23147 | LG2 | 28156444 | 28156394 | 28156494 |
| Marker23167 | LG2 | 28201123 | 28201073 | 28201173 |
| Marker23174 | LG2 | 28226015 | 28225965 | 28226065 |
| Marker23176 | LG2 | 28231165 | 28231115 | 28231215 |
| Marker23182 | LG2 | 28242288 | 28242238 | 28242338 |
| Marker23205 | LG2 | 28256280 | 28256230 | 28256330 |
| Marker23259 | LG2 | 28335485 | 28335435 | 28335535 |
| Marker23261 | LG2 | 28343211 | 28343161 | 28343261 |
| Marker23268 | LG2 | 28359548 | 28359498 | 28359598 |
| Marker23281 | LG2 | 28370425 | 28370375 | 28370475 |
| Marker23292 | LG2 | 28407647 | 28407597 | 28407697 |
| Marker23335 | LG2 | 28534634 | 28534584 | 28534684 |
| Marker23345 | LG2 | 28562547 | 28562497 | 28562597 |
| Marker23347 | LG2 | 28565559 | 28565509 | 28565609 |
| Marker23389 | LG2 | 28591867 | 28591817 | 28591917 |
| Marker23413 | LG2 | 28681160 | 28681110 | 28681210 |
| Marker23424 | LG2 | 28694715 | 28694665 | 28694765 |
| Marker23439 | LG2 | 28700221 | 28700171 | 28700271 |
| Marker23466 | LG2 | 28726741 | 28726691 | 28726791 |
| Marker23468 | LG2 | 28729441 | 28729391 | 28729491 |
| Marker23473 | LG2 | 28734405 | 28734355 | 28734455 |
| Marker23474 | LG2 | 28739465 | 28739415 | 28739515 |
| Marker23478 | LG2 | 28743362 | 28743312 | 28743412 |
| Marker23494 | LG2 | 28817342 | 28817292 | 28817392 |
| Marker23508 | LG2 | 28833462 | 28833412 | 28833512 |
| Marker23521 | LG2 | 28864644 | 28864594 | 28864694 |
| Marker23523 | LG2 | 28866305 | 28866255 | 28866355 |
| Marker23525 | LG2 | 28866894 | 28866844 | 28866944 |
| Marker23554 | LG2 | 28924056 | 28924006 | 28924106 |
| Marker23570 | LG2 | 28946028 | 28945978 | 28946078 |
| Marker23575 | LG2 | 28951650 | 28951600 | 28951700 |
| Marker23583 | LG2 | 28969263 | 28969213 | 28969313 |
| Marker23592 | LG2 | 28971075 | 28971025 | 28971125 |
| Marker23593 | LG2 | 28974834 | 28974784 | 28974884 |
| Marker23626 | LG2 | 29132080 | 29132030 | 29132130 |
| Marker23628 | LG2 | 29138824 | 29138774 | 29138874 |
| Marker23629 | LG2 | 29150029 | 29149979 | 29150079 |
| Marker22446 | LG2 | 29163686 | 29163636 | 29163736 |
| Marker22424 | LG2 | 29233762 | 29233712 | 29233812 |
| Marker22392 | LG2 | 29276143 | 29276093 | 29276193 |
| Marker22377 | LG2 | 29301826 | 29301776 | 29301876 |
| Marker22367 | LG2 | 29307802 | 29307752 | 29307852 |
| Marker22366 | LG2 | 29308454 | 29308404 | 29308504 |
| Marker22364 | LG2 | 29322727 | 29322677 | 29322777 |
| Marker277 | LG2 | 29366991 | 29366941 | 29367041 |
| Marker22346 | LG2 | 29373842 | 29373792 | 29373892 |
| Marker22324 | LG2 | 29380221 | 29380171 | 29380271 |
| Marker22297 | LG2 | 29415675 | 29415625 | 29415725 |
| Marker22296 | LG2 | 29418985 | 29418935 | 29419035 |
| Marker22293 | LG2 | 29430401 | 29430351 | 29430451 |
| Marker22268 | LG2 | 29463145 | 29463095 | 29463195 |
| Marker22260 | LG2 | 29480166 | 29480116 | 29480216 |
| Marker22259 | LG2 | 29483805 | 29483755 | 29483855 |
| Marker22252 | LG2 | 29494560 | 29494510 | 29494610 |
| Marker22251 | LG2 | 29501432 | 29501382 | 29501482 |
| Marker22247 | LG2 | 29539971 | 29539921 | 29540021 |
| Marker22214 | LG2 | 29632249 | 29632199 | 29632299 |
| Marker22212 | LG2 | 29638192 | 29638142 | 29638242 |
| Marker22211 | LG2 | 29640327 | 29640277 | 29640377 |
| Marker22210 | LG2 | 29640805 | 29640755 | 29640855 |
| Marker22207 | LG2 | 29646652 | 29646602 | 29646702 |
| Marker22184 | LG2 | 29719696 | 29719646 | 29719746 |
| Marker22177 | LG2 | 29779038 | 29778988 | 29779088 |
| Marker22174 | LG2 | 29780072 | 29780022 | 29780122 |
| Marker22118 | LG2 | 29971749 | 29971699 | 29971799 |
| Marker22116 | LG2 | 29980206 | 29980156 | 29980256 |
| Marker22102 | LG2 | 30008773 | 30008723 | 30008823 |
| Marker22081 | LG2 | 30030255 | 30030205 | 30030305 |
| Marker22079 | LG2 | 30030561 | 30030511 | 30030611 |
| Marker22052 | LG2 | 30063911 | 30063861 | 30063961 |
| Marker22051 | LG2 | 30069024 | 30068974 | 30069074 |
| Marker22042 | LG2 | 30103856 | 30103806 | 30103906 |
| Marker22041 | LG2 | 30107846 | 30107796 | 30107896 |
| Marker22021 | LG2 | 30152597 | 30152547 | 30152647 |
| Marker22005 | LG2 | 30195278 | 30195228 | 30195328 |
| Marker21985 | LG2 | 30233162 | 30233112 | 30233212 |
| Marker21982 | LG2 | 30239048 | 30238998 | 30239098 |
| Marker21972 | LG2 | 30267258 | 30267208 | 30267308 |
| Marker21964 | LG2 | 30273927 | 30273877 | 30273977 |
| Marker21955 | LG2 | 30282800 | 30282750 | 30282850 |
| Marker21949 | LG2 | 30284954 | 30284904 | 30285004 |
| Marker21943 | LG2 | 30306703 | 30306653 | 30306753 |
| Marker21932 | LG2 | 30312486 | 30312436 | 30312536 |
| Marker21929 | LG2 | 30313396 | 30313346 | 30313446 |
| Marker21926 | LG2 | 30326492 | 30326442 | 30326542 |
| Marker21919 | LG2 | 30331688 | 30331638 | 30331738 |
| Marker21917 | LG2 | 30335408 | 30335358 | 30335458 |
| Marker21912 | LG2 | 30354189 | 30354139 | 30354239 |
| Marker21908 | LG2 | 30357473 | 30357423 | 30357523 |
| Marker21837 | LG2 | 30434812 | 30434762 | 30434862 |
| Marker21808 | LG2 | 30508645 | 30508595 | 30508695 |
| Marker21806 | LG2 | 30508836 | 30508786 | 30508886 |
| Marker21805 | LG2 | 30509889 | 30509839 | 30509939 |
| Marker21787 | LG2 | 30542072 | 30542022 | 30542122 |
| Marker21785 | LG2 | 30542347 | 30542297 | 30542397 |
| Marker21783 | LG2 | 30547720 | 30547670 | 30547770 |
| Marker21702 | LG2 | 30702668 | 30702618 | 30702718 |
| Marker21680 | LG2 | 30765699 | 30765649 | 30765749 |
| Marker21575 | LG2 | 31099947 | 31099897 | 31099997 |
| Marker21570 | LG2 | 31113706 | 31113656 | 31113756 |
| Marker21554 | LG2 | 31133468 | 31133418 | 31133518 |
| Marker21543 | LG2 | 31143027 | 31142977 | 31143077 |
| Marker21542 | LG2 | 31143685 | 31143635 | 31143735 |
| Marker21541 | LG2 | 31143874 | 31143824 | 31143924 |
| Marker21508 | LG2 | 31181939 | 31181889 | 31181989 |
| Marker21501 | LG2 | 31193518 | 31193468 | 31193568 |
| Marker21499 | LG2 | 31204366 | 31204316 | 31204416 |
| Marker21488 | LG2 | 31235399 | 31235349 | 31235449 |
| Marker21479 | LG2 | 31242895 | 31242845 | 31242945 |
| Marker21471 | LG2 | 31254425 | 31254375 | 31254475 |
| Marker21459 | LG2 | 31271103 | 31271053 | 31271153 |
| Marker21456 | LG2 | 31272398 | 31272348 | 31272448 |
| Marker21444 | LG2 | 31282127 | 31282077 | 31282177 |
| Marker21438 | LG2 | 31302564 | 31302514 | 31302614 |
| Marker21434 | LG2 | 31306741 | 31306691 | 31306791 |
| Marker21426 | LG2 | 31337308 | 31337258 | 31337358 |
| Marker21421 | LG2 | 31347284 | 31347234 | 31347334 |
| Marker21409 | LG2 | 31361395 | 31361345 | 31361445 |
| Marker21389 | LG2 | 31387157 | 31387107 | 31387207 |
| Marker21388 | LG2 | 31390077 | 31390027 | 31390127 |
| Marker21386 | LG2 | 31391521 | 31391471 | 31391571 |
| Marker21381 | LG2 | 31409119 | 31409069 | 31409169 |
| Marker21367 | LG2 | 31431968 | 31431918 | 31432018 |
| Marker21356 | LG2 | 31448382 | 31448332 | 31448432 |
| Marker21351 | LG2 | 31455208 | 31455158 | 31455258 |
| Marker21333 | LG2 | 31479921 | 31479871 | 31479971 |
| Marker21329 | LG2 | 31483677 | 31483627 | 31483727 |
| Marker21328 | LG2 | 31484644 | 31484594 | 31484694 |
| Marker21312 | LG2 | 31489704 | 31489654 | 31489754 |
| Marker21308 | LG2 | 31491338 | 31491288 | 31491388 |
| Marker21306 | LG2 | 31503677 | 31503627 | 31503727 |
| Marker21297 | LG2 | 31527691 | 31527641 | 31527741 |
| Marker21281 | LG2 | 31550075 | 31550025 | 31550125 |
| Marker21236 | LG2 | 31655142 | 31655092 | 31655192 |
| Marker21220 | LG2 | 31682099 | 31682049 | 31682149 |
| Marker21219 | LG2 | 31692740 | 31692690 | 31692790 |
| Marker21209 | LG2 | 31726761 | 31726711 | 31726811 |
| Marker21208 | LG2 | 31727028 | 31726978 | 31727078 |
| Marker21195 | LG2 | 31744680 | 31744630 | 31744730 |
| Marker21181 | LG2 | 31777983 | 31777933 | 31778033 |
| Marker21174 | LG2 | 31783440 | 31783390 | 31783490 |
| Marker21151 | LG2 | 31837964 | 31837914 | 31838014 |
| Marker21139 | LG2 | 31862298 | 31862248 | 31862348 |
| Marker21134 | LG2 | 31870597 | 31870547 | 31870647 |
| Marker21133 | LG2 | 31870649 | 31870599 | 31870699 |
| Marker21132 | LG2 | 31873490 | 31873440 | 31873540 |
| Marker21126 | LG2 | 31877579 | 31877529 | 31877629 |
| Marker21108 | LG2 | 31934785 | 31934735 | 31934835 |
| Marker21107 | LG2 | 31954310 | 31954260 | 31954360 |
| Marker21094 | LG2 | 31972171 | 31972121 | 31972221 |
| Marker21089 | LG2 | 31988214 | 31988164 | 31988264 |
| Marker21076 | LG2 | 32027367 | 32027317 | 32027417 |
| Marker21062 | LG2 | 32058127 | 32058077 | 32058177 |
| Marker21052 | LG2 | 32078973 | 32078923 | 32079023 |
| Marker21031 | LG2 | 32105009 | 32104959 | 32105059 |
| Marker21018 | LG2 | 32119044 | 32118994 | 32119094 |
| Marker21013 | LG2 | 32122217 | 32122167 | 32122267 |
| Marker21008 | LG2 | 32123803 | 32123753 | 32123853 |
| Marker21004 | LG2 | 32137518 | 32137468 | 32137568 |
| Marker20997 | LG2 | 32141603 | 32141553 | 32141653 |
| Marker20993 | LG2 | 32151313 | 32151263 | 32151363 |
| Marker20990 | LG2 | 32155421 | 32155371 | 32155471 |
| Marker20960 | LG2 | 32202470 | 32202420 | 32202520 |
| Marker20955 | LG2 | 32203873 | 32203823 | 32203923 |
| Marker20952 | LG2 | 32208144 | 32208094 | 32208194 |
| Marker20951 | LG2 | 32209844 | 32209794 | 32209894 |
| Marker20948 | LG2 | 32213785 | 32213735 | 32213835 |
| Marker20945 | LG2 | 32215621 | 32215571 | 32215671 |
| Marker20931 | LG2 | 32234957 | 32234907 | 32235007 |
| Marker20903 | LG2 | 32301491 | 32301441 | 32301541 |
| Marker20902 | LG2 | 32302490 | 32302440 | 32302540 |
| Marker20897 | LG2 | 32313390 | 32313340 | 32313440 |
| Marker20885 | LG2 | 32315261 | 32315211 | 32315311 |
| Marker20879 | LG2 | 32332389 | 32332339 | 32332439 |
| Marker20878 | LG2 | 32334986 | 32334936 | 32335036 |
| Marker20858 | LG2 | 32367813 | 32367763 | 32367863 |
| Marker20824 | LG2 | 32433460 | 32433410 | 32433510 |
| Marker20816 | LG2 | 32462853 | 32462803 | 32462903 |
| Marker20804 | LG2 | 32481771 | 32481721 | 32481821 |
| Marker20803 | LG2 | 32482932 | 32482882 | 32482982 |
| Marker20782 | LG2 | 32497229 | 32497179 | 32497279 |
| Marker20780 | LG2 | 32499444 | 32499394 | 32499494 |
| Marker20776 | LG2 | 32507607 | 32507557 | 32507657 |
| Marker20773 | LG2 | 32524280 | 32524230 | 32524330 |
| Marker20741 | LG2 | 32617698 | 32617648 | 32617748 |
| Marker20715 | LG2 | 32639327 | 32639277 | 32639377 |
| Marker20707 | LG2 | 32658255 | 32658205 | 32658305 |
| Marker20700 | LG2 | 32672829 | 32672779 | 32672879 |
| Marker20692 | LG2 | 32679088 | 32679038 | 32679138 |
| Marker20678 | LG2 | 32719615 | 32719565 | 32719665 |
| Marker20676 | LG2 | 32720053 | 32720003 | 32720103 |
| Marker20639 | LG2 | 32784288 | 32784238 | 32784338 |
| Marker20635 | LG2 | 32800444 | 32800394 | 32800494 |
| Marker20634 | LG2 | 32813778 | 32813728 | 32813828 |
| Marker20613 | LG2 | 32844181 | 32844131 | 32844231 |
| Marker24409 | LG2 | 32857743 | 32857693 | 32857793 |
| Marker20557 | LG2 | 32917336 | 32917286 | 32917386 |
| Marker20547 | LG2 | 32920972 | 32920922 | 32921022 |
| Marker20534 | LG2 | 32933910 | 32933860 | 32933960 |
| Marker20524 | LG2 | 32951025 | 32950975 | 32951075 |
| Marker20466 | LG2 | 33031912 | 33031862 | 33031962 |
| Marker20452 | LG2 | 33040929 | 33040879 | 33040979 |
| Marker20447 | LG2 | 33042843 | 33042793 | 33042893 |
| Marker20430 | LG2 | 33054417 | 33054367 | 33054467 |
| Marker20394 | LG2 | 33116651 | 33116601 | 33116701 |
| Marker20389 | LG2 | 33128521 | 33128471 | 33128571 |
| Marker20379 | LG2 | 33145260 | 33145210 | 33145310 |
| Marker20344 | LG2 | 33181219 | 33181169 | 33181269 |
| Marker20339 | LG2 | 33183063 | 33183013 | 33183113 |
| Marker20334 | LG2 | 33189031 | 33188981 | 33189081 |
| Marker20317 | LG2 | 33204468 | 33204418 | 33204518 |
| Marker20282 | LG2 | 33251616 | 33251566 | 33251666 |
| Marker20278 | LG2 | 33256455 | 33256405 | 33256505 |
| Marker20206 | LG2 | 33401800 | 33401750 | 33401850 |
| Marker20201 | LG2 | 33410501 | 33410451 | 33410551 |
| Marker20186 | LG2 | 33419980 | 33419930 | 33420030 |
| Marker20184 | LG2 | 33426818 | 33426768 | 33426868 |
| Marker20140 | LG2 | 33477197 | 33477147 | 33477247 |
| Marker20138 | LG2 | 33477468 | 33477418 | 33477518 |
| Marker20135 | LG2 | 33479251 | 33479201 | 33479301 |
| Marker20120 | LG2 | 33491388 | 33491338 | 33491438 |
| Marker20110 | LG2 | 33496805 | 33496755 | 33496855 |
| Marker20099 | LG2 | 33523923 | 33523873 | 33523973 |
| Marker20095 | LG2 | 33527338 | 33527288 | 33527388 |
| Marker20094 | LG2 | 33530341 | 33530291 | 33530391 |
| Marker20090 | LG2 | 33534306 | 33534256 | 33534356 |
| Marker20082 | LG2 | 33558589 | 33558539 | 33558639 |
| Marker20070 | LG2 | 33586446 | 33586396 | 33586496 |
| Marker20053 | LG2 | 33624700 | 33624650 | 33624750 |
| Marker20045 | LG2 | 33630521 | 33630471 | 33630571 |
| Marker20022 | LG2 | 33640597 | 33640547 | 33640647 |
| Marker20018 | LG2 | 33649654 | 33649604 | 33649704 |
| Marker19990 | LG2 | 33670785 | 33670735 | 33670835 |
| Marker19969 | LG2 | 33708318 | 33708268 | 33708368 |
| Marker19967 | LG2 | 33718433 | 33718383 | 33718483 |
| Marker19943 | LG2 | 33771309 | 33771259 | 33771359 |
| Marker19941 | LG2 | 33773625 | 33773575 | 33773675 |
| Marker19554 | LG2 | 33835591 | 33835541 | 33835641 |
| Marker19556 | LG2 | 33835820 | 33835770 | 33835870 |
| Marker19574 | LG2 | 33869306 | 33869256 | 33869356 |
| Marker19591 | LG2 | 33876326 | 33876276 | 33876376 |
| Marker19598 | LG2 | 33892075 | 33892025 | 33892125 |
| Marker19604 | LG2 | 33899041 | 33898991 | 33899091 |
| Marker19615 | LG2 | 33961662 | 33961612 | 33961712 |
| Marker19616 | LG2 | 33961992 | 33961942 | 33962042 |
| Marker19621 | LG2 | 33973012 | 33972962 | 33973062 |
| Marker19658 | LG2 | 34007716 | 34007666 | 34007766 |
| Marker19666 | LG2 | 34019583 | 34019533 | 34019633 |
| Marker19672 | LG2 | 34020659 | 34020609 | 34020709 |
| Marker19676 | LG2 | 34030989 | 34030939 | 34031039 |
| Marker19687 | LG2 | 34061611 | 34061561 | 34061661 |
| Marker19692 | LG2 | 34063201 | 34063151 | 34063251 |
| Marker19700 | LG2 | 34078428 | 34078378 | 34078478 |
| Marker19717 | LG2 | 34100403 | 34100353 | 34100453 |
| Marker19722 | LG2 | 34108892 | 34108842 | 34108942 |
| Marker19736 | LG2 | 34119556 | 34119506 | 34119606 |
| Marker19744 | LG2 | 34145499 | 34145449 | 34145549 |
| Marker19765 | LG2 | 34166049 | 34165999 | 34166099 |
| Marker19789 | LG2 | 34221339 | 34221289 | 34221389 |
| Marker19791 | LG2 | 34221368 | 34221318 | 34221418 |
| Marker19837 | LG2 | 34334313 | 34334263 | 34334363 |
| Marker19838 | LG2 | 34344774 | 34344724 | 34344824 |
| Marker19841 | LG2 | 34349019 | 34348969 | 34349069 |
| Marker19857 | LG2 | 34408185 | 34408135 | 34408235 |
| Marker19861 | LG2 | 34420960 | 34420910 | 34421010 |
| Marker19875 | LG2 | 34434050 | 34434000 | 34434100 |
| Marker19879 | LG2 | 34454073 | 34454023 | 34454123 |
| Marker19885 | LG2 | 34456119 | 34456069 | 34456169 |
| Marker19889 | LG2 | 34462699 | 34462649 | 34462749 |
| Marker19894 | LG2 | 34465218 | 34465168 | 34465268 |
| Marker19896 | LG2 | 34465476 | 34465426 | 34465526 |
| Marker19900 | LG2 | 34469810 | 34469760 | 34469860 |
| Marker19909 | LG2 | 34489356 | 34489306 | 34489406 |
| Marker59481 | LG2 | 34546968 | 34546918 | 34547018 |
| Marker19460 | LG2 | 34560087 | 34560037 | 34560137 |
| Marker19481 | LG2 | 34627435 | 34627385 | 34627485 |
| Marker15793 | LG2 | 34734786 | 34734736 | 34734836 |
| Marker15792 | LG2 | 34735027 | 34734977 | 34735077 |
| Marker120358 | LG2 | 35000003 | 34999953 | 35000053 |
| Marker120364 | LG2 | 35000955 | 35000905 | 35001005 |
| Marker117268 | LG2 | 35055181 | 35055131 | 35055231 |
| Marker18340 | LG2 | 35088443 | 35088393 | 35088493 |
| Marker18380 | LG2 | 35123118 | 35123068 | 35123168 |
| Marker18384 | LG2 | 35136198 | 35136148 | 35136248 |
| Marker18418 | LG2 | 35160109 | 35160059 | 35160159 |
| Marker18437 | LG2 | 35172109 | 35172059 | 35172159 |
| Marker18443 | LG2 | 35188005 | 35187955 | 35188055 |
| Marker18452 | LG2 | 35198204 | 35198154 | 35198254 |
| Marker18457 | LG2 | 35211058 | 35211008 | 35211108 |
| Marker18459 | LG2 | 35226733 | 35226683 | 35226783 |
| Marker18462 | LG2 | 35227050 | 35227000 | 35227100 |
| Marker18499 | LG2 | 35257273 | 35257223 | 35257323 |
| Marker18515 | LG2 | 35279078 | 35279028 | 35279128 |
| Marker18521 | LG2 | 35324747 | 35324697 | 35324797 |
| Marker18567 | LG2 | 35390176 | 35390126 | 35390226 |
| Marker18569 | LG2 | 35390695 | 35390645 | 35390745 |
| Marker18587 | LG2 | 35421967 | 35421917 | 35422017 |
| Marker18609 | LG2 | 35567844 | 35567794 | 35567894 |
| Marker18661 | LG2 | 35614294 | 35614244 | 35614344 |
| Marker18662 | LG2 | 35614556 | 35614506 | 35614606 |
| Marker18695 | LG2 | 35665691 | 35665641 | 35665741 |
| Marker18707 | LG2 | 35691640 | 35691590 | 35691690 |
| Marker18716 | LG2 | 35705929 | 35705879 | 35705979 |
| Marker18739 | LG2 | 35731095 | 35731045 | 35731145 |
| Marker18758 | LG2 | 35754842 | 35754792 | 35754892 |
| Marker18784 | LG2 | 35854887 | 35854837 | 35854937 |
| Marker18785 | LG2 | 35865876 | 35865826 | 35865926 |
| Marker18789 | LG2 | 35867835 | 35867785 | 35867885 |
| Marker18791 | LG2 | 35890984 | 35890934 | 35891034 |
| Marker18806 | LG2 | 35965636 | 35965586 | 35965686 |
| Marker18846 | LG2 | 36045618 | 36045568 | 36045668 |
| Marker18860 | LG2 | 36074912 | 36074862 | 36074962 |
| Marker18871 | LG2 | 36096508 | 36096458 | 36096558 |
| Marker18873 | LG2 | 36098403 | 36098353 | 36098453 |
| Marker18909 | LG2 | 36129232 | 36129182 | 36129282 |
| Marker18910 | LG2 | 36129748 | 36129698 | 36129798 |
| Marker18915 | LG2 | 36154657 | 36154607 | 36154707 |
| Marker18918 | LG2 | 36172973 | 36172923 | 36173023 |
| Marker18937 | LG2 | 36229700 | 36229650 | 36229750 |
| Marker18949 | LG2 | 36254540 | 36254490 | 36254590 |
| Marker18951 | LG2 | 36259645 | 36259595 | 36259695 |
| Marker18952 | LG2 | 36263564 | 36263514 | 36263614 |
| Marker18991 | LG2 | 36295762 | 36295712 | 36295812 |
| Marker18995 | LG2 | 36301866 | 36301816 | 36301916 |
| Marker19006 | LG2 | 36311538 | 36311488 | 36311588 |
| Marker19023 | LG2 | 36351785 | 36351735 | 36351835 |
| Marker19038 | LG2 | 36370472 | 36370422 | 36370522 |
| Marker19042 | LG2 | 36371208 | 36371158 | 36371258 |
| Marker19052 | LG2 | 36384834 | 36384784 | 36384884 |
| Marker19055 | LG2 | 36385408 | 36385358 | 36385458 |
| Marker19056 | LG2 | 36393648 | 36393598 | 36393698 |
| Marker19061 | LG2 | 36405245 | 36405195 | 36405295 |
| Marker19066 | LG2 | 36415902 | 36415852 | 36415952 |
| Marker19076 | LG2 | 36425873 | 36425823 | 36425923 |
| Marker19081 | LG2 | 36436782 | 36436732 | 36436832 |
| Marker19084 | LG2 | 36438421 | 36438371 | 36438471 |
| Marker19089 | LG2 | 36441460 | 36441410 | 36441510 |
| Marker19091 | LG2 | 36443505 | 36443455 | 36443555 |
| Marker19093 | LG2 | 36447235 | 36447185 | 36447285 |
| Marker19099 | LG2 | 36458369 | 36458319 | 36458419 |
| Marker19380 | LG2 | 36527146 | 36527096 | 36527196 |
| Marker19364 | LG2 | 36534685 | 36534635 | 36534735 |
| Marker19352 | LG2 | 36551641 | 36551591 | 36551691 |
| Marker19345 | LG2 | 36563214 | 36563164 | 36563264 |
| Marker19341 | LG2 | 36584458 | 36584408 | 36584508 |
| Marker19338 | LG2 | 36584888 | 36584838 | 36584938 |
| Marker75263 | LG2 | 36637011 | 36636961 | 36637061 |
| Marker75268 | LG2 | 36637309 | 36637259 | 36637359 |
| Marker75275 | LG2 | 36640295 | 36640245 | 36640345 |
| Marker75286 | LG2 | 36643865 | 36643815 | 36643915 |
| Marker75330 | LG2 | 36694843 | 36694793 | 36694893 |
| Marker75335 | LG2 | 36698881 | 36698831 | 36698931 |
| Marker75340 | LG2 | 36703230 | 36703180 | 36703280 |
| Marker75341 | LG2 | 36703804 | 36703754 | 36703854 |
| Marker75342 | LG2 | 36704760 | 36704710 | 36704810 |
| Marker75347 | LG2 | 36721516 | 36721466 | 36721566 |
| Marker75364 | LG2 | 36759476 | 36759426 | 36759526 |
| Marker75369 | LG2 | 36778691 | 36778641 | 36778741 |
| Marker75391 | LG2 | 36853571 | 36853521 | 36853621 |
| Marker75393 | LG2 | 36858503 | 36858453 | 36858553 |
| Marker75437 | LG2 | 36960045 | 36959995 | 36960095 |
| Marker75458 | LG2 | 36988946 | 36988896 | 36988996 |
| Marker75470 | LG2 | 37019635 | 37019585 | 37019685 |
| Marker75472 | LG2 | 37021970 | 37021920 | 37022020 |
| Marker75480 | LG2 | 37034926 | 37034876 | 37034976 |
| Marker75485 | LG2 | 37039921 | 37039871 | 37039971 |
| Marker75518 | LG2 | 37110597 | 37110547 | 37110647 |
| Marker75519 | LG2 | 37110852 | 37110802 | 37110902 |
| Marker75528 | LG2 | 37141454 | 37141404 | 37141504 |
| Marker75532 | LG2 | 37147892 | 37147842 | 37147942 |
| Marker75547 | LG2 | 37167147 | 37167097 | 37167197 |
| Marker75552 | LG2 | 37175077 | 37175027 | 37175127 |
| Marker75569 | LG2 | 37202484 | 37202434 | 37202534 |
| Marker75570 | LG2 | 37203342 | 37203292 | 37203392 |
| Marker75582 | LG2 | 37258381 | 37258331 | 37258431 |
| Marker75593 | LG2 | 37304544 | 37304494 | 37304594 |
| Marker75594 | LG2 | 37304758 | 37304708 | 37304808 |
| Marker19156 | LG2 | 37313917 | 37313867 | 37313967 |
| Marker19167 | LG2 | 37332298 | 37332248 | 37332348 |
| Marker19173 | LG2 | 37335310 | 37335260 | 37335360 |
| Marker19185 | LG2 | 37353548 | 37353498 | 37353598 |
| Marker19205 | LG2 | 37371380 | 37371330 | 37371430 |
| Marker19211 | LG2 | 37377906 | 37377856 | 37377956 |
| Marker19217 | LG2 | 37378027 | 37377977 | 37378077 |
| Marker19226 | LG2 | 37382595 | 37382545 | 37382645 |
| Marker19249 | LG2 | 37412671 | 37412621 | 37412721 |
| Marker19287 | LG2 | 37467329 | 37467279 | 37467379 |
| Marker18254 | LG2 | 37575091 | 37575041 | 37575141 |
| Marker18261 | LG2 | 37584342 | 37584292 | 37584392 |
| Marker18265 | LG2 | 37586562 | 37586512 | 37586612 |
| Marker18266 | LG2 | 37588390 | 37588340 | 37588440 |
| Marker18268 | LG2 | 37588608 | 37588558 | 37588658 |
| Marker18314 | LG2 | 37673303 | 37673253 | 37673353 |
| Marker17871 | LG2 | 37725096 | 37725046 | 37725146 |
| Marker17877 | LG2 | 37735429 | 37735379 | 37735479 |
| Marker17878 | LG2 | 37735638 | 37735588 | 37735688 |
| Marker17879 | LG2 | 37741826 | 37741776 | 37741876 |
| Marker17919 | LG2 | 37783070 | 37783020 | 37783120 |
| Marker17922 | LG2 | 37785251 | 37785201 | 37785301 |
| Marker17941 | LG2 | 37805438 | 37805388 | 37805488 |
| Marker18000 | LG2 | 37942982 | 37942932 | 37943032 |
| Marker18007 | LG2 | 37952434 | 37952384 | 37952484 |
| Marker18008 | LG2 | 37952621 | 37952571 | 37952671 |
| Marker18010 | LG2 | 37955374 | 37955324 | 37955424 |
| Marker18023 | LG2 | 37965241 | 37965191 | 37965291 |
| Marker18045 | LG2 | 37974072 | 37974022 | 37974122 |
| Marker18110 | LG2 | 38057938 | 38057888 | 38057988 |
| Marker18124 | LG2 | 38109993 | 38109943 | 38110043 |
| Marker18129 | LG2 | 38133368 | 38133318 | 38133418 |
| Marker18144 | LG2 | 38150130 | 38150080 | 38150180 |
| Marker18164 | LG2 | 38197097 | 38197047 | 38197147 |
| Marker18180 | LG2 | 38222395 | 38222345 | 38222445 |
| Marker18194 | LG2 | 38237587 | 38237537 | 38237637 |
| Marker18197 | LG2 | 38241521 | 38241471 | 38241571 |
| Marker18205 | LG2 | 38260791 | 38260741 | 38260841 |
| Marker18206 | LG2 | 38260984 | 38260934 | 38261034 |
| Marker17824 | LG2 | 38290814 | 38290764 | 38290864 |
| Marker17814 | LG2 | 38299363 | 38299313 | 38299413 |
| Marker17783 | LG2 | 38344024 | 38343974 | 38344074 |
| Marker17757 | LG2 | 38368433 | 38368383 | 38368483 |
| Marker17754 | LG2 | 38370453 | 38370403 | 38370503 |
| Marker17706 | LG2 | 38467063 | 38467013 | 38467113 |
| Marker17702 | LG2 | 38467580 | 38467530 | 38467630 |
| Marker17680 | LG2 | 38522454 | 38522404 | 38522504 |
| Marker17657 | LG2 | 38535204 | 38535154 | 38535254 |
| Marker17644 | LG2 | 38560508 | 38560458 | 38560558 |
| Marker17643 | LG2 | 38563301 | 38563251 | 38563351 |
| Marker17642 | LG2 | 38565568 | 38565518 | 38565618 |
| Marker17595 | LG2 | 38673219 | 38673169 | 38673269 |
| Marker17590 | LG2 | 38686414 | 38686364 | 38686464 |
| Marker17589 | LG2 | 38686596 | 38686546 | 38686646 |
| Marker17554 | LG2 | 38727398 | 38727348 | 38727448 |
| Marker17546 | LG2 | 38755643 | 38755593 | 38755693 |
| Marker17534 | LG2 | 38786164 | 38786114 | 38786214 |
| Marker17521 | LG2 | 38801500 | 38801450 | 38801550 |
| Marker17519 | LG2 | 38817051 | 38817001 | 38817101 |
| Marker17518 | LG2 | 38817290 | 38817240 | 38817340 |
| Marker17514 | LG2 | 38818506 | 38818456 | 38818556 |
| Marker17510 | LG2 | 38823171 | 38823121 | 38823221 |
| Marker17505 | LG2 | 38845579 | 38845529 | 38845629 |
| Marker17502 | LG2 | 38849980 | 38849930 | 38850030 |
| Marker17108 | LG2 | 38905616 | 38905566 | 38905666 |
| Marker17107 | LG2 | 38911437 | 38911387 | 38911487 |
| Marker17091 | LG2 | 38919974 | 38919924 | 38920024 |
| Marker17040 | LG2 | 39020479 | 39020429 | 39020529 |
| Marker17021 | LG2 | 39027727 | 39027677 | 39027777 |
| Marker16986 | LG2 | 39075412 | 39075362 | 39075462 |
| Marker16981 | LG2 | 39077310 | 39077260 | 39077360 |
| Marker16964 | LG2 | 39093857 | 39093807 | 39093907 |
| Marker16959 | LG2 | 39107600 | 39107550 | 39107650 |
| Marker16950 | LG2 | 39107880 | 39107830 | 39107930 |
| Marker16915 | LG2 | 39132538 | 39132488 | 39132588 |
| Marker16902 | LG2 | 39146352 | 39146302 | 39146402 |
| Marker16894 | LG2 | 39166999 | 39166949 | 39167049 |
| Marker16889 | LG2 | 39180606 | 39180556 | 39180656 |
| Marker16886 | LG2 | 39189494 | 39189444 | 39189544 |
| Marker16864 | LG2 | 39228050 | 39228000 | 39228100 |
| Marker16853 | LG2 | 39238916 | 39238866 | 39238966 |
| Marker16852 | LG2 | 39242705 | 39242655 | 39242755 |
| Marker16808 | LG2 | 39295856 | 39295806 | 39295906 |
| Marker16739 | LG2 | 39440116 | 39440066 | 39440166 |
| Marker16712 | LG2 | 39468071 | 39468021 | 39468121 |
| Marker16707 | LG2 | 39468393 | 39468343 | 39468443 |
| Marker16706 | LG2 | 39469361 | 39469311 | 39469411 |
| Marker16705 | LG2 | 39469708 | 39469658 | 39469758 |
| Marker16699 | LG2 | 39474504 | 39474454 | 39474554 |
| Marker16687 | LG2 | 39496756 | 39496706 | 39496806 |
| Marker16671 | LG2 | 39522048 | 39521998 | 39522098 |
| Marker16637 | LG2 | 39611144 | 39611094 | 39611194 |
| Marker16618 | LG2 | 39670290 | 39670240 | 39670340 |
| Marker116130 | LG2 | 39694231 | 39694181 | 39694281 |
| Marker116116 | LG2 | 39746874 | 39746824 | 39746924 |
| Marker16495 | LG2 | 39850386 | 39850336 | 39850436 |
| Marker16499 | LG2 | 39856880 | 39856830 | 39856930 |
| Marker16523 | LG2 | 39888146 | 39888096 | 39888196 |
| Marker16547 | LG2 | 39905746 | 39905696 | 39905796 |
| Marker16551 | LG2 | 39906016 | 39905966 | 39906066 |
| Marker16574 | LG2 | 39942646 | 39942596 | 39942696 |
| Marker16575 | LG2 | 39943279 | 39943229 | 39943329 |
| Marker16581 | LG2 | 39943563 | 39943513 | 39943613 |
| Marker16317 | LG2 | 39975540 | 39975490 | 39975590 |
| Marker16308 | LG2 | 39982002 | 39981952 | 39982052 |
| Marker16300 | LG2 | 40013453 | 40013403 | 40013503 |
| Marker16294 | LG2 | 40019335 | 40019285 | 40019385 |
| Marker16293 | LG2 | 40024192 | 40024142 | 40024242 |
| Marker16278 | LG2 | 40041870 | 40041820 | 40041920 |
| Marker16277 | LG2 | 40045151 | 40045101 | 40045201 |
| Marker16272 | LG2 | 40052888 | 40052838 | 40052938 |
| Marker16229 | LG2 | 40162983 | 40162933 | 40163033 |
| Marker16204 | LG2 | 40184020 | 40183970 | 40184070 |
| Marker16194 | LG2 | 40205897 | 40205847 | 40205947 |
| Marker16189 | LG2 | 40207120 | 40207070 | 40207170 |
| Marker16163 | LG2 | 40209445 | 40209395 | 40209495 |
| Marker16142 | LG2 | 40217853 | 40217803 | 40217903 |
| Marker16115 | LG2 | 40281045 | 40280995 | 40281095 |
| Marker16110 | LG2 | 40281212 | 40281162 | 40281262 |
| Marker16096 | LG2 | 40296704 | 40296654 | 40296754 |
| Marker16094 | LG2 | 40298341 | 40298291 | 40298391 |
| Marker16084 | LG2 | 40319155 | 40319105 | 40319205 |
| Marker16066 | LG2 | 40335570 | 40335520 | 40335620 |
| Marker16031 | LG2 | 40389765 | 40389715 | 40389815 |
| Marker15999 | LG2 | 40428598 | 40428548 | 40428648 |
| Marker15996 | LG2 | 40432970 | 40432920 | 40433020 |
| Marker15974 | LG2 | 40463182 | 40463132 | 40463232 |
| Marker15973 | LG2 | 40463459 | 40463409 | 40463509 |
| Marker15968 | LG2 | 40479836 | 40479786 | 40479886 |
| Marker15953 | LG2 | 40495058 | 40495008 | 40495108 |
| Marker15950 | LG2 | 40501326 | 40501276 | 40501376 |
| Marker15943 | LG2 | 40509266 | 40509216 | 40509316 |
| Marker15926 | LG2 | 40551733 | 40551683 | 40551783 |
| Marker15910 | LG2 | 40564828 | 40564778 | 40564878 |
| Marker15890 | LG2 | 40580657 | 40580607 | 40580707 |
| Marker15886 | LG2 | 40583299 | 40583249 | 40583349 |
| Marker15885 | LG2 | 40595538 | 40595488 | 40595588 |
| Marker15884 | LG2 | 40596036 | 40595986 | 40596086 |
| Marker15878 | LG2 | 40596335 | 40596285 | 40596385 |
| Marker3555 | LG2 | 40648836 | 40648786 | 40648886 |
| Marker15859 | LG2 | 40655066 | 40655016 | 40655116 |
| Marker17423 | LG2 | 40767428 | 40767378 | 40767478 |
| Marker17385 | LG2 | 40836694 | 40836644 | 40836744 |
| Marker17366 | LG2 | 40857261 | 40857211 | 40857311 |
| Marker17355 | LG2 | 40864213 | 40864163 | 40864263 |
| Marker17331 | LG2 | 40899328 | 40899278 | 40899378 |
| Marker17257 | LG2 | 41022105 | 41022055 | 41022155 |
| Marker17233 | LG2 | 41038722 | 41038672 | 41038772 |
| Marker17217 | LG2 | 41060406 | 41060356 | 41060456 |
| Marker17216 | LG2 | 41076899 | 41076849 | 41076949 |
| Marker17215 | LG2 | 41077069 | 41077019 | 41077119 |
| Marker17209 | LG2 | 41095573 | 41095523 | 41095623 |
| Marker17200 | LG2 | 41103138 | 41103088 | 41103188 |
| Marker17183 | LG2 | 41139382 | 41139332 | 41139432 |
| Marker17165 | LG2 | 41159595 | 41159545 | 41159645 |
| Marker17159 | LG2 | 41180728 | 41180678 | 41180778 |
| Marker17144 | LG2 | 41228156 | 41228106 | 41228206 |
| Marker15684 | LG2 | 41275589 | 41275539 | 41275639 |
| Marker15677 | LG2 | 41295297 | 41295247 | 41295347 |
| Marker15669 | LG2 | 41317496 | 41317446 | 41317546 |
| Marker15664 | LG2 | 41319001 | 41318951 | 41319051 |
| Marker15662 | LG2 | 41325374 | 41325324 | 41325424 |
| Marker15661 | LG2 | 41327244 | 41327194 | 41327294 |
| Marker15646 | LG2 | 41352780 | 41352730 | 41352830 |
| Marker15630 | LG2 | 41361707 | 41361657 | 41361757 |
| Marker15476 | LG2 | 41482606 | 41482556 | 41482656 |
| Marker15462 | LG2 | 41500611 | 41500561 | 41500661 |
| Marker15440 | LG2 | 41532993 | 41532943 | 41533043 |
| Marker15436 | LG2 | 41535910 | 41535860 | 41535960 |
| Marker15435 | LG2 | 41540435 | 41540385 | 41540485 |
| Marker15432 | LG2 | 41540669 | 41540619 | 41540719 |
| Marker15431 | LG2 | 41541098 | 41541048 | 41541148 |
| Marker15398 | LG2 | 41647454 | 41647404 | 41647504 |
| Marker15392 | LG2 | 41654095 | 41654045 | 41654145 |
| Marker15378 | LG2 | 41674023 | 41673973 | 41674073 |
| Marker15375 | LG2 | 41680645 | 41680595 | 41680695 |
| Marker15358 | LG2 | 41699563 | 41699513 | 41699613 |
| Marker14756 | LG2 | 41751243 | 41751193 | 41751293 |
| Marker14757 | LG2 | 41752707 | 41752657 | 41752757 |
| Marker14759 | LG2 | 41752931 | 41752881 | 41752981 |
| Marker14780 | LG2 | 41761676 | 41761626 | 41761726 |
| Marker14783 | LG2 | 41762887 | 41762837 | 41762937 |
| Marker14785 | LG2 | 41768456 | 41768406 | 41768506 |
| Marker14798 | LG2 | 41782895 | 41782845 | 41782945 |
| Marker14821 | LG2 | 41840370 | 41840320 | 41840420 |
| Marker14854 | LG2 | 41943480 | 41943430 | 41943530 |
| Marker15342 | LG2 | 42032147 | 42032097 | 42032197 |
| Marker15320 | LG2 | 42056784 | 42056734 | 42056834 |
| Marker15306 | LG2 | 42070457 | 42070407 | 42070507 |
| Marker15299 | LG2 | 42084164 | 42084114 | 42084214 |
| Marker15282 | LG2 | 42095057 | 42095007 | 42095107 |
| Marker15279 | LG2 | 42102902 | 42102852 | 42102952 |
| Marker15278 | LG2 | 42106466 | 42106416 | 42106516 |
| Marker15267 | LG2 | 42125093 | 42125043 | 42125143 |
| Marker15237 | LG2 | 42187535 | 42187485 | 42187585 |
| Marker15212 | LG2 | 42238938 | 42238888 | 42238988 |
| Marker15193 | LG2 | 42267127 | 42267077 | 42267177 |
| Marker15174 | LG2 | 42300974 | 42300924 | 42301024 |
| Marker15152 | LG2 | 42350489 | 42350439 | 42350539 |
| Marker15143 | LG2 | 42370087 | 42370037 | 42370137 |
| Marker15130 | LG2 | 42405072 | 42405022 | 42405122 |
| Marker15116 | LG2 | 42450477 | 42450427 | 42450527 |
| Marker15115 | LG2 | 42451365 | 42451315 | 42451415 |
| Marker15102 | LG2 | 42454523 | 42454473 | 42454573 |
| Marker15100 | LG2 | 42465070 | 42465020 | 42465120 |
| Marker15093 | LG2 | 42476917 | 42476867 | 42476967 |
| Marker15089 | LG2 | 42493125 | 42493075 | 42493175 |
| Marker15088 | LG2 | 42494678 | 42494628 | 42494728 |
| Marker15067 | LG2 | 42511264 | 42511214 | 42511314 |
| Marker15066 | LG2 | 42512940 | 42512890 | 42512990 |
| Marker15061 | LG2 | 42520562 | 42520512 | 42520612 |
| Marker15060 | LG2 | 42520768 | 42520718 | 42520818 |
| Marker15004 | LG2 | 42567119 | 42567069 | 42567169 |
| Marker15003 | LG2 | 42572397 | 42572347 | 42572447 |
| Marker14998 | LG2 | 42577379 | 42577329 | 42577429 |
| Marker14990 | LG2 | 42601689 | 42601639 | 42601739 |
| Marker14964 | LG2 | 42670309 | 42670259 | 42670359 |
| Marker14963 | LG2 | 42670513 | 42670463 | 42670563 |
| Marker14962 | LG2 | 42678067 | 42678017 | 42678117 |
| Marker14955 | LG2 | 42685898 | 42685848 | 42685948 |
| Marker14952 | LG2 | 42708715 | 42708665 | 42708765 |
| Marker14924 | LG2 | 42753496 | 42753446 | 42753546 |
| Marker14901 | LG2 | 42861812 | 42861762 | 42861862 |
| Marker14899 | LG2 | 42866956 | 42866906 | 42867006 |
| Marker14886 | LG2 | 42879782 | 42879732 | 42879832 |
| Marker14885 | LG2 | 42879996 | 42879946 | 42880046 |
| Marker14873 | LG2 | 42919047 | 42918997 | 42919097 |
| Marker14872 | LG2 | 42921257 | 42921207 | 42921307 |
| Marker14871 | LG2 | 42925344 | 42925294 | 42925394 |
| Marker45697 | LG3 | 15243 | 15193 | 15293 |
| Marker45696 | LG3 | 26411 | 26361 | 26461 |
| Marker45681 | LG3 | 71442 | 71392 | 71492 |
| Marker46824 | LG3 | 195058 | 195008 | 195108 |
| Marker46832 | LG3 | 200007 | 199957 | 200057 |
| Marker46864 | LG3 | 236031 | 235981 | 236081 |
| Marker46903 | LG3 | 298658 | 298608 | 298708 |
| Marker46937 | LG3 | 337654 | 337604 | 337704 |
| Marker46958 | LG3 | 466735 | 466685 | 466785 |
| Marker46960 | LG3 | 466873 | 466823 | 466923 |
| Marker46628 | LG3 | 742728 | 742678 | 742778 |
| Marker46593 | LG3 | 777600 | 777550 | 777650 |
| Marker46578 | LG3 | 812334 | 812284 | 812384 |
| Marker46541 | LG3 | 888370 | 888320 | 888420 |
| Marker46540 | LG3 | 888428 | 888378 | 888478 |
| Marker46522 | LG3 | 918103 | 918053 | 918153 |
| Marker46508 | LG3 | 944260 | 944210 | 944310 |
| Marker45293 | LG3 | 1267450 | 1267400 | 1267500 |
| Marker45301 | LG3 | 1276825 | 1276775 | 1276875 |
| Marker45332 | LG3 | 1300616 | 1300566 | 1300666 |
| Marker45334 | LG3 | 1301484 | 1301434 | 1301534 |
| Marker45343 | LG3 | 1328059 | 1328009 | 1328109 |
| Marker45351 | LG3 | 1328719 | 1328669 | 1328769 |
| Marker45354 | LG3 | 1328973 | 1328923 | 1329023 |
| Marker45356 | LG3 | 1336599 | 1336549 | 1336649 |
| Marker45372 | LG3 | 1356423 | 1356373 | 1356473 |
| Marker45375 | LG3 | 1366622 | 1366572 | 1366672 |
| Marker45466 | LG3 | 1451859 | 1451809 | 1451909 |
| Marker45472 | LG3 | 1453658 | 1453608 | 1453708 |
| Marker45497 | LG3 | 1472008 | 1471958 | 1472058 |
| Marker45507 | LG3 | 1477467 | 1477417 | 1477517 |
| Marker45523 | LG3 | 1505622 | 1505572 | 1505672 |
| Marker115636 | LG3 | 2075309 | 2075259 | 2075359 |
| Marker115632 | LG3 | 2076291 | 2076241 | 2076341 |
| Marker46363 | LG3 | 2235900 | 2235850 | 2235950 |
| Marker46387 | LG3 | 2254546 | 2254496 | 2254596 |
| Marker46400 | LG3 | 2272831 | 2272781 | 2272881 |
| Marker46415 | LG3 | 2280334 | 2280284 | 2280384 |
| Marker46440 | LG3 | 2331966 | 2331916 | 2332016 |
| Marker46457 | LG3 | 2358893 | 2358843 | 2358943 |
| Marker48301 | LG3 | 2500874 | 2500824 | 2500924 |
| Marker48307 | LG3 | 2514226 | 2514176 | 2514276 |
| Marker48323 | LG3 | 2540634 | 2540584 | 2540684 |
| Marker48324 | LG3 | 2541334 | 2541284 | 2541384 |
| Marker48950 | LG3 | 2566296 | 2566246 | 2566346 |
| Marker48947 | LG3 | 2601071 | 2601021 | 2601121 |
| Marker48946 | LG3 | 2605238 | 2605188 | 2605288 |
| Marker47193 | LG3 | 2946540 | 2946490 | 2946590 |
| Marker47972 | LG3 | 3062097 | 3062047 | 3062147 |
| Marker47978 | LG3 | 3068081 | 3068031 | 3068131 |
| Marker47980 | LG3 | 3069086 | 3069036 | 3069136 |
| Marker48037 | LG3 | 3170053 | 3170003 | 3170103 |
| Marker122061 | LG3 | 3200251 | 3200201 | 3200301 |
| Marker48082 | LG3 | 3263311 | 3263261 | 3263361 |
| Marker48100 | LG3 | 3432858 | 3432808 | 3432908 |
| Marker48131 | LG3 | 3470180 | 3470130 | 3470230 |
| Marker48150 | LG3 | 3499398 | 3499348 | 3499448 |
| Marker47656 | LG3 | 3585697 | 3585647 | 3585747 |
| Marker47658 | LG3 | 3587647 | 3587597 | 3587697 |
| Marker47691 | LG3 | 3642927 | 3642877 | 3642977 |
| Marker47795 | LG3 | 3800579 | 3800529 | 3800629 |
| Marker47800 | LG3 | 3800772 | 3800722 | 3800822 |
| Marker47841 | LG3 | 3836934 | 3836884 | 3836984 |
| Marker47846 | LG3 | 3839069 | 3839019 | 3839119 |
| Marker47849 | LG3 | 3839913 | 3839863 | 3839963 |
| Marker15426 | LG3 | 3892281 | 3892231 | 3892331 |
| Marker47892 | LG3 | 3942438 | 3942388 | 3942488 |
| Marker47927 | LG3 | 3960309 | 3960259 | 3960359 |
| Marker47932 | LG3 | 3963229 | 3963179 | 3963279 |
| Marker47933 | LG3 | 3968198 | 3968148 | 3968248 |
| Marker47935 | LG3 | 3969303 | 3969253 | 3969353 |
| Marker47941 | LG3 | 3974139 | 3974089 | 3974189 |
| Marker47461 | LG3 | 4036115 | 4036065 | 4036165 |
| Marker47459 | LG3 | 4043337 | 4043287 | 4043387 |
| Marker47456 | LG3 | 4047485 | 4047435 | 4047535 |
| Marker49150 | LG3 | 4131722 | 4131672 | 4131772 |
| Marker47452 | LG3 | 4156545 | 4156495 | 4156595 |
| Marker47445 | LG3 | 4170929 | 4170879 | 4170979 |
| Marker47399 | LG3 | 4341499 | 4341449 | 4341549 |
| Marker4990 | LG3 | 4354987 | 4354937 | 4355037 |
| Marker48425 | LG3 | 4401376 | 4401326 | 4401426 |
| Marker47874 | LG3 | 4508821 | 4508771 | 4508871 |
| Marker48358 | LG3 | 4610729 | 4610679 | 4610779 |
| Marker48222 | LG3 | 4644146 | 4644096 | 4644196 |
| Marker48218 | LG3 | 4659501 | 4659451 | 4659551 |
| Marker48217 | LG3 | 4660286 | 4660236 | 4660336 |
| Marker49039 | LG3 | 4895551 | 4895501 | 4895601 |
| Marker49062 | LG3 | 4951953 | 4951903 | 4952003 |
| Marker49076 | LG3 | 4953408 | 4953358 | 4953458 |
| Marker49083 | LG3 | 4956222 | 4956172 | 4956272 |
| Marker49085 | LG3 | 4958269 | 4958219 | 4958319 |
| Marker49094 | LG3 | 4981899 | 4981849 | 4981949 |
| Marker60206 | LG3 | 5015060 | 5015010 | 5015110 |
| Marker49120 | LG3 | 5020458 | 5020408 | 5020508 |
| Marker49127 | LG3 | 5027538 | 5027488 | 5027588 |
| Marker49135 | LG3 | 5035056 | 5035006 | 5035106 |
| Marker49174 | LG3 | 5130935 | 5130885 | 5130985 |
| Marker49176 | LG3 | 5132651 | 5132601 | 5132701 |
| Marker49216 | LG3 | 5179660 | 5179610 | 5179710 |
| Marker49228 | LG3 | 5210617 | 5210567 | 5210667 |
| Marker49235 | LG3 | 5223391 | 5223341 | 5223441 |
| Marker49239 | LG3 | 5224657 | 5224607 | 5224707 |
| Marker49280 | LG3 | 5294257 | 5294207 | 5294307 |
| Marker49324 | LG3 | 5403476 | 5403426 | 5403526 |
| Marker113092 | LG3 | 5474439 | 5474389 | 5474489 |
| Marker112039 | LG3 | 5511656 | 5511606 | 5511706 |
| Marker49361 | LG3 | 5535621 | 5535571 | 5535671 |
| Marker100126 | LG3 | 5556611 | 5556561 | 5556661 |
| Marker48703 | LG3 | 5744080 | 5744030 | 5744130 |
| Marker48648 | LG3 | 5810331 | 5810281 | 5810381 |
| Marker46250 | LG3 | 5979250 | 5979200 | 5979300 |
| Marker46251 | LG3 | 5979429 | 5979379 | 5979479 |
| Marker44779 | LG3 | 6178366 | 6178316 | 6178416 |
| Marker44705 | LG3 | 6257235 | 6257185 | 6257285 |
| Marker44694 | LG3 | 6266300 | 6266250 | 6266350 |
| Marker44692 | LG3 | 6266464 | 6266414 | 6266514 |
| Marker45699 | LG3 | 6561167 | 6561117 | 6561217 |
| Marker45790 | LG3 | 6669078 | 6669028 | 6669128 |
| Marker45797 | LG3 | 6687565 | 6687515 | 6687615 |
| Marker45799 | LG3 | 6689127 | 6689077 | 6689177 |
| Marker45802 | LG3 | 6689732 | 6689682 | 6689782 |
| Marker45624 | LG3 | 6830944 | 6830894 | 6830994 |
| Marker45611 | LG3 | 6846413 | 6846363 | 6846463 |
| Marker45601 | LG3 | 6848257 | 6848207 | 6848307 |
| Marker45562 | LG3 | 6901226 | 6901176 | 6901276 |
| Marker45561 | LG3 | 6901271 | 6901221 | 6901321 |
| Marker118098 | LG3 | 6937996 | 6937946 | 6938046 |
| Marker118412 | LG3 | 7015967 | 7015917 | 7016017 |
| Marker45849 | LG3 | 7046468 | 7046418 | 7046518 |
| Marker45870 | LG3 | 7070549 | 7070499 | 7070599 |
| Marker45887 | LG3 | 7131858 | 7131808 | 7131908 |
| Marker45889 | LG3 | 7131922 | 7131872 | 7131972 |
| Marker46110 | LG3 | 7924252 | 7924202 | 7924302 |
| Marker104096 | LG3 | 7970637 | 7970587 | 7970687 |
| Marker45080 | LG3 | 8150891 | 8150841 | 8150941 |
| Marker45044 | LG3 | 8224006 | 8223956 | 8224056 |
| Marker82278 | LG3 | 8234091 | 8234041 | 8234141 |
| Marker45025 | LG3 | 8355580 | 8355530 | 8355630 |
| Marker45024 | LG3 | 8355793 | 8355743 | 8355843 |
| Marker113822 | LG3 | 8381097 | 8381047 | 8381147 |
| Marker113783 | LG3 | 8481965 | 8481915 | 8482015 |
| Marker119672 | LG3 | 8716216 | 8716166 | 8716266 |
| Marker45632 | LG3 | 8716550 | 8716500 | 8716600 |
| Marker45648 | LG3 | 8729295 | 8729245 | 8729345 |
| Marker44933 | LG3 | 8892355 | 8892305 | 8892405 |
| Marker44934 | LG3 | 8893416 | 8893366 | 8893466 |
| Marker44943 | LG3 | 8905756 | 8905706 | 8905806 |
| Marker44610 | LG3 | 9036202 | 9036152 | 9036252 |
| Marker44609 | LG3 | 9036255 | 9036205 | 9036305 |
| Marker44571 | LG3 | 9079688 | 9079638 | 9079738 |
| Marker44565 | LG3 | 9089982 | 9089932 | 9090032 |
| Marker44560 | LG3 | 9099241 | 9099191 | 9099291 |
| Marker44536 | LG3 | 9140788 | 9140738 | 9140838 |
| Marker44441 | LG3 | 9216760 | 9216710 | 9216810 |
| Marker43910 | LG3 | 9337234 | 9337184 | 9337284 |
| Marker43944 | LG3 | 9383699 | 9383649 | 9383749 |
| Marker43961 | LG3 | 9406107 | 9406057 | 9406157 |
| Marker43962 | LG3 | 9410197 | 9410147 | 9410247 |
| Marker43965 | LG3 | 9410658 | 9410608 | 9410708 |
| Marker44036 | LG3 | 9552813 | 9552763 | 9552863 |
| Marker44037 | LG3 | 9563130 | 9563080 | 9563180 |
| Marker44038 | LG3 | 9563339 | 9563289 | 9563389 |
| Marker44114 | LG3 | 9612778 | 9612728 | 9612828 |
| Marker44139 | LG3 | 9667819 | 9667769 | 9667869 |
| Marker44180 | LG3 | 9728164 | 9728114 | 9728214 |
| Marker44189 | LG3 | 9750268 | 9750218 | 9750318 |
| Marker44192 | LG3 | 9750859 | 9750809 | 9750909 |
| Marker44206 | LG3 | 9776675 | 9776625 | 9776725 |
| Marker44215 | LG3 | 9803278 | 9803228 | 9803328 |
| Marker44220 | LG3 | 9803576 | 9803526 | 9803626 |
| Marker44238 | LG3 | 9819027 | 9818977 | 9819077 |
| Marker44241 | LG3 | 9825958 | 9825908 | 9826008 |
| Marker44399 | LG3 | 9896993 | 9896943 | 9897043 |
| Marker44289 | LG3 | 9933412 | 9933362 | 9933462 |
| Marker44312 | LG3 | 9960738 | 9960688 | 9960788 |
| Marker44314 | LG3 | 9960929 | 9960879 | 9960979 |
| Marker44315 | LG3 | 9962187 | 9962137 | 9962237 |
| Marker44325 | LG3 | 9973445 | 9973395 | 9973495 |
| Marker44333 | LG3 | 9979506 | 9979456 | 9979556 |
| Marker44368 | LG3 | 10009279 | 10009229 | 10009329 |
| Marker44373 | LG3 | 10018960 | 10018910 | 10019010 |
| Marker44379 | LG3 | 10041888 | 10041838 | 10041938 |
| Marker44386 | LG3 | 10049372 | 10049322 | 10049422 |
| Marker44402 | LG3 | 10063141 | 10063091 | 10063191 |
| Marker43722 | LG3 | 10093984 | 10093934 | 10094034 |
| Marker43782 | LG3 | 10166780 | 10166730 | 10166830 |
| Marker43791 | LG3 | 10169329 | 10169279 | 10169379 |
| Marker43812 | LG3 | 10188981 | 10188931 | 10189031 |
| Marker43817 | LG3 | 10189199 | 10189149 | 10189249 |
| Marker43865 | LG3 | 10233800 | 10233750 | 10233850 |
| Marker43873 | LG3 | 10234345 | 10234295 | 10234395 |
| Marker43883 | LG3 | 10272544 | 10272494 | 10272594 |
| Marker43886 | LG3 | 10272788 | 10272738 | 10272838 |
| Marker112712 | LG3 | 10289703 | 10289653 | 10289753 |
| Marker112690 | LG3 | 10314014 | 10313964 | 10314064 |
| Marker112680 | LG3 | 10327881 | 10327831 | 10327931 |
| Marker112668 | LG3 | 10336750 | 10336700 | 10336800 |
| Marker112648 | LG3 | 10377382 | 10377332 | 10377432 |
| Marker112629 | LG3 | 10419343 | 10419293 | 10419393 |
| Marker112607 | LG3 | 10433068 | 10433018 | 10433118 |
| Marker112595 | LG3 | 10447904 | 10447854 | 10447954 |
| Marker112562 | LG3 | 10484947 | 10484897 | 10484997 |
| Marker112525 | LG3 | 10495889 | 10495839 | 10495939 |
| Marker112530 | LG3 | 10564417 | 10564367 | 10564467 |
| Marker112481 | LG3 | 10633918 | 10633868 | 10633968 |
| Marker112476 | LG3 | 10646542 | 10646492 | 10646592 |
| Marker112471 | LG3 | 10667865 | 10667815 | 10667915 |
| Marker112456 | LG3 | 10692394 | 10692344 | 10692444 |
| Marker112444 | LG3 | 10701367 | 10701317 | 10701417 |
| Marker112442 | LG3 | 10701616 | 10701566 | 10701666 |
| Marker112436 | LG3 | 10712478 | 10712428 | 10712528 |
| Marker112406 | LG3 | 10748362 | 10748312 | 10748412 |
| Marker43616 | LG3 | 10929362 | 10929312 | 10929412 |
| Marker43590 | LG3 | 10947036 | 10946986 | 10947086 |
| Marker43589 | LG3 | 10947297 | 10947247 | 10947347 |
| Marker43581 | LG3 | 10956509 | 10956459 | 10956559 |
| Marker43577 | LG3 | 10960505 | 10960455 | 10960555 |
| Marker43545 | LG3 | 11043371 | 11043321 | 11043421 |
| Marker43531 | LG3 | 11051567 | 11051517 | 11051617 |
| Marker43344 | LG3 | 11166528 | 11166478 | 11166578 |
| Marker43351 | LG3 | 11168214 | 11168164 | 11168264 |
| Marker43415 | LG3 | 11297955 | 11297905 | 11298005 |
| Marker43419 | LG3 | 11305407 | 11305357 | 11305457 |
| Marker43420 | LG3 | 11321580 | 11321530 | 11321630 |
| Marker43428 | LG3 | 11327122 | 11327072 | 11327172 |
| Marker43436 | LG3 | 11332924 | 11332874 | 11332974 |
| Marker43451 | LG3 | 11346898 | 11346848 | 11346948 |
| Marker43453 | LG3 | 11347080 | 11347030 | 11347130 |
| Marker43483 | LG3 | 11359996 | 11359946 | 11360046 |
| Marker43495 | LG3 | 11375565 | 11375515 | 11375615 |
| Marker43500 | LG3 | 11382471 | 11382421 | 11382521 |
| Marker43501 | LG3 | 11384775 | 11384725 | 11384825 |
| Marker43505 | LG3 | 11396291 | 11396241 | 11396341 |
| Marker43509 | LG3 | 11402677 | 11402627 | 11402727 |
| Marker43522 | LG3 | 11413359 | 11413309 | 11413409 |
| Marker43314 | LG3 | 11577118 | 11577068 | 11577168 |
| Marker43299 | LG3 | 11617119 | 11617069 | 11617169 |
| Marker43286 | LG3 | 11639558 | 11639508 | 11639608 |
| Marker43236 | LG3 | 11748613 | 11748563 | 11748663 |
| Marker43235 | LG3 | 11754787 | 11754737 | 11754837 |
| Marker43222 | LG3 | 11771199 | 11771149 | 11771249 |
| Marker43189 | LG3 | 11861480 | 11861430 | 11861530 |
| Marker43186 | LG3 | 11868082 | 11868032 | 11868132 |
| Marker43185 | LG3 | 11882735 | 11882685 | 11882785 |
| Marker43184 | LG3 | 11887361 | 11887311 | 11887411 |
| Marker43169 | LG3 | 11949643 | 11949593 | 11949693 |
| Marker43129 | LG3 | 12039126 | 12039076 | 12039176 |
| Marker43126 | LG3 | 12055666 | 12055616 | 12055716 |
| Marker43117 | LG3 | 12076846 | 12076796 | 12076896 |
| Marker43101 | LG3 | 12089770 | 12089720 | 12089820 |
| Marker43093 | LG3 | 12099925 | 12099875 | 12099975 |
| Marker43081 | LG3 | 12146230 | 12146180 | 12146280 |
| Marker43073 | LG3 | 12172671 | 12172621 | 12172721 |
| Marker43043 | LG3 | 12204775 | 12204725 | 12204825 |
| Marker42994 | LG3 | 12297999 | 12297949 | 12298049 |
| Marker42987 | LG3 | 12304473 | 12304423 | 12304523 |
| Marker42979 | LG3 | 12308291 | 12308241 | 12308341 |
| Marker42978 | LG3 | 12310129 | 12310079 | 12310179 |
| Marker42958 | LG3 | 12324279 | 12324229 | 12324329 |
| Marker42957 | LG3 | 12324880 | 12324830 | 12324930 |
| Marker42935 | LG3 | 12391760 | 12391710 | 12391810 |
| Marker42927 | LG3 | 12403600 | 12403550 | 12403650 |
| Marker42925 | LG3 | 12409324 | 12409274 | 12409374 |
| Marker42920 | LG3 | 12409587 | 12409537 | 12409637 |
| Marker42910 | LG3 | 12420564 | 12420514 | 12420614 |
| Marker42901 | LG3 | 12430214 | 12430164 | 12430264 |
| Marker42898 | LG3 | 12431705 | 12431655 | 12431755 |
| Marker42885 | LG3 | 12454038 | 12453988 | 12454088 |
| Marker42876 | LG3 | 12456920 | 12456870 | 12456970 |
| Marker42864 | LG3 | 12479444 | 12479394 | 12479494 |
| Marker42860 | LG3 | 12483621 | 12483571 | 12483671 |
| Marker42836 | LG3 | 12513256 | 12513206 | 12513306 |
| Marker42818 | LG3 | 12557235 | 12557185 | 12557285 |
| Marker42802 | LG3 | 12572853 | 12572803 | 12572903 |
| Marker42782 | LG3 | 12599743 | 12599693 | 12599793 |
| Marker42775 | LG3 | 12611194 | 12611144 | 12611244 |
| Marker42768 | LG3 | 12614767 | 12614717 | 12614817 |
| Marker42753 | LG3 | 12621600 | 12621550 | 12621650 |
| Marker42744 | LG3 | 12626268 | 12626218 | 12626318 |
| Marker42743 | LG3 | 12628477 | 12628427 | 12628527 |
| Marker42741 | LG3 | 12629476 | 12629426 | 12629526 |
| Marker42718 | LG3 | 12688201 | 12688151 | 12688251 |
| Marker121810 | LG3 | 12698808 | 12698758 | 12698858 |
| Marker42282 | LG3 | 12713928 | 12713878 | 12713978 |
| Marker42284 | LG3 | 12714155 | 12714105 | 12714205 |
| Marker42286 | LG3 | 12724792 | 12724742 | 12724842 |
| Marker42293 | LG3 | 12765861 | 12765811 | 12765911 |
| Marker42299 | LG3 | 12779303 | 12779253 | 12779353 |
| Marker42337 | LG3 | 12806379 | 12806329 | 12806429 |
| Marker35794 | LG3 | 12991060 | 12991010 | 12991110 |
| Marker42385 | LG3 | 13059739 | 13059689 | 13059789 |
| Marker42393 | LG3 | 13061960 | 13061910 | 13062010 |
| Marker118377 | LG3 | 13244054 | 13244004 | 13244104 |
| Marker42096 | LG3 | 13284740 | 13284690 | 13284790 |
| Marker42109 | LG3 | 13312459 | 13312409 | 13312509 |
| Marker42110 | LG3 | 13314322 | 13314272 | 13314372 |
| Marker42113 | LG3 | 13326001 | 13325951 | 13326051 |
| Marker42117 | LG3 | 13340504 | 13340454 | 13340554 |
| Marker42119 | LG3 | 13347861 | 13347811 | 13347911 |
| Marker42122 | LG3 | 13353412 | 13353362 | 13353462 |
| Marker42129 | LG3 | 13368261 | 13368211 | 13368311 |
| Marker42135 | LG3 | 13379251 | 13379201 | 13379301 |
| Marker42152 | LG3 | 13417588 | 13417538 | 13417638 |
| Marker42154 | LG3 | 13418668 | 13418618 | 13418718 |
| Marker42159 | LG3 | 13423437 | 13423387 | 13423487 |
| Marker42178 | LG3 | 13439400 | 13439350 | 13439450 |
| Marker42196 | LG3 | 13448485 | 13448435 | 13448535 |
| Marker42236 | LG3 | 13496223 | 13496173 | 13496273 |
| Marker42240 | LG3 | 13501415 | 13501365 | 13501465 |
| Marker42244 | LG3 | 13519344 | 13519294 | 13519394 |
| Marker42259 | LG3 | 13545301 | 13545251 | 13545351 |
| Marker42419 | LG3 | 13557408 | 13557358 | 13557458 |
| Marker42420 | LG3 | 13558170 | 13558120 | 13558220 |
| Marker42434 | LG3 | 13571523 | 13571473 | 13571573 |
| Marker42442 | LG3 | 13582830 | 13582780 | 13582880 |
| Marker42448 | LG3 | 13584095 | 13584045 | 13584145 |
| Marker42451 | LG3 | 13594464 | 13594414 | 13594514 |
| Marker42470 | LG3 | 13604386 | 13604336 | 13604436 |
| Marker42475 | LG3 | 13632780 | 13632730 | 13632830 |
| Marker42476 | LG3 | 13647802 | 13647752 | 13647852 |
| Marker42490 | LG3 | 13667600 | 13667550 | 13667650 |
| Marker42494 | LG3 | 13766286 | 13766236 | 13766336 |
| Marker42456 | LG3 | 13796253 | 13796203 | 13796303 |
| Marker42507 | LG3 | 13918917 | 13918867 | 13918967 |
| Marker42548 | LG3 | 13983449 | 13983399 | 13983499 |
| Marker42574 | LG3 | 14040098 | 14040048 | 14040148 |
| Marker42583 | LG3 | 14046355 | 14046305 | 14046405 |
| Marker42679 | LG3 | 14104370 | 14104320 | 14104420 |
| Marker42673 | LG3 | 14118516 | 14118466 | 14118566 |
| Marker42671 | LG3 | 14119194 | 14119144 | 14119244 |
| Marker42668 | LG3 | 14119799 | 14119749 | 14119849 |
| Marker42664 | LG3 | 14137179 | 14137129 | 14137229 |
| Marker42596 | LG3 | 14225152 | 14225102 | 14225202 |
| Marker41837 | LG3 | 14295449 | 14295399 | 14295499 |
| Marker41836 | LG3 | 14301419 | 14301369 | 14301469 |
| Marker41817 | LG3 | 14313157 | 14313107 | 14313207 |
| Marker41810 | LG3 | 14322689 | 14322639 | 14322739 |
| Marker41923 | LG3 | 14344277 | 14344227 | 14344327 |
| Marker41929 | LG3 | 14368080 | 14368030 | 14368130 |
| Marker41931 | LG3 | 14368140 | 14368090 | 14368190 |
| Marker119665 | LG3 | 14411818 | 14411768 | 14411868 |
| Marker41962 | LG3 | 14456270 | 14456220 | 14456320 |
| Marker42003 | LG3 | 14754312 | 14754262 | 14754362 |
| Marker42006 | LG3 | 14763327 | 14763277 | 14763377 |
| Marker111940 | LG3 | 14820146 | 14820096 | 14820196 |
| Marker42049 | LG3 | 14869973 | 14869923 | 14870023 |
| Marker42064 | LG3 | 14957826 | 14957776 | 14957876 |
| Marker42065 | LG3 | 14958072 | 14958022 | 14958122 |
| Marker42717 | LG3 | 15023029 | 15022979 | 15023079 |
| Marker42695 | LG3 | 15074941 | 15074891 | 15074991 |
| Marker42689 | LG3 | 15127632 | 15127582 | 15127682 |
| Marker41679 | LG3 | 15280789 | 15280739 | 15280839 |
| Marker41678 | LG3 | 15281304 | 15281254 | 15281354 |
| Marker41669 | LG3 | 15284758 | 15284708 | 15284808 |
| Marker41668 | LG3 | 15284915 | 15284865 | 15284965 |
| Marker41650 | LG3 | 15327650 | 15327600 | 15327700 |
| Marker41643 | LG3 | 15336356 | 15336306 | 15336406 |
| Marker41630 | LG3 | 15377752 | 15377702 | 15377802 |
| Marker41604 | LG3 | 15421873 | 15421823 | 15421923 |
| Marker41558 | LG3 | 15452648 | 15452598 | 15452698 |
| Marker41557 | LG3 | 15453398 | 15453348 | 15453448 |
| Marker41545 | LG3 | 15476035 | 15475985 | 15476085 |
| Marker41539 | LG3 | 15491762 | 15491712 | 15491812 |
| Marker41536 | LG3 | 15493196 | 15493146 | 15493246 |
| Marker41510 | LG3 | 15533026 | 15532976 | 15533076 |
| Marker41484 | LG3 | 15598011 | 15597961 | 15598061 |
| Marker41718 | LG3 | 15730948 | 15730898 | 15730998 |
| Marker40827 | LG3 | 15887081 | 15887031 | 15887131 |
| Marker40872 | LG3 | 15983012 | 15982962 | 15983062 |
| Marker40915 | LG3 | 16028639 | 16028589 | 16028689 |
| Marker41040 | LG3 | 16110487 | 16110437 | 16110537 |
| Marker40952 | LG3 | 16119080 | 16119030 | 16119130 |
| Marker40969 | LG3 | 16152004 | 16151954 | 16152054 |
| Marker40973 | LG3 | 16156579 | 16156529 | 16156629 |
| Marker40981 | LG3 | 16178913 | 16178863 | 16178963 |
| Marker41021 | LG3 | 16232512 | 16232462 | 16232562 |
| Marker41046 | LG3 | 16265139 | 16265089 | 16265189 |
| Marker41056 | LG3 | 16273376 | 16273326 | 16273426 |
| Marker41065 | LG3 | 16304945 | 16304895 | 16304995 |
| Marker41071 | LG3 | 16312315 | 16312265 | 16312365 |
| Marker41073 | LG3 | 16322495 | 16322445 | 16322545 |
| Marker41074 | LG3 | 16335892 | 16335842 | 16335942 |
| Marker41076 | LG3 | 16347264 | 16347214 | 16347314 |
| Marker41091 | LG3 | 16369761 | 16369711 | 16369811 |
| Marker41136 | LG3 | 16406444 | 16406394 | 16406494 |
| Marker41140 | LG3 | 16410198 | 16410148 | 16410248 |
| Marker41144 | LG3 | 16412564 | 16412514 | 16412614 |
| Marker41152 | LG3 | 16433992 | 16433942 | 16434042 |
| Marker41172 | LG3 | 16456822 | 16456772 | 16456872 |
| Marker41181 | LG3 | 16476212 | 16476162 | 16476262 |
| Marker41182 | LG3 | 16479648 | 16479598 | 16479698 |
| Marker41183 | LG3 | 16483363 | 16483313 | 16483413 |
| Marker41280 | LG3 | 16565573 | 16565523 | 16565623 |
| Marker41281 | LG3 | 16569719 | 16569669 | 16569769 |
| Marker41282 | LG3 | 16569939 | 16569889 | 16569989 |
| Marker41284 | LG3 | 16580677 | 16580627 | 16580727 |
| Marker41285 | LG3 | 16584284 | 16584234 | 16584334 |
| Marker73843 | LG3 | 16657671 | 16657621 | 16657721 |
| Marker41340 | LG3 | 16687337 | 16687287 | 16687387 |
| Marker41344 | LG3 | 16689512 | 16689462 | 16689562 |
| Marker41347 | LG3 | 16695923 | 16695873 | 16695973 |
| Marker41351 | LG3 | 16696879 | 16696829 | 16696929 |
| Marker41353 | LG3 | 16707061 | 16707011 | 16707111 |
| Marker41355 | LG3 | 16728116 | 16728066 | 16728166 |
| Marker41358 | LG3 | 16733136 | 16733086 | 16733186 |
| Marker41359 | LG3 | 16749577 | 16749527 | 16749627 |
| Marker41369 | LG3 | 16754941 | 16754891 | 16754991 |
| Marker40426 | LG3 | 16892303 | 16892253 | 16892353 |
| Marker40438 | LG3 | 16906125 | 16906075 | 16906175 |
| Marker40446 | LG3 | 16910935 | 16910885 | 16910985 |
| Marker40449 | LG3 | 16934473 | 16934423 | 16934523 |
| Marker40451 | LG3 | 16937195 | 16937145 | 16937245 |
| Marker40455 | LG3 | 16956125 | 16956075 | 16956175 |
| Marker40458 | LG3 | 16958684 | 16958634 | 16958734 |
| Marker40466 | LG3 | 16994063 | 16994013 | 16994113 |
| Marker40472 | LG3 | 17009882 | 17009832 | 17009932 |
| Marker40473 | LG3 | 17010094 | 17010044 | 17010144 |
| Marker40475 | LG3 | 17018882 | 17018832 | 17018932 |
| Marker40503 | LG3 | 17127590 | 17127540 | 17127640 |
| Marker40555 | LG3 | 17137491 | 17137441 | 17137541 |
| Marker40578 | LG3 | 17154438 | 17154388 | 17154488 |
| Marker40583 | LG3 | 17164487 | 17164437 | 17164537 |
| Marker40585 | LG3 | 17179415 | 17179365 | 17179465 |
| Marker40586 | LG3 | 17182645 | 17182595 | 17182695 |
| Marker40588 | LG3 | 17183865 | 17183815 | 17183915 |
| Marker40603 | LG3 | 17237631 | 17237581 | 17237681 |
| Marker40607 | LG3 | 17255915 | 17255865 | 17255965 |
| Marker40613 | LG3 | 17268750 | 17268700 | 17268800 |
| Marker40666 | LG3 | 17426976 | 17426926 | 17427026 |
| Marker40667 | LG3 | 17427014 | 17426964 | 17427064 |
| Marker40668 | LG3 | 17430639 | 17430589 | 17430689 |
| Marker40677 | LG3 | 17448566 | 17448516 | 17448616 |
| Marker40697 | LG3 | 17482363 | 17482313 | 17482413 |
| Marker40702 | LG3 | 17502864 | 17502814 | 17502914 |
| Marker40703 | LG3 | 17503068 | 17503018 | 17503118 |
| Marker40712 | LG3 | 17515308 | 17515258 | 17515358 |
| Marker40713 | LG3 | 17518662 | 17518612 | 17518712 |
| Marker40714 | LG3 | 17522328 | 17522278 | 17522378 |
| Marker119270 | LG3 | 17539244 | 17539194 | 17539294 |
| Marker119272 | LG3 | 17539312 | 17539262 | 17539362 |
| Marker40770 | LG3 | 17637781 | 17637731 | 17637831 |
| Marker40798 | LG3 | 17652668 | 17652618 | 17652718 |
| Marker40802 | LG3 | 17657282 | 17657232 | 17657332 |
| Marker40371 | LG3 | 17777877 | 17777827 | 17777927 |
| Marker40381 | LG3 | 17786096 | 17786046 | 17786146 |
| Marker40397 | LG3 | 17814726 | 17814676 | 17814776 |
| Marker40398 | LG3 | 17819435 | 17819385 | 17819485 |
| Marker40412 | LG3 | 17831779 | 17831729 | 17831829 |
| Marker40329 | LG3 | 17935698 | 17935648 | 17935748 |
| Marker40313 | LG3 | 17962095 | 17962045 | 17962145 |
| Marker40287 | LG3 | 18005839 | 18005789 | 18005889 |
| Marker40281 | LG3 | 18038844 | 18038794 | 18038894 |
| Marker40277 | LG3 | 18090743 | 18090693 | 18090793 |
| Marker40276 | LG3 | 18106258 | 18106208 | 18106308 |
| Marker40274 | LG3 | 18116614 | 18116564 | 18116664 |
| Marker40253 | LG3 | 18147706 | 18147656 | 18147756 |
| Marker117514 | LG3 | 18218188 | 18218138 | 18218238 |
| Marker40198 | LG3 | 18317106 | 18317056 | 18317156 |
| Marker40181 | LG3 | 18371209 | 18371159 | 18371259 |
| Marker40171 | LG3 | 18378588 | 18378538 | 18378638 |
| Marker40169 | LG3 | 18385218 | 18385168 | 18385268 |
| Marker40165 | LG3 | 18390308 | 18390258 | 18390358 |
| Marker40163 | LG3 | 18417657 | 18417607 | 18417707 |
| Marker40152 | LG3 | 18436596 | 18436546 | 18436646 |
| Marker40141 | LG3 | 18449743 | 18449693 | 18449793 |
| Marker40136 | LG3 | 18449921 | 18449871 | 18449971 |
| Marker40128 | LG3 | 18460446 | 18460396 | 18460496 |
| Marker40108 | LG3 | 18485523 | 18485473 | 18485573 |
| Marker40087 | LG3 | 18497181 | 18497131 | 18497231 |
| Marker40079 | LG3 | 18497294 | 18497244 | 18497344 |
| Marker40084 | LG3 | 18497727 | 18497677 | 18497777 |
| Marker40075 | LG3 | 18508032 | 18507982 | 18508082 |
| Marker40074 | LG3 | 18510983 | 18510933 | 18511033 |
| Marker40024 | LG3 | 18604052 | 18604002 | 18604102 |
| Marker39997 | LG3 | 18685302 | 18685252 | 18685352 |
| Marker39996 | LG3 | 18689260 | 18689210 | 18689310 |
| Marker39991 | LG3 | 18694273 | 18694223 | 18694323 |
| Marker39959 | LG3 | 18728512 | 18728462 | 18728562 |
| Marker39938 | LG3 | 18781022 | 18780972 | 18781072 |
| Marker39888 | LG3 | 19111343 | 19111293 | 19111393 |
| Marker39874 | LG3 | 19122487 | 19122437 | 19122537 |
| Marker39862 | LG3 | 19151584 | 19151534 | 19151634 |
| Marker39811 | LG3 | 19225286 | 19225236 | 19225336 |
| Marker39791 | LG3 | 19243235 | 19243185 | 19243285 |
| Marker39789 | LG3 | 19246023 | 19245973 | 19246073 |
| Marker39788 | LG3 | 19246234 | 19246184 | 19246284 |
| Marker39787 | LG3 | 19247238 | 19247188 | 19247288 |
| Marker39770 | LG3 | 19263182 | 19263132 | 19263232 |
| Marker39757 | LG3 | 19308004 | 19307954 | 19308054 |
| Marker39753 | LG3 | 19310296 | 19310246 | 19310346 |
| Marker39751 | LG3 | 19310687 | 19310637 | 19310737 |
| Marker39744 | LG3 | 19319719 | 19319669 | 19319769 |
| Marker39735 | LG3 | 19332676 | 19332626 | 19332726 |
| Marker39730 | LG3 | 19338046 | 19337996 | 19338096 |
| Marker39696 | LG3 | 19368115 | 19368065 | 19368165 |
| Marker39689 | LG3 | 19373371 | 19373321 | 19373421 |
| Marker39687 | LG3 | 19373986 | 19373936 | 19374036 |
| Marker39669 | LG3 | 19405637 | 19405587 | 19405687 |
| Marker39667 | LG3 | 19407210 | 19407160 | 19407260 |
| Marker39658 | LG3 | 19422590 | 19422540 | 19422640 |
| Marker39657 | LG3 | 19422794 | 19422744 | 19422844 |
| Marker39625 | LG3 | 19454746 | 19454696 | 19454796 |
| Marker39623 | LG3 | 19455516 | 19455466 | 19455566 |
| Marker39622 | LG3 | 19456310 | 19456260 | 19456360 |
| Marker39572 | LG3 | 19507573 | 19507523 | 19507623 |
| Marker39549 | LG3 | 19529922 | 19529872 | 19529972 |
| Marker39494 | LG3 | 19622035 | 19621985 | 19622085 |
| Marker39469 | LG3 | 19639253 | 19639203 | 19639303 |
| Marker39448 | LG3 | 19682837 | 19682787 | 19682887 |
| Marker39444 | LG3 | 19688662 | 19688612 | 19688712 |
| Marker39438 | LG3 | 19695592 | 19695542 | 19695642 |
| Marker39431 | LG3 | 19708911 | 19708861 | 19708961 |
| Marker39430 | LG3 | 19710949 | 19710899 | 19710999 |
| Marker39422 | LG3 | 19729004 | 19728954 | 19729054 |
| Marker39421 | LG3 | 19730480 | 19730430 | 19730530 |
| Marker39420 | LG3 | 19734551 | 19734501 | 19734601 |
| Marker39397 | LG3 | 19839832 | 19839782 | 19839882 |
| Marker39343 | LG3 | 19894383 | 19894333 | 19894433 |
| Marker39275 | LG3 | 20007815 | 20007765 | 20007865 |
| Marker39272 | LG3 | 20007976 | 20007926 | 20008026 |
| Marker39268 | LG3 | 20104181 | 20104131 | 20104231 |
| Marker39265 | LG3 | 20104451 | 20104401 | 20104501 |
| Marker39247 | LG3 | 20147055 | 20147005 | 20147105 |
| Marker39227 | LG3 | 20207722 | 20207672 | 20207772 |
| Marker39221 | LG3 | 20210903 | 20210853 | 20210953 |
| Marker39211 | LG3 | 20241331 | 20241281 | 20241381 |
| Marker39204 | LG3 | 20258191 | 20258141 | 20258241 |
| Marker39196 | LG3 | 20284390 | 20284340 | 20284440 |
| Marker39178 | LG3 | 20321213 | 20321163 | 20321263 |
| Marker39173 | LG3 | 20344782 | 20344732 | 20344832 |
| Marker39160 | LG3 | 20372291 | 20372241 | 20372341 |
| Marker39153 | LG3 | 20384905 | 20384855 | 20384955 |
| Marker39138 | LG3 | 20426365 | 20426315 | 20426415 |
| Marker39116 | LG3 | 20430570 | 20430520 | 20430620 |
| Marker39099 | LG3 | 20469140 | 20469090 | 20469190 |
| Marker39080 | LG3 | 20528989 | 20528939 | 20529039 |
| Marker39077 | LG3 | 20529208 | 20529158 | 20529258 |
| Marker39076 | LG3 | 20530466 | 20530416 | 20530516 |
| Marker39075 | LG3 | 20530540 | 20530490 | 20530590 |
| Marker39018 | LG3 | 20608985 | 20608935 | 20609035 |
| Marker39007 | LG3 | 20644938 | 20644888 | 20644988 |
| Marker39002 | LG3 | 20654560 | 20654510 | 20654610 |
| Marker38977 | LG3 | 20681185 | 20681135 | 20681235 |
| Marker38929 | LG3 | 20747610 | 20747560 | 20747660 |
| Marker38921 | LG3 | 20786995 | 20786945 | 20787045 |
| Marker38913 | LG3 | 20793674 | 20793624 | 20793724 |
| Marker38908 | LG3 | 20802851 | 20802801 | 20802901 |
| Marker38900 | LG3 | 20815951 | 20815901 | 20816001 |
| Marker38895 | LG3 | 20824290 | 20824240 | 20824340 |
| Marker38856 | LG3 | 20935333 | 20935283 | 20935383 |
| Marker38839 | LG3 | 20954286 | 20954236 | 20954336 |
| Marker26430 | LG3 | 20966520 | 20966470 | 20966570 |
| Marker26428 | LG3 | 20966714 | 20966664 | 20966764 |
| Marker38847 | LG3 | 20973671 | 20973621 | 20973721 |
| Marker121768 | LG3 | 20974630 | 20974580 | 20974680 |
| Marker38588 | LG3 | 21044893 | 21044843 | 21044943 |
| Marker38589 | LG3 | 21045403 | 21045353 | 21045453 |
| Marker38610 | LG3 | 21072268 | 21072218 | 21072318 |
| Marker38615 | LG3 | 21077485 | 21077435 | 21077535 |
| Marker38622 | LG3 | 21095407 | 21095357 | 21095457 |
| Marker38633 | LG3 | 21106870 | 21106820 | 21106920 |
| Marker38651 | LG3 | 21126003 | 21125953 | 21126053 |
| Marker38656 | LG3 | 21134886 | 21134836 | 21134936 |
| Marker38673 | LG3 | 21164299 | 21164249 | 21164349 |
| Marker38685 | LG3 | 21176997 | 21176947 | 21177047 |
| Marker38697 | LG3 | 21200700 | 21200650 | 21200750 |
| Marker38706 | LG3 | 21211941 | 21211891 | 21211991 |
| Marker38726 | LG3 | 21229012 | 21228962 | 21229062 |
| Marker38728 | LG3 | 21229045 | 21228995 | 21229095 |
| Marker38730 | LG3 | 21251505 | 21251455 | 21251555 |
| Marker38734 | LG3 | 21260091 | 21260041 | 21260141 |
| Marker38746 | LG3 | 21263265 | 21263215 | 21263315 |
| Marker38757 | LG3 | 21287093 | 21287043 | 21287143 |
| Marker38787 | LG3 | 21326300 | 21326250 | 21326350 |
| Marker38798 | LG3 | 21376610 | 21376560 | 21376660 |
| Marker38365 | LG3 | 21458554 | 21458504 | 21458604 |
| Marker38356 | LG3 | 21485040 | 21484990 | 21485090 |
| Marker38306 | LG3 | 21612838 | 21612788 | 21612888 |
| Marker38302 | LG3 | 21619186 | 21619136 | 21619236 |
| Marker38548 | LG3 | 21708252 | 21708202 | 21708302 |
| Marker38535 | LG3 | 21772693 | 21772643 | 21772743 |
| Marker38526 | LG3 | 21777050 | 21777000 | 21777100 |
| Marker38514 | LG3 | 21805802 | 21805752 | 21805852 |
| Marker38474 | LG3 | 21898773 | 21898723 | 21898823 |
| Marker38470 | LG3 | 21910071 | 21910021 | 21910121 |
| Marker38469 | LG3 | 21915720 | 21915670 | 21915770 |
| Marker38443 | LG3 | 21975970 | 21975920 | 21976020 |
| Marker38439 | LG3 | 21982563 | 21982513 | 21982613 |
| Marker38433 | LG3 | 21983086 | 21983036 | 21983136 |
| Marker38429 | LG3 | 21983820 | 21983770 | 21983870 |
| Marker38261 | LG3 | 22085010 | 22084960 | 22085060 |
| Marker38259 | LG3 | 22122977 | 22122927 | 22123027 |
| Marker38235 | LG3 | 22178136 | 22178086 | 22178186 |
| Marker38223 | LG3 | 22191468 | 22191418 | 22191518 |
| Marker38214 | LG3 | 22196412 | 22196362 | 22196462 |
| Marker38213 | LG3 | 22196854 | 22196804 | 22196904 |
| Marker38208 | LG3 | 22197778 | 22197728 | 22197828 |
| Marker38198 | LG3 | 22217210 | 22217160 | 22217260 |
| Marker38172 | LG3 | 22244281 | 22244231 | 22244331 |
| Marker38166 | LG3 | 22247010 | 22246960 | 22247060 |
| Marker38162 | LG3 | 22253078 | 22253028 | 22253128 |
| Marker38153 | LG3 | 22257710 | 22257660 | 22257760 |
| Marker38820 | LG3 | 22298619 | 22298569 | 22298669 |
| Marker118707 | LG3 | 22343572 | 22343522 | 22343622 |
| Marker38141 | LG3 | 22376963 | 22376913 | 22377013 |
| Marker38139 | LG3 | 22386130 | 22386080 | 22386180 |
| Marker38135 | LG3 | 22393665 | 22393615 | 22393715 |
| Marker38134 | LG3 | 22394687 | 22394637 | 22394737 |
| Marker38127 | LG3 | 22414314 | 22414264 | 22414364 |
| Marker38126 | LG3 | 22414520 | 22414470 | 22414570 |
| Marker38109 | LG3 | 22497911 | 22497861 | 22497961 |
| Marker38068 | LG3 | 22592811 | 22592761 | 22592861 |
| Marker38067 | LG3 | 22602276 | 22602226 | 22602326 |
| Marker38058 | LG3 | 22614250 | 22614200 | 22614300 |
| Marker38055 | LG3 | 22628756 | 22628706 | 22628806 |
| Marker38043 | LG3 | 22677916 | 22677866 | 22677966 |
| Marker38035 | LG3 | 22681024 | 22680974 | 22681074 |
| Marker38032 | LG3 | 22681573 | 22681523 | 22681623 |
| Marker38030 | LG3 | 22694387 | 22694337 | 22694437 |
| Marker38019 | LG3 | 22717074 | 22717024 | 22717124 |
| Marker38015 | LG3 | 22717690 | 22717640 | 22717740 |
| Marker38005 | LG3 | 22740625 | 22740575 | 22740675 |
| Marker37984 | LG3 | 22753200 | 22753150 | 22753250 |
| Marker37975 | LG3 | 22790736 | 22790686 | 22790786 |
| Marker37965 | LG3 | 22811576 | 22811526 | 22811626 |
| Marker37945 | LG3 | 22818280 | 22818230 | 22818330 |
| Marker37909 | LG3 | 22866038 | 22865988 | 22866088 |
| Marker37897 | LG3 | 22889984 | 22889934 | 22890034 |
| Marker37884 | LG3 | 22932773 | 22932723 | 22932823 |
| Marker37855 | LG3 | 22975032 | 22974982 | 22975082 |
| Marker37832 | LG3 | 23045191 | 23045141 | 23045241 |
| Marker37811 | LG3 | 23107000 | 23106950 | 23107050 |
| Marker37792 | LG3 | 23148231 | 23148181 | 23148281 |
| Marker37757 | LG3 | 23204146 | 23204096 | 23204196 |
| Marker37756 | LG3 | 23204336 | 23204286 | 23204386 |
| Marker37721 | LG3 | 23248295 | 23248245 | 23248345 |
| Marker37689 | LG3 | 23310561 | 23310511 | 23310611 |
| Marker37685 | LG3 | 23313299 | 23313249 | 23313349 |
| Marker37662 | LG3 | 23333901 | 23333851 | 23333951 |
| Marker37636 | LG3 | 23393761 | 23393711 | 23393811 |
| Marker37631 | LG3 | 23395415 | 23395365 | 23395465 |
| Marker37630 | LG3 | 23396942 | 23396892 | 23396992 |
| Marker37628 | LG3 | 23398663 | 23398613 | 23398713 |
| Marker37627 | LG3 | 23406722 | 23406672 | 23406772 |
| Marker37622 | LG3 | 23417885 | 23417835 | 23417935 |
| Marker37609 | LG3 | 23489778 | 23489728 | 23489828 |
| Marker37600 | LG3 | 23508154 | 23508104 | 23508204 |
| Marker37587 | LG3 | 23517288 | 23517238 | 23517338 |
| Marker37571 | LG3 | 23550543 | 23550493 | 23550593 |
| Marker37570 | LG3 | 23578405 | 23578355 | 23578455 |
| Marker37568 | LG3 | 23584423 | 23584373 | 23584473 |
| Marker37565 | LG3 | 23595692 | 23595642 | 23595742 |
| Marker37562 | LG3 | 23609875 | 23609825 | 23609925 |
| Marker37558 | LG3 | 23651152 | 23651102 | 23651202 |
| Marker37555 | LG3 | 23665744 | 23665694 | 23665794 |
| Marker37554 | LG3 | 23668650 | 23668600 | 23668700 |
| Marker37529 | LG3 | 23710802 | 23710752 | 23710852 |
| Marker37514 | LG3 | 23734904 | 23734854 | 23734954 |
| Marker37138 | LG3 | 23902887 | 23902837 | 23902937 |
| Marker37440 | LG3 | 23914752 | 23914702 | 23914802 |
| Marker37429 | LG3 | 23918448 | 23918398 | 23918498 |
| Marker37423 | LG3 | 23923078 | 23923028 | 23923128 |
| Marker37421 | LG3 | 23923958 | 23923908 | 23924008 |
| Marker37408 | LG3 | 23935834 | 23935784 | 23935884 |
| Marker37407 | LG3 | 23937632 | 23937582 | 23937682 |
| Marker37398 | LG3 | 23961043 | 23960993 | 23961093 |
| Marker37395 | LG3 | 23981352 | 23981302 | 23981402 |
| Marker37390 | LG3 | 23994104 | 23994054 | 23994154 |
| Marker37389 | LG3 | 23996830 | 23996780 | 23996880 |
| Marker37382 | LG3 | 24015221 | 24015171 | 24015271 |
| Marker37375 | LG3 | 24019834 | 24019784 | 24019884 |
| Marker37365 | LG3 | 24043326 | 24043276 | 24043376 |
| Marker37363 | LG3 | 24044508 | 24044458 | 24044558 |
| Marker37357 | LG3 | 24059719 | 24059669 | 24059769 |
| Marker37350 | LG3 | 24067019 | 24066969 | 24067069 |
| Marker37330 | LG3 | 24169111 | 24169061 | 24169161 |
| Marker37328 | LG3 | 24177404 | 24177354 | 24177454 |
| Marker37324 | LG3 | 24182171 | 24182121 | 24182221 |
| Marker37320 | LG3 | 24193143 | 24193093 | 24193193 |
| Marker37315 | LG3 | 24193390 | 24193340 | 24193440 |
| Marker37313 | LG3 | 24202133 | 24202083 | 24202183 |
| Marker37284 | LG3 | 24236054 | 24236004 | 24236104 |
| Marker37279 | LG3 | 24263693 | 24263643 | 24263743 |
| Marker37145 | LG3 | 24306046 | 24305996 | 24306096 |
| Marker37148 | LG3 | 24327477 | 24327427 | 24327527 |
| Marker37155 | LG3 | 24333035 | 24332985 | 24333085 |
| Marker37161 | LG3 | 24339897 | 24339847 | 24339947 |
| Marker37163 | LG3 | 24345131 | 24345081 | 24345181 |
| Marker37192 | LG3 | 24398375 | 24398325 | 24398425 |
| Marker37194 | LG3 | 24407320 | 24407270 | 24407370 |
| Marker37195 | LG3 | 24416839 | 24416789 | 24416889 |
| Marker37217 | LG3 | 24458908 | 24458858 | 24458958 |
| Marker37229 | LG3 | 24483341 | 24483291 | 24483391 |
| Marker37246 | LG3 | 24526897 | 24526847 | 24526947 |
| Marker37259 | LG3 | 24539724 | 24539674 | 24539774 |
| Marker37267 | LG3 | 24550821 | 24550771 | 24550871 |
| Marker47628 | LG3 | 24594767 | 24594717 | 24594817 |
| Marker47614 | LG3 | 24606351 | 24606301 | 24606401 |
| Marker47610 | LG3 | 24608360 | 24608310 | 24608410 |
| Marker47598 | LG3 | 24632494 | 24632444 | 24632544 |
| Marker47593 | LG3 | 24639000 | 24638950 | 24639050 |
| Marker47583 | LG3 | 24652049 | 24651999 | 24652099 |
| Marker47582 | LG3 | 24652876 | 24652826 | 24652926 |
| Marker47578 | LG3 | 24673064 | 24673014 | 24673114 |
| Marker47572 | LG3 | 24684072 | 24684022 | 24684122 |
| Marker47571 | LG3 | 24684252 | 24684202 | 24684302 |
| Marker47563 | LG3 | 24697946 | 24697896 | 24697996 |
| Marker47557 | LG3 | 24701745 | 24701695 | 24701795 |
| Marker47553 | LG3 | 24713319 | 24713269 | 24713369 |
| Marker47543 | LG3 | 24722214 | 24722164 | 24722264 |
| Marker47537 | LG3 | 24738437 | 24738387 | 24738487 |
| Marker47533 | LG3 | 24744482 | 24744432 | 24744532 |
| Marker47526 | LG3 | 24754275 | 24754225 | 24754325 |
| Marker47514 | LG3 | 24774633 | 24774583 | 24774683 |
| Marker47501 | LG3 | 24799513 | 24799463 | 24799563 |
| Marker47498 | LG3 | 24801151 | 24801101 | 24801201 |
| Marker37017 | LG3 | 24878888 | 24878838 | 24878938 |
| Marker37124 | LG3 | 24960141 | 24960091 | 24960191 |
| Marker37110 | LG3 | 24989434 | 24989384 | 24989484 |
| Marker37107 | LG3 | 24995102 | 24995052 | 24995152 |
| Marker37089 | LG3 | 25033076 | 25033026 | 25033126 |
| Marker37084 | LG3 | 25039129 | 25039079 | 25039179 |
| Marker37078 | LG3 | 25044681 | 25044631 | 25044731 |
| Marker37074 | LG3 | 25049892 | 25049842 | 25049942 |
| Marker37063 | LG3 | 25055636 | 25055586 | 25055686 |
| Marker37061 | LG3 | 25062020 | 25061970 | 25062070 |
| Marker37044 | LG3 | 25093138 | 25093088 | 25093188 |
| Marker37038 | LG3 | 25103296 | 25103246 | 25103346 |
| Marker63202 | LG4 | 35116 | 35066 | 35166 |
| Marker63215 | LG4 | 78144 | 78094 | 78194 |
| Marker63218 | LG4 | 92347 | 92297 | 92397 |
| Marker63223 | LG4 | 108761 | 108711 | 108811 |
| Marker63228 | LG4 | 109859 | 109809 | 109909 |
| Marker63234 | LG4 | 118429 | 118379 | 118479 |
| Marker63263 | LG4 | 148461 | 148411 | 148511 |
| Marker63265 | LG4 | 149209 | 149159 | 149259 |
| Marker63271 | LG4 | 157739 | 157689 | 157789 |
| Marker63284 | LG4 | 170471 | 170421 | 170521 |
| Marker63299 | LG4 | 202135 | 202085 | 202185 |
| Marker63300 | LG4 | 203905 | 203855 | 203955 |
| Marker62796 | LG4 | 208884 | 208834 | 208934 |
| Marker62811 | LG4 | 218890 | 218840 | 218940 |
| Marker62812 | LG4 | 220596 | 220546 | 220646 |
| Marker62815 | LG4 | 227783 | 227733 | 227833 |
| Marker62825 | LG4 | 247497 | 247447 | 247547 |
| Marker62827 | LG4 | 247761 | 247711 | 247811 |
| Marker62859 | LG4 | 301884 | 301834 | 301934 |
| Marker62942 | LG4 | 507892 | 507842 | 507942 |
| Marker62943 | LG4 | 510138 | 510088 | 510188 |
| Marker62945 | LG4 | 512290 | 512240 | 512340 |
| Marker62949 | LG4 | 522064 | 522014 | 522114 |
| Marker62951 | LG4 | 529206 | 529156 | 529256 |
| Marker62965 | LG4 | 553318 | 553268 | 553368 |
| Marker62969 | LG4 | 565364 | 565314 | 565414 |
| Marker62986 | LG4 | 582341 | 582291 | 582391 |
| Marker62991 | LG4 | 590317 | 590267 | 590367 |
| Marker62993 | LG4 | 594353 | 594303 | 594403 |
| Marker63000 | LG4 | 605121 | 605071 | 605171 |
| Marker63031 | LG4 | 649039 | 648989 | 649089 |
| Marker63033 | LG4 | 649099 | 649049 | 649149 |
| Marker63050 | LG4 | 660463 | 660413 | 660513 |
| Marker63063 | LG4 | 684424 | 684374 | 684474 |
| Marker63121 | LG4 | 728782 | 728732 | 728832 |
| Marker63148 | LG4 | 754202 | 754152 | 754252 |
| Marker63149 | LG4 | 756270 | 756220 | 756320 |
| Marker63168 | LG4 | 779766 | 779716 | 779816 |
| Marker63169 | LG4 | 779953 | 779903 | 780003 |
| Marker63174 | LG4 | 783238 | 783188 | 783288 |
| Marker63181 | LG4 | 802663 | 802613 | 802713 |
| Marker61096 | LG4 | 878456 | 878406 | 878506 |
| Marker61102 | LG4 | 886817 | 886767 | 886867 |
| Marker61104 | LG4 | 887567 | 887517 | 887617 |
| Marker61117 | LG4 | 893538 | 893488 | 893588 |
| Marker61119 | LG4 | 897047 | 896997 | 897097 |
| Marker61120 | LG4 | 897293 | 897243 | 897343 |
| Marker61125 | LG4 | 900270 | 900220 | 900320 |
| Marker61126 | LG4 | 901070 | 901020 | 901120 |
| Marker61134 | LG4 | 903428 | 903378 | 903478 |
| Marker61138 | LG4 | 914332 | 914282 | 914382 |
| Marker61140 | LG4 | 915737 | 915687 | 915787 |
| Marker61162 | LG4 | 955842 | 955792 | 955892 |
| Marker61164 | LG4 | 957023 | 956973 | 957073 |
| Marker61269 | LG4 | 1074570 | 1074520 | 1074620 |
| Marker61323 | LG4 | 1188349 | 1188299 | 1188399 |
| Marker61339 | LG4 | 1212622 | 1212572 | 1212672 |
| Marker61342 | LG4 | 1212888 | 1212838 | 1212938 |
| Marker61344 | LG4 | 1215196 | 1215146 | 1215246 |
| Marker61365 | LG4 | 1240483 | 1240433 | 1240533 |
| Marker61377 | LG4 | 1243741 | 1243691 | 1243791 |
| Marker61391 | LG4 | 1276311 | 1276261 | 1276361 |
| Marker61392 | LG4 | 1281070 | 1281020 | 1281120 |
| Marker61407 | LG4 | 1296877 | 1296827 | 1296927 |
| Marker61416 | LG4 | 1316945 | 1316895 | 1316995 |
| Marker61422 | LG4 | 1324370 | 1324320 | 1324420 |
| Marker61455 | LG4 | 1381576 | 1381526 | 1381626 |
| Marker61456 | LG4 | 1381612 | 1381562 | 1381662 |
| Marker61457 | LG4 | 1382521 | 1382471 | 1382571 |
| Marker61479 | LG4 | 1393414 | 1393364 | 1393464 |
| Marker61481 | LG4 | 1394369 | 1394319 | 1394419 |
| Marker61510 | LG4 | 1432505 | 1432455 | 1432555 |
| Marker61515 | LG4 | 1439822 | 1439772 | 1439872 |
| Marker105258 | LG4 | 1456912 | 1456862 | 1456962 |
| Marker61535 | LG4 | 1461706 | 1461656 | 1461756 |
| Marker61547 | LG4 | 1473754 | 1473704 | 1473804 |
| Marker61555 | LG4 | 1475163 | 1475113 | 1475213 |
| Marker61557 | LG4 | 1475427 | 1475377 | 1475477 |
| Marker61571 | LG4 | 1499914 | 1499864 | 1499964 |
| Marker61572 | LG4 | 1500084 | 1500034 | 1500134 |
| Marker61573 | LG4 | 1502082 | 1502032 | 1502132 |
| Marker61586 | LG4 | 1512266 | 1512216 | 1512316 |
| Marker61587 | LG4 | 1519844 | 1519794 | 1519894 |
| Marker61591 | LG4 | 1525722 | 1525672 | 1525772 |
| Marker61612 | LG4 | 1542553 | 1542503 | 1542603 |
| Marker61622 | LG4 | 1554833 | 1554783 | 1554883 |
| Marker61651 | LG4 | 1612176 | 1612126 | 1612226 |
| Marker61657 | LG4 | 1626695 | 1626645 | 1626745 |
| Marker61659 | LG4 | 1626748 | 1626698 | 1626798 |
| Marker61682 | LG4 | 1661749 | 1661699 | 1661799 |
| Marker61688 | LG4 | 1673285 | 1673235 | 1673335 |
| Marker61691 | LG4 | 1678319 | 1678269 | 1678369 |
| Marker61699 | LG4 | 1697626 | 1697576 | 1697676 |
| Marker61701 | LG4 | 1704494 | 1704444 | 1704544 |
| Marker61731 | LG4 | 1765411 | 1765361 | 1765461 |
| Marker61732 | LG4 | 1765966 | 1765916 | 1766016 |
| Marker61767 | LG4 | 1797852 | 1797802 | 1797902 |
| Marker61770 | LG4 | 1806372 | 1806322 | 1806422 |
| Marker61772 | LG4 | 1806409 | 1806359 | 1806459 |
| Marker61804 | LG4 | 1872338 | 1872288 | 1872388 |
| Marker61811 | LG4 | 1879236 | 1879186 | 1879286 |
| Marker61816 | LG4 | 1880196 | 1880146 | 1880246 |
| Marker61856 | LG4 | 1926690 | 1926640 | 1926740 |
| Marker61861 | LG4 | 1932168 | 1932118 | 1932218 |
| Marker61871 | LG4 | 1953431 | 1953381 | 1953481 |
| Marker61872 | LG4 | 1957225 | 1957175 | 1957275 |
| Marker61876 | LG4 | 1959073 | 1959023 | 1959123 |
| Marker61884 | LG4 | 1964900 | 1964850 | 1964950 |
| Marker61895 | LG4 | 2005823 | 2005773 | 2005873 |
| Marker61906 | LG4 | 2013186 | 2013136 | 2013236 |
| Marker61907 | LG4 | 2014047 | 2013997 | 2014097 |
| Marker61912 | LG4 | 2018996 | 2018946 | 2019046 |
| Marker61914 | LG4 | 2025787 | 2025737 | 2025837 |
| Marker61920 | LG4 | 2046103 | 2046053 | 2046153 |
| Marker61961 | LG4 | 2113966 | 2113916 | 2114016 |
| Marker61973 | LG4 | 2119195 | 2119145 | 2119245 |
| Marker61980 | LG4 | 2134762 | 2134712 | 2134812 |
| Marker61986 | LG4 | 2152896 | 2152846 | 2152946 |
| Marker61987 | LG4 | 2153051 | 2153001 | 2153101 |
| Marker61989 | LG4 | 2154760 | 2154710 | 2154810 |
| Marker61991 | LG4 | 2155002 | 2154952 | 2155052 |
| Marker61995 | LG4 | 2168251 | 2168201 | 2168301 |
| Marker62000 | LG4 | 2172615 | 2172565 | 2172665 |
| Marker62008 | LG4 | 2184390 | 2184340 | 2184440 |
| Marker62016 | LG4 | 2201355 | 2201305 | 2201405 |
| Marker62018 | LG4 | 2206025 | 2205975 | 2206075 |
| Marker62020 | LG4 | 2211091 | 2211041 | 2211141 |
| Marker62044 | LG4 | 2254102 | 2254052 | 2254152 |
| Marker62052 | LG4 | 2261978 | 2261928 | 2262028 |
| Marker62055 | LG4 | 2267670 | 2267620 | 2267720 |
| Marker62065 | LG4 | 2279669 | 2279619 | 2279719 |
| Marker62066 | LG4 | 2288404 | 2288354 | 2288454 |
| Marker62067 | LG4 | 2288918 | 2288868 | 2288968 |
| Marker62069 | LG4 | 2290943 | 2290893 | 2290993 |
| Marker62073 | LG4 | 2296844 | 2296794 | 2296894 |
| Marker62076 | LG4 | 2300328 | 2300278 | 2300378 |
| Marker62081 | LG4 | 2304263 | 2304213 | 2304313 |
| Marker62098 | LG4 | 2322145 | 2322095 | 2322195 |
| Marker62104 | LG4 | 2329569 | 2329519 | 2329619 |
| Marker62105 | LG4 | 2330824 | 2330774 | 2330874 |
| Marker62114 | LG4 | 2358583 | 2358533 | 2358633 |
| Marker62115 | LG4 | 2358764 | 2358714 | 2358814 |
| Marker62125 | LG4 | 2367651 | 2367601 | 2367701 |
| Marker62135 | LG4 | 2375444 | 2375394 | 2375494 |
| Marker62139 | LG4 | 2378904 | 2378854 | 2378954 |
| Marker62148 | LG4 | 2390051 | 2390001 | 2390101 |
| Marker62197 | LG4 | 2429571 | 2429521 | 2429621 |
| Marker62199 | LG4 | 2429815 | 2429765 | 2429865 |
| Marker62206 | LG4 | 2438424 | 2438374 | 2438474 |
| Marker62210 | LG4 | 2445879 | 2445829 | 2445929 |
| Marker62220 | LG4 | 2457384 | 2457334 | 2457434 |
| Marker62221 | LG4 | 2457599 | 2457549 | 2457649 |
| Marker62224 | LG4 | 2461699 | 2461649 | 2461749 |
| Marker62230 | LG4 | 2479218 | 2479168 | 2479268 |
| Marker62244 | LG4 | 2488126 | 2488076 | 2488176 |
| Marker62252 | LG4 | 2496101 | 2496051 | 2496151 |
| Marker62258 | LG4 | 2516320 | 2516270 | 2516370 |
| Marker62275 | LG4 | 2546277 | 2546227 | 2546327 |
| Marker62284 | LG4 | 2562975 | 2562925 | 2563025 |
| Marker62290 | LG4 | 2573373 | 2573323 | 2573423 |
| Marker62315 | LG4 | 2612837 | 2612787 | 2612887 |
| Marker62316 | LG4 | 2612991 | 2612941 | 2613041 |
| Marker62318 | LG4 | 2617236 | 2617186 | 2617286 |
| Marker62327 | LG4 | 2627278 | 2627228 | 2627328 |
| Marker62340 | LG4 | 2642534 | 2642484 | 2642584 |
| Marker62341 | LG4 | 2642730 | 2642680 | 2642780 |
| Marker62343 | LG4 | 2643594 | 2643544 | 2643644 |
| Marker62358 | LG4 | 2656326 | 2656276 | 2656376 |
| Marker62366 | LG4 | 2660556 | 2660506 | 2660606 |
| Marker62384 | LG4 | 2684052 | 2684002 | 2684102 |
| Marker62408 | LG4 | 2775185 | 2775135 | 2775235 |
| Marker62491 | LG4 | 2864393 | 2864343 | 2864443 |
| Marker62502 | LG4 | 2910875 | 2910825 | 2910925 |
| Marker62506 | LG4 | 2913491 | 2913441 | 2913541 |
| Marker62528 | LG4 | 2938942 | 2938892 | 2938992 |
| Marker62539 | LG4 | 2968066 | 2968016 | 2968116 |
| Marker62541 | LG4 | 2970097 | 2970047 | 2970147 |
| Marker62557 | LG4 | 2989348 | 2989298 | 2989398 |
| Marker62583 | LG4 | 3031823 | 3031773 | 3031873 |
| Marker62591 | LG4 | 3038516 | 3038466 | 3038566 |
| Marker62597 | LG4 | 3040489 | 3040439 | 3040539 |
| Marker62599 | LG4 | 3044004 | 3043954 | 3044054 |
| Marker62602 | LG4 | 3067795 | 3067745 | 3067845 |
| Marker62606 | LG4 | 3071577 | 3071527 | 3071627 |
| Marker62614 | LG4 | 3083384 | 3083334 | 3083434 |
| Marker62620 | LG4 | 3139933 | 3139883 | 3139983 |
| Marker62621 | LG4 | 3145816 | 3145766 | 3145866 |
| Marker62663 | LG4 | 3220692 | 3220642 | 3220742 |
| Marker62692 | LG4 | 3278686 | 3278636 | 3278736 |
| Marker62706 | LG4 | 3294650 | 3294600 | 3294700 |
| Marker62786 | LG4 | 3407677 | 3407627 | 3407727 |
| Marker62787 | LG4 | 3408476 | 3408426 | 3408526 |
| Marker120789 | LG4 | 3418972 | 3418922 | 3419022 |
| Marker60530 | LG4 | 3603206 | 3603156 | 3603256 |
| Marker60537 | LG4 | 3615479 | 3615429 | 3615529 |
| Marker60543 | LG4 | 3616974 | 3616924 | 3617024 |
| Marker60544 | LG4 | 3619639 | 3619589 | 3619689 |
| Marker60565 | LG4 | 3650292 | 3650242 | 3650342 |
| Marker60573 | LG4 | 3658857 | 3658807 | 3658907 |
| Marker60576 | LG4 | 3660057 | 3660007 | 3660107 |
| Marker60580 | LG4 | 3667525 | 3667475 | 3667575 |
| Marker60587 | LG4 | 3675406 | 3675356 | 3675456 |
| Marker60598 | LG4 | 3696733 | 3696683 | 3696783 |
| Marker60600 | LG4 | 3699125 | 3699075 | 3699175 |
| Marker60623 | LG4 | 3733892 | 3733842 | 3733942 |
| Marker60630 | LG4 | 3748867 | 3748817 | 3748917 |
| Marker60637 | LG4 | 3755502 | 3755452 | 3755552 |
| Marker60643 | LG4 | 3768278 | 3768228 | 3768328 |
| Marker60659 | LG4 | 3789226 | 3789176 | 3789276 |
| Marker60678 | LG4 | 3835671 | 3835621 | 3835721 |
| Marker60682 | LG4 | 3836793 | 3836743 | 3836843 |
| Marker60710 | LG4 | 3848349 | 3848299 | 3848399 |
| Marker60711 | LG4 | 3848607 | 3848557 | 3848657 |
| Marker60723 | LG4 | 3858767 | 3858717 | 3858817 |
| Marker60727 | LG4 | 3866889 | 3866839 | 3866939 |
| Marker60761 | LG4 | 3927164 | 3927114 | 3927214 |
| Marker60765 | LG4 | 3928942 | 3928892 | 3928992 |
| Marker60772 | LG4 | 3942828 | 3942778 | 3942878 |
| Marker60782 | LG4 | 3946316 | 3946266 | 3946366 |
| Marker60803 | LG4 | 3974248 | 3974198 | 3974298 |
| Marker60815 | LG4 | 3998110 | 3998060 | 3998160 |
| Marker60819 | LG4 | 3998292 | 3998242 | 3998342 |
| Marker60832 | LG4 | 4040413 | 4040363 | 4040463 |
| Marker60834 | LG4 | 4049128 | 4049078 | 4049178 |
| Marker60836 | LG4 | 4057158 | 4057108 | 4057208 |
| Marker60840 | LG4 | 4061516 | 4061466 | 4061566 |
| Marker103361 | LG4 | 4151291 | 4151241 | 4151341 |
| Marker103319 | LG4 | 4176796 | 4176746 | 4176846 |
| Marker109347 | LG4 | 4214124 | 4214074 | 4214174 |
| Marker109342 | LG4 | 4217133 | 4217083 | 4217183 |
| Marker109252 | LG4 | 4373880 | 4373830 | 4373930 |
| Marker109250 | LG4 | 4379243 | 4379193 | 4379293 |
| Marker109246 | LG4 | 4393060 | 4393010 | 4393110 |
| Marker109234 | LG4 | 4408098 | 4408048 | 4408148 |
| Marker109229 | LG4 | 4411394 | 4411344 | 4411444 |
| Marker109201 | LG4 | 4462556 | 4462506 | 4462606 |
| Marker109197 | LG4 | 4471634 | 4471584 | 4471684 |
| Marker109191 | LG4 | 4472601 | 4472551 | 4472651 |
| Marker109148 | LG4 | 4639190 | 4639140 | 4639240 |
| Marker109145 | LG4 | 4639395 | 4639345 | 4639445 |
| Marker109139 | LG4 | 4640467 | 4640417 | 4640517 |
| Marker109123 | LG4 | 4656059 | 4656009 | 4656109 |
| Marker109113 | LG4 | 4663582 | 4663532 | 4663632 |
| Marker109099 | LG4 | 4703090 | 4703040 | 4703140 |
| Marker109086 | LG4 | 4739331 | 4739281 | 4739381 |
| Marker109084 | LG4 | 4747312 | 4747262 | 4747362 |
| Marker109078 | LG4 | 4761034 | 4760984 | 4761084 |
| Marker60915 | LG4 | 4829421 | 4829371 | 4829471 |
| Marker60912 | LG4 | 4832286 | 4832236 | 4832336 |
| Marker60907 | LG4 | 4834726 | 4834676 | 4834776 |
| Marker60114 | LG4 | 4932228 | 4932178 | 4932278 |
| Marker60115 | LG4 | 4932476 | 4932426 | 4932526 |
| Marker60132 | LG4 | 4978265 | 4978215 | 4978315 |
| Marker60180 | LG4 | 5079923 | 5079873 | 5079973 |
| Marker59832 | LG4 | 5177522 | 5177472 | 5177572 |
| Marker60268 | LG4 | 5345200 | 5345150 | 5345250 |
| Marker60332 | LG4 | 5520849 | 5520799 | 5520899 |
| Marker60334 | LG4 | 5521569 | 5521519 | 5521619 |
| Marker60345 | LG4 | 5530869 | 5530819 | 5530919 |
| Marker60350 | LG4 | 5534523 | 5534473 | 5534573 |
| Marker60374 | LG4 | 5599278 | 5599228 | 5599328 |
| Marker60376 | LG4 | 5600223 | 5600173 | 5600273 |
| Marker60380 | LG4 | 5604824 | 5604774 | 5604874 |
| Marker60382 | LG4 | 5604893 | 5604843 | 5604943 |
| Marker60421 | LG4 | 5706561 | 5706511 | 5706611 |
| Marker61009 | LG4 | 5776997 | 5776947 | 5777047 |
| Marker60988 | LG4 | 5935639 | 5935589 | 5935689 |
| Marker60987 | LG4 | 5936786 | 5936736 | 5936836 |
| Marker60985 | LG4 | 5943594 | 5943544 | 5943644 |
| Marker60099 | LG4 | 5974148 | 5974098 | 5974198 |
| Marker60085 | LG4 | 5997855 | 5997805 | 5997905 |
| Marker121426 | LG4 | 6056252 | 6056202 | 6056302 |
| Marker60082 | LG4 | 6075791 | 6075741 | 6075841 |
| Marker60075 | LG4 | 6106233 | 6106183 | 6106283 |
| Marker60004 | LG4 | 6184427 | 6184377 | 6184477 |
| Marker59992 | LG4 | 6218342 | 6218292 | 6218392 |
| Marker59991 | LG4 | 6218560 | 6218510 | 6218610 |
| Marker59960 | LG4 | 6259508 | 6259458 | 6259558 |
| Marker59954 | LG4 | 6269393 | 6269343 | 6269443 |
| Marker59931 | LG4 | 6287601 | 6287551 | 6287651 |
| Marker59926 | LG4 | 6298873 | 6298823 | 6298923 |
| Marker59905 | LG4 | 6315420 | 6315370 | 6315470 |
| Marker59874 | LG4 | 6361482 | 6361432 | 6361532 |
| Marker59857 | LG4 | 6385511 | 6385461 | 6385561 |
| Marker59518 | LG4 | 6400260 | 6400210 | 6400310 |
| Marker59543 | LG4 | 6459616 | 6459566 | 6459666 |
| Marker59544 | LG4 | 6471685 | 6471635 | 6471735 |
| Marker59568 | LG4 | 6532524 | 6532474 | 6532574 |
| Marker59601 | LG4 | 6553630 | 6553580 | 6553680 |
| Marker59604 | LG4 | 6557444 | 6557394 | 6557494 |
| Marker59611 | LG4 | 6566057 | 6566007 | 6566107 |
| Marker59614 | LG4 | 6569991 | 6569941 | 6570041 |
| Marker59617 | LG4 | 6574242 | 6574192 | 6574292 |
| Marker59622 | LG4 | 6587531 | 6587481 | 6587581 |
| Marker59638 | LG4 | 6608901 | 6608851 | 6608951 |
| Marker59639 | LG4 | 6608941 | 6608891 | 6608991 |
| Marker59659 | LG4 | 6624877 | 6624827 | 6624927 |
| Marker59663 | LG4 | 6634658 | 6634608 | 6634708 |
| Marker59745 | LG4 | 6712325 | 6712275 | 6712375 |
| Marker59754 | LG4 | 6739640 | 6739590 | 6739690 |
| Marker59760 | LG4 | 6741059 | 6741009 | 6741109 |
| Marker59025 | LG4 | 6858911 | 6858861 | 6858961 |
| Marker59028 | LG4 | 6862042 | 6861992 | 6862092 |
| Marker59034 | LG4 | 6885919 | 6885869 | 6885969 |
| Marker59037 | LG4 | 6891047 | 6890997 | 6891097 |
| Marker59039 | LG4 | 6891237 | 6891187 | 6891287 |
| Marker59049 | LG4 | 6900420 | 6900370 | 6900470 |
| Marker59060 | LG4 | 6915624 | 6915574 | 6915674 |
| Marker59070 | LG4 | 6931932 | 6931882 | 6931982 |
| Marker59076 | LG4 | 6939814 | 6939764 | 6939864 |
| Marker59085 | LG4 | 6964519 | 6964469 | 6964569 |
| Marker59089 | LG4 | 6969655 | 6969605 | 6969705 |
| Marker59112 | LG4 | 6996457 | 6996407 | 6996507 |
| Marker59129 | LG4 | 7028545 | 7028495 | 7028595 |
| Marker59130 | LG4 | 7031449 | 7031399 | 7031499 |
| Marker59143 | LG4 | 7053561 | 7053511 | 7053611 |
| Marker59146 | LG4 | 7054712 | 7054662 | 7054762 |
| Marker59156 | LG4 | 7066498 | 7066448 | 7066548 |
| Marker59179 | LG4 | 7112336 | 7112286 | 7112386 |
| Marker59201 | LG4 | 7167314 | 7167264 | 7167364 |
| Marker59222 | LG4 | 7190620 | 7190570 | 7190670 |
| Marker59226 | LG4 | 7204620 | 7204570 | 7204670 |
| Marker59257 | LG4 | 7233337 | 7233287 | 7233387 |
| Marker59283 | LG4 | 7277072 | 7277022 | 7277122 |
| Marker59314 | LG4 | 7297771 | 7297721 | 7297821 |
| Marker59322 | LG4 | 7310424 | 7310374 | 7310474 |
| Marker59323 | LG4 | 7310630 | 7310580 | 7310680 |
| Marker59328 | LG4 | 7315539 | 7315489 | 7315589 |
| Marker59370 | LG4 | 7415042 | 7414992 | 7415092 |
| Marker59427 | LG4 | 7442941 | 7442891 | 7442991 |
| Marker59434 | LG4 | 7445138 | 7445088 | 7445188 |
| Marker59448 | LG4 | 7454285 | 7454235 | 7454335 |
| Marker59449 | LG4 | 7454507 | 7454457 | 7454557 |
| Marker59453 | LG4 | 7457810 | 7457760 | 7457860 |
| Marker59460 | LG4 | 7464984 | 7464934 | 7465034 |
| Marker59478 | LG4 | 7486701 | 7486651 | 7486751 |
| Marker58999 | LG4 | 7533598 | 7533548 | 7533648 |
| Marker58997 | LG4 | 7533863 | 7533813 | 7533913 |
| Marker58990 | LG4 | 7566424 | 7566374 | 7566474 |
| Marker58989 | LG4 | 7570619 | 7570569 | 7570669 |
| Marker58976 | LG4 | 7587832 | 7587782 | 7587882 |
| Marker58971 | LG4 | 7594703 | 7594653 | 7594753 |
| Marker58928 | LG4 | 7620543 | 7620493 | 7620593 |
| Marker58927 | LG4 | 7620735 | 7620685 | 7620785 |
| Marker58925 | LG4 | 7629194 | 7629144 | 7629244 |
| Marker58913 | LG4 | 7675447 | 7675397 | 7675497 |
| Marker58893 | LG4 | 7720189 | 7720139 | 7720239 |
| Marker58887 | LG4 | 7745521 | 7745471 | 7745571 |
| Marker58860 | LG4 | 7787273 | 7787223 | 7787323 |
| Marker58834 | LG4 | 7875492 | 7875442 | 7875542 |
| Marker55620 | LG4 | 7982280 | 7982230 | 7982330 |
| Marker57870 | LG4 | 7994285 | 7994235 | 7994335 |
| Marker57891 | LG4 | 8254495 | 8254445 | 8254545 |
| Marker58748 | LG4 | 8276687 | 8276637 | 8276737 |
| Marker58732 | LG4 | 8300424 | 8300374 | 8300474 |
| Marker58722 | LG4 | 8314962 | 8314912 | 8315012 |
| Marker58721 | LG4 | 8315125 | 8315075 | 8315175 |
| Marker58700 | LG4 | 8349512 | 8349462 | 8349562 |
| Marker58664 | LG4 | 8447262 | 8447212 | 8447312 |
| Marker58653 | LG4 | 8496048 | 8495998 | 8496098 |
| Marker58647 | LG4 | 8526608 | 8526558 | 8526658 |
| Marker58611 | LG4 | 8600113 | 8600063 | 8600163 |
| Marker58600 | LG4 | 8603392 | 8603342 | 8603442 |
| Marker58599 | LG4 | 8605636 | 8605586 | 8605686 |
| Marker58598 | LG4 | 8605855 | 8605805 | 8605905 |
| Marker58591 | LG4 | 8622077 | 8622027 | 8622127 |
| Marker58588 | LG4 | 8634871 | 8634821 | 8634921 |
| Marker58569 | LG4 | 8664346 | 8664296 | 8664396 |
| Marker58494 | LG4 | 8773289 | 8773239 | 8773339 |
| Marker58476 | LG4 | 8793620 | 8793570 | 8793670 |
| Marker58473 | LG4 | 8799106 | 8799056 | 8799156 |
| Marker58462 | LG4 | 8822584 | 8822534 | 8822634 |
| Marker58459 | LG4 | 8826661 | 8826611 | 8826711 |
| Marker58457 | LG4 | 8835018 | 8834968 | 8835068 |
| Marker58447 | LG4 | 8858034 | 8857984 | 8858084 |
| Marker58445 | LG4 | 8858704 | 8858654 | 8858754 |
| Marker58441 | LG4 | 8860365 | 8860315 | 8860415 |
| Marker58439 | LG4 | 8860411 | 8860361 | 8860461 |
| Marker58421 | LG4 | 8870006 | 8869956 | 8870056 |
| Marker58417 | LG4 | 8893734 | 8893684 | 8893784 |
| Marker58412 | LG4 | 8895071 | 8895021 | 8895121 |
| Marker58388 | LG4 | 8919845 | 8919795 | 8919895 |
| Marker58351 | LG4 | 8960291 | 8960241 | 8960341 |
| Marker58348 | LG4 | 8972501 | 8972451 | 8972551 |
| Marker58342 | LG4 | 8983702 | 8983652 | 8983752 |
| Marker58337 | LG4 | 9006083 | 9006033 | 9006133 |
| Marker57834 | LG4 | 9148176 | 9148126 | 9148226 |
| Marker57739 | LG4 | 9206264 | 9206214 | 9206314 |
| Marker57746 | LG4 | 9211797 | 9211747 | 9211847 |
| Marker57797 | LG4 | 9269607 | 9269557 | 9269657 |
| Marker57638 | LG4 | 9367223 | 9367173 | 9367273 |
| Marker57640 | LG4 | 9367420 | 9367370 | 9367470 |
| Marker57693 | LG4 | 9435979 | 9435929 | 9436029 |
| Marker57709 | LG4 | 9468371 | 9468321 | 9468421 |
| Marker57732 | LG4 | 9515181 | 9515131 | 9515231 |
| Marker57736 | LG4 | 9524051 | 9524001 | 9524101 |
| Marker57572 | LG4 | 9676054 | 9676004 | 9676104 |
| Marker57574 | LG4 | 9677731 | 9677681 | 9677781 |
| Marker57578 | LG4 | 9678289 | 9678239 | 9678339 |
| Marker57591 | LG4 | 9693317 | 9693267 | 9693367 |
| Marker57595 | LG4 | 9701404 | 9701354 | 9701454 |
| Marker112330 | LG4 | 9817444 | 9817394 | 9817494 |
| Marker112327 | LG4 | 9826746 | 9826696 | 9826796 |
| Marker112309 | LG4 | 9899753 | 9899703 | 9899803 |
| Marker120775 | LG4 | 9943094 | 9943044 | 9943144 |
| Marker57460 | LG4 | 9989144 | 9989094 | 9989194 |
| Marker57505 | LG4 | 10010053 | 10010003 | 10010103 |
| Marker57508 | LG4 | 10018479 | 10018429 | 10018529 |
| Marker57517 | LG4 | 10028937 | 10028887 | 10028987 |
| Marker57090 | LG4 | 10094405 | 10094355 | 10094455 |
| Marker57169 | LG4 | 10215787 | 10215737 | 10215837 |
| Marker57175 | LG4 | 10219591 | 10219541 | 10219641 |
| Marker57180 | LG4 | 10227316 | 10227266 | 10227366 |
| Marker57186 | LG4 | 10250493 | 10250443 | 10250543 |
| Marker57191 | LG4 | 10264594 | 10264544 | 10264644 |
| Marker57193 | LG4 | 10267454 | 10267404 | 10267504 |
| Marker57194 | LG4 | 10268433 | 10268383 | 10268483 |
| Marker57202 | LG4 | 10275510 | 10275460 | 10275560 |
| Marker57207 | LG4 | 10279325 | 10279275 | 10279375 |
| Marker57221 | LG4 | 10285682 | 10285632 | 10285732 |
| Marker57223 | LG4 | 10289328 | 10289278 | 10289378 |
| Marker57225 | LG4 | 10290290 | 10290240 | 10290340 |
| Marker57227 | LG4 | 10290543 | 10290493 | 10290593 |
| Marker57235 | LG4 | 10296987 | 10296937 | 10297037 |
| Marker57241 | LG4 | 10306670 | 10306620 | 10306720 |
| Marker57256 | LG4 | 10349789 | 10349739 | 10349839 |
| Marker57268 | LG4 | 10360706 | 10360656 | 10360756 |
| Marker57304 | LG4 | 10374532 | 10374482 | 10374582 |
| Marker57322 | LG4 | 10419378 | 10419328 | 10419428 |
| Marker57333 | LG4 | 10451603 | 10451553 | 10451653 |
| Marker57335 | LG4 | 10456766 | 10456716 | 10456816 |
| Marker57368 | LG4 | 10474502 | 10474452 | 10474552 |
| Marker57411 | LG4 | 10512800 | 10512750 | 10512850 |
| Marker57416 | LG4 | 10525433 | 10525383 | 10525483 |
| Marker57419 | LG4 | 10527938 | 10527888 | 10527988 |
| Marker117055 | LG4 | 10581428 | 10581378 | 10581478 |
| Marker117054 | LG4 | 10582842 | 10582792 | 10582892 |
| Marker117051 | LG4 | 10583079 | 10583029 | 10583129 |
| Marker117035 | LG4 | 10594508 | 10594458 | 10594558 |
| Marker116994 | LG4 | 10622384 | 10622334 | 10622434 |
| Marker56822 | LG4 | 10693616 | 10693566 | 10693666 |
| Marker56820 | LG4 | 10693845 | 10693795 | 10693895 |
| Marker56794 | LG4 | 10822928 | 10822878 | 10822978 |
| Marker56728 | LG4 | 10931725 | 10931675 | 10931775 |
| Marker56723 | LG4 | 10937312 | 10937262 | 10937362 |
| Marker56644 | LG4 | 11140241 | 11140191 | 11140291 |
| Marker56610 | LG4 | 11157452 | 11157402 | 11157502 |
| Marker56595 | LG4 | 11182164 | 11182114 | 11182214 |
| Marker56594 | LG4 | 11182674 | 11182624 | 11182724 |
| Marker56593 | LG4 | 11184718 | 11184668 | 11184768 |
| Marker112369 | LG4 | 11351469 | 11351419 | 11351519 |
| Marker112343 | LG4 | 11431514 | 11431464 | 11431564 |
| Marker57083 | LG4 | 11490770 | 11490720 | 11490820 |
| Marker57070 | LG4 | 11528169 | 11528119 | 11528219 |
| Marker57055 | LG4 | 11532531 | 11532481 | 11532581 |
| Marker57040 | LG4 | 11554839 | 11554789 | 11554889 |
| Marker18559 | LG4 | 11592606 | 11592556 | 11592656 |
| Marker57010 | LG4 | 11620241 | 11620191 | 11620291 |
| Marker56970 | LG4 | 11656735 | 11656685 | 11656785 |
| Marker56955 | LG4 | 11674882 | 11674832 | 11674932 |
| Marker56942 | LG4 | 11705937 | 11705887 | 11705987 |
| Marker113767 | LG4 | 11825762 | 11825712 | 11825812 |
| Marker113757 | LG4 | 11835466 | 11835416 | 11835516 |
| Marker113749 | LG4 | 11850368 | 11850318 | 11850418 |
| Marker113721 | LG4 | 11889304 | 11889254 | 11889354 |
| Marker113632 | LG4 | 12004268 | 12004218 | 12004318 |
| Marker113625 | LG4 | 12042782 | 12042732 | 12042832 |
| Marker113529 | LG4 | 12133767 | 12133717 | 12133817 |
| Marker56420 | LG4 | 12193405 | 12193355 | 12193455 |
| Marker16059 | LG4 | 12732763 | 12732713 | 12732813 |
| Marker56545 | LG4 | 12767809 | 12767759 | 12767859 |
| Marker56533 | LG4 | 12826041 | 12825991 | 12826091 |
| Marker56266 | LG4 | 12935721 | 12935671 | 12935771 |
| Marker56313 | LG4 | 13000617 | 13000567 | 13000667 |
| Marker56345 | LG4 | 13055317 | 13055267 | 13055367 |
| Marker56348 | LG4 | 13056027 | 13055977 | 13056077 |
| Marker56360 | LG4 | 13066411 | 13066361 | 13066461 |
| Marker55750 | LG4 | 13085206 | 13085156 | 13085256 |
| Marker55758 | LG4 | 13091218 | 13091168 | 13091268 |
| Marker55769 | LG4 | 13130419 | 13130369 | 13130469 |
| Marker55778 | LG4 | 13146164 | 13146114 | 13146214 |
| Marker55787 | LG4 | 13147971 | 13147921 | 13148021 |
| Marker55792 | LG4 | 13150403 | 13150353 | 13150453 |
| Marker55843 | LG4 | 13205496 | 13205446 | 13205546 |
| Marker55899 | LG4 | 13527152 | 13527102 | 13527202 |
| Marker57020 | LG4 | 13540155 | 13540105 | 13540205 |
| Marker55246 | LG4 | 13611272 | 13611222 | 13611322 |
| Marker55248 | LG4 | 13611539 | 13611489 | 13611589 |
| Marker55253 | LG4 | 13614756 | 13614706 | 13614806 |
| Marker55257 | LG4 | 13624711 | 13624661 | 13624761 |
| Marker55260 | LG4 | 13627657 | 13627607 | 13627707 |
| Marker55280 | LG4 | 13659835 | 13659785 | 13659885 |
| Marker55536 | LG4 | 13730269 | 13730219 | 13730319 |
| Marker55532 | LG4 | 13731161 | 13731111 | 13731211 |
| Marker55525 | LG4 | 13735027 | 13734977 | 13735077 |
| Marker55506 | LG4 | 13883296 | 13883246 | 13883346 |
| Marker55501 | LG4 | 13890539 | 13890489 | 13890589 |
| Marker55474 | LG4 | 13908582 | 13908532 | 13908632 |
| Marker55462 | LG4 | 13933019 | 13932969 | 13933069 |
| Marker55425 | LG4 | 13954106 | 13954056 | 13954156 |
| Marker55415 | LG4 | 14000392 | 14000342 | 14000442 |
| Marker55290 | LG4 | 14115615 | 14115565 | 14115665 |
| Marker55298 | LG4 | 14136329 | 14136279 | 14136379 |
| Marker55341 | LG4 | 14196293 | 14196243 | 14196343 |
| Marker56022 | LG4 | 14347744 | 14347694 | 14347794 |
| Marker56082 | LG4 | 14385469 | 14385419 | 14385519 |
| Marker56084 | LG4 | 14395382 | 14395332 | 14395432 |
| Marker56092 | LG4 | 14405576 | 14405526 | 14405626 |
| Marker56102 | LG4 | 14418952 | 14418902 | 14419002 |
| Marker56126 | LG4 | 14464178 | 14464128 | 14464228 |
| Marker54817 | LG4 | 14561266 | 14561216 | 14561316 |
| Marker54830 | LG4 | 14633250 | 14633200 | 14633300 |
| Marker54835 | LG4 | 14646209 | 14646159 | 14646259 |
| Marker54848 | LG4 | 14678328 | 14678278 | 14678378 |
| Marker55551 | LG4 | 14742604 | 14742554 | 14742654 |
| Marker55419 | LG4 | 14752549 | 14752499 | 14752599 |
| Marker55561 | LG4 | 14758397 | 14758347 | 14758447 |
| Marker55577 | LG4 | 14778078 | 14778028 | 14778128 |
| Marker55666 | LG4 | 14827784 | 14827734 | 14827834 |
| Marker56071 | LG4 | 15307301 | 15307251 | 15307351 |
| Marker115109 | LG4 | 15403200 | 15403150 | 15403250 |
| Marker115079 | LG4 | 15431260 | 15431210 | 15431310 |
| Marker115043 | LG4 | 15471434 | 15471384 | 15471484 |
| Marker113347 | LG4 | 15514459 | 15514409 | 15514509 |
| Marker113327 | LG4 | 15544866 | 15544816 | 15544916 |
| Marker113300 | LG4 | 15597740 | 15597690 | 15597790 |
| Marker113291 | LG4 | 15612048 | 15611998 | 15612098 |
| Marker113267 | LG4 | 15686305 | 15686255 | 15686355 |
| Marker113253 | LG4 | 15702463 | 15702413 | 15702513 |
| Marker113252 | LG4 | 15702525 | 15702475 | 15702575 |
| Marker113245 | LG4 | 15705307 | 15705257 | 15705357 |
| Marker113227 | LG4 | 15729586 | 15729536 | 15729636 |
| Marker113224 | LG4 | 15730133 | 15730083 | 15730183 |
| Marker54789 | LG4 | 15739917 | 15739867 | 15739967 |
| Marker54759 | LG4 | 15834832 | 15834782 | 15834882 |
| Marker54756 | LG4 | 15835109 | 15835059 | 15835159 |
| Marker54675 | LG4 | 15941712 | 15941662 | 15941762 |
| Marker117403 | LG4 | 16026170 | 16026120 | 16026220 |
| Marker116764 | LG4 | 16038187 | 16038137 | 16038237 |
| Marker55947 | LG4 | 16094201 | 16094151 | 16094251 |
| Marker55949 | LG4 | 16098400 | 16098350 | 16098450 |
| Marker55969 | LG4 | 16120048 | 16119998 | 16120098 |
| Marker55971 | LG4 | 16121544 | 16121494 | 16121594 |
| Marker56191 | LG4 | 16256590 | 16256540 | 16256640 |
| Marker56200 | LG4 | 16291007 | 16290957 | 16291057 |
| Marker56237 | LG4 | 16334969 | 16334919 | 16335019 |
| Marker244 | LG4 | 16356986 | 16356936 | 16357036 |
| Marker113465 | LG4 | 16359162 | 16359112 | 16359212 |
| Marker113415 | LG4 | 16399158 | 16399108 | 16399208 |
| Marker54592 | LG4 | 16525422 | 16525372 | 16525472 |
| Marker25876 | LG4 | 16591405 | 16591355 | 16591455 |
| Marker54646 | LG4 | 16732919 | 16732869 | 16732969 |
| Marker54663 | LG4 | 16742251 | 16742201 | 16742301 |
| Marker55130 | LG4 | 16800767 | 16800717 | 16800817 |
| Marker55101 | LG4 | 16817669 | 16817619 | 16817719 |
| Marker55052 | LG4 | 16951634 | 16951584 | 16951684 |
| Marker55051 | LG4 | 16955724 | 16955674 | 16955774 |
| Marker55048 | LG4 | 16955899 | 16955849 | 16955949 |
| Marker54531 | LG4 | 17101673 | 17101623 | 17101723 |
| Marker54513 | LG4 | 17124276 | 17124226 | 17124326 |
| Marker54491 | LG4 | 17146924 | 17146874 | 17146974 |
| Marker54475 | LG4 | 17174054 | 17174004 | 17174104 |
| Marker54465 | LG4 | 17213485 | 17213435 | 17213535 |
| Marker54443 | LG4 | 17228004 | 17227954 | 17228054 |
| Marker54426 | LG4 | 17243606 | 17243556 | 17243656 |
| Marker54420 | LG4 | 17243835 | 17243785 | 17243885 |
| Marker54359 | LG4 | 17314998 | 17314948 | 17315048 |
| Marker54338 | LG4 | 17321552 | 17321502 | 17321602 |
| Marker54336 | LG4 | 17323407 | 17323357 | 17323457 |
| Marker54334 | LG4 | 17325893 | 17325843 | 17325943 |
| Marker54332 | LG4 | 17333746 | 17333696 | 17333796 |
| Marker54328 | LG4 | 17336179 | 17336129 | 17336229 |
| Marker54326 | LG4 | 17338203 | 17338153 | 17338253 |
| Marker54320 | LG4 | 17344414 | 17344364 | 17344464 |
| Marker54293 | LG4 | 17403539 | 17403489 | 17403589 |
| Marker54280 | LG4 | 17429687 | 17429637 | 17429737 |
| Marker54279 | LG4 | 17429732 | 17429682 | 17429782 |
| Marker54274 | LG4 | 17440141 | 17440091 | 17440191 |
| Marker54272 | LG4 | 17443713 | 17443663 | 17443763 |
| Marker54267 | LG4 | 17463359 | 17463309 | 17463409 |
| Marker54248 | LG4 | 17502014 | 17501964 | 17502064 |
| Marker54244 | LG4 | 17506887 | 17506837 | 17506937 |
| Marker54242 | LG4 | 17507952 | 17507902 | 17508002 |
| Marker54239 | LG4 | 17515077 | 17515027 | 17515127 |
| Marker54213 | LG4 | 17589789 | 17589739 | 17589839 |
| Marker54208 | LG4 | 17599075 | 17599025 | 17599125 |
| Marker54206 | LG4 | 17602748 | 17602698 | 17602798 |
| Marker54199 | LG4 | 17607842 | 17607792 | 17607892 |
| Marker54196 | LG4 | 17612615 | 17612565 | 17612665 |
| Marker54122 | LG4 | 17829850 | 17829800 | 17829900 |
| Marker54112 | LG4 | 17882537 | 17882487 | 17882587 |
| Marker54064 | LG4 | 17943028 | 17942978 | 17943078 |
| Marker73527 | LG4 | 17967217 | 17967167 | 17967267 |
| Marker54048 | LG4 | 17985213 | 17985163 | 17985263 |
| Marker54044 | LG4 | 17990200 | 17990150 | 17990250 |
| Marker54040 | LG4 | 17997109 | 17997059 | 17997159 |
| Marker53934 | LG4 | 18101190 | 18101140 | 18101240 |
| Marker53895 | LG4 | 18187800 | 18187750 | 18187850 |
| Marker53891 | LG4 | 18189195 | 18189145 | 18189245 |
| Marker53885 | LG4 | 18208272 | 18208222 | 18208322 |
| Marker53865 | LG4 | 18248029 | 18247979 | 18248079 |
| Marker53861 | LG4 | 18264223 | 18264173 | 18264273 |
| Marker53843 | LG4 | 18295756 | 18295706 | 18295806 |
| Marker53841 | LG4 | 18296414 | 18296364 | 18296464 |
| Marker53832 | LG4 | 18302388 | 18302338 | 18302438 |
| Marker53825 | LG4 | 18305092 | 18305042 | 18305142 |
| Marker53824 | LG4 | 18305103 | 18305053 | 18305153 |
| Marker53790 | LG4 | 18410360 | 18410310 | 18410410 |
| Marker53789 | LG4 | 18410524 | 18410474 | 18410574 |
| Marker53740 | LG4 | 18461345 | 18461295 | 18461395 |
| Marker53709 | LG4 | 18520916 | 18520866 | 18520966 |
| Marker53705 | LG4 | 18521481 | 18521431 | 18521531 |
| Marker53702 | LG4 | 18523785 | 18523735 | 18523835 |
| Marker53698 | LG4 | 18525579 | 18525529 | 18525629 |
| Marker53690 | LG4 | 18545726 | 18545676 | 18545776 |
| Marker53679 | LG4 | 18560854 | 18560804 | 18560904 |
| Marker53674 | LG4 | 18565628 | 18565578 | 18565678 |
| Marker53672 | LG4 | 18568732 | 18568682 | 18568782 |
| Marker53334 | LG4 | 18588066 | 18588016 | 18588116 |
| Marker53336 | LG4 | 18595059 | 18595009 | 18595109 |
| Marker53346 | LG4 | 18602623 | 18602573 | 18602673 |
| Marker53360 | LG4 | 18607232 | 18607182 | 18607282 |
| Marker53378 | LG4 | 18611518 | 18611468 | 18611568 |
| Marker53382 | LG4 | 18615466 | 18615416 | 18615516 |
| Marker53449 | LG4 | 18701322 | 18701272 | 18701372 |
| Marker53661 | LG4 | 18725230 | 18725180 | 18725280 |
| Marker53657 | LG4 | 18725304 | 18725254 | 18725354 |
| Marker53619 | LG4 | 18745516 | 18745466 | 18745566 |
| Marker53600 | LG4 | 18766350 | 18766300 | 18766400 |
| Marker53546 | LG4 | 18862687 | 18862637 | 18862737 |
| Marker53543 | LG4 | 18863153 | 18863103 | 18863203 |
| Marker53540 | LG4 | 18863719 | 18863669 | 18863769 |
| Marker53509 | LG4 | 18973771 | 18973721 | 18973821 |
| Marker53505 | LG4 | 18979582 | 18979532 | 18979632 |
| Marker53503 | LG4 | 18980292 | 18980242 | 18980342 |
| Marker53499 | LG4 | 18982184 | 18982134 | 18982234 |
| Marker53477 | LG4 | 19028772 | 19028722 | 19028822 |
| Marker53476 | LG4 | 19035353 | 19035303 | 19035403 |
| Marker53452 | LG4 | 19056676 | 19056626 | 19056726 |
| Marker110006 | LG4 | 19170029 | 19169979 | 19170079 |
| Marker110004 | LG4 | 19170283 | 19170233 | 19170333 |
| Marker110002 | LG4 | 19182074 | 19182024 | 19182124 |
| Marker110001 | LG4 | 19185977 | 19185927 | 19186027 |
| Marker109937 | LG4 | 19258917 | 19258867 | 19258967 |
| Marker109907 | LG4 | 19299188 | 19299138 | 19299238 |
| Marker109858 | LG4 | 19345268 | 19345218 | 19345318 |
| Marker109848 | LG4 | 19352713 | 19352663 | 19352763 |
| Marker109800 | LG4 | 19424440 | 19424390 | 19424490 |
| Marker109780 | LG4 | 19466248 | 19466198 | 19466298 |
| Marker53292 | LG4 | 19482692 | 19482642 | 19482742 |
| Marker53291 | LG4 | 19497427 | 19497377 | 19497477 |
| Marker53269 | LG4 | 19520958 | 19520908 | 19521008 |
| Marker23634 | LG4 | 19550428 | 19550378 | 19550478 |
| Marker53255 | LG4 | 19560812 | 19560762 | 19560862 |
| Marker53199 | LG4 | 19598957 | 19598907 | 19599007 |
| Marker53192 | LG4 | 19600776 | 19600726 | 19600826 |
| Marker53140 | LG4 | 19649684 | 19649634 | 19649734 |
| Marker53126 | LG4 | 19661503 | 19661453 | 19661553 |
| Marker53118 | LG4 | 19672583 | 19672533 | 19672633 |
| Marker53112 | LG4 | 19677915 | 19677865 | 19677965 |
| Marker53093 | LG4 | 19712879 | 19712829 | 19712929 |
| Marker53080 | LG4 | 19735760 | 19735710 | 19735810 |
| Marker53075 | LG4 | 19739016 | 19738966 | 19739066 |
| Marker53027 | LG4 | 19800551 | 19800501 | 19800601 |
| Marker53022 | LG4 | 19801367 | 19801317 | 19801417 |
| Marker52981 | LG4 | 19827887 | 19827837 | 19827937 |
| Marker52967 | LG4 | 19833594 | 19833544 | 19833644 |
| Marker52959 | LG4 | 19847680 | 19847630 | 19847730 |
| Marker52926 | LG4 | 19891048 | 19890998 | 19891098 |
| Marker52891 | LG4 | 19947068 | 19947018 | 19947118 |
| Marker52879 | LG4 | 19976167 | 19976117 | 19976217 |
| Marker52878 | LG4 | 19976419 | 19976369 | 19976469 |
| Marker52828 | LG4 | 20059844 | 20059794 | 20059894 |
| Marker52827 | LG4 | 20060088 | 20060038 | 20060138 |
| Marker52808 | LG4 | 20086413 | 20086363 | 20086463 |
| Marker52807 | LG4 | 20088227 | 20088177 | 20088277 |
| Marker52796 | LG4 | 20108858 | 20108808 | 20108908 |
| Marker52472 | LG4 | 20135429 | 20135379 | 20135479 |
| Marker52474 | LG4 | 20138384 | 20138334 | 20138434 |
| Marker52485 | LG4 | 20153647 | 20153597 | 20153697 |
| Marker52506 | LG4 | 20161782 | 20161732 | 20161832 |
| Marker52538 | LG4 | 20179312 | 20179262 | 20179362 |
| Marker52540 | LG4 | 20181135 | 20181085 | 20181185 |
| Marker52546 | LG4 | 20187433 | 20187383 | 20187483 |
| Marker52548 | LG4 | 20187624 | 20187574 | 20187674 |
| Marker52553 | LG4 | 20189224 | 20189174 | 20189274 |
| Marker52559 | LG4 | 20189491 | 20189441 | 20189541 |
| Marker52588 | LG4 | 20210023 | 20209973 | 20210073 |
| Marker52599 | LG4 | 20215491 | 20215441 | 20215541 |
| Marker52645 | LG4 | 20273724 | 20273674 | 20273774 |
| Marker52652 | LG4 | 20293555 | 20293505 | 20293605 |
| Marker52653 | LG4 | 20295624 | 20295574 | 20295674 |
| Marker52660 | LG4 | 20301288 | 20301238 | 20301338 |
| Marker52675 | LG4 | 20303500 | 20303450 | 20303550 |
| Marker52685 | LG4 | 20306182 | 20306132 | 20306232 |
| Marker52697 | LG4 | 20325436 | 20325386 | 20325486 |
| Marker52698 | LG4 | 20325586 | 20325536 | 20325636 |
| Marker52705 | LG4 | 20333899 | 20333849 | 20333949 |
| Marker52740 | LG4 | 20352137 | 20352087 | 20352187 |
| Marker52743 | LG4 | 20371617 | 20371567 | 20371667 |
| Marker52767 | LG4 | 20400407 | 20400357 | 20400457 |
| Marker52778 | LG4 | 20442823 | 20442773 | 20442873 |
| Marker52431 | LG4 | 20654853 | 20654803 | 20654903 |
| Marker52427 | LG4 | 20656816 | 20656766 | 20656866 |
| Marker65554 | LG4 | 20699697 | 20699647 | 20699747 |
| Marker119440 | LG4 | 20734693 | 20734643 | 20734743 |
| Marker65520 | LG4 | 20753087 | 20753037 | 20753137 |
| Marker65521 | LG4 | 20754436 | 20754386 | 20754486 |
| Marker65522 | LG4 | 20758273 | 20758223 | 20758323 |
| Marker64728 | LG4 | 20787833 | 20787783 | 20787883 |
| Marker64741 | LG4 | 20805257 | 20805207 | 20805307 |
| Marker64770 | LG4 | 20855496 | 20855446 | 20855546 |
| Marker64795 | LG4 | 20874936 | 20874886 | 20874986 |
| Marker64824 | LG4 | 20969621 | 20969571 | 20969671 |
| Marker64855 | LG4 | 20993453 | 20993403 | 20993503 |
| Marker64859 | LG4 | 20995717 | 20995667 | 20995767 |
| Marker64860 | LG4 | 20995990 | 20995940 | 20996040 |
| Marker64924 | LG4 | 21071097 | 21071047 | 21071147 |
| Marker64936 | LG4 | 21098000 | 21097950 | 21098050 |
| Marker52363 | LG4 | 21185937 | 21185887 | 21185987 |
| Marker52392 | LG4 | 21229266 | 21229216 | 21229316 |
| Marker52399 | LG4 | 21248189 | 21248139 | 21248239 |
| Marker52402 | LG4 | 21256658 | 21256608 | 21256708 |
| Marker52353 | LG4 | 21373738 | 21373688 | 21373788 |
| Marker52346 | LG4 | 21382642 | 21382592 | 21382692 |
| Marker52344 | LG4 | 21387428 | 21387378 | 21387478 |
| Marker52336 | LG4 | 21409458 | 21409408 | 21409508 |
| Marker52322 | LG4 | 21420746 | 21420696 | 21420796 |
| Marker52318 | LG4 | 21423144 | 21423094 | 21423194 |
| Marker52309 | LG4 | 21424167 | 21424117 | 21424217 |
| Marker52285 | LG4 | 21440952 | 21440902 | 21441002 |
| Marker52279 | LG4 | 21442850 | 21442800 | 21442900 |
| Marker52274 | LG4 | 21446000 | 21445950 | 21446050 |
| Marker52273 | LG4 | 21448113 | 21448063 | 21448163 |
| Marker52257 | LG4 | 21455162 | 21455112 | 21455212 |
| Marker52261 | LG4 | 21476701 | 21476651 | 21476751 |
| Marker52241 | LG4 | 21495020 | 21494970 | 21495070 |
| Marker52236 | LG4 | 21510345 | 21510295 | 21510395 |
| Marker52228 | LG4 | 21522997 | 21522947 | 21523047 |
| Marker52221 | LG4 | 21546382 | 21546332 | 21546432 |
| Marker52220 | LG4 | 21546394 | 21546344 | 21546444 |
| Marker52214 | LG4 | 21557849 | 21557799 | 21557899 |
| Marker29516 | LG4 | 21568855 | 21568805 | 21568905 |
| Marker52202 | LG4 | 21575678 | 21575628 | 21575728 |
| Marker52182 | LG4 | 21582659 | 21582609 | 21582709 |
| Marker52174 | LG4 | 21604102 | 21604052 | 21604152 |
| Marker52154 | LG4 | 21634293 | 21634243 | 21634343 |
| Marker52152 | LG4 | 21643827 | 21643777 | 21643877 |
| Marker52148 | LG4 | 21650556 | 21650506 | 21650606 |
| Marker52128 | LG4 | 21690195 | 21690145 | 21690245 |
| Marker52116 | LG4 | 21694852 | 21694802 | 21694902 |
| Marker52105 | LG4 | 21708752 | 21708702 | 21708802 |
| Marker52096 | LG4 | 21711939 | 21711889 | 21711989 |
| Marker52077 | LG4 | 21748793 | 21748743 | 21748843 |
| Marker114574 | LG4 | 21969321 | 21969271 | 21969371 |
| Marker114583 | LG4 | 21981986 | 21981936 | 21982036 |
| Marker114586 | LG4 | 21983186 | 21983136 | 21983236 |
| Marker114611 | LG4 | 22020374 | 22020324 | 22020424 |
| Marker114613 | LG4 | 22037098 | 22037048 | 22037148 |
| Marker52072 | LG4 | 22095465 | 22095415 | 22095515 |
| Marker52055 | LG4 | 22119836 | 22119786 | 22119886 |
| Marker52027 | LG4 | 22157708 | 22157658 | 22157758 |
| Marker52014 | LG4 | 22170673 | 22170623 | 22170723 |
| Marker52008 | LG4 | 22174334 | 22174284 | 22174384 |
| Marker52003 | LG4 | 22179539 | 22179489 | 22179589 |
| Marker51968 | LG4 | 22222104 | 22222054 | 22222154 |
| Marker51967 | LG4 | 22222133 | 22222083 | 22222183 |
| Marker51966 | LG4 | 22223059 | 22223009 | 22223109 |
| Marker51949 | LG4 | 22253945 | 22253895 | 22253995 |
| Marker51923 | LG4 | 22270429 | 22270379 | 22270479 |
| Marker51913 | LG4 | 22280607 | 22280557 | 22280657 |
| Marker49737 | LG4 | 22305084 | 22305034 | 22305134 |
| Marker51838 | LG4 | 22332286 | 22332236 | 22332336 |
| Marker51835 | LG4 | 22332525 | 22332475 | 22332575 |
| Marker51785 | LG4 | 22460162 | 22460112 | 22460212 |
| Marker51775 | LG4 | 22496550 | 22496500 | 22496600 |
| Marker51720 | LG4 | 22537912 | 22537862 | 22537962 |
| Marker51716 | LG4 | 22544552 | 22544502 | 22544602 |
| Marker51712 | LG4 | 22570488 | 22570438 | 22570538 |
| Marker51676 | LG4 | 22615173 | 22615123 | 22615223 |
| Marker51655 | LG4 | 22632768 | 22632718 | 22632818 |
| Marker51637 | LG4 | 22642765 | 22642715 | 22642815 |
| Marker51636 | LG4 | 22648001 | 22647951 | 22648051 |
| Marker51633 | LG4 | 22649300 | 22649250 | 22649350 |
| Marker51625 | LG4 | 22661829 | 22661779 | 22661879 |
| Marker51620 | LG4 | 22717670 | 22717620 | 22717720 |
| Marker51616 | LG4 | 22717900 | 22717850 | 22717950 |
| Marker51599 | LG4 | 22741445 | 22741395 | 22741495 |
| Marker51593 | LG4 | 22757102 | 22757052 | 22757152 |
| Marker51536 | LG4 | 22844340 | 22844290 | 22844390 |
| Marker51533 | LG4 | 22844613 | 22844563 | 22844663 |
| Marker51521 | LG4 | 22866637 | 22866587 | 22866687 |
| Marker51448 | LG4 | 23033708 | 23033658 | 23033758 |
| Marker51441 | LG4 | 23039389 | 23039339 | 23039439 |
| Marker51440 | LG4 | 23039590 | 23039540 | 23039640 |
| Marker51433 | LG4 | 23047335 | 23047285 | 23047385 |
| Marker51431 | LG4 | 23049064 | 23049014 | 23049114 |
| Marker51422 | LG4 | 23068395 | 23068345 | 23068445 |
| Marker51360 | LG4 | 23205502 | 23205452 | 23205552 |
| Marker51352 | LG4 | 23224815 | 23224765 | 23224865 |
| Marker51348 | LG4 | 23233586 | 23233536 | 23233636 |
| Marker51342 | LG4 | 23255884 | 23255834 | 23255934 |
| Marker51327 | LG4 | 23268817 | 23268767 | 23268867 |
| Marker51316 | LG4 | 23277665 | 23277615 | 23277715 |
| Marker51299 | LG4 | 23323027 | 23322977 | 23323077 |
| Marker51286 | LG4 | 23334128 | 23334078 | 23334178 |
| Marker51285 | LG4 | 23334189 | 23334139 | 23334239 |
| Marker51275 | LG4 | 23336945 | 23336895 | 23336995 |
| Marker51270 | LG4 | 23337816 | 23337766 | 23337866 |
| Marker51257 | LG4 | 23339260 | 23339210 | 23339310 |
| Marker51242 | LG4 | 23354331 | 23354281 | 23354381 |
| Marker51238 | LG4 | 23359127 | 23359077 | 23359177 |
| Marker51218 | LG4 | 23393618 | 23393568 | 23393668 |
| Marker51217 | LG4 | 23393845 | 23393795 | 23393895 |
| Marker51216 | LG4 | 23397892 | 23397842 | 23397942 |
| Marker51215 | LG4 | 23399944 | 23399894 | 23399994 |
| Marker51212 | LG4 | 23420963 | 23420913 | 23421013 |
| Marker51170 | LG4 | 23454598 | 23454548 | 23454648 |
| Marker51147 | LG4 | 23482787 | 23482737 | 23482837 |
| Marker51144 | LG4 | 23483079 | 23483029 | 23483129 |
| Marker51131 | LG4 | 23513964 | 23513914 | 23514014 |
| Marker51129 | LG4 | 23516583 | 23516533 | 23516633 |
| Marker51123 | LG4 | 23531911 | 23531861 | 23531961 |
| Marker51065 | LG4 | 23570229 | 23570179 | 23570279 |
| Marker51054 | LG4 | 23598250 | 23598200 | 23598300 |
| Marker51045 | LG4 | 23605490 | 23605440 | 23605540 |
| Marker51036 | LG4 | 23635014 | 23634964 | 23635064 |
| Marker51031 | LG4 | 23636759 | 23636709 | 23636809 |
| Marker51028 | LG4 | 23639810 | 23639760 | 23639860 |
| Marker51003 | LG4 | 23649712 | 23649662 | 23649762 |
| Marker50936 | LG4 | 23725533 | 23725483 | 23725583 |
| Marker50378 | LG4 | 23820651 | 23820601 | 23820701 |
| Marker50381 | LG4 | 23820879 | 23820829 | 23820929 |
| Marker50385 | LG4 | 23837802 | 23837752 | 23837852 |
| Marker50466 | LG4 | 23946600 | 23946550 | 23946650 |
| Marker50467 | LG4 | 23954117 | 23954067 | 23954167 |
| Marker50469 | LG4 | 23954345 | 23954295 | 23954395 |
| Marker50479 | LG4 | 23972481 | 23972431 | 23972531 |
| Marker50484 | LG4 | 23977500 | 23977450 | 23977550 |
| Marker50498 | LG4 | 23982621 | 23982571 | 23982671 |
| Marker50571 | LG4 | 24096118 | 24096068 | 24096168 |
| Marker50586 | LG4 | 24107229 | 24107179 | 24107279 |
| Marker50594 | LG4 | 24116518 | 24116468 | 24116568 |
| Marker50603 | LG4 | 24128392 | 24128342 | 24128442 |
| Marker50684 | LG4 | 24204608 | 24204558 | 24204658 |
| Marker50702 | LG4 | 24330528 | 24330478 | 24330578 |
| Marker50706 | LG4 | 24345933 | 24345883 | 24345983 |
| Marker50795 | LG4 | 24449502 | 24449452 | 24449552 |
| Marker50812 | LG4 | 24488418 | 24488368 | 24488468 |
| Marker116930 | LG4 | 24569920 | 24569870 | 24569970 |
| Marker116909 | LG4 | 24588520 | 24588470 | 24588570 |
| Marker116902 | LG4 | 24597523 | 24597473 | 24597573 |
| Marker116900 | LG4 | 24606549 | 24606499 | 24606599 |
| Marker50252 | LG4 | 24736250 | 24736200 | 24736300 |
| Marker50249 | LG4 | 24736437 | 24736387 | 24736487 |
| Marker50232 | LG4 | 24763310 | 24763260 | 24763360 |
| Marker50229 | LG4 | 24763586 | 24763536 | 24763636 |
| Marker50216 | LG4 | 24809141 | 24809091 | 24809191 |
| Marker50208 | LG4 | 24815034 | 24814984 | 24815084 |
| Marker50203 | LG4 | 24819245 | 24819195 | 24819295 |
| Marker50202 | LG4 | 24824447 | 24824397 | 24824497 |
| Marker50196 | LG4 | 24839556 | 24839506 | 24839606 |
| Marker50176 | LG4 | 24865105 | 24865055 | 24865155 |
| Marker50154 | LG4 | 24906188 | 24906138 | 24906238 |
| Marker50152 | LG4 | 24906487 | 24906437 | 24906537 |
| Marker50147 | LG4 | 24909457 | 24909407 | 24909507 |
| Marker50125 | LG4 | 24941546 | 24941496 | 24941596 |
| Marker50122 | LG4 | 24942867 | 24942817 | 24942917 |
| Marker50111 | LG4 | 24948247 | 24948197 | 24948297 |
| Marker50106 | LG4 | 24957947 | 24957897 | 24957997 |
| Marker50056 | LG4 | 25037868 | 25037818 | 25037918 |
| Marker50039 | LG4 | 25091594 | 25091544 | 25091644 |
| Marker50036 | LG4 | 25097858 | 25097808 | 25097908 |
| Marker50024 | LG4 | 25110197 | 25110147 | 25110247 |
| Marker50021 | LG4 | 25120131 | 25120081 | 25120181 |
| Marker49996 | LG4 | 25143311 | 25143261 | 25143361 |
| Marker49994 | LG4 | 25144004 | 25143954 | 25144054 |
| Marker49993 | LG4 | 25148833 | 25148783 | 25148883 |
| Marker49947 | LG4 | 25207980 | 25207930 | 25208030 |
| Marker49912 | LG4 | 25241485 | 25241435 | 25241535 |
| Marker49904 | LG4 | 25258365 | 25258315 | 25258415 |
| Marker49901 | LG4 | 25274904 | 25274854 | 25274954 |
| Marker49900 | LG4 | 25276222 | 25276172 | 25276272 |
| Marker49899 | LG4 | 25278572 | 25278522 | 25278622 |
| Marker49872 | LG4 | 25294309 | 25294259 | 25294359 |
| Marker49864 | LG4 | 25294824 | 25294774 | 25294874 |
| Marker49851 | LG4 | 25297268 | 25297218 | 25297318 |
| Marker49809 | LG4 | 25346385 | 25346335 | 25346435 |
| Marker49806 | LG4 | 25349776 | 25349726 | 25349826 |
| Marker49788 | LG4 | 25357619 | 25357569 | 25357669 |
| Marker49785 | LG4 | 25365698 | 25365648 | 25365748 |
| Marker49784 | LG4 | 25366858 | 25366808 | 25366908 |
| Marker49778 | LG4 | 25371155 | 25371105 | 25371205 |
| Marker49775 | LG4 | 25371385 | 25371335 | 25371435 |
| Marker49763 | LG4 | 25457326 | 25457276 | 25457376 |
| Marker45541 | LG4 | 25472606 | 25472556 | 25472656 |
| Marker49751 | LG4 | 25474343 | 25474293 | 25474393 |
| Marker49746 | LG4 | 25475035 | 25474985 | 25475085 |
| Marker49726 | LG4 | 25491722 | 25491672 | 25491772 |
| Marker49707 | LG4 | 25521801 | 25521751 | 25521851 |
| Marker49696 | LG4 | 25565300 | 25565250 | 25565350 |
| Marker49691 | LG4 | 25592226 | 25592176 | 25592276 |
| Marker49690 | LG4 | 25598643 | 25598593 | 25598693 |
| Marker49640 | LG4 | 25643579 | 25643529 | 25643629 |
| Marker49633 | LG4 | 25650647 | 25650597 | 25650697 |
| Marker49630 | LG4 | 25652659 | 25652609 | 25652709 |
| Marker49628 | LG4 | 25653310 | 25653260 | 25653360 |
| Marker49621 | LG4 | 25673567 | 25673517 | 25673617 |
| Marker49600 | LG4 | 25705813 | 25705763 | 25705863 |
| Marker49583 | LG4 | 25732894 | 25732844 | 25732944 |
| Marker49574 | LG4 | 25751876 | 25751826 | 25751926 |
| Marker49555 | LG4 | 25775949 | 25775899 | 25775999 |
| Marker49549 | LG4 | 25790596 | 25790546 | 25790646 |
| Marker49542 | LG4 | 25797048 | 25796998 | 25797098 |
| Marker49540 | LG4 | 25797254 | 25797204 | 25797304 |
| Marker63913 | LG5 | 42214 | 42164 | 42264 |
| Marker63782 | LG5 | 272746 | 272696 | 272796 |
| Marker63733 | LG5 | 443679 | 443629 | 443729 |
| Marker63731 | LG5 | 444277 | 444227 | 444327 |
| Marker120505 | LG5 | 467332 | 467282 | 467382 |
| Marker118868 | LG5 | 492566 | 492516 | 492616 |
| Marker118866 | LG5 | 497606 | 497556 | 497656 |
| Marker118849 | LG5 | 527566 | 527516 | 527616 |
| Marker64043 | LG5 | 582095 | 582045 | 582145 |
| Marker64041 | LG5 | 586187 | 586137 | 586237 |
| Marker64016 | LG5 | 614853 | 614803 | 614903 |
| Marker64015 | LG5 | 618609 | 618559 | 618659 |
| Marker64006 | LG5 | 632649 | 632599 | 632699 |
| Marker63997 | LG5 | 641706 | 641656 | 641756 |
| Marker63953 | LG5 | 676992 | 676942 | 677042 |
| Marker8154 | LG5 | 720457 | 720407 | 720507 |
| Marker8128 | LG5 | 751885 | 751835 | 751935 |
| Marker8120 | LG5 | 762215 | 762165 | 762265 |
| Marker8119 | LG5 | 762462 | 762412 | 762512 |
| Marker8106 | LG5 | 785135 | 785085 | 785185 |
| Marker63659 | LG5 | 952549 | 952499 | 952599 |
| Marker63670 | LG5 | 989052 | 989002 | 989102 |
| Marker63679 | LG5 | 1026934 | 1026884 | 1026984 |
| Marker63686 | LG5 | 1030514 | 1030464 | 1030564 |
| Marker63687 | LG5 | 1032681 | 1032631 | 1032731 |
| Marker63694 | LG5 | 1045444 | 1045394 | 1045494 |
| Marker63491 | LG5 | 1160361 | 1160311 | 1160411 |
| Marker63577 | LG5 | 1234140 | 1234090 | 1234190 |
| Marker63588 | LG5 | 1242904 | 1242854 | 1242954 |
| Marker63593 | LG5 | 1248644 | 1248594 | 1248694 |
| Marker63606 | LG5 | 1253513 | 1253463 | 1253563 |
| Marker64393 | LG5 | 1487489 | 1487439 | 1487539 |
| Marker64391 | LG5 | 1487822 | 1487772 | 1487872 |
| Marker64340 | LG5 | 1517227 | 1517177 | 1517277 |
| Marker64310 | LG5 | 1557189 | 1557139 | 1557239 |
| Marker64307 | LG5 | 1570874 | 1570824 | 1570924 |
| Marker64275 | LG5 | 1604214 | 1604164 | 1604264 |
| Marker64254 | LG5 | 1623656 | 1623606 | 1623706 |
| Marker64215 | LG5 | 1673520 | 1673470 | 1673570 |
| Marker64213 | LG5 | 1674069 | 1674019 | 1674119 |
| Marker64200 | LG5 | 1721607 | 1721557 | 1721657 |
| Marker64099 | LG5 | 1783031 | 1782981 | 1783081 |
| Marker64100 | LG5 | 1783314 | 1783264 | 1783364 |
| Marker64114 | LG5 | 1793708 | 1793658 | 1793758 |
| Marker64634 | LG5 | 2195064 | 2195014 | 2195114 |
| Marker64622 | LG5 | 2231601 | 2231551 | 2231651 |
| Marker64601 | LG5 | 2270302 | 2270252 | 2270352 |
| Marker64594 | LG5 | 2274437 | 2274387 | 2274487 |
| Marker64492 | LG5 | 3420422 | 3420372 | 3420472 |
| Marker121814 | LG5 | 3426788 | 3426738 | 3426838 |
| Marker121978 | LG5 | 3530459 | 3530409 | 3530509 |
| Marker65190 | LG5 | 3601459 | 3601409 | 3601509 |
| Marker65219 | LG5 | 3629783 | 3629733 | 3629833 |
| Marker65222 | LG5 | 3636803 | 3636753 | 3636853 |
| Marker65356 | LG5 | 3915891 | 3915841 | 3915941 |
| Marker65358 | LG5 | 3917602 | 3917552 | 3917652 |
| Marker65359 | LG5 | 3928252 | 3928202 | 3928302 |
| Marker65427 | LG5 | 3996321 | 3996271 | 3996371 |
| Marker65459 | LG5 | 4026080 | 4026030 | 4026130 |
| Marker114376 | LG5 | 4069606 | 4069556 | 4069656 |
| Marker64985 | LG5 | 4280503 | 4280453 | 4280553 |
| Marker64986 | LG5 | 4280725 | 4280675 | 4280775 |
| Marker65153 | LG5 | 4689685 | 4689635 | 4689735 |
| Marker65179 | LG5 | 4731307 | 4731257 | 4731357 |
| Marker121486 | LG5 | 4793595 | 4793545 | 4793645 |
| Marker121490 | LG5 | 4795455 | 4795405 | 4795505 |
| Marker121492 | LG5 | 4796403 | 4796353 | 4796453 |
| Marker121293 | LG5 | 4846459 | 4846409 | 4846509 |
| Marker119396 | LG5 | 4848789 | 4848739 | 4848839 |
| Marker118297 | LG5 | 5127454 | 5127404 | 5127504 |
| Marker30521 | LG5 | 5284505 | 5284455 | 5284555 |
| Marker119529 | LG5 | 5358010 | 5357960 | 5358060 |
| Marker121215 | LG5 | 5378671 | 5378621 | 5378721 |
| Marker119528 | LG5 | 5379135 | 5379085 | 5379185 |
| Marker65743 | LG5 | 5472795 | 5472745 | 5472845 |
| Marker65724 | LG5 | 5515135 | 5515085 | 5515185 |
| Marker65696 | LG5 | 5538818 | 5538768 | 5538868 |
| Marker65691 | LG5 | 5557438 | 5557388 | 5557488 |
| Marker65684 | LG5 | 5560816 | 5560766 | 5560866 |
| Marker65646 | LG5 | 5611205 | 5611155 | 5611255 |
| Marker65612 | LG5 | 5627951 | 5627901 | 5628001 |
| Marker65605 | LG5 | 5632434 | 5632384 | 5632484 |
| Marker49369 | LG5 | 5701652 | 5701602 | 5701702 |
| Marker49383 | LG5 | 5721577 | 5721527 | 5721627 |
| Marker49390 | LG5 | 5725071 | 5725021 | 5725121 |
| Marker29086 | LG5 | 5763777 | 5763727 | 5763827 |
| Marker49419 | LG5 | 5763929 | 5763879 | 5763979 |
| Marker49440 | LG5 | 5778308 | 5778258 | 5778358 |
| Marker48911 | LG5 | 5835628 | 5835578 | 5835678 |
| Marker48905 | LG5 | 5836163 | 5836113 | 5836213 |
| Marker48875 | LG5 | 5926239 | 5926189 | 5926289 |
| Marker113263 | LG5 | 5966421 | 5966371 | 5966471 |
| Marker113266 | LG5 | 5966678 | 5966628 | 5966728 |
| Marker65775 | LG5 | 6137914 | 6137864 | 6137964 |
| Marker65793 | LG5 | 6147545 | 6147495 | 6147595 |
| Marker118460 | LG5 | 6154723 | 6154673 | 6154773 |
| Marker118477 | LG5 | 6161193 | 6161143 | 6161243 |
| Marker119129 | LG5 | 6189566 | 6189516 | 6189616 |
| Marker65953 | LG5 | 6386972 | 6386922 | 6387022 |
| Marker65938 | LG5 | 6395622 | 6395572 | 6395672 |
| Marker65923 | LG5 | 6409298 | 6409248 | 6409348 |
| Marker65917 | LG5 | 6421920 | 6421870 | 6421970 |
| Marker65914 | LG5 | 6426596 | 6426546 | 6426646 |
| Marker66701 | LG5 | 6655727 | 6655677 | 6655777 |
| Marker66709 | LG5 | 6669546 | 6669496 | 6669596 |
| Marker66720 | LG5 | 6680760 | 6680710 | 6680810 |
| Marker66763 | LG5 | 6741154 | 6741104 | 6741204 |
| Marker66765 | LG5 | 6742389 | 6742339 | 6742439 |
| Marker66797 | LG5 | 6778549 | 6778499 | 6778599 |
| Marker66801 | LG5 | 6780050 | 6780000 | 6780100 |
| Marker66828 | LG5 | 6807871 | 6807821 | 6807921 |
| Marker119219 | LG5 | 6971751 | 6971701 | 6971801 |
| Marker118750 | LG5 | 7189020 | 7188970 | 7189070 |
| Marker115470 | LG5 | 7298161 | 7298111 | 7298211 |
| Marker115419 | LG5 | 7369420 | 7369370 | 7369470 |
| Marker66588 | LG5 | 7440392 | 7440342 | 7440442 |
| Marker66607 | LG5 | 7449100 | 7449050 | 7449150 |
| Marker67472 | LG5 | 7616892 | 7616842 | 7616942 |
| Marker66469 | LG5 | 7726862 | 7726812 | 7726912 |
| Marker66436 | LG5 | 7778345 | 7778295 | 7778395 |
| Marker21877 | LG5 | 7806778 | 7806728 | 7806828 |
| Marker66399 | LG5 | 7843192 | 7843142 | 7843242 |
| Marker66367 | LG5 | 7881478 | 7881428 | 7881528 |
| Marker66361 | LG5 | 7919636 | 7919586 | 7919686 |
| Marker66345 | LG5 | 7936479 | 7936429 | 7936529 |
| Marker67526 | LG5 | 8011427 | 8011377 | 8011477 |
| Marker67640 | LG5 | 8109372 | 8109322 | 8109422 |
| Marker67662 | LG5 | 8154785 | 8154735 | 8154835 |
| Marker67691 | LG5 | 8181249 | 8181199 | 8181299 |
| Marker67697 | LG5 | 8187672 | 8187622 | 8187722 |
| Marker67755 | LG5 | 8240089 | 8240039 | 8240139 |
| Marker67759 | LG5 | 8247904 | 8247854 | 8247954 |
| Marker66921 | LG5 | 8483690 | 8483640 | 8483740 |
| Marker67114 | LG5 | 8742030 | 8741980 | 8742080 |
| Marker67152 | LG5 | 8770284 | 8770234 | 8770334 |
| Marker67155 | LG5 | 8773895 | 8773845 | 8773945 |
| Marker67193 | LG5 | 8810601 | 8810551 | 8810651 |
| Marker67198 | LG5 | 8820716 | 8820666 | 8820766 |
| Marker67963 | LG5 | 9165949 | 9165899 | 9165999 |
| Marker67950 | LG5 | 9189668 | 9189618 | 9189718 |
| Marker67941 | LG5 | 9198210 | 9198160 | 9198260 |
| Marker67939 | LG5 | 9204815 | 9204765 | 9204865 |
| Marker67936 | LG5 | 9210369 | 9210319 | 9210419 |
| Marker67913 | LG5 | 9231510 | 9231460 | 9231560 |
| Marker67904 | LG5 | 9237641 | 9237591 | 9237691 |
| Marker67515 | LG5 | 9303844 | 9303794 | 9303894 |
| Marker7398 | LG5 | 9529527 | 9529477 | 9529577 |
| Marker68217 | LG5 | 9896608 | 9896558 | 9896658 |
| Marker68245 | LG5 | 9916252 | 9916202 | 9916302 |
| Marker66034 | LG5 | 10007237 | 10007187 | 10007287 |
| Marker66029 | LG5 | 10010161 | 10010111 | 10010211 |
| Marker67235 | LG5 | 10131119 | 10131069 | 10131169 |
| Marker67230 | LG5 | 10140082 | 10140032 | 10140132 |
| Marker66142 | LG5 | 10518688 | 10518638 | 10518738 |
| Marker89057 | LG5 | 10533029 | 10532979 | 10533079 |
| Marker66194 | LG5 | 10545180 | 10545130 | 10545230 |
| Marker3211 | LG5 | 10571637 | 10571587 | 10571687 |
| Marker68014 | LG5 | 10655860 | 10655810 | 10655910 |
| Marker68036 | LG5 | 10682348 | 10682298 | 10682398 |
| Marker68051 | LG5 | 10689980 | 10689930 | 10690030 |
| Marker68052 | LG5 | 10690195 | 10690145 | 10690245 |
| Marker68128 | LG5 | 10764836 | 10764786 | 10764886 |
| Marker119133 | LG5 | 10948153 | 10948103 | 10948203 |
| Marker68600 | LG5 | 11021324 | 11021274 | 11021374 |
| Marker68617 | LG5 | 11188594 | 11188544 | 11188644 |
| Marker68673 | LG5 | 11242216 | 11242166 | 11242266 |
| Marker68686 | LG5 | 11253995 | 11253945 | 11254045 |
| Marker68690 | LG5 | 11284637 | 11284587 | 11284687 |
| Marker68371 | LG5 | 11357302 | 11357252 | 11357352 |
| Marker68381 | LG5 | 11373220 | 11373170 | 11373270 |
| Marker68385 | LG5 | 11374176 | 11374126 | 11374226 |
| Marker69780 | LG5 | 11443696 | 11443646 | 11443746 |
| Marker69783 | LG5 | 11446054 | 11446004 | 11446104 |
| Marker69820 | LG5 | 11473852 | 11473802 | 11473902 |
| Marker69825 | LG5 | 11474149 | 11474099 | 11474199 |
| Marker68432 | LG5 | 11657212 | 11657162 | 11657262 |
| Marker68435 | LG5 | 11665040 | 11664990 | 11665090 |
| Marker68495 | LG5 | 11776751 | 11776701 | 11776801 |
| Marker68498 | LG5 | 11791063 | 11791013 | 11791113 |
| Marker16327 | LG5 | 11853350 | 11853300 | 11853400 |
| Marker16360 | LG5 | 11917457 | 11917407 | 11917507 |
| Marker68869 | LG5 | 11977140 | 11977090 | 11977190 |
| Marker16405 | LG5 | 12104965 | 12104915 | 12105015 |
| Marker68841 | LG5 | 12113983 | 12113933 | 12114033 |
| Marker68821 | LG5 | 12124414 | 12124364 | 12124464 |
| Marker68807 | LG5 | 12135621 | 12135571 | 12135671 |
| Marker68768 | LG5 | 12173876 | 12173826 | 12173926 |
| Marker68766 | LG5 | 12183497 | 12183447 | 12183547 |
| Marker68747 | LG5 | 12213743 | 12213693 | 12213793 |
| Marker68724 | LG5 | 12222528 | 12222478 | 12222578 |
| Marker68722 | LG5 | 12226614 | 12226564 | 12226664 |
| Marker68721 | LG5 | 12226861 | 12226811 | 12226911 |
| Marker68883 | LG5 | 12462075 | 12462025 | 12462125 |
| Marker68938 | LG5 | 12483459 | 12483409 | 12483509 |
| Marker68945 | LG5 | 12521132 | 12521082 | 12521182 |
| Marker68952 | LG5 | 12544876 | 12544826 | 12544926 |
| Marker69000 | LG5 | 12677763 | 12677713 | 12677813 |
| Marker69016 | LG5 | 12694220 | 12694170 | 12694270 |
| Marker69023 | LG5 | 12713901 | 12713851 | 12713951 |
| Marker68695 | LG5 | 12736202 | 12736152 | 12736252 |
| Marker69069 | LG5 | 12892096 | 12892046 | 12892146 |
| Marker69074 | LG5 | 12892377 | 12892327 | 12892427 |
| Marker69079 | LG5 | 12918770 | 12918720 | 12918820 |
| Marker69088 | LG5 | 12922012 | 12921962 | 12922062 |
| Marker69097 | LG5 | 12940402 | 12940352 | 12940452 |
| Marker69214 | LG5 | 13014226 | 13014176 | 13014276 |
| Marker69349 | LG5 | 13106934 | 13106884 | 13106984 |
| Marker69380 | LG5 | 13152029 | 13151979 | 13152079 |
| Marker69448 | LG5 | 13215425 | 13215375 | 13215475 |
| Marker69465 | LG5 | 13259674 | 13259624 | 13259724 |
| Marker69471 | LG5 | 13262427 | 13262377 | 13262477 |
| Marker69509 | LG5 | 13314669 | 13314619 | 13314719 |
| Marker69568 | LG5 | 13344205 | 13344155 | 13344255 |
| Marker120891 | LG5 | 13352549 | 13352499 | 13352599 |
| Marker69613 | LG5 | 13415011 | 13414961 | 13415061 |
| Marker69665 | LG5 | 13466883 | 13466833 | 13466933 |
| Marker69677 | LG5 | 13483926 | 13483876 | 13483976 |
| Marker69899 | LG5 | 13646423 | 13646373 | 13646473 |
| Marker69898 | LG5 | 13648520 | 13648470 | 13648570 |
| Marker69891 | LG5 | 13656174 | 13656124 | 13656224 |
| Marker69848 | LG5 | 13701160 | 13701110 | 13701210 |
| Marker69827 | LG5 | 13766884 | 13766834 | 13766934 |
| Marker70164 | LG5 | 13791229 | 13791179 | 13791279 |
| Marker70182 | LG5 | 13872710 | 13872660 | 13872760 |
| Marker70040 | LG5 | 14019540 | 14019490 | 14019590 |
| Marker70039 | LG5 | 14026959 | 14026909 | 14027009 |
| Marker70025 | LG5 | 14068883 | 14068833 | 14068933 |
| Marker69985 | LG5 | 14105143 | 14105093 | 14105193 |
| Marker69981 | LG5 | 14116510 | 14116460 | 14116560 |
| Marker66561 | LG5 | 14124406 | 14124356 | 14124456 |
| Marker70227 | LG5 | 14260523 | 14260473 | 14260573 |
| Marker70252 | LG5 | 14463273 | 14463223 | 14463323 |
| Marker70248 | LG5 | 14465312 | 14465262 | 14465362 |
| Marker70299 | LG5 | 14469432 | 14469382 | 14469482 |
| Marker70318 | LG5 | 14484352 | 14484302 | 14484402 |
| Marker70324 | LG5 | 14493214 | 14493164 | 14493264 |
| Marker70370 | LG5 | 14527840 | 14527790 | 14527890 |
| Marker70392 | LG5 | 14540369 | 14540319 | 14540419 |
| Marker70395 | LG5 | 14541643 | 14541593 | 14541693 |
| Marker70461 | LG5 | 14645103 | 14645053 | 14645153 |
| Marker70499 | LG5 | 14706046 | 14705996 | 14706096 |
| Marker70557 | LG5 | 14728818 | 14728768 | 14728868 |
| Marker70559 | LG5 | 14732332 | 14732282 | 14732382 |
| Marker70573 | LG5 | 14775157 | 14775107 | 14775207 |
| Marker70587 | LG5 | 14795999 | 14795949 | 14796049 |
| Marker70589 | LG5 | 14796270 | 14796220 | 14796320 |
| Marker70592 | LG5 | 14799052 | 14799002 | 14799102 |
| Marker70598 | LG5 | 14810721 | 14810671 | 14810771 |
| Marker70602 | LG5 | 14817382 | 14817332 | 14817432 |
| Marker121705 | LG5 | 14882394 | 14882344 | 14882444 |
| Marker70635 | LG5 | 14900192 | 14900142 | 14900242 |
| Marker70656 | LG5 | 14926284 | 14926234 | 14926334 |
| Marker70730 | LG5 | 14971575 | 14971525 | 14971625 |
| Marker70751 | LG5 | 14992413 | 14992363 | 14992463 |
| Marker70767 | LG5 | 15005135 | 15005085 | 15005185 |
| Marker70777 | LG5 | 15008209 | 15008159 | 15008259 |
| Marker70778 | LG5 | 15011493 | 15011443 | 15011543 |
| Marker70789 | LG5 | 15019032 | 15018982 | 15019082 |
| Marker70807 | LG5 | 15031933 | 15031883 | 15031983 |
| Marker70809 | LG5 | 15032111 | 15032061 | 15032161 |
| Marker70810 | LG5 | 15036339 | 15036289 | 15036389 |
| Marker70821 | LG5 | 15045824 | 15045774 | 15045874 |
| Marker70860 | LG5 | 15071721 | 15071671 | 15071771 |
| Marker70866 | LG5 | 15077476 | 15077426 | 15077526 |
| Marker70914 | LG5 | 15135923 | 15135873 | 15135973 |
| Marker70924 | LG5 | 15148194 | 15148144 | 15148244 |
| Marker70940 | LG5 | 15156488 | 15156438 | 15156538 |
| Marker70944 | LG5 | 15165789 | 15165739 | 15165839 |
| Marker70945 | LG5 | 15165989 | 15165939 | 15166039 |
| Marker70963 | LG5 | 15212600 | 15212550 | 15212650 |
| Marker71020 | LG5 | 15404394 | 15404344 | 15404444 |
| Marker71054 | LG5 | 15537245 | 15537195 | 15537295 |
| Marker71076 | LG5 | 15542742 | 15542692 | 15542792 |
| Marker71084 | LG5 | 15549329 | 15549279 | 15549379 |
| Marker71106 | LG5 | 15572949 | 15572899 | 15572999 |
| Marker71107 | LG5 | 15574707 | 15574657 | 15574757 |
| Marker71093 | LG5 | 15586261 | 15586211 | 15586311 |
| Marker71110 | LG5 | 15599556 | 15599506 | 15599606 |
| Marker71113 | LG5 | 15599977 | 15599927 | 15600027 |
| Marker71125 | LG5 | 15611068 | 15611018 | 15611118 |
| Marker71127 | LG5 | 15621645 | 15621595 | 15621695 |
| Marker71130 | LG5 | 15636320 | 15636270 | 15636370 |
| Marker71139 | LG5 | 15656674 | 15656624 | 15656724 |
| Marker71140 | LG5 | 15656876 | 15656826 | 15656926 |
| Marker71255 | LG5 | 15939675 | 15939625 | 15939725 |
| Marker71259 | LG5 | 15945845 | 15945795 | 15945895 |
| Marker71320 | LG5 | 16043052 | 16043002 | 16043102 |
| Marker71325 | LG5 | 16044908 | 16044858 | 16044958 |
| Marker71328 | LG5 | 16059133 | 16059083 | 16059183 |
| Marker71367 | LG5 | 16102835 | 16102785 | 16102885 |
| Marker71369 | LG5 | 16103071 | 16103021 | 16103121 |
| Marker71376 | LG5 | 16115829 | 16115779 | 16115879 |
| Marker71395 | LG5 | 16145779 | 16145729 | 16145829 |
| Marker71429 | LG5 | 16212859 | 16212809 | 16212909 |
| Marker71907 | LG5 | 16309855 | 16309805 | 16309905 |
| Marker71929 | LG5 | 16349435 | 16349385 | 16349485 |
| Marker71932 | LG5 | 16356836 | 16356786 | 16356886 |
| Marker71933 | LG5 | 16359475 | 16359425 | 16359525 |
| Marker71937 | LG5 | 16368059 | 16368009 | 16368109 |
| Marker71938 | LG5 | 16371690 | 16371640 | 16371740 |
| Marker71945 | LG5 | 16372825 | 16372775 | 16372875 |
| Marker71946 | LG5 | 16377300 | 16377250 | 16377350 |
| Marker71948 | LG5 | 16377505 | 16377455 | 16377555 |
| Marker71951 | LG5 | 16378984 | 16378934 | 16379034 |
| Marker71955 | LG5 | 16380744 | 16380694 | 16380794 |
| Marker71957 | LG5 | 16381012 | 16380962 | 16381062 |
| Marker71965 | LG5 | 16398253 | 16398203 | 16398303 |
| Marker71991 | LG5 | 16455051 | 16455001 | 16455101 |
| Marker72007 | LG5 | 16482000 | 16481950 | 16482050 |
| Marker72026 | LG5 | 16497416 | 16497366 | 16497466 |
| Marker72028 | LG5 | 16497663 | 16497613 | 16497713 |
| Marker72029 | LG5 | 16499318 | 16499268 | 16499368 |
| Marker72039 | LG5 | 16510153 | 16510103 | 16510203 |
| Marker72116 | LG5 | 16563422 | 16563372 | 16563472 |
| Marker71544 | LG5 | 16750206 | 16750156 | 16750256 |
| Marker71562 | LG5 | 16772304 | 16772254 | 16772354 |
| Marker71565 | LG5 | 16776211 | 16776161 | 16776261 |
| Marker71568 | LG5 | 16778106 | 16778056 | 16778156 |
| Marker71569 | LG5 | 16779878 | 16779828 | 16779928 |
| Marker71586 | LG5 | 16796673 | 16796623 | 16796723 |
| Marker71606 | LG5 | 16822160 | 16822110 | 16822210 |
| Marker71622 | LG5 | 16875851 | 16875801 | 16875901 |
| Marker71676 | LG5 | 16895930 | 16895880 | 16895980 |
| Marker71742 | LG5 | 16963096 | 16963046 | 16963146 |
| Marker71749 | LG5 | 16970246 | 16970196 | 16970296 |
| Marker71765 | LG5 | 16990204 | 16990154 | 16990254 |
| Marker71768 | LG5 | 16993186 | 16993136 | 16993236 |
| Marker71791 | LG5 | 17034187 | 17034137 | 17034237 |
| Marker71808 | LG5 | 17043652 | 17043602 | 17043702 |
| Marker71815 | LG5 | 17046712 | 17046662 | 17046762 |
| Marker71817 | LG5 | 17049943 | 17049893 | 17049993 |
| Marker71828 | LG5 | 17060055 | 17060005 | 17060105 |
| Marker71835 | LG5 | 17063454 | 17063404 | 17063504 |
| Marker71844 | LG5 | 17070647 | 17070597 | 17070697 |
| Marker71864 | LG5 | 17113997 | 17113947 | 17114047 |
| Marker115743 | LG5 | 17123511 | 17123461 | 17123561 |
| Marker71872 | LG5 | 17133594 | 17133544 | 17133644 |
| Marker115717 | LG5 | 17206704 | 17206654 | 17206754 |
| Marker115700 | LG5 | 17247978 | 17247928 | 17248028 |
| Marker72316 | LG5 | 17389135 | 17389085 | 17389185 |
| Marker72312 | LG5 | 17397276 | 17397226 | 17397326 |
| Marker72180 | LG5 | 17450505 | 17450455 | 17450555 |
| Marker72174 | LG5 | 17465089 | 17465039 | 17465139 |
| Marker72162 | LG5 | 17472795 | 17472745 | 17472845 |
| Marker114271 | LG5 | 17524535 | 17524485 | 17524585 |
| Marker114288 | LG5 | 17634544 | 17634494 | 17634594 |
| Marker114337 | LG5 | 17685478 | 17685428 | 17685528 |
| Marker114349 | LG5 | 17707635 | 17707585 | 17707685 |
| Marker72647 | LG5 | 17733794 | 17733744 | 17733844 |
| Marker72644 | LG5 | 17755513 | 17755463 | 17755563 |
| Marker72635 | LG5 | 17778870 | 17778820 | 17778920 |
| Marker72634 | LG5 | 17781684 | 17781634 | 17781734 |
| Marker72629 | LG5 | 17785241 | 17785191 | 17785291 |
| Marker72556 | LG5 | 17868553 | 17868503 | 17868603 |
| Marker72506 | LG5 | 17912649 | 17912599 | 17912699 |
| Marker72499 | LG5 | 17918954 | 17918904 | 17919004 |
| Marker72497 | LG5 | 17921328 | 17921278 | 17921378 |
| Marker72494 | LG5 | 17928626 | 17928576 | 17928676 |
| Marker72466 | LG5 | 17985156 | 17985106 | 17985206 |
| Marker72452 | LG5 | 18005640 | 18005590 | 18005690 |
| Marker72448 | LG5 | 18022572 | 18022522 | 18022622 |
| Marker72435 | LG5 | 18031635 | 18031585 | 18031685 |
| Marker72426 | LG5 | 18046192 | 18046142 | 18046242 |
| Marker72406 | LG5 | 18071604 | 18071554 | 18071654 |
| Marker72398 | LG5 | 18086119 | 18086069 | 18086169 |
| Marker72379 | LG5 | 18135537 | 18135487 | 18135587 |
| Marker72377 | LG5 | 18138076 | 18138026 | 18138126 |
| Marker72370 | LG5 | 18148675 | 18148625 | 18148725 |
| Marker72355 | LG5 | 18148952 | 18148902 | 18149002 |
| Marker72335 | LG5 | 18220652 | 18220602 | 18220702 |
| Marker73173 | LG5 | 18314183 | 18314133 | 18314233 |
| Marker73170 | LG5 | 18318564 | 18318514 | 18318614 |
| Marker73167 | LG5 | 18325328 | 18325278 | 18325378 |
| Marker73159 | LG5 | 18332318 | 18332268 | 18332368 |
| Marker73149 | LG5 | 18346118 | 18346068 | 18346168 |
| Marker73130 | LG5 | 18377295 | 18377245 | 18377345 |
| Marker73128 | LG5 | 18377741 | 18377691 | 18377791 |
| Marker73124 | LG5 | 18385712 | 18385662 | 18385762 |
| Marker73117 | LG5 | 18387815 | 18387765 | 18387865 |
| Marker73107 | LG5 | 18403675 | 18403625 | 18403725 |
| Marker73098 | LG5 | 18416549 | 18416499 | 18416599 |
| Marker121382 | LG5 | 18462167 | 18462117 | 18462217 |
| Marker73063 | LG5 | 18533100 | 18533050 | 18533150 |
| Marker73046 | LG5 | 18568152 | 18568102 | 18568202 |
| Marker73045 | LG5 | 18569396 | 18569346 | 18569446 |
| Marker73039 | LG5 | 18599753 | 18599703 | 18599803 |
| Marker73036 | LG5 | 18636494 | 18636444 | 18636544 |
| Marker73023 | LG5 | 18662326 | 18662276 | 18662376 |
| Marker73016 | LG5 | 18668052 | 18668002 | 18668102 |
| Marker73008 | LG5 | 18676375 | 18676325 | 18676425 |
| Marker72980 | LG5 | 18749011 | 18748961 | 18749061 |
| Marker72973 | LG5 | 18766994 | 18766944 | 18767044 |
| Marker72971 | LG5 | 18774227 | 18774177 | 18774277 |
| Marker72970 | LG5 | 18779277 | 18779227 | 18779327 |
| Marker72964 | LG5 | 18786006 | 18785956 | 18786056 |
| Marker72950 | LG5 | 18807265 | 18807215 | 18807315 |
| Marker72949 | LG5 | 18818471 | 18818421 | 18818521 |
| Marker72948 | LG5 | 18818714 | 18818664 | 18818764 |
| Marker72926 | LG5 | 18852370 | 18852320 | 18852420 |
| Marker72847 | LG5 | 18965953 | 18965903 | 18966003 |
| Marker72835 | LG5 | 18988690 | 18988640 | 18988740 |
| Marker72830 | LG5 | 18988950 | 18988900 | 18989000 |
| Marker72829 | LG5 | 18994123 | 18994073 | 18994173 |
| Marker72825 | LG5 | 19050182 | 19050132 | 19050232 |
| Marker72806 | LG5 | 19108064 | 19108014 | 19108114 |
| Marker72794 | LG5 | 19141724 | 19141674 | 19141774 |
| Marker72790 | LG5 | 19154824 | 19154774 | 19154874 |
| Marker72769 | LG5 | 19182279 | 19182229 | 19182329 |
| Marker72758 | LG5 | 19202728 | 19202678 | 19202778 |
| Marker72753 | LG5 | 19212052 | 19212002 | 19212102 |
| Marker72747 | LG5 | 19213938 | 19213888 | 19213988 |
| Marker72745 | LG5 | 19214236 | 19214186 | 19214286 |
| Marker72739 | LG5 | 19236571 | 19236521 | 19236621 |
| Marker72717 | LG5 | 19260453 | 19260403 | 19260503 |
| Marker72677 | LG5 | 19318558 | 19318508 | 19318608 |
| Marker72676 | LG5 | 19326303 | 19326253 | 19326353 |
| Marker111841 | LG5 | 19418365 | 19418315 | 19418415 |
| Marker111840 | LG5 | 19418425 | 19418375 | 19418475 |
| Marker111825 | LG5 | 19459148 | 19459098 | 19459198 |
| Marker111813 | LG5 | 19471101 | 19471051 | 19471151 |
| Marker73311 | LG5 | 19585091 | 19585041 | 19585141 |
| Marker73307 | LG5 | 19593218 | 19593168 | 19593268 |
| Marker73285 | LG5 | 19647621 | 19647571 | 19647671 |
| Marker73280 | LG5 | 19649755 | 19649705 | 19649805 |
| Marker73270 | LG5 | 19663937 | 19663887 | 19663987 |
| Marker73266 | LG5 | 19668109 | 19668059 | 19668159 |
| Marker73265 | LG5 | 19669616 | 19669566 | 19669666 |
| Marker73264 | LG5 | 19670475 | 19670425 | 19670525 |
| Marker73254 | LG5 | 19695074 | 19695024 | 19695124 |
| Marker73236 | LG5 | 19740844 | 19740794 | 19740894 |
| Marker73228 | LG5 | 19757011 | 19756961 | 19757061 |
| Marker73201 | LG5 | 19810017 | 19809967 | 19810067 |
| Marker73198 | LG5 | 19827254 | 19827204 | 19827304 |
| Marker73197 | LG5 | 19827311 | 19827261 | 19827361 |
| Marker73188 | LG5 | 19836555 | 19836505 | 19836605 |
| Marker111750 | LG5 | 19894203 | 19894153 | 19894253 |
| Marker111751 | LG5 | 19895664 | 19895614 | 19895714 |
| Marker73336 | LG5 | 20175185 | 20175135 | 20175235 |
| Marker73356 | LG5 | 20187575 | 20187525 | 20187625 |
| Marker10327 | LG5 | 20190917 | 20190867 | 20190967 |
| Marker10331 | LG5 | 20191758 | 20191708 | 20191808 |
| Marker73387 | LG5 | 20235190 | 20235140 | 20235240 |
| Marker73401 | LG5 | 20264627 | 20264577 | 20264677 |
| Marker73468 | LG5 | 20405080 | 20405030 | 20405130 |
| Marker73475 | LG5 | 20416792 | 20416742 | 20416842 |
| Marker73476 | LG5 | 20418978 | 20418928 | 20419028 |
| Marker73481 | LG5 | 20420762 | 20420712 | 20420812 |
| Marker73483 | LG5 | 20431866 | 20431816 | 20431916 |
| Marker73486 | LG5 | 20435749 | 20435699 | 20435799 |
| Marker73495 | LG5 | 20449449 | 20449399 | 20449499 |
| Marker73497 | LG5 | 20458872 | 20458822 | 20458922 |
| Marker73504 | LG5 | 20470325 | 20470275 | 20470375 |
| Marker73508 | LG5 | 20472780 | 20472730 | 20472830 |
| Marker73509 | LG5 | 20473095 | 20473045 | 20473145 |
| Marker73510 | LG5 | 20473850 | 20473800 | 20473900 |
| Marker73511 | LG5 | 20474024 | 20473974 | 20474074 |
| Marker73512 | LG5 | 20477936 | 20477886 | 20477986 |
| Marker73539 | LG5 | 20565526 | 20565476 | 20565576 |
| Marker73551 | LG5 | 20615368 | 20615318 | 20615418 |
| Marker73552 | LG5 | 20621398 | 20621348 | 20621448 |
| Marker73555 | LG5 | 20622788 | 20622738 | 20622838 |
| Marker73562 | LG5 | 20628454 | 20628404 | 20628504 |
| Marker73599 | LG5 | 20721546 | 20721496 | 20721596 |
| Marker73631 | LG5 | 20830179 | 20830129 | 20830229 |
| Marker73660 | LG5 | 20925120 | 20925070 | 20925170 |
| Marker73702 | LG5 | 20966841 | 20966791 | 20966891 |
| Marker73720 | LG5 | 21017929 | 21017879 | 21017979 |
| Marker73734 | LG5 | 21071639 | 21071589 | 21071689 |
| Marker74098 | LG5 | 21542472 | 21542422 | 21542522 |
| Marker74105 | LG5 | 21568685 | 21568635 | 21568735 |
| Marker74132 | LG5 | 21603936 | 21603886 | 21603986 |
| Marker74145 | LG5 | 21628331 | 21628281 | 21628381 |
| Marker74151 | LG5 | 21635515 | 21635465 | 21635565 |
| Marker74170 | LG5 | 21668214 | 21668164 | 21668264 |
| Marker74171 | LG5 | 21674926 | 21674876 | 21674976 |
| Marker74173 | LG5 | 21677907 | 21677857 | 21677957 |
| Marker74189 | LG5 | 21697100 | 21697050 | 21697150 |
| Marker74215 | LG5 | 21732512 | 21732462 | 21732562 |
| Marker74220 | LG5 | 21754854 | 21754804 | 21754904 |
| Marker74247 | LG5 | 21767872 | 21767822 | 21767922 |
| Marker74270 | LG5 | 21790679 | 21790629 | 21790729 |
| Marker74292 | LG5 | 21869294 | 21869244 | 21869344 |
| Marker74293 | LG5 | 21870092 | 21870042 | 21870142 |
| Marker74301 | LG5 | 21883185 | 21883135 | 21883235 |
| Marker74341 | LG5 | 21948120 | 21948070 | 21948170 |
| Marker74354 | LG5 | 21974659 | 21974609 | 21974709 |
| Marker74370 | LG5 | 22008971 | 22008921 | 22009021 |
| Marker74376 | LG5 | 22035715 | 22035665 | 22035765 |
| Marker74380 | LG5 | 22043835 | 22043785 | 22043885 |
| Marker74397 | LG5 | 22076082 | 22076032 | 22076132 |
| Marker74407 | LG5 | 22084613 | 22084563 | 22084663 |
| Marker74408 | LG5 | 22088561 | 22088511 | 22088611 |
| Marker74425 | LG5 | 22114628 | 22114578 | 22114678 |
| Marker74437 | LG5 | 22132823 | 22132773 | 22132873 |
| Marker73853 | LG5 | 22156246 | 22156196 | 22156296 |
| Marker73861 | LG5 | 22187186 | 22187136 | 22187236 |
| Marker73866 | LG5 | 22194217 | 22194167 | 22194267 |
| Marker73867 | LG5 | 22194411 | 22194361 | 22194461 |
| Marker73879 | LG5 | 22226184 | 22226134 | 22226234 |
| Marker73882 | LG5 | 22229179 | 22229129 | 22229229 |
| Marker73885 | LG5 | 22231130 | 22231080 | 22231180 |
| Marker73887 | LG5 | 22234971 | 22234921 | 22235021 |
| Marker74450 | LG5 | 22322307 | 22322257 | 22322357 |
| Marker74469 | LG5 | 22356854 | 22356804 | 22356904 |
| Marker74473 | LG5 | 22367927 | 22367877 | 22367977 |
| Marker74477 | LG5 | 22382526 | 22382476 | 22382576 |
| Marker74479 | LG5 | 22382757 | 22382707 | 22382807 |
| Marker74522 | LG5 | 22461880 | 22461830 | 22461930 |
| Marker74536 | LG5 | 22469259 | 22469209 | 22469309 |
| Marker74543 | LG5 | 22473496 | 22473446 | 22473546 |
| Marker74566 | LG5 | 22508027 | 22507977 | 22508077 |
| Marker74574 | LG5 | 22524108 | 22524058 | 22524158 |
| Marker74576 | LG5 | 22525257 | 22525207 | 22525307 |
| Marker74580 | LG5 | 22528665 | 22528615 | 22528715 |
| Marker74588 | LG5 | 22534603 | 22534553 | 22534653 |
| Marker74590 | LG5 | 22535317 | 22535267 | 22535367 |
| Marker74603 | LG5 | 22550185 | 22550135 | 22550235 |
| Marker74606 | LG5 | 22556388 | 22556338 | 22556438 |
| Marker74613 | LG5 | 22573769 | 22573719 | 22573819 |
| Marker74615 | LG5 | 22574996 | 22574946 | 22575046 |
| Marker74663 | LG5 | 22634776 | 22634726 | 22634826 |
| Marker74668 | LG5 | 22643722 | 22643672 | 22643772 |
| Marker74669 | LG5 | 22643916 | 22643866 | 22643966 |
| Marker74725 | LG5 | 22734306 | 22734256 | 22734356 |
| Marker74805 | LG5 | 22789634 | 22789584 | 22789684 |
| Marker74804 | LG5 | 22789855 | 22789805 | 22789905 |
| Marker74810 | LG5 | 22821321 | 22821271 | 22821371 |
| Marker74823 | LG5 | 22831292 | 22831242 | 22831342 |
| Marker74841 | LG5 | 22854222 | 22854172 | 22854272 |
| Marker74843 | LG5 | 22861592 | 22861542 | 22861642 |
| Marker74857 | LG5 | 22881751 | 22881701 | 22881801 |
| Marker74858 | LG5 | 22882054 | 22882004 | 22882104 |
| Marker74859 | LG5 | 22888435 | 22888385 | 22888485 |
| Marker74860 | LG5 | 22893014 | 22892964 | 22893064 |
| Marker74876 | LG5 | 22910305 | 22910255 | 22910355 |
| Marker74901 | LG5 | 22945690 | 22945640 | 22945740 |
| Marker74922 | LG5 | 22964374 | 22964324 | 22964424 |
| Marker74929 | LG5 | 22979008 | 22978958 | 22979058 |
| Marker74952 | LG5 | 23009148 | 23009098 | 23009198 |
| Marker74956 | LG5 | 23009524 | 23009474 | 23009574 |
| Marker74960 | LG5 | 23017524 | 23017474 | 23017574 |
| Marker74974 | LG5 | 23034329 | 23034279 | 23034379 |
| Marker74989 | LG5 | 23050343 | 23050293 | 23050393 |
| Marker75697 | LG5 | 23078072 | 23078022 | 23078122 |
| Marker75721 | LG5 | 23115432 | 23115382 | 23115482 |
| Marker75741 | LG5 | 23146613 | 23146563 | 23146663 |
| Marker75742 | LG5 | 23149664 | 23149614 | 23149714 |
| Marker75750 | LG5 | 23165874 | 23165824 | 23165924 |
| Marker75756 | LG5 | 23174742 | 23174692 | 23174792 |
| Marker75205 | LG5 | 23233854 | 23233804 | 23233904 |
| Marker75155 | LG5 | 23296855 | 23296805 | 23296905 |
| Marker75044 | LG5 | 23366473 | 23366423 | 23366523 |
| Marker75014 | LG5 | 23387527 | 23387477 | 23387577 |
| Marker75110 | LG5 | 23454112 | 23454062 | 23454162 |
| Marker75121 | LG5 | 23467576 | 23467526 | 23467626 |
| Marker75131 | LG5 | 23479199 | 23479149 | 23479249 |
| Marker75143 | LG5 | 23487251 | 23487201 | 23487301 |
| Marker75145 | LG5 | 23508038 | 23507988 | 23508088 |
| Marker75149 | LG5 | 23512628 | 23512578 | 23512678 |
| Marker75150 | LG5 | 23512851 | 23512801 | 23512901 |
| Marker75153 | LG5 | 23515515 | 23515465 | 23515565 |
| Marker75154 | LG5 | 23519272 | 23519222 | 23519322 |
| Marker75659 | LG5 | 23619472 | 23619422 | 23619522 |
| Marker75658 | LG5 | 23620582 | 23620532 | 23620632 |
| Marker75597 | LG5 | 23638339 | 23638289 | 23638389 |
| Marker75611 | LG5 | 23655873 | 23655823 | 23655923 |
| Marker75614 | LG5 | 23662189 | 23662139 | 23662239 |
| Marker75636 | LG5 | 23707196 | 23707146 | 23707246 |
| Marker75646 | LG5 | 23740356 | 23740306 | 23740406 |
| Marker76301 | LG5 | 23829625 | 23829575 | 23829675 |
| Marker76294 | LG5 | 23841477 | 23841427 | 23841527 |
| Marker76275 | LG5 | 23860757 | 23860707 | 23860807 |
| Marker76268 | LG5 | 23876949 | 23876899 | 23876999 |
| Marker76261 | LG5 | 23932866 | 23932816 | 23932916 |
| Marker76233 | LG5 | 23964035 | 23963985 | 23964085 |
| Marker76228 | LG5 | 23991429 | 23991379 | 23991479 |
| Marker76218 | LG5 | 23998781 | 23998731 | 23998831 |
| Marker76203 | LG5 | 24004816 | 24004766 | 24004866 |
| Marker76181 | LG5 | 24048522 | 24048472 | 24048572 |
| Marker76180 | LG5 | 24050564 | 24050514 | 24050614 |
| Marker76167 | LG5 | 24073960 | 24073910 | 24074010 |
| Marker76128 | LG5 | 24200861 | 24200811 | 24200911 |
| Marker76108 | LG5 | 24225234 | 24225184 | 24225284 |
| Marker76098 | LG5 | 24235964 | 24235914 | 24236014 |
| Marker76094 | LG5 | 24237863 | 24237813 | 24237913 |
| Marker76072 | LG5 | 24278510 | 24278460 | 24278560 |
| Marker76070 | LG5 | 24291465 | 24291415 | 24291515 |
| Marker76061 | LG5 | 24306473 | 24306423 | 24306523 |
| Marker76059 | LG5 | 24309192 | 24309142 | 24309242 |
| Marker76054 | LG5 | 24313228 | 24313178 | 24313278 |
| Marker76051 | LG5 | 24320704 | 24320654 | 24320754 |
| Marker76040 | LG5 | 24335881 | 24335831 | 24335931 |
| Marker76035 | LG5 | 24342227 | 24342177 | 24342277 |
| Marker76028 | LG5 | 24346735 | 24346685 | 24346785 |
| Marker76001 | LG5 | 24397951 | 24397901 | 24398001 |
| Marker75988 | LG5 | 24446079 | 24446029 | 24446129 |
| Marker75976 | LG5 | 24484563 | 24484513 | 24484613 |
| Marker75953 | LG5 | 24508747 | 24508697 | 24508797 |
| Marker75928 | LG5 | 24572839 | 24572789 | 24572889 |
| Marker75925 | LG5 | 24573049 | 24572999 | 24573099 |
| Marker75924 | LG5 | 24573811 | 24573761 | 24573861 |
| Marker75881 | LG5 | 24637854 | 24637804 | 24637904 |
| Marker75879 | LG5 | 24641940 | 24641890 | 24641990 |
| Marker75846 | LG5 | 24701447 | 24701397 | 24701497 |
| Marker75844 | LG5 | 24712241 | 24712191 | 24712291 |
| Marker75838 | LG5 | 24733666 | 24733616 | 24733716 |
| Marker75834 | LG5 | 24765523 | 24765473 | 24765573 |
| Marker75833 | LG5 | 24765660 | 24765610 | 24765710 |
| Marker75832 | LG5 | 24781624 | 24781574 | 24781674 |
| Marker75806 | LG5 | 24841226 | 24841176 | 24841276 |
| Marker75801 | LG5 | 24862012 | 24861962 | 24862062 |
| Marker75790 | LG5 | 24890688 | 24890638 | 24890738 |
| Marker75788 | LG5 | 24899127 | 24899077 | 24899177 |
| Marker75787 | LG5 | 24899365 | 24899315 | 24899415 |
| Marker75786 | LG5 | 24901999 | 24901949 | 24902049 |
| Marker75781 | LG5 | 24912452 | 24912402 | 24912502 |
| Marker75775 | LG5 | 24953838 | 24953788 | 24953888 |
| Marker75774 | LG5 | 24983096 | 24983046 | 24983146 |
| Marker75768 | LG5 | 25059527 | 25059477 | 25059577 |
| Marker75760 | LG5 | 25076594 | 25076544 | 25076644 |
| Marker76890 | LG5 | 25114829 | 25114779 | 25114879 |
| Marker76884 | LG5 | 25123412 | 25123362 | 25123462 |
| Marker76868 | LG5 | 25167115 | 25167065 | 25167165 |
| Marker76867 | LG5 | 25182491 | 25182441 | 25182541 |
| Marker76862 | LG5 | 25199201 | 25199151 | 25199251 |
| Marker76859 | LG5 | 25215563 | 25215513 | 25215613 |
| Marker76850 | LG5 | 25243389 | 25243339 | 25243439 |
| Marker76821 | LG5 | 25346510 | 25346460 | 25346560 |
| Marker76816 | LG5 | 25359088 | 25359038 | 25359138 |
| Marker76792 | LG5 | 25425420 | 25425370 | 25425470 |
| Marker76787 | LG5 | 25434185 | 25434135 | 25434235 |
| Marker76784 | LG5 | 25449695 | 25449645 | 25449745 |
| Marker76765 | LG5 | 25469566 | 25469516 | 25469616 |
| Marker76762 | LG5 | 25480347 | 25480297 | 25480397 |
| Marker76745 | LG5 | 25580815 | 25580765 | 25580865 |
| Marker76743 | LG5 | 25581062 | 25581012 | 25581112 |
| Marker76733 | LG5 | 25664100 | 25664050 | 25664150 |
| Marker76731 | LG5 | 25674831 | 25674781 | 25674881 |
| Marker76730 | LG5 | 25675015 | 25674965 | 25675065 |
| Marker76722 | LG5 | 25750535 | 25750485 | 25750585 |
| Marker76721 | LG5 | 25750806 | 25750756 | 25750856 |
| Marker76700 | LG5 | 25838073 | 25838023 | 25838123 |
| Marker76697 | LG5 | 25839426 | 25839376 | 25839476 |
| Marker76694 | LG5 | 25852963 | 25852913 | 25853013 |
| Marker76680 | LG5 | 25878066 | 25878016 | 25878116 |
| Marker76665 | LG5 | 25939665 | 25939615 | 25939715 |
| Marker76661 | LG5 | 25940623 | 25940573 | 25940673 |
| Marker76660 | LG5 | 25940891 | 25940841 | 25940941 |
| Marker76659 | LG5 | 25945497 | 25945447 | 25945547 |
| Marker76653 | LG5 | 25948862 | 25948812 | 25948912 |
| Marker76652 | LG5 | 25949118 | 25949068 | 25949168 |
| Marker76642 | LG5 | 25964170 | 25964120 | 25964220 |
| Marker76639 | LG5 | 25967519 | 25967469 | 25967569 |
| Marker76629 | LG5 | 25995682 | 25995632 | 25995732 |
| Marker63442 | LG5 | 26012054 | 26012004 | 26012104 |
| Marker76614 | LG5 | 26105936 | 26105886 | 26105986 |
| Marker76598 | LG5 | 26146560 | 26146510 | 26146610 |
| Marker76583 | LG5 | 26165609 | 26165559 | 26165659 |
| Marker76575 | LG5 | 26196090 | 26196040 | 26196140 |
| Marker76565 | LG5 | 26208084 | 26208034 | 26208134 |
| Marker76560 | LG5 | 26209167 | 26209117 | 26209217 |
| Marker76550 | LG5 | 26229485 | 26229435 | 26229535 |
| Marker76531 | LG5 | 26256193 | 26256143 | 26256243 |
| Marker76516 | LG5 | 26276335 | 26276285 | 26276385 |
| Marker76501 | LG5 | 26285641 | 26285591 | 26285691 |
| Marker76500 | LG5 | 26285830 | 26285780 | 26285880 |
| Marker76476 | LG5 | 26304864 | 26304814 | 26304914 |
| Marker76454 | LG5 | 26360311 | 26360261 | 26360361 |
| Marker76450 | LG5 | 26372117 | 26372067 | 26372167 |
| Marker76436 | LG5 | 26414295 | 26414245 | 26414345 |
| Marker76418 | LG5 | 26461233 | 26461183 | 26461283 |
| Marker24860 | LG5 | 26484720 | 26484670 | 26484770 |
| Marker76394 | LG5 | 26488704 | 26488654 | 26488754 |
| Marker76386 | LG5 | 26506463 | 26506413 | 26506513 |
| Marker76382 | LG5 | 26514627 | 26514577 | 26514677 |
| Marker76373 | LG5 | 26563869 | 26563819 | 26563919 |
| Marker76368 | LG5 | 26590336 | 26590286 | 26590386 |
| Marker76359 | LG5 | 26634320 | 26634270 | 26634370 |
| Marker76358 | LG5 | 26636421 | 26636371 | 26636471 |
| Marker76357 | LG5 | 26639956 | 26639906 | 26640006 |
| Marker76356 | LG5 | 26640772 | 26640722 | 26640822 |
| Marker76354 | LG5 | 26662247 | 26662197 | 26662297 |
| Marker77450 | LG6 | 37941 | 37891 | 37991 |
| Marker77462 | LG6 | 50964 | 50914 | 51014 |
| Marker77493 | LG6 | 103642 | 103592 | 103692 |
| Marker77503 | LG6 | 115163 | 115113 | 115213 |
| Marker77504 | LG6 | 116620 | 116570 | 116670 |
| Marker77506 | LG6 | 118639 | 118589 | 118689 |
| Marker77537 | LG6 | 164277 | 164227 | 164327 |
| Marker77543 | LG6 | 176271 | 176221 | 176321 |
| Marker77555 | LG6 | 199256 | 199206 | 199306 |
| Marker77560 | LG6 | 212757 | 212707 | 212807 |
| Marker77577 | LG6 | 234124 | 234074 | 234174 |
| Marker77591 | LG6 | 242038 | 241988 | 242088 |
| Marker77606 | LG6 | 260511 | 260461 | 260561 |
| Marker77615 | LG6 | 291989 | 291939 | 292039 |
| Marker77622 | LG6 | 300431 | 300381 | 300481 |
| Marker77627 | LG6 | 317284 | 317234 | 317334 |
| Marker77725 | LG6 | 474378 | 474328 | 474428 |
| Marker77350 | LG6 | 640312 | 640262 | 640362 |
| Marker77331 | LG6 | 660704 | 660654 | 660754 |
| Marker77323 | LG6 | 665269 | 665219 | 665319 |
| Marker77316 | LG6 | 691112 | 691062 | 691162 |
| Marker77312 | LG6 | 705534 | 705484 | 705584 |
| Marker77308 | LG6 | 710688 | 710638 | 710738 |
| Marker77304 | LG6 | 712651 | 712601 | 712701 |
| Marker77291 | LG6 | 728565 | 728515 | 728615 |
| Marker77281 | LG6 | 732763 | 732713 | 732813 |
| Marker77278 | LG6 | 732956 | 732906 | 733006 |
| Marker77276 | LG6 | 734623 | 734573 | 734673 |
| Marker77270 | LG6 | 740421 | 740371 | 740471 |
| Marker77253 | LG6 | 762133 | 762083 | 762183 |
| Marker77241 | LG6 | 773677 | 773627 | 773727 |
| Marker77240 | LG6 | 773938 | 773888 | 773988 |
| Marker77237 | LG6 | 787810 | 787760 | 787860 |
| Marker77216 | LG6 | 824814 | 824764 | 824864 |
| Marker77213 | LG6 | 839657 | 839607 | 839707 |
| Marker77211 | LG6 | 847602 | 847552 | 847652 |
| Marker77199 | LG6 | 851851 | 851801 | 851901 |
| Marker77187 | LG6 | 866007 | 865957 | 866057 |
| Marker77185 | LG6 | 876619 | 876569 | 876669 |
| Marker77161 | LG6 | 924038 | 923988 | 924088 |
| Marker77156 | LG6 | 926470 | 926420 | 926520 |
| Marker77138 | LG6 | 957374 | 957324 | 957424 |
| Marker77085 | LG6 | 1020637 | 1020587 | 1020687 |
| Marker77060 | LG6 | 1081160 | 1081110 | 1081210 |
| Marker77002 | LG6 | 1167000 | 1166950 | 1167050 |
| Marker76999 | LG6 | 1176561 | 1176511 | 1176611 |
| Marker76993 | LG6 | 1181257 | 1181207 | 1181307 |
| Marker76931 | LG6 | 1272385 | 1272335 | 1272435 |
| Marker76920 | LG6 | 1288827 | 1288777 | 1288877 |
| Marker76901 | LG6 | 1319800 | 1319750 | 1319850 |
| Marker77824 | LG6 | 1370281 | 1370231 | 1370331 |
| Marker77831 | LG6 | 1376929 | 1376879 | 1376979 |
| Marker77853 | LG6 | 1407769 | 1407719 | 1407819 |
| Marker77858 | LG6 | 1410678 | 1410628 | 1410728 |
| Marker77859 | LG6 | 1416062 | 1416012 | 1416112 |
| Marker77861 | LG6 | 1421209 | 1421159 | 1421259 |
| Marker77896 | LG6 | 1460991 | 1460941 | 1461041 |
| Marker77900 | LG6 | 1467331 | 1467281 | 1467381 |
| Marker77917 | LG6 | 1478004 | 1477954 | 1478054 |
| Marker77966 | LG6 | 1540966 | 1540916 | 1541016 |
| Marker77991 | LG6 | 1603054 | 1603004 | 1603104 |
| Marker78310 | LG6 | 1645802 | 1645752 | 1645852 |
| Marker78304 | LG6 | 1649385 | 1649335 | 1649435 |
| Marker78300 | LG6 | 1654414 | 1654364 | 1654464 |
| Marker78298 | LG6 | 1655337 | 1655287 | 1655387 |
| Marker78281 | LG6 | 1667066 | 1667016 | 1667116 |
| Marker78264 | LG6 | 1675734 | 1675684 | 1675784 |
| Marker78255 | LG6 | 1687956 | 1687906 | 1688006 |
| Marker78232 | LG6 | 1720955 | 1720905 | 1721005 |
| Marker78194 | LG6 | 1788733 | 1788683 | 1788783 |
| Marker78175 | LG6 | 1838670 | 1838620 | 1838720 |
| Marker78159 | LG6 | 1888396 | 1888346 | 1888446 |
| Marker78141 | LG6 | 1920732 | 1920682 | 1920782 |
| Marker78139 | LG6 | 1923410 | 1923360 | 1923460 |
| Marker78116 | LG6 | 1945994 | 1945944 | 1946044 |
| Marker78081 | LG6 | 2028010 | 2027960 | 2028060 |
| Marker78070 | LG6 | 2034146 | 2034096 | 2034196 |
| Marker78064 | LG6 | 2042618 | 2042568 | 2042668 |
| Marker78030 | LG6 | 2141446 | 2141396 | 2141496 |
| Marker79557 | LG6 | 2197158 | 2197108 | 2197208 |
| Marker79544 | LG6 | 2245303 | 2245253 | 2245353 |
| Marker79537 | LG6 | 2254408 | 2254358 | 2254458 |
| Marker79518 | LG6 | 2292498 | 2292448 | 2292548 |
| Marker79510 | LG6 | 2303828 | 2303778 | 2303878 |
| Marker79496 | LG6 | 2319232 | 2319182 | 2319282 |
| Marker79476 | LG6 | 2407616 | 2407566 | 2407666 |
| Marker79454 | LG6 | 2438984 | 2438934 | 2439034 |
| Marker79453 | LG6 | 2439045 | 2438995 | 2439095 |
| Marker79441 | LG6 | 2459447 | 2459397 | 2459497 |
| Marker79423 | LG6 | 2489071 | 2489021 | 2489121 |
| Marker79421 | LG6 | 2491286 | 2491236 | 2491336 |
| Marker79414 | LG6 | 2507934 | 2507884 | 2507984 |
| Marker79398 | LG6 | 2517507 | 2517457 | 2517557 |
| Marker79378 | LG6 | 2550223 | 2550173 | 2550273 |
| Marker79376 | LG6 | 2555090 | 2555040 | 2555140 |
| Marker79371 | LG6 | 2575085 | 2575035 | 2575135 |
| Marker79336 | LG6 | 2652181 | 2652131 | 2652231 |
| Marker79283 | LG6 | 2749497 | 2749447 | 2749547 |
| Marker79275 | LG6 | 2764762 | 2764712 | 2764812 |
| Marker79239 | LG6 | 2822068 | 2822018 | 2822118 |
| Marker79233 | LG6 | 2826512 | 2826462 | 2826562 |
| Marker79227 | LG6 | 2840818 | 2840768 | 2840868 |
| Marker79211 | LG6 | 2857186 | 2857136 | 2857236 |
| Marker79182 | LG6 | 2873814 | 2873764 | 2873864 |
| Marker79146 | LG6 | 2941994 | 2941944 | 2942044 |
| Marker79133 | LG6 | 2958954 | 2958904 | 2959004 |
| Marker79130 | LG6 | 2968499 | 2968449 | 2968549 |
| Marker79125 | LG6 | 2972552 | 2972502 | 2972602 |
| Marker79081 | LG6 | 3035425 | 3035375 | 3035475 |
| Marker79072 | LG6 | 3040653 | 3040603 | 3040703 |
| Marker79062 | LG6 | 3061914 | 3061864 | 3061964 |
| Marker79046 | LG6 | 3076229 | 3076179 | 3076279 |
| Marker78932 | LG6 | 3187663 | 3187613 | 3187713 |
| Marker78968 | LG6 | 3264440 | 3264390 | 3264490 |
| Marker78971 | LG6 | 3268206 | 3268156 | 3268256 |
| Marker78978 | LG6 | 3280113 | 3280063 | 3280163 |
| Marker78998 | LG6 | 3300305 | 3300255 | 3300355 |
| Marker78840 | LG6 | 3375699 | 3375649 | 3375749 |
| Marker78823 | LG6 | 3395843 | 3395793 | 3395893 |
| Marker78797 | LG6 | 3415538 | 3415488 | 3415588 |
| Marker78779 | LG6 | 3451063 | 3451013 | 3451113 |
| Marker78762 | LG6 | 3476309 | 3476259 | 3476359 |
| Marker78761 | LG6 | 3476333 | 3476283 | 3476383 |
| Marker78750 | LG6 | 3480653 | 3480603 | 3480703 |
| Marker78717 | LG6 | 3529839 | 3529789 | 3529889 |
| Marker78715 | LG6 | 3530949 | 3530899 | 3530999 |
| Marker78713 | LG6 | 3532522 | 3532472 | 3532572 |
| Marker78697 | LG6 | 3561912 | 3561862 | 3561962 |
| Marker78618 | LG6 | 3670768 | 3670718 | 3670818 |
| Marker78612 | LG6 | 3720739 | 3720689 | 3720789 |
| Marker78603 | LG6 | 3737500 | 3737450 | 3737550 |
| Marker78587 | LG6 | 3764967 | 3764917 | 3765017 |
| Marker78586 | LG6 | 3765178 | 3765128 | 3765228 |
| Marker78557 | LG6 | 3809720 | 3809670 | 3809770 |
| Marker78553 | LG6 | 3816355 | 3816305 | 3816405 |
| Marker78542 | LG6 | 3824050 | 3824000 | 3824100 |
| Marker78538 | LG6 | 3837507 | 3837457 | 3837557 |
| Marker78536 | LG6 | 3851463 | 3851413 | 3851513 |
| Marker78520 | LG6 | 3879575 | 3879525 | 3879625 |
| Marker78518 | LG6 | 3883106 | 3883056 | 3883156 |
| Marker78497 | LG6 | 3917498 | 3917448 | 3917548 |
| Marker78492 | LG6 | 3924861 | 3924811 | 3924911 |
| Marker78441 | LG6 | 3959707 | 3959657 | 3959757 |
| Marker78417 | LG6 | 3996277 | 3996227 | 3996327 |
| Marker78384 | LG6 | 4021121 | 4021071 | 4021171 |
| Marker78382 | LG6 | 4023527 | 4023477 | 4023577 |
| Marker78381 | LG6 | 4027225 | 4027175 | 4027275 |
| Marker78369 | LG6 | 4046865 | 4046815 | 4046915 |
| Marker78357 | LG6 | 4062349 | 4062299 | 4062399 |
| Marker78349 | LG6 | 4066121 | 4066071 | 4066171 |
| Marker78346 | LG6 | 4072410 | 4072360 | 4072460 |
| Marker114432 | LG6 | 4202848 | 4202798 | 4202898 |
| Marker114434 | LG6 | 4205287 | 4205237 | 4205337 |
| Marker114440 | LG6 | 4224605 | 4224555 | 4224655 |
| Marker114501 | LG6 | 4333727 | 4333677 | 4333777 |
| Marker114505 | LG6 | 4356621 | 4356571 | 4356671 |
| Marker79992 | LG6 | 4412036 | 4411986 | 4412086 |
| Marker79980 | LG6 | 4435084 | 4435034 | 4435134 |
| Marker79965 | LG6 | 4468565 | 4468515 | 4468615 |
| Marker79964 | LG6 | 4469486 | 4469436 | 4469536 |
| Marker79960 | LG6 | 4470785 | 4470735 | 4470835 |
| Marker79940 | LG6 | 4483074 | 4483024 | 4483124 |
| Marker79921 | LG6 | 4495954 | 4495904 | 4496004 |
| Marker79901 | LG6 | 4519839 | 4519789 | 4519889 |
| Marker79882 | LG6 | 4540268 | 4540218 | 4540318 |
| Marker79879 | LG6 | 4548889 | 4548839 | 4548939 |
| Marker79865 | LG6 | 4569614 | 4569564 | 4569664 |
| Marker79863 | LG6 | 4583172 | 4583122 | 4583222 |
| Marker79853 | LG6 | 4590195 | 4590145 | 4590245 |
| Marker79845 | LG6 | 4660191 | 4660141 | 4660241 |
| Marker79806 | LG6 | 4694915 | 4694865 | 4694965 |
| Marker79795 | LG6 | 4714911 | 4714861 | 4714961 |
| Marker79783 | LG6 | 4732724 | 4732674 | 4732774 |
| Marker79776 | LG6 | 4760957 | 4760907 | 4761007 |
| Marker79735 | LG6 | 4843975 | 4843925 | 4844025 |
| Marker79694 | LG6 | 4932506 | 4932456 | 4932556 |
| Marker79692 | LG6 | 4932581 | 4932531 | 4932631 |
| Marker79684 | LG6 | 4948325 | 4948275 | 4948375 |
| Marker79681 | LG6 | 4954246 | 4954196 | 4954296 |
| Marker79678 | LG6 | 4957891 | 4957841 | 4957941 |
| Marker79652 | LG6 | 5005900 | 5005850 | 5005950 |
| Marker80096 | LG6 | 5096753 | 5096703 | 5096803 |
| Marker80092 | LG6 | 5099415 | 5099365 | 5099465 |
| Marker80091 | LG6 | 5101624 | 5101574 | 5101674 |
| Marker79637 | LG6 | 5138471 | 5138421 | 5138521 |
| Marker80111 | LG6 | 5206172 | 5206122 | 5206222 |
| Marker80062 | LG6 | 5278537 | 5278487 | 5278587 |
| Marker80061 | LG6 | 5282867 | 5282817 | 5282917 |
| Marker80042 | LG6 | 5341704 | 5341654 | 5341754 |
| Marker80034 | LG6 | 5370919 | 5370869 | 5370969 |
| Marker80009 | LG6 | 5395601 | 5395551 | 5395651 |
| Marker80718 | LG6 | 5452319 | 5452269 | 5452369 |
| Marker80715 | LG6 | 5456694 | 5456644 | 5456744 |
| Marker80702 | LG6 | 5522262 | 5522212 | 5522312 |
| Marker80676 | LG6 | 5682453 | 5682403 | 5682503 |
| Marker80674 | LG6 | 5682652 | 5682602 | 5682702 |
| Marker80568 | LG6 | 5801186 | 5801136 | 5801236 |
| Marker80567 | LG6 | 5812114 | 5812064 | 5812164 |
| Marker80487 | LG6 | 5926087 | 5926037 | 5926137 |
| Marker80464 | LG6 | 6013504 | 6013454 | 6013554 |
| Marker80450 | LG6 | 6040335 | 6040285 | 6040385 |
| Marker80397 | LG6 | 6067475 | 6067425 | 6067525 |
| Marker80362 | LG6 | 6170075 | 6170025 | 6170125 |
| Marker80315 | LG6 | 6260834 | 6260784 | 6260884 |
| Marker80313 | LG6 | 6261019 | 6260969 | 6261069 |
| Marker80292 | LG6 | 6284725 | 6284675 | 6284775 |
| Marker80289 | LG6 | 6290373 | 6290323 | 6290423 |
| Marker80281 | LG6 | 6298253 | 6298203 | 6298303 |
| Marker80260 | LG6 | 6324894 | 6324844 | 6324944 |
| Marker80231 | LG6 | 6365881 | 6365831 | 6365931 |
| Marker80179 | LG6 | 6407284 | 6407234 | 6407334 |
| Marker80168 | LG6 | 6418055 | 6418005 | 6418105 |
| Marker80160 | LG6 | 6423335 | 6423285 | 6423385 |
| Marker80159 | LG6 | 6423486 | 6423436 | 6423536 |
| Marker80157 | LG6 | 6431745 | 6431695 | 6431795 |
| Marker80142 | LG6 | 6447844 | 6447794 | 6447894 |
| Marker80137 | LG6 | 6459558 | 6459508 | 6459608 |
| Marker80128 | LG6 | 6464435 | 6464385 | 6464485 |
| Marker80122 | LG6 | 6469288 | 6469238 | 6469338 |
| Marker81050 | LG6 | 6484887 | 6484837 | 6484937 |
| Marker81045 | LG6 | 6506368 | 6506318 | 6506418 |
| Marker81037 | LG6 | 6523270 | 6523220 | 6523320 |
| Marker81035 | LG6 | 6524381 | 6524331 | 6524431 |
| Marker81032 | LG6 | 6524642 | 6524592 | 6524692 |
| Marker81030 | LG6 | 6528829 | 6528779 | 6528879 |
| Marker81015 | LG6 | 6559353 | 6559303 | 6559403 |
| Marker80999 | LG6 | 6577340 | 6577290 | 6577390 |
| Marker80998 | LG6 | 6578784 | 6578734 | 6578834 |
| Marker80965 | LG6 | 6603986 | 6603936 | 6604036 |
| Marker80920 | LG6 | 6665513 | 6665463 | 6665563 |
| Marker80918 | LG6 | 6665757 | 6665707 | 6665807 |
| Marker80915 | LG6 | 6670825 | 6670775 | 6670875 |
| Marker80848 | LG6 | 6731404 | 6731354 | 6731454 |
| Marker80823 | LG6 | 6736101 | 6736051 | 6736151 |
| Marker80789 | LG6 | 6743590 | 6743540 | 6743640 |
| Marker81072 | LG6 | 6863108 | 6863058 | 6863158 |
| Marker81166 | LG6 | 6924797 | 6924747 | 6924847 |
| Marker81130 | LG6 | 6969677 | 6969627 | 6969727 |
| Marker81120 | LG6 | 6982301 | 6982251 | 6982351 |
| Marker81184 | LG6 | 7009240 | 7009190 | 7009290 |
| Marker81192 | LG6 | 7039362 | 7039312 | 7039412 |
| Marker81215 | LG6 | 7056936 | 7056886 | 7056986 |
| Marker81244 | LG6 | 7121573 | 7121523 | 7121623 |
| Marker81247 | LG6 | 7125314 | 7125264 | 7125364 |
| Marker81316 | LG6 | 7217078 | 7217028 | 7217128 |
| Marker81322 | LG6 | 7217579 | 7217529 | 7217629 |
| Marker81378 | LG6 | 7331781 | 7331731 | 7331831 |
| Marker81383 | LG6 | 7358814 | 7358764 | 7358864 |
| Marker81439 | LG6 | 7410427 | 7410377 | 7410477 |
| Marker81457 | LG6 | 7454957 | 7454907 | 7455007 |
| Marker81478 | LG6 | 7488356 | 7488306 | 7488406 |
| Marker81840 | LG6 | 7569668 | 7569618 | 7569718 |
| Marker81823 | LG6 | 7591969 | 7591919 | 7592019 |
| Marker81800 | LG6 | 7626367 | 7626317 | 7626417 |
| Marker81776 | LG6 | 7660294 | 7660244 | 7660344 |
| Marker81775 | LG6 | 7660488 | 7660438 | 7660538 |
| Marker81764 | LG6 | 7663511 | 7663461 | 7663561 |
| Marker81743 | LG6 | 7689370 | 7689320 | 7689420 |
| Marker81708 | LG6 | 7723281 | 7723231 | 7723331 |
| Marker81690 | LG6 | 7744481 | 7744431 | 7744531 |
| Marker81689 | LG6 | 7749688 | 7749638 | 7749738 |
| Marker81686 | LG6 | 7749922 | 7749872 | 7749972 |
| Marker81659 | LG6 | 7799918 | 7799868 | 7799968 |
| Marker81631 | LG6 | 7845677 | 7845627 | 7845727 |
| Marker81627 | LG6 | 7854680 | 7854630 | 7854730 |
| Marker81611 | LG6 | 7877966 | 7877916 | 7878016 |
| Marker81597 | LG6 | 7886599 | 7886549 | 7886649 |
| Marker81594 | LG6 | 7896143 | 7896093 | 7896193 |
| Marker81553 | LG6 | 7949547 | 7949497 | 7949597 |
| Marker81530 | LG6 | 7992543 | 7992493 | 7992593 |
| Marker81528 | LG6 | 8006394 | 8006344 | 8006444 |
| Marker82259 | LG6 | 8056916 | 8056866 | 8056966 |
| Marker82238 | LG6 | 8077169 | 8077119 | 8077219 |
| Marker82236 | LG6 | 8082756 | 8082706 | 8082806 |
| Marker82231 | LG6 | 8083469 | 8083419 | 8083519 |
| Marker82224 | LG6 | 8091247 | 8091197 | 8091297 |
| Marker82200 | LG6 | 8112168 | 8112118 | 8112218 |
| Marker82193 | LG6 | 8124980 | 8124930 | 8125030 |
| Marker82174 | LG6 | 8142062 | 8142012 | 8142112 |
| Marker121772 | LG6 | 8152565 | 8152515 | 8152615 |
| Marker82159 | LG6 | 8158029 | 8157979 | 8158079 |
| Marker53434 | LG6 | 8205601 | 8205551 | 8205651 |
| Marker12184 | LG6 | 8207464 | 8207414 | 8207514 |
| Marker81945 | LG6 | 8244837 | 8244787 | 8244887 |
| Marker81932 | LG6 | 8262433 | 8262383 | 8262483 |
| Marker81925 | LG6 | 8285445 | 8285395 | 8285495 |
| Marker81917 | LG6 | 8293974 | 8293924 | 8294024 |
| Marker81903 | LG6 | 8340284 | 8340234 | 8340334 |
| Marker81888 | LG6 | 8360061 | 8360011 | 8360111 |
| Marker82341 | LG6 | 8383030 | 8382980 | 8383080 |
| Marker82339 | LG6 | 8384483 | 8384433 | 8384533 |
| Marker82317 | LG6 | 8401526 | 8401476 | 8401576 |
| Marker82287 | LG6 | 8425209 | 8425159 | 8425259 |
| Marker82284 | LG6 | 8435038 | 8434988 | 8435088 |
| Marker81976 | LG6 | 8456014 | 8455964 | 8456064 |
| Marker82013 | LG6 | 8552958 | 8552908 | 8553008 |
| Marker82015 | LG6 | 8553167 | 8553117 | 8553217 |
| Marker82019 | LG6 | 8579398 | 8579348 | 8579448 |
| Marker82020 | LG6 | 8584256 | 8584206 | 8584306 |
| Marker82036 | LG6 | 8605380 | 8605330 | 8605430 |
| Marker78710 | LG6 | 8618143 | 8618093 | 8618193 |
| Marker82058 | LG6 | 8641542 | 8641492 | 8641592 |
| Marker82073 | LG6 | 8663785 | 8663735 | 8663835 |
| Marker82083 | LG6 | 8673688 | 8673638 | 8673738 |
| Marker82373 | LG6 | 8729618 | 8729568 | 8729668 |
| Marker82405 | LG6 | 8863822 | 8863772 | 8863872 |
| Marker82409 | LG6 | 8867891 | 8867841 | 8867941 |
| Marker82672 | LG6 | 9093065 | 9093015 | 9093115 |
| Marker82707 | LG6 | 9144073 | 9144023 | 9144123 |
| Marker82731 | LG6 | 9151334 | 9151284 | 9151384 |
| Marker82436 | LG6 | 9189347 | 9189297 | 9189397 |
| Marker82441 | LG6 | 9191158 | 9191108 | 9191208 |
| Marker82476 | LG6 | 9242757 | 9242707 | 9242807 |
| Marker82486 | LG6 | 9287311 | 9287261 | 9287361 |
| Marker82509 | LG6 | 9302773 | 9302723 | 9302823 |
| Marker82591 | LG6 | 9596270 | 9596220 | 9596320 |
| Marker82590 | LG6 | 9597462 | 9597412 | 9597512 |
| Marker82895 | LG6 | 9865919 | 9865869 | 9865969 |
| Marker82942 | LG6 | 9928748 | 9928698 | 9928798 |
| Marker82953 | LG6 | 9942603 | 9942553 | 9942653 |
| Marker82957 | LG6 | 9949915 | 9949865 | 9949965 |
| Marker82972 | LG6 | 9984203 | 9984153 | 9984253 |
| Marker83004 | LG6 | 10055504 | 10055454 | 10055554 |
| Marker83024 | LG6 | 10065266 | 10065216 | 10065316 |
| Marker83066 | LG6 | 10125014 | 10124964 | 10125064 |
| Marker83072 | LG6 | 10136458 | 10136408 | 10136508 |
| Marker117993 | LG6 | 10215082 | 10215032 | 10215132 |
| Marker118001 | LG6 | 10249932 | 10249882 | 10249982 |
| Marker83402 | LG6 | 10323202 | 10323152 | 10323252 |
| Marker83430 | LG6 | 10396655 | 10396605 | 10396705 |
| Marker83438 | LG6 | 10405909 | 10405859 | 10405959 |
| Marker83441 | LG6 | 10406135 | 10406085 | 10406185 |
| Marker83468 | LG6 | 10457747 | 10457697 | 10457797 |
| Marker83498 | LG6 | 10525692 | 10525642 | 10525742 |
| Marker83506 | LG6 | 10538117 | 10538067 | 10538167 |
| Marker83508 | LG6 | 10543899 | 10543849 | 10543949 |
| Marker83517 | LG6 | 10585271 | 10585221 | 10585321 |
| Marker83518 | LG6 | 10604871 | 10604821 | 10604921 |
| Marker83524 | LG6 | 10611541 | 10611491 | 10611591 |
| Marker83528 | LG6 | 10618698 | 10618648 | 10618748 |
| Marker83550 | LG6 | 10693293 | 10693243 | 10693343 |
| Marker83553 | LG6 | 10693830 | 10693780 | 10693880 |
| Marker83556 | LG6 | 10716358 | 10716308 | 10716408 |
| Marker83558 | LG6 | 10726618 | 10726568 | 10726668 |
| Marker83560 | LG6 | 10737575 | 10737525 | 10737625 |
| Marker83565 | LG6 | 10759006 | 10758956 | 10759056 |
| Marker83566 | LG6 | 10767634 | 10767584 | 10767684 |
| Marker83567 | LG6 | 10771680 | 10771630 | 10771730 |
| Marker83568 | LG6 | 10774218 | 10774168 | 10774268 |
| Marker83602 | LG6 | 10869271 | 10869221 | 10869321 |
| Marker83615 | LG6 | 10890449 | 10890399 | 10890499 |
| Marker83616 | LG6 | 10893082 | 10893032 | 10893132 |
| Marker83678 | LG6 | 10967753 | 10967703 | 10967803 |
| Marker83691 | LG6 | 10990203 | 10990153 | 10990253 |
| Marker83697 | LG6 | 11002618 | 11002568 | 11002668 |
| Marker83712 | LG6 | 11015297 | 11015247 | 11015347 |
| Marker83743 | LG6 | 11037079 | 11037029 | 11037129 |
| Marker83748 | LG6 | 11039358 | 11039308 | 11039408 |
| Marker83755 | LG6 | 11044501 | 11044451 | 11044551 |
| Marker83759 | LG6 | 11052879 | 11052829 | 11052929 |
| Marker83769 | LG6 | 11060928 | 11060878 | 11060978 |
| Marker83772 | LG6 | 11064309 | 11064259 | 11064359 |
| Marker83778 | LG6 | 11078346 | 11078296 | 11078396 |
| Marker83785 | LG6 | 11099919 | 11099869 | 11099969 |
| Marker102671 | LG6 | 11117050 | 11117000 | 11117100 |
| Marker83814 | LG6 | 11155237 | 11155187 | 11155287 |
| Marker83847 | LG6 | 11196167 | 11196117 | 11196217 |
| Marker83858 | LG6 | 11207042 | 11206992 | 11207092 |
| Marker83871 | LG6 | 11216493 | 11216443 | 11216543 |
| Marker83878 | LG6 | 11218935 | 11218885 | 11218985 |
| Marker83887 | LG6 | 11249981 | 11249931 | 11250031 |
| Marker83948 | LG6 | 11321902 | 11321852 | 11321952 |
| Marker83949 | LG6 | 11325072 | 11325022 | 11325122 |
| Marker84006 | LG6 | 11426321 | 11426271 | 11426371 |
| Marker83340 | LG6 | 11464938 | 11464888 | 11464988 |
| Marker83321 | LG6 | 11484199 | 11484149 | 11484249 |
| Marker83302 | LG6 | 11511628 | 11511578 | 11511678 |
| Marker83294 | LG6 | 11517899 | 11517849 | 11517949 |
| Marker83277 | LG6 | 11537764 | 11537714 | 11537814 |
| Marker83258 | LG6 | 11588953 | 11588903 | 11589003 |
| Marker83249 | LG6 | 11589606 | 11589556 | 11589656 |
| Marker83248 | LG6 | 11589839 | 11589789 | 11589889 |
| Marker83237 | LG6 | 11600480 | 11600430 | 11600530 |
| Marker83220 | LG6 | 11608067 | 11608017 | 11608117 |
| Marker83189 | LG6 | 11656588 | 11656538 | 11656638 |
| Marker83109 | LG6 | 11796902 | 11796852 | 11796952 |
| Marker83087 | LG6 | 11823854 | 11823804 | 11823904 |
| Marker83082 | LG6 | 11833312 | 11833262 | 11833362 |
| Marker84028 | LG6 | 11840850 | 11840800 | 11840900 |
| Marker84030 | LG6 | 11842502 | 11842452 | 11842552 |
| Marker84033 | LG6 | 11844346 | 11844296 | 11844396 |
| Marker84044 | LG6 | 11853412 | 11853362 | 11853462 |
| Marker84058 | LG6 | 11855733 | 11855683 | 11855783 |
| Marker84059 | LG6 | 11856448 | 11856398 | 11856498 |
| Marker84064 | LG6 | 11856728 | 11856678 | 11856778 |
| Marker84069 | LG6 | 11857617 | 11857567 | 11857667 |
| Marker84109 | LG6 | 11959152 | 11959102 | 11959202 |
| Marker84242 | LG6 | 12137955 | 12137905 | 12138005 |
| Marker84247 | LG6 | 12143694 | 12143644 | 12143744 |
| Marker84263 | LG6 | 12186255 | 12186205 | 12186305 |
| Marker84268 | LG6 | 12193262 | 12193212 | 12193312 |
| Marker84285 | LG6 | 12216716 | 12216666 | 12216766 |
| Marker84293 | LG6 | 12224440 | 12224390 | 12224490 |
| Marker84336 | LG6 | 12267728 | 12267678 | 12267778 |
| Marker84365 | LG6 | 12276760 | 12276710 | 12276810 |
| Marker84370 | LG6 | 12284252 | 12284202 | 12284302 |
| Marker84371 | LG6 | 12285877 | 12285827 | 12285927 |
| Marker84375 | LG6 | 12297834 | 12297784 | 12297884 |
| Marker84377 | LG6 | 12305563 | 12305513 | 12305613 |
| Marker84378 | LG6 | 12305835 | 12305785 | 12305885 |
| Marker85413 | LG6 | 12491296 | 12491246 | 12491346 |
| Marker85416 | LG6 | 12494747 | 12494697 | 12494797 |
| Marker85437 | LG6 | 12524394 | 12524344 | 12524444 |
| Marker85502 | LG6 | 12569709 | 12569659 | 12569759 |
| Marker85503 | LG6 | 12571755 | 12571705 | 12571805 |
| Marker84876 | LG6 | 12672101 | 12672051 | 12672151 |
| Marker84860 | LG6 | 12718541 | 12718491 | 12718591 |
| Marker84830 | LG6 | 12814072 | 12814022 | 12814122 |
| Marker84922 | LG6 | 12897018 | 12896968 | 12897068 |
| Marker84919 | LG6 | 12899008 | 12898958 | 12899058 |
| Marker85954 | LG6 | 12973803 | 12973753 | 12973853 |
| Marker85970 | LG6 | 12984966 | 12984916 | 12985016 |
| Marker86059 | LG6 | 13041792 | 13041742 | 13041842 |
| Marker86064 | LG6 | 13047666 | 13047616 | 13047716 |
| Marker84758 | LG6 | 13071252 | 13071202 | 13071302 |
| Marker84782 | LG6 | 13089076 | 13089026 | 13089126 |
| Marker84801 | LG6 | 13118495 | 13118445 | 13118545 |
| Marker84806 | LG6 | 13122133 | 13122083 | 13122183 |
| Marker84993 | LG6 | 13136661 | 13136611 | 13136711 |
| Marker85008 | LG6 | 13146367 | 13146317 | 13146417 |
| Marker85037 | LG6 | 13172283 | 13172233 | 13172333 |
| Marker85038 | LG6 | 13172359 | 13172309 | 13172409 |
| Marker85044 | LG6 | 13226163 | 13226113 | 13226213 |
| Marker85048 | LG6 | 13230261 | 13230211 | 13230311 |
| Marker86560 | LG6 | 13472034 | 13471984 | 13472084 |
| Marker86562 | LG6 | 13476492 | 13476442 | 13476542 |
| Marker85915 | LG6 | 13625530 | 13625480 | 13625580 |
| Marker86747 | LG6 | 13646786 | 13646736 | 13646836 |
| Marker85882 | LG6 | 13668201 | 13668151 | 13668251 |
| Marker86289 | LG6 | 13691211 | 13691161 | 13691261 |
| Marker86273 | LG6 | 13706791 | 13706741 | 13706841 |
| Marker86252 | LG6 | 13718409 | 13718359 | 13718459 |
| Marker86247 | LG6 | 13720994 | 13720944 | 13721044 |
| Marker86228 | LG6 | 13744860 | 13744810 | 13744910 |
| Marker86212 | LG6 | 13755417 | 13755367 | 13755467 |
| Marker86132 | LG6 | 13883276 | 13883226 | 13883326 |
| Marker86131 | LG6 | 13886312 | 13886262 | 13886362 |
| Marker86130 | LG6 | 13890168 | 13890118 | 13890218 |
| Marker86127 | LG6 | 13890435 | 13890385 | 13890485 |
| Marker86110 | LG6 | 13906543 | 13906493 | 13906593 |
| Marker86109 | LG6 | 13906888 | 13906838 | 13906938 |
| Marker85874 | LG6 | 13974099 | 13974049 | 13974149 |
| Marker85868 | LG6 | 13977626 | 13977576 | 13977676 |
| Marker84732 | LG6 | 14071008 | 14070958 | 14071058 |
| Marker86755 | LG6 | 14178325 | 14178275 | 14178375 |
| Marker86760 | LG6 | 14190239 | 14190189 | 14190289 |
| Marker86761 | LG6 | 14191435 | 14191385 | 14191485 |
| Marker86779 | LG6 | 14208507 | 14208457 | 14208557 |
| Marker85824 | LG6 | 14269955 | 14269905 | 14270005 |
| Marker85933 | LG6 | 14326051 | 14326001 | 14326101 |
| Marker85937 | LG6 | 14333699 | 14333649 | 14333749 |
| Marker14880 | LG6 | 14447442 | 14447392 | 14447492 |
| Marker86753 | LG6 | 14572400 | 14572350 | 14572450 |
| Marker117134 | LG6 | 14737733 | 14737683 | 14737783 |
| Marker86295 | LG6 | 14790084 | 14790034 | 14790134 |
| Marker86299 | LG6 | 14794997 | 14794947 | 14795047 |
| Marker86300 | LG6 | 14795367 | 14795317 | 14795417 |
| Marker86302 | LG6 | 14805445 | 14805395 | 14805495 |
| Marker86303 | LG6 | 14805594 | 14805544 | 14805644 |
| Marker86510 | LG6 | 14973123 | 14973073 | 14973173 |
| Marker86508 | LG6 | 14976529 | 14976479 | 14976579 |
| Marker86497 | LG6 | 14989633 | 14989583 | 14989683 |
| Marker86484 | LG6 | 15000405 | 15000355 | 15000455 |
| Marker87001 | LG6 | 15032292 | 15032242 | 15032342 |
| Marker86808 | LG6 | 15062911 | 15062861 | 15062961 |
| Marker86812 | LG6 | 15069453 | 15069403 | 15069503 |
| Marker86840 | LG6 | 15098621 | 15098571 | 15098671 |
| Marker86841 | LG6 | 15098729 | 15098679 | 15098779 |
| Marker117632 | LG6 | 15195265 | 15195215 | 15195315 |
| Marker119175 | LG6 | 15266220 | 15266170 | 15266270 |
| Marker116837 | LG6 | 15328213 | 15328163 | 15328263 |
| Marker116838 | LG6 | 15328284 | 15328234 | 15328334 |
| Marker116862 | LG6 | 15347613 | 15347563 | 15347663 |
| Marker86700 | LG6 | 15468485 | 15468435 | 15468535 |
| Marker86742 | LG6 | 15526929 | 15526879 | 15526979 |
| Marker86428 | LG6 | 15562447 | 15562397 | 15562497 |
| Marker86434 | LG6 | 15570486 | 15570436 | 15570536 |
| Marker87156 | LG6 | 15671797 | 15671747 | 15671847 |
| Marker87129 | LG6 | 15674835 | 15674785 | 15674885 |
| Marker87121 | LG6 | 15681197 | 15681147 | 15681247 |
| Marker87087 | LG6 | 15748425 | 15748375 | 15748475 |
| Marker87062 | LG6 | 15781774 | 15781724 | 15781824 |
| Marker87061 | LG6 | 15781950 | 15781900 | 15782000 |
| Marker118301 | LG6 | 15808817 | 15808767 | 15808867 |
| Marker86967 | LG6 | 15955986 | 15955936 | 15956036 |
| Marker86972 | LG6 | 16042268 | 16042218 | 16042318 |
| Marker86976 | LG6 | 16050466 | 16050416 | 16050516 |
| Marker86977 | LG6 | 16053622 | 16053572 | 16053672 |
| Marker86982 | LG6 | 16063642 | 16063592 | 16063692 |
| Marker86990 | LG6 | 16070421 | 16070371 | 16070471 |
| Marker87342 | LG6 | 16290774 | 16290724 | 16290824 |
| Marker87339 | LG6 | 16291900 | 16291850 | 16291950 |
| Marker87329 | LG6 | 16306305 | 16306255 | 16306355 |
| Marker87326 | LG6 | 16306555 | 16306505 | 16306605 |
| Marker87263 | LG6 | 16356285 | 16356235 | 16356335 |
| Marker88975 | LG6 | 16463478 | 16463428 | 16463528 |
| Marker88978 | LG6 | 16470295 | 16470245 | 16470345 |
| Marker88996 | LG6 | 16498009 | 16497959 | 16498059 |
| Marker89007 | LG6 | 16506635 | 16506585 | 16506685 |
| Marker89060 | LG6 | 16523958 | 16523908 | 16524008 |
| Marker89080 | LG6 | 16557892 | 16557842 | 16557942 |
| Marker89096 | LG6 | 16570680 | 16570630 | 16570730 |
| Marker63443 | LG6 | 16675435 | 16675385 | 16675485 |
| Marker89118 | LG6 | 16678850 | 16678800 | 16678900 |
| Marker88429 | LG6 | 16791519 | 16791469 | 16791569 |
| Marker88387 | LG6 | 16829705 | 16829655 | 16829755 |
| Marker88331 | LG6 | 16894160 | 16894110 | 16894210 |
| Marker88316 | LG6 | 16927687 | 16927637 | 16927737 |
| Marker88312 | LG6 | 16932836 | 16932786 | 16932886 |
| Marker88265 | LG6 | 16979973 | 16979923 | 16980023 |
| Marker88264 | LG6 | 16980197 | 16980147 | 16980247 |
| Marker109573 | LG6 | 16993819 | 16993769 | 16993869 |
| Marker109577 | LG6 | 17000225 | 17000175 | 17000275 |
| Marker109597 | LG6 | 17009129 | 17009079 | 17009179 |
| Marker109610 | LG6 | 17021456 | 17021406 | 17021506 |
| Marker109614 | LG6 | 17024131 | 17024081 | 17024181 |
| Marker109615 | LG6 | 17027413 | 17027363 | 17027463 |
| Marker109617 | LG6 | 17027574 | 17027524 | 17027624 |
| Marker109625 | LG6 | 17029262 | 17029212 | 17029312 |
| Marker109646 | LG6 | 17050244 | 17050194 | 17050294 |
| Marker109738 | LG6 | 17138823 | 17138773 | 17138873 |
| Marker109744 | LG6 | 17145000 | 17144950 | 17145050 |
| Marker109747 | LG6 | 17167253 | 17167203 | 17167303 |
| Marker109759 | LG6 | 17183791 | 17183741 | 17183841 |
| Marker88195 | LG6 | 17332573 | 17332523 | 17332623 |
| Marker88157 | LG6 | 17398059 | 17398009 | 17398109 |
| Marker88126 | LG6 | 17436319 | 17436269 | 17436369 |
| Marker88070 | LG6 | 17465095 | 17465045 | 17465145 |
| Marker88031 | LG6 | 17686121 | 17686071 | 17686171 |
| Marker88022 | LG6 | 17696592 | 17696542 | 17696642 |
| Marker88015 | LG6 | 17706185 | 17706135 | 17706235 |
| Marker87994 | LG6 | 17722460 | 17722410 | 17722510 |
| Marker89495 | LG6 | 17783129 | 17783079 | 17783179 |
| Marker89483 | LG6 | 17821832 | 17821782 | 17821882 |
| Marker89466 | LG6 | 17828283 | 17828233 | 17828333 |
| Marker89465 | LG6 | 17830202 | 17830152 | 17830252 |
| Marker89457 | LG6 | 17836048 | 17835998 | 17836098 |
| Marker89397 | LG6 | 17880797 | 17880747 | 17880847 |
| Marker119614 | LG6 | 17892899 | 17892849 | 17892949 |
| Marker119605 | LG6 | 17896093 | 17896043 | 17896143 |
| Marker87963 | LG6 | 18006472 | 18006422 | 18006522 |
| Marker87941 | LG6 | 18044708 | 18044658 | 18044758 |
| Marker87932 | LG6 | 18053885 | 18053835 | 18053935 |
| Marker87901 | LG6 | 18098718 | 18098668 | 18098768 |
| Marker87860 | LG6 | 18137029 | 18136979 | 18137079 |
| Marker87799 | LG6 | 18151974 | 18151924 | 18152024 |
| Marker87783 | LG6 | 18170025 | 18169975 | 18170075 |
| Marker59186 | LG6 | 18176948 | 18176898 | 18176998 |
| Marker87732 | LG6 | 18226839 | 18226789 | 18226889 |
| Marker87726 | LG6 | 18231108 | 18231058 | 18231158 |
| Marker87616 | LG6 | 18316378 | 18316328 | 18316428 |
| Marker87614 | LG6 | 18318329 | 18318279 | 18318379 |
| Marker87601 | LG6 | 18350630 | 18350580 | 18350680 |
| Marker87599 | LG6 | 18352096 | 18352046 | 18352146 |
| Marker87584 | LG6 | 18389986 | 18389936 | 18390036 |
| Marker87579 | LG6 | 18417787 | 18417737 | 18417837 |
| Marker87535 | LG6 | 18500063 | 18500013 | 18500113 |
| Marker87483 | LG6 | 18551232 | 18551182 | 18551282 |
| Marker87449 | LG6 | 18563856 | 18563806 | 18563906 |
| Marker88479 | LG6 | 18629614 | 18629564 | 18629664 |
| Marker88480 | LG6 | 18629867 | 18629817 | 18629917 |
| Marker88493 | LG6 | 18635371 | 18635321 | 18635421 |
| Marker88520 | LG6 | 18669029 | 18668979 | 18669079 |
| Marker88525 | LG6 | 18683701 | 18683651 | 18683751 |
| Marker88605 | LG6 | 18801096 | 18801046 | 18801146 |
| Marker88660 | LG6 | 18860557 | 18860507 | 18860607 |
| Marker88715 | LG6 | 18971560 | 18971510 | 18971610 |
| Marker88722 | LG6 | 18975572 | 18975522 | 18975622 |
| Marker88730 | LG6 | 18978406 | 18978356 | 18978456 |
| Marker88733 | LG6 | 18982339 | 18982289 | 18982389 |
| Marker88734 | LG6 | 18985415 | 18985365 | 18985465 |
| Marker88783 | LG6 | 19077623 | 19077573 | 19077673 |
| Marker88798 | LG6 | 19108475 | 19108425 | 19108525 |
| Marker88813 | LG6 | 19110497 | 19110447 | 19110547 |
| Marker88828 | LG6 | 19118555 | 19118505 | 19118605 |
| Marker89178 | LG6 | 19217966 | 19217916 | 19218016 |
| Marker89184 | LG6 | 19218213 | 19218163 | 19218263 |
| Marker89212 | LG6 | 19237155 | 19237105 | 19237205 |
| Marker39848 | LG6 | 19275725 | 19275675 | 19275775 |
| Marker89353 | LG6 | 19339939 | 19339889 | 19339989 |
| Marker113893 | LG6 | 19429927 | 19429877 | 19429977 |
| Marker113884 | LG6 | 19436870 | 19436820 | 19436920 |
| Marker113882 | LG6 | 19437157 | 19437107 | 19437207 |
| Marker113852 | LG6 | 19485653 | 19485603 | 19485703 |
| Marker113846 | LG6 | 19517431 | 19517381 | 19517481 |
| Marker113837 | LG6 | 19536061 | 19536011 | 19536111 |
| Marker70719 | LG6 | 19625247 | 19625197 | 19625297 |
| Marker70721 | LG6 | 19625431 | 19625381 | 19625481 |
| Marker89246 | LG6 | 19782748 | 19782698 | 19782798 |
| Marker89250 | LG6 | 19787922 | 19787872 | 19787972 |
| Marker89272 | LG6 | 19808727 | 19808677 | 19808777 |
| Marker89295 | LG6 | 19858192 | 19858142 | 19858242 |
| Marker89299 | LG6 | 19860717 | 19860667 | 19860767 |
| Marker89306 | LG6 | 19870622 | 19870572 | 19870672 |
| Marker89310 | LG6 | 19906520 | 19906470 | 19906570 |
| Marker89314 | LG6 | 19911288 | 19911238 | 19911338 |
| Marker88895 | LG6 | 19962323 | 19962273 | 19962373 |
| Marker88929 | LG6 | 19993272 | 19993222 | 19993322 |
| Marker88942 | LG6 | 20029657 | 20029607 | 20029707 |
| Marker70680 | LG6 | 20124887 | 20124837 | 20124937 |
| Marker70708 | LG6 | 20170540 | 20170490 | 20170590 |
| Marker70710 | LG6 | 20170789 | 20170739 | 20170839 |
| Marker89990 | LG7 | 1851 | 1801 | 1901 |
| Marker89996 | LG7 | 27567 | 27517 | 27617 |
| Marker90026 | LG7 | 67955 | 67905 | 68005 |
| Marker90030 | LG7 | 78555 | 78505 | 78605 |
| Marker90036 | LG7 | 87645 | 87595 | 87695 |
| Marker90048 | LG7 | 104434 | 104384 | 104484 |
| Marker33129 | LG7 | 121711 | 121661 | 121761 |
| Marker33144 | LG7 | 124987 | 124937 | 125037 |
| Marker117235 | LG7 | 221160 | 221110 | 221210 |
| Marker117233 | LG7 | 221695 | 221645 | 221745 |
| Marker117223 | LG7 | 224657 | 224607 | 224707 |
| Marker89985 | LG7 | 257600 | 257550 | 257650 |
| Marker89973 | LG7 | 271500 | 271450 | 271550 |
| Marker89971 | LG7 | 278548 | 278498 | 278598 |
| Marker89948 | LG7 | 316684 | 316634 | 316734 |
| Marker89940 | LG7 | 338706 | 338656 | 338756 |
| Marker89915 | LG7 | 361980 | 361930 | 362030 |
| Marker89886 | LG7 | 377160 | 377110 | 377210 |
| Marker89872 | LG7 | 415639 | 415589 | 415689 |
| Marker89848 | LG7 | 436194 | 436144 | 436244 |
| Marker89837 | LG7 | 448210 | 448160 | 448260 |
| Marker89826 | LG7 | 459835 | 459785 | 459885 |
| Marker89823 | LG7 | 464155 | 464105 | 464205 |
| Marker89822 | LG7 | 464187 | 464137 | 464237 |
| Marker89820 | LG7 | 471861 | 471811 | 471911 |
| Marker89815 | LG7 | 472599 | 472549 | 472649 |
| Marker89792 | LG7 | 499990 | 499940 | 500040 |
| Marker89783 | LG7 | 504437 | 504387 | 504487 |
| Marker89782 | LG7 | 504657 | 504607 | 504707 |
| Marker89765 | LG7 | 526603 | 526553 | 526653 |
| Marker89750 | LG7 | 536314 | 536264 | 536364 |
| Marker89722 | LG7 | 610098 | 610048 | 610148 |
| Marker89718 | LG7 | 612267 | 612217 | 612317 |
| Marker89710 | LG7 | 635263 | 635213 | 635313 |
| Marker89708 | LG7 | 635294 | 635244 | 635344 |
| Marker89629 | LG7 | 775267 | 775217 | 775317 |
| Marker89625 | LG7 | 790140 | 790090 | 790190 |
| Marker89624 | LG7 | 790733 | 790683 | 790783 |
| Marker89621 | LG7 | 792183 | 792133 | 792233 |
| Marker89617 | LG7 | 797992 | 797942 | 798042 |
| Marker89612 | LG7 | 814017 | 813967 | 814067 |
| Marker89607 | LG7 | 815682 | 815632 | 815732 |
| Marker89574 | LG7 | 885238 | 885188 | 885288 |
| Marker89568 | LG7 | 887563 | 887513 | 887613 |
| Marker89567 | LG7 | 891586 | 891536 | 891636 |
| Marker89558 | LG7 | 914604 | 914554 | 914654 |
| Marker89556 | LG7 | 916670 | 916620 | 916720 |
| Marker89551 | LG7 | 930818 | 930768 | 930868 |
| Marker73796 | LG7 | 1066521 | 1066471 | 1066571 |
| Marker73798 | LG7 | 1071028 | 1070978 | 1071078 |
| Marker73811 | LG7 | 1089914 | 1089864 | 1089964 |
| Marker90196 | LG7 | 1253612 | 1253562 | 1253662 |
| Marker90155 | LG7 | 1290978 | 1290928 | 1291028 |
| Marker90068 | LG7 | 1385198 | 1385148 | 1385248 |
| Marker117172 | LG7 | 1398636 | 1398586 | 1398686 |
| Marker117178 | LG7 | 1399876 | 1399826 | 1399926 |
| Marker117179 | LG7 | 1403062 | 1403012 | 1403112 |
| Marker90295 | LG7 | 1480592 | 1480542 | 1480642 |
| Marker90314 | LG7 | 1506992 | 1506942 | 1507042 |
| Marker90342 | LG7 | 1543377 | 1543327 | 1543427 |
| Marker90352 | LG7 | 1563195 | 1563145 | 1563245 |
| Marker90354 | LG7 | 1563445 | 1563395 | 1563495 |
| Marker90400 | LG7 | 1652303 | 1652253 | 1652353 |
| Marker90414 | LG7 | 1676401 | 1676351 | 1676451 |
| Marker90476 | LG7 | 1773885 | 1773835 | 1773935 |
| Marker90480 | LG7 | 1775056 | 1775006 | 1775106 |
| Marker90494 | LG7 | 1789892 | 1789842 | 1789942 |
| Marker90495 | LG7 | 1808862 | 1808812 | 1808912 |
| Marker90500 | LG7 | 1824595 | 1824545 | 1824645 |
| Marker90511 | LG7 | 1830084 | 1830034 | 1830134 |
| Marker90515 | LG7 | 1831876 | 1831826 | 1831926 |
| Marker90519 | LG7 | 1834184 | 1834134 | 1834234 |
| Marker90525 | LG7 | 1858132 | 1858082 | 1858182 |
| Marker90581 | LG7 | 1972712 | 1972662 | 1972762 |
| Marker90604 | LG7 | 1997689 | 1997639 | 1997739 |
| Marker90627 | LG7 | 2027883 | 2027833 | 2027933 |
| Marker90644 | LG7 | 2053716 | 2053666 | 2053766 |
| Marker90653 | LG7 | 2065850 | 2065800 | 2065900 |
| Marker90655 | LG7 | 2066056 | 2066006 | 2066106 |
| Marker90658 | LG7 | 2070863 | 2070813 | 2070913 |
| Marker90783 | LG7 | 2163144 | 2163094 | 2163194 |
| Marker90785 | LG7 | 2164172 | 2164122 | 2164222 |
| Marker90786 | LG7 | 2164326 | 2164276 | 2164376 |
| Marker90807 | LG7 | 2180572 | 2180522 | 2180622 |
| Marker90864 | LG7 | 2222656 | 2222606 | 2222706 |
| Marker90872 | LG7 | 2232459 | 2232409 | 2232509 |
| Marker90873 | LG7 | 2234614 | 2234564 | 2234664 |
| Marker90876 | LG7 | 2236739 | 2236689 | 2236789 |
| Marker90877 | LG7 | 2236961 | 2236911 | 2237011 |
| Marker90883 | LG7 | 2244451 | 2244401 | 2244501 |
| Marker90899 | LG7 | 2269888 | 2269838 | 2269938 |
| Marker90900 | LG7 | 2271992 | 2271942 | 2272042 |
| Marker90925 | LG7 | 2307484 | 2307434 | 2307534 |
| Marker90931 | LG7 | 2317106 | 2317056 | 2317156 |
| Marker90935 | LG7 | 2329829 | 2329779 | 2329879 |
| Marker90954 | LG7 | 2352237 | 2352187 | 2352287 |
| Marker90969 | LG7 | 2369029 | 2368979 | 2369079 |
| Marker90996 | LG7 | 2400975 | 2400925 | 2401025 |
| Marker90997 | LG7 | 2410212 | 2410162 | 2410262 |
| Marker90998 | LG7 | 2413492 | 2413442 | 2413542 |
| Marker91026 | LG7 | 2499152 | 2499102 | 2499202 |
| Marker91027 | LG7 | 2502829 | 2502779 | 2502879 |
| Marker91059 | LG7 | 2563810 | 2563760 | 2563860 |
| Marker91061 | LG7 | 2563989 | 2563939 | 2564039 |
| Marker91101 | LG7 | 2614342 | 2614292 | 2614392 |
| Marker91109 | LG7 | 2632181 | 2632131 | 2632231 |
| Marker91167 | LG7 | 2751198 | 2751148 | 2751248 |
| Marker91202 | LG7 | 2792185 | 2792135 | 2792235 |
| Marker91253 | LG7 | 2898143 | 2898093 | 2898193 |
| Marker91270 | LG7 | 2919124 | 2919074 | 2919174 |
| Marker91310 | LG7 | 2940428 | 2940378 | 2940478 |
| Marker91313 | LG7 | 2940661 | 2940611 | 2940711 |
| Marker91330 | LG7 | 2976220 | 2976170 | 2976270 |
| Marker91336 | LG7 | 3090011 | 3089961 | 3090061 |
| Marker91337 | LG7 | 3090787 | 3090737 | 3090837 |
| Marker91367 | LG7 | 3147522 | 3147472 | 3147572 |
| Marker91372 | LG7 | 3153903 | 3153853 | 3153953 |
| Marker91382 | LG7 | 3158172 | 3158122 | 3158222 |
| Marker91394 | LG7 | 3188343 | 3188293 | 3188393 |
| Marker91411 | LG7 | 3222376 | 3222326 | 3222426 |
| Marker91422 | LG7 | 3233039 | 3232989 | 3233089 |
| Marker91439 | LG7 | 3253282 | 3253232 | 3253332 |
| Marker91449 | LG7 | 3278735 | 3278685 | 3278785 |
| Marker91463 | LG7 | 3336806 | 3336756 | 3336856 |
| Marker91470 | LG7 | 3343339 | 3343289 | 3343389 |
| Marker91504 | LG7 | 3364244 | 3364194 | 3364294 |
| Marker91518 | LG7 | 3376804 | 3376754 | 3376854 |
| Marker91562 | LG7 | 3435455 | 3435405 | 3435505 |
| Marker91570 | LG7 | 3435732 | 3435682 | 3435782 |
| Marker91602 | LG7 | 3469750 | 3469700 | 3469800 |
| Marker91603 | LG7 | 3469794 | 3469744 | 3469844 |
| Marker91609 | LG7 | 3473203 | 3473153 | 3473253 |
| Marker91612 | LG7 | 3489619 | 3489569 | 3489669 |
| Marker91741 | LG7 | 3645826 | 3645776 | 3645876 |
| Marker91746 | LG7 | 3645948 | 3645898 | 3645998 |
| Marker91812 | LG7 | 3764046 | 3763996 | 3764096 |
| Marker91850 | LG7 | 3857532 | 3857482 | 3857582 |
| Marker115367 | LG7 | 3860728 | 3860678 | 3860778 |
| Marker91862 | LG7 | 3890741 | 3890691 | 3890791 |
| Marker91864 | LG7 | 3892286 | 3892236 | 3892336 |
| Marker91885 | LG7 | 3908221 | 3908171 | 3908271 |
| Marker91899 | LG7 | 3942090 | 3942040 | 3942140 |
| Marker91907 | LG7 | 3946810 | 3946760 | 3946860 |
| Marker91909 | LG7 | 3951918 | 3951868 | 3951968 |
| Marker91911 | LG7 | 3954167 | 3954117 | 3954217 |
| Marker91927 | LG7 | 3985779 | 3985729 | 3985829 |
| Marker91929 | LG7 | 3986930 | 3986880 | 3986980 |
| Marker91933 | LG7 | 3987990 | 3987940 | 3988040 |
| Marker91941 | LG7 | 3992372 | 3992322 | 3992422 |
| Marker91945 | LG7 | 4019210 | 4019160 | 4019260 |
| Marker91979 | LG7 | 4032099 | 4032049 | 4032149 |
| Marker92034 | LG7 | 4166805 | 4166755 | 4166855 |
| Marker92041 | LG7 | 4174179 | 4174129 | 4174229 |
| Marker92071 | LG7 | 4188854 | 4188804 | 4188904 |
| Marker92073 | LG7 | 4190008 | 4189958 | 4190058 |
| Marker92096 | LG7 | 4203540 | 4203490 | 4203590 |
| Marker92117 | LG7 | 4213319 | 4213269 | 4213369 |
| Marker92138 | LG7 | 4237317 | 4237267 | 4237367 |
| Marker92165 | LG7 | 4262881 | 4262831 | 4262931 |
| Marker93252 | LG7 | 4359922 | 4359872 | 4359972 |
| Marker93274 | LG7 | 4408341 | 4408291 | 4408391 |
| Marker93302 | LG7 | 4431550 | 4431500 | 4431600 |
| Marker93316 | LG7 | 4454343 | 4454293 | 4454393 |
| Marker93318 | LG7 | 4458821 | 4458771 | 4458871 |
| Marker93339 | LG7 | 4476322 | 4476272 | 4476372 |
| Marker93342 | LG7 | 4478960 | 4478910 | 4479010 |
| Marker93344 | LG7 | 4484821 | 4484771 | 4484871 |
| Marker93345 | LG7 | 4496323 | 4496273 | 4496373 |
| Marker93364 | LG7 | 4526700 | 4526650 | 4526750 |
| Marker93376 | LG7 | 4540078 | 4540028 | 4540128 |
| Marker93398 | LG7 | 4594611 | 4594561 | 4594661 |
| Marker93410 | LG7 | 4635742 | 4635692 | 4635792 |
| Marker93415 | LG7 | 4636702 | 4636652 | 4636752 |
| Marker93416 | LG7 | 4636735 | 4636685 | 4636785 |
| Marker93436 | LG7 | 4670987 | 4670937 | 4671037 |
| Marker93437 | LG7 | 4675812 | 4675762 | 4675862 |
| Marker93443 | LG7 | 4697138 | 4697088 | 4697188 |
| Marker93449 | LG7 | 4738931 | 4738881 | 4738981 |
| Marker93497 | LG7 | 4792114 | 4792064 | 4792164 |
| Marker93489 | LG7 | 4824388 | 4824338 | 4824438 |
| Marker93510 | LG7 | 4887970 | 4887920 | 4888020 |
| Marker93513 | LG7 | 4889396 | 4889346 | 4889446 |
| Marker93520 | LG7 | 4898422 | 4898372 | 4898472 |
| Marker93539 | LG7 | 4943091 | 4943041 | 4943141 |
| Marker93551 | LG7 | 4970721 | 4970671 | 4970771 |
| Marker93587 | LG7 | 5006121 | 5006071 | 5006171 |
| Marker93623 | LG7 | 5053031 | 5052981 | 5053081 |
| Marker93632 | LG7 | 5053830 | 5053780 | 5053880 |
| Marker93038 | LG7 | 5676205 | 5676155 | 5676255 |
| Marker93044 | LG7 | 5711759 | 5711709 | 5711809 |
| Marker93060 | LG7 | 5716925 | 5716875 | 5716975 |
| Marker93066 | LG7 | 5781865 | 5781815 | 5781915 |
| Marker93086 | LG7 | 5839203 | 5839153 | 5839253 |
| Marker93123 | LG7 | 5863456 | 5863406 | 5863506 |
| Marker93124 | LG7 | 5870856 | 5870806 | 5870906 |
| Marker93150 | LG7 | 5910802 | 5910752 | 5910852 |
| Marker93159 | LG7 | 5945038 | 5944988 | 5945088 |
| Marker93209 | LG7 | 6006558 | 6006508 | 6006608 |
| Marker93013 | LG7 | 6116368 | 6116318 | 6116418 |
| Marker93012 | LG7 | 6116587 | 6116537 | 6116637 |
| Marker92910 | LG7 | 6245402 | 6245352 | 6245452 |
| Marker92834 | LG7 | 6327635 | 6327585 | 6327685 |
| Marker92814 | LG7 | 6330782 | 6330732 | 6330832 |
| Marker92813 | LG7 | 6336932 | 6336882 | 6336982 |
| Marker92810 | LG7 | 6340416 | 6340366 | 6340466 |
| Marker92797 | LG7 | 6371284 | 6371234 | 6371334 |
| Marker92783 | LG7 | 6385516 | 6385466 | 6385566 |
| Marker92725 | LG7 | 6439552 | 6439502 | 6439602 |
| Marker92723 | LG7 | 6441239 | 6441189 | 6441289 |
| Marker92713 | LG7 | 6451326 | 6451276 | 6451376 |
| Marker92609 | LG7 | 6528294 | 6528244 | 6528344 |
| Marker92549 | LG7 | 6665947 | 6665897 | 6665997 |
| Marker92488 | LG7 | 6736166 | 6736116 | 6736216 |
| Marker92483 | LG7 | 6744218 | 6744168 | 6744268 |
| Marker92477 | LG7 | 6766592 | 6766542 | 6766642 |
| Marker92465 | LG7 | 6776455 | 6776405 | 6776505 |
| Marker92458 | LG7 | 6784340 | 6784290 | 6784390 |
| Marker92452 | LG7 | 6784597 | 6784547 | 6784647 |
| Marker92444 | LG7 | 6800762 | 6800712 | 6800812 |
| Marker92443 | LG7 | 6800789 | 6800739 | 6800839 |
| Marker92439 | LG7 | 6801867 | 6801817 | 6801917 |
| Marker92411 | LG7 | 6823090 | 6823040 | 6823140 |
| Marker92396 | LG7 | 6826524 | 6826474 | 6826574 |
| Marker92388 | LG7 | 6830720 | 6830670 | 6830770 |
| Marker92344 | LG7 | 6869115 | 6869065 | 6869165 |
| Marker92341 | LG7 | 6879140 | 6879090 | 6879190 |
| Marker92326 | LG7 | 6902332 | 6902282 | 6902382 |
| Marker92305 | LG7 | 6926697 | 6926647 | 6926747 |
| Marker92289 | LG7 | 6944544 | 6944494 | 6944594 |
| Marker92288 | LG7 | 6947059 | 6947009 | 6947109 |
| Marker92275 | LG7 | 6968769 | 6968719 | 6968819 |
| Marker92261 | LG7 | 6977726 | 6977676 | 6977776 |
| Marker92971 | LG7 | 6999573 | 6999523 | 6999623 |
| Marker92950 | LG7 | 7011291 | 7011241 | 7011341 |
| Marker92948 | LG7 | 7011741 | 7011691 | 7011791 |
| Marker92945 | LG7 | 7019198 | 7019148 | 7019248 |
| Marker92206 | LG7 | 7052298 | 7052248 | 7052348 |
| Marker58283 | LG7 | 7130718 | 7130668 | 7130768 |
| Marker58276 | LG7 | 7152510 | 7152460 | 7152560 |
| Marker58252 | LG7 | 7175419 | 7175369 | 7175469 |
| Marker58201 | LG7 | 7214456 | 7214406 | 7214506 |
| Marker58189 | LG7 | 7223099 | 7223049 | 7223149 |
| Marker58138 | LG7 | 7266929 | 7266879 | 7266979 |
| Marker58129 | LG7 | 7282564 | 7282514 | 7282614 |
| Marker58102 | LG7 | 7353304 | 7353254 | 7353354 |
| Marker58093 | LG7 | 7382842 | 7382792 | 7382892 |
| Marker58062 | LG7 | 7440014 | 7439964 | 7440064 |
| Marker58058 | LG7 | 7459731 | 7459681 | 7459781 |
| Marker58048 | LG7 | 7468186 | 7468136 | 7468236 |
| Marker58018 | LG7 | 7497581 | 7497531 | 7497631 |
| Marker57966 | LG7 | 7575747 | 7575697 | 7575797 |
| Marker57932 | LG7 | 7630504 | 7630454 | 7630554 |
| Marker57911 | LG7 | 7670849 | 7670799 | 7670899 |
| Marker57910 | LG7 | 7672985 | 7672935 | 7673035 |
| Marker113191 | LG7 | 7688034 | 7687984 | 7688084 |
| Marker113151 | LG7 | 7724522 | 7724472 | 7724572 |
| Marker113135 | LG7 | 7740369 | 7740319 | 7740419 |
| Marker113133 | LG7 | 7740599 | 7740549 | 7740649 |
| Marker113117 | LG7 | 7754338 | 7754288 | 7754388 |
| Marker113111 | LG7 | 7772956 | 7772906 | 7773006 |
| Marker115210 | LG7 | 7846243 | 7846193 | 7846293 |
| Marker115191 | LG7 | 7854015 | 7853965 | 7854065 |
| Marker115189 | LG7 | 7864785 | 7864735 | 7864835 |
| Marker93860 | LG7 | 7937328 | 7937278 | 7937378 |
| Marker93806 | LG7 | 8023632 | 8023582 | 8023682 |
| Marker93762 | LG7 | 8078736 | 8078686 | 8078786 |
| Marker93758 | LG7 | 8085780 | 8085730 | 8085830 |
| Marker93800 | LG7 | 8176081 | 8176031 | 8176131 |
| Marker93700 | LG7 | 8226402 | 8226352 | 8226452 |
| Marker93668 | LG7 | 8279069 | 8279019 | 8279119 |
| Marker93663 | LG7 | 8279358 | 8279308 | 8279408 |
| Marker93662 | LG7 | 8284658 | 8284608 | 8284708 |
| Marker93657 | LG7 | 8289562 | 8289512 | 8289612 |
| Marker94130 | LG7 | 8461299 | 8461249 | 8461349 |
| Marker94132 | LG7 | 8461501 | 8461451 | 8461551 |
| Marker94148 | LG7 | 8475667 | 8475617 | 8475717 |
| Marker94219 | LG7 | 8575754 | 8575704 | 8575804 |
| Marker94235 | LG7 | 8840680 | 8840630 | 8840730 |
| Marker94409 | LG7 | 8919030 | 8918980 | 8919080 |
| Marker94398 | LG7 | 8932194 | 8932144 | 8932244 |
| Marker94394 | LG7 | 8936940 | 8936890 | 8936990 |
| Marker94375 | LG7 | 8959936 | 8959886 | 8959986 |
| Marker94368 | LG7 | 8964347 | 8964297 | 8964397 |
| Marker94356 | LG7 | 8983021 | 8982971 | 8983071 |
| Marker94770 | LG7 | 9084076 | 9084026 | 9084126 |
| Marker94768 | LG7 | 9087693 | 9087643 | 9087743 |
| Marker94738 | LG7 | 9128926 | 9128876 | 9128976 |
| Marker94681 | LG7 | 9247906 | 9247856 | 9247956 |
| Marker94680 | LG7 | 9251966 | 9251916 | 9252016 |
| Marker94679 | LG7 | 9252008 | 9251958 | 9252058 |
| Marker94641 | LG7 | 9291986 | 9291936 | 9292036 |
| Marker94640 | LG7 | 9308351 | 9308301 | 9308401 |
| Marker94617 | LG7 | 9330950 | 9330900 | 9331000 |
| Marker94592 | LG7 | 9392671 | 9392621 | 9392721 |
| Marker94585 | LG7 | 9416716 | 9416666 | 9416766 |
| Marker94536 | LG7 | 9465025 | 9464975 | 9465075 |
| Marker94482 | LG7 | 9530569 | 9530519 | 9530619 |
| Marker94448 | LG7 | 9541567 | 9541517 | 9541617 |
| Marker94794 | LG7 | 9586877 | 9586827 | 9586927 |
| Marker94797 | LG7 | 9595111 | 9595061 | 9595161 |
| Marker94800 | LG7 | 9595349 | 9595299 | 9595399 |
| Marker94821 | LG7 | 9625668 | 9625618 | 9625718 |
| Marker94824 | LG7 | 9625869 | 9625819 | 9625919 |
| Marker94843 | LG7 | 9646985 | 9646935 | 9647035 |
| Marker94849 | LG7 | 9653415 | 9653365 | 9653465 |
| Marker94893 | LG7 | 9680014 | 9679964 | 9680064 |
| Marker94902 | LG7 | 9684734 | 9684684 | 9684784 |
| Marker94914 | LG7 | 9702876 | 9702826 | 9702926 |
| Marker94926 | LG7 | 9714054 | 9714004 | 9714104 |
| Marker94945 | LG7 | 9746301 | 9746251 | 9746351 |
| Marker94950 | LG7 | 9761748 | 9761698 | 9761798 |
| Marker94960 | LG7 | 9774083 | 9774033 | 9774133 |
| Marker94972 | LG7 | 9798116 | 9798066 | 9798166 |
| Marker95019 | LG7 | 9833996 | 9833946 | 9834046 |
| Marker95048 | LG7 | 9893097 | 9893047 | 9893147 |
| Marker95056 | LG7 | 9915355 | 9915305 | 9915405 |
| Marker95170 | LG7 | 10207299 | 10207249 | 10207349 |
| Marker95159 | LG7 | 10234427 | 10234377 | 10234477 |
| Marker95154 | LG7 | 10234670 | 10234620 | 10234720 |
| Marker95123 | LG7 | 10265668 | 10265618 | 10265718 |
| Marker95103 | LG7 | 10335596 | 10335546 | 10335646 |
| Marker95097 | LG7 | 10342740 | 10342690 | 10342790 |
| Marker95602 | LG7 | 10355253 | 10355203 | 10355303 |
| Marker95586 | LG7 | 10362535 | 10362485 | 10362585 |
| Marker95574 | LG7 | 10412609 | 10412559 | 10412659 |
| Marker95572 | LG7 | 10416627 | 10416577 | 10416677 |
| Marker95543 | LG7 | 10447478 | 10447428 | 10447528 |
| Marker95526 | LG7 | 10491645 | 10491595 | 10491695 |
| Marker95517 | LG7 | 10522474 | 10522424 | 10522524 |
| Marker95514 | LG7 | 10530768 | 10530718 | 10530818 |
| Marker95505 | LG7 | 10553772 | 10553722 | 10553822 |
| Marker95474 | LG7 | 10589443 | 10589393 | 10589493 |
| Marker95433 | LG7 | 10637246 | 10637196 | 10637296 |
| Marker95392 | LG7 | 10688636 | 10688586 | 10688686 |
| Marker95384 | LG7 | 10695291 | 10695241 | 10695341 |
| Marker96629 | LG7 | 11635661 | 11635611 | 11635711 |
| Marker96658 | LG7 | 11691532 | 11691482 | 11691582 |
| Marker96680 | LG7 | 11743538 | 11743488 | 11743588 |
| Marker96702 | LG7 | 11773306 | 11773256 | 11773356 |
| Marker96708 | LG7 | 11773979 | 11773929 | 11774029 |
| Marker96719 | LG7 | 11788399 | 11788349 | 11788449 |
| Marker96722 | LG7 | 11791929 | 11791879 | 11791979 |
| Marker96723 | LG7 | 11792167 | 11792117 | 11792217 |
| Marker96724 | LG7 | 11800360 | 11800310 | 11800410 |
| Marker32354 | LG7 | 12547871 | 12547821 | 12547921 |
| Marker32362 | LG7 | 12567424 | 12567374 | 12567474 |
| Marker32382 | LG7 | 12597777 | 12597727 | 12597827 |
| Marker32385 | LG7 | 12597978 | 12597928 | 12598028 |
| Marker32390 | LG7 | 12620919 | 12620869 | 12620969 |
| Marker32392 | LG7 | 12624936 | 12624886 | 12624986 |
| Marker32417 | LG7 | 12669161 | 12669111 | 12669211 |
| Marker32426 | LG7 | 12704211 | 12704161 | 12704261 |
| Marker32429 | LG7 | 12708918 | 12708868 | 12708968 |
| Marker32438 | LG7 | 12720067 | 12720017 | 12720117 |
| Marker32484 | LG7 | 12766503 | 12766453 | 12766553 |
| Marker32537 | LG7 | 12877920 | 12877870 | 12877970 |
| Marker32587 | LG7 | 12945697 | 12945647 | 12945747 |
| Marker32606 | LG7 | 12989289 | 12989239 | 12989339 |
| Marker32613 | LG7 | 13004353 | 13004303 | 13004403 |
| Marker32623 | LG7 | 13010480 | 13010430 | 13010530 |
| Marker32624 | LG7 | 13011608 | 13011558 | 13011658 |
| Marker32625 | LG7 | 13011867 | 13011817 | 13011917 |
| Marker96641 | LG7 | 13131285 | 13131235 | 13131335 |
| Marker96639 | LG7 | 13133302 | 13133252 | 13133352 |
| Marker96634 | LG7 | 13141120 | 13141070 | 13141170 |
| Marker96621 | LG7 | 13158822 | 13158772 | 13158872 |
| Marker96613 | LG7 | 13178471 | 13178421 | 13178521 |
| Marker96590 | LG7 | 13223605 | 13223555 | 13223655 |
| Marker96582 | LG7 | 13237313 | 13237263 | 13237363 |
| Marker96572 | LG7 | 13240755 | 13240705 | 13240805 |
| Marker96544 | LG7 | 13267202 | 13267152 | 13267252 |
| Marker96527 | LG7 | 13292152 | 13292102 | 13292202 |
| Marker96474 | LG7 | 13366635 | 13366585 | 13366685 |
| Marker96459 | LG7 | 13396829 | 13396779 | 13396879 |
| Marker96437 | LG7 | 13428943 | 13428893 | 13428993 |
| Marker96417 | LG7 | 13454944 | 13454894 | 13454994 |
| Marker96394 | LG7 | 13473102 | 13473052 | 13473152 |
| Marker96388 | LG7 | 13487783 | 13487733 | 13487833 |
| Marker96362 | LG7 | 13539171 | 13539121 | 13539221 |
| Marker96358 | LG7 | 13549867 | 13549817 | 13549917 |
| Marker96353 | LG7 | 13560450 | 13560400 | 13560500 |
| Marker96324 | LG7 | 13612538 | 13612488 | 13612588 |
| Marker96307 | LG7 | 13628178 | 13628128 | 13628228 |
| Marker96300 | LG7 | 13655810 | 13655760 | 13655860 |
| Marker96288 | LG7 | 13696081 | 13696031 | 13696131 |
| Marker96241 | LG7 | 13740076 | 13740026 | 13740126 |
| Marker96211 | LG7 | 13884226 | 13884176 | 13884276 |
| Marker96201 | LG7 | 13907860 | 13907810 | 13907910 |
| Marker96162 | LG7 | 13970082 | 13970032 | 13970132 |
| Marker96131 | LG7 | 14007277 | 14007227 | 14007327 |
| Marker96126 | LG7 | 14022250 | 14022200 | 14022300 |
| Marker96125 | LG7 | 14040132 | 14040082 | 14040182 |
| Marker96122 | LG7 | 14040671 | 14040621 | 14040721 |
| Marker96086 | LG7 | 14126135 | 14126085 | 14126185 |
| Marker96070 | LG7 | 14168212 | 14168162 | 14168262 |
| Marker96069 | LG7 | 14184538 | 14184488 | 14184588 |
| Marker96039 | LG7 | 14234715 | 14234665 | 14234765 |
| Marker96036 | LG7 | 14238667 | 14238617 | 14238717 |
| Marker95988 | LG7 | 14339753 | 14339703 | 14339803 |
| Marker95948 | LG7 | 14378222 | 14378172 | 14378272 |
| Marker95943 | LG7 | 14398242 | 14398192 | 14398292 |
| Marker116154 | LG7 | 14501768 | 14501718 | 14501818 |
| Marker116162 | LG7 | 14505276 | 14505226 | 14505326 |
| Marker116171 | LG7 | 14530666 | 14530616 | 14530716 |
| Marker97182 | LG7 | 14822793 | 14822743 | 14822843 |
| Marker97168 | LG7 | 14858241 | 14858191 | 14858291 |
| Marker97133 | LG7 | 14933384 | 14933334 | 14933434 |
| Marker97119 | LG7 | 14965369 | 14965319 | 14965419 |
| Marker97106 | LG7 | 14978138 | 14978088 | 14978188 |
| Marker97089 | LG7 | 14985940 | 14985890 | 14985990 |
| Marker97087 | LG7 | 14988314 | 14988264 | 14988364 |
| Marker97082 | LG7 | 14996900 | 14996850 | 14996950 |
| Marker97042 | LG7 | 15059415 | 15059365 | 15059465 |
| Marker97012 | LG7 | 15105935 | 15105885 | 15105985 |
| Marker96999 | LG7 | 15132128 | 15132078 | 15132178 |
| Marker96995 | LG7 | 15133528 | 15133478 | 15133578 |
| Marker96952 | LG7 | 15200734 | 15200684 | 15200784 |
| Marker96946 | LG7 | 15204427 | 15204377 | 15204477 |
| Marker96917 | LG7 | 15271211 | 15271161 | 15271261 |
| Marker96916 | LG7 | 15277011 | 15276961 | 15277061 |
| Marker96907 | LG7 | 15288013 | 15287963 | 15288063 |
| Marker96905 | LG7 | 15293516 | 15293466 | 15293566 |
| Marker96903 | LG7 | 15296307 | 15296257 | 15296357 |
| Marker96897 | LG7 | 15299943 | 15299893 | 15299993 |
| Marker96895 | LG7 | 15307891 | 15307841 | 15307941 |
| Marker96894 | LG7 | 15309486 | 15309436 | 15309536 |
| Marker96863 | LG7 | 15374111 | 15374061 | 15374161 |
| Marker97376 | LG7 | 15431373 | 15431323 | 15431423 |
| Marker97334 | LG7 | 15535063 | 15535013 | 15535113 |
| Marker97303 | LG7 | 15574878 | 15574828 | 15574928 |
| Marker97285 | LG7 | 15604146 | 15604096 | 15604196 |
| Marker97250 | LG7 | 15692656 | 15692606 | 15692706 |
| Marker97692 | LG7 | 15787973 | 15787923 | 15788023 |
| Marker97665 | LG7 | 15872684 | 15872634 | 15872734 |
| Marker97632 | LG7 | 15913570 | 15913520 | 15913620 |
| Marker97585 | LG7 | 15989349 | 15989299 | 15989399 |
| Marker97539 | LG7 | 16192615 | 16192565 | 16192665 |
| Marker97525 | LG7 | 16214403 | 16214353 | 16214453 |
| Marker97478 | LG7 | 16276922 | 16276872 | 16276972 |
| Marker97452 | LG7 | 16304831 | 16304781 | 16304881 |
| Marker97422 | LG7 | 16340115 | 16340065 | 16340165 |
| Marker97808 | LG7 | 16467138 | 16467088 | 16467188 |
| Marker97806 | LG7 | 16467343 | 16467293 | 16467393 |
| Marker97805 | LG7 | 16469100 | 16469050 | 16469150 |
| Marker97795 | LG7 | 16494368 | 16494318 | 16494418 |
| Marker97794 | LG7 | 16496482 | 16496432 | 16496532 |
| Marker97790 | LG7 | 16503935 | 16503885 | 16503985 |
| Marker97788 | LG7 | 16505373 | 16505323 | 16505423 |
| Marker97787 | LG7 | 16512529 | 16512479 | 16512579 |
| Marker97774 | LG7 | 16572217 | 16572167 | 16572267 |
| Marker97917 | LG7 | 16783026 | 16782976 | 16783076 |
| Marker97906 | LG7 | 16812824 | 16812774 | 16812874 |
| Marker97899 | LG7 | 16828621 | 16828571 | 16828671 |
| Marker97854 | LG7 | 16877609 | 16877559 | 16877659 |
| Marker97853 | LG7 | 16878433 | 16878383 | 16878483 |
| Marker97841 | LG7 | 16911437 | 16911387 | 16911487 |
| Marker97834 | LG7 | 16922655 | 16922605 | 16922705 |
| Marker97830 | LG7 | 17040230 | 17040180 | 17040280 |
| Marker97829 | LG7 | 17043427 | 17043377 | 17043477 |
| Marker97828 | LG7 | 17043616 | 17043566 | 17043666 |
| Marker110875 | LG7 | 17102615 | 17102565 | 17102665 |
| Marker110872 | LG7 | 17105095 | 17105045 | 17105145 |
| Marker110870 | LG7 | 17113178 | 17113128 | 17113228 |
| Marker110865 | LG7 | 17117154 | 17117104 | 17117204 |
| Marker110864 | LG7 | 17120012 | 17119962 | 17120062 |
| Marker110846 | LG7 | 17168997 | 17168947 | 17169047 |
| Marker110804 | LG7 | 17221071 | 17221021 | 17221121 |
| Marker110797 | LG7 | 17227357 | 17227307 | 17227407 |
| Marker110784 | LG7 | 17254309 | 17254259 | 17254359 |
| Marker98239 | LG7 | 17448415 | 17448365 | 17448465 |
| Marker98235 | LG7 | 17455821 | 17455771 | 17455871 |
| Marker98233 | LG7 | 17458489 | 17458439 | 17458539 |
| Marker98229 | LG7 | 17461954 | 17461904 | 17462004 |
| Marker98228 | LG7 | 17462295 | 17462245 | 17462345 |
| Marker98215 | LG7 | 17497679 | 17497629 | 17497729 |
| Marker98211 | LG7 | 17501020 | 17500970 | 17501070 |
| Marker98210 | LG7 | 17503365 | 17503315 | 17503415 |
| Marker98204 | LG7 | 17520934 | 17520884 | 17520984 |
| Marker98203 | LG7 | 17525931 | 17525881 | 17525981 |
| Marker98202 | LG7 | 17527716 | 17527666 | 17527766 |
| Marker98201 | LG7 | 17531098 | 17531048 | 17531148 |
| Marker98200 | LG7 | 17535175 | 17535125 | 17535225 |
| Marker98191 | LG7 | 17598583 | 17598533 | 17598633 |
| Marker98188 | LG7 | 17631936 | 17631886 | 17631986 |
| Marker98185 | LG7 | 17633162 | 17633112 | 17633212 |
| Marker98166 | LG7 | 17646811 | 17646761 | 17646861 |
| Marker98124 | LG7 | 17713286 | 17713236 | 17713336 |
| Marker98120 | LG7 | 17722916 | 17722866 | 17722966 |
| Marker98081 | LG7 | 17817702 | 17817652 | 17817752 |
| Marker98014 | LG7 | 17952602 | 17952552 | 17952652 |
| Marker97996 | LG7 | 18055474 | 18055424 | 18055524 |
| Marker97985 | LG7 | 18067303 | 18067253 | 18067353 |
| Marker98450 | LG7 | 18176016 | 18175966 | 18176066 |
| Marker98292 | LG7 | 18527684 | 18527634 | 18527734 |
| Marker98304 | LG7 | 18532557 | 18532507 | 18532607 |
| Marker98331 | LG7 | 18579097 | 18579047 | 18579147 |
| Marker98332 | LG7 | 18580538 | 18580488 | 18580588 |
| Marker98335 | LG7 | 18596379 | 18596329 | 18596429 |
| Marker98343 | LG7 | 18609622 | 18609572 | 18609672 |
| Marker98376 | LG7 | 18690847 | 18690797 | 18690897 |
| Marker102154 | LG8 | 6768 | 6718 | 6818 |
| Marker102170 | LG8 | 21894 | 21844 | 21944 |
| Marker102178 | LG8 | 36804 | 36754 | 36854 |
| Marker102186 | LG8 | 48934 | 48884 | 48984 |
| Marker102205 | LG8 | 83984 | 83934 | 84034 |
| Marker102209 | LG8 | 88165 | 88115 | 88215 |
| Marker102238 | LG8 | 127446 | 127396 | 127496 |
| Marker102239 | LG8 | 127665 | 127615 | 127715 |
| Marker102247 | LG8 | 131749 | 131699 | 131799 |
| Marker102256 | LG8 | 152955 | 152905 | 153005 |
| Marker117947 | LG8 | 185425 | 185375 | 185475 |
| Marker100547 | LG8 | 194693 | 194643 | 194743 |
| Marker118419 | LG8 | 248946 | 248896 | 248996 |
| Marker118416 | LG8 | 255440 | 255390 | 255490 |
| Marker118402 | LG8 | 278175 | 278125 | 278225 |
| Marker25801 | LG8 | 294592 | 294542 | 294642 |
| Marker25791 | LG8 | 341274 | 341224 | 341324 |
| Marker25776 | LG8 | 359172 | 359122 | 359222 |
| Marker25772 | LG8 | 359482 | 359432 | 359532 |
| Marker25769 | LG8 | 369400 | 369350 | 369450 |
| Marker25768 | LG8 | 369529 | 369479 | 369579 |
| Marker25760 | LG8 | 380053 | 380003 | 380103 |
| Marker25744 | LG8 | 395375 | 395325 | 395425 |
| Marker25732 | LG8 | 404895 | 404845 | 404945 |
| Marker25728 | LG8 | 407062 | 407012 | 407112 |
| Marker25725 | LG8 | 409520 | 409470 | 409570 |
| Marker25693 | LG8 | 439776 | 439726 | 439826 |
| Marker25683 | LG8 | 452666 | 452616 | 452716 |
| Marker111962 | LG8 | 711705 | 711655 | 711755 |
| Marker111975 | LG8 | 733616 | 733566 | 733666 |
| Marker112028 | LG8 | 805193 | 805143 | 805243 |
| Marker112057 | LG8 | 828669 | 828619 | 828719 |
| Marker112094 | LG8 | 900772 | 900722 | 900822 |
| Marker112130 | LG8 | 994735 | 994685 | 994785 |
| Marker112137 | LG8 | 1001422 | 1001372 | 1001472 |
| Marker112158 | LG8 | 1019705 | 1019655 | 1019755 |
| Marker112162 | LG8 | 1022320 | 1022270 | 1022370 |
| Marker99713 | LG8 | 1065645 | 1065595 | 1065695 |
| Marker99715 | LG8 | 1077356 | 1077306 | 1077406 |
| Marker100163 | LG8 | 1112890 | 1112840 | 1112940 |
| Marker100162 | LG8 | 1113704 | 1113654 | 1113754 |
| Marker100029 | LG8 | 1605568 | 1605518 | 1605618 |
| Marker100031 | LG8 | 1610625 | 1610575 | 1610675 |
| Marker100032 | LG8 | 1618863 | 1618813 | 1618913 |
| Marker100061 | LG8 | 1675897 | 1675847 | 1675947 |
| Marker100079 | LG8 | 1697361 | 1697311 | 1697411 |
| Marker100092 | LG8 | 1706226 | 1706176 | 1706276 |
| Marker100095 | LG8 | 1711718 | 1711668 | 1711768 |
| Marker100098 | LG8 | 1711901 | 1711851 | 1711951 |
| Marker115345 | LG8 | 1726335 | 1726285 | 1726385 |
| Marker115233 | LG8 | 1878139 | 1878089 | 1878189 |
| Marker111088 | LG8 | 1910506 | 1910456 | 1910556 |
| Marker111028 | LG8 | 1936772 | 1936722 | 1936822 |
| Marker110988 | LG8 | 1951920 | 1951870 | 1951970 |
| Marker110971 | LG8 | 1966428 | 1966378 | 1966478 |
| Marker110941 | LG8 | 2017872 | 2017822 | 2017922 |
| Marker110933 | LG8 | 2046642 | 2046592 | 2046692 |
| Marker110909 | LG8 | 2103978 | 2103928 | 2104028 |
| Marker110908 | LG8 | 2104710 | 2104660 | 2104760 |
| Marker110898 | LG8 | 2112604 | 2112554 | 2112654 |
| Marker110893 | LG8 | 2123992 | 2123942 | 2124042 |
| Marker99244 | LG8 | 2154105 | 2154055 | 2154155 |
| Marker99246 | LG8 | 2158800 | 2158750 | 2158850 |
| Marker99518 | LG8 | 2168017 | 2167967 | 2168067 |
| Marker99492 | LG8 | 2178511 | 2178461 | 2178561 |
| Marker99440 | LG8 | 2212726 | 2212676 | 2212776 |
| Marker99437 | LG8 | 2215303 | 2215253 | 2215353 |
| Marker118859 | LG8 | 2230443 | 2230393 | 2230493 |
| Marker98765 | LG8 | 2285218 | 2285168 | 2285268 |
| Marker98738 | LG8 | 2298616 | 2298566 | 2298666 |
| Marker98735 | LG8 | 2299620 | 2299570 | 2299670 |
| Marker98720 | LG8 | 2303419 | 2303369 | 2303469 |
| Marker98707 | LG8 | 2320589 | 2320539 | 2320639 |
| Marker98705 | LG8 | 2325209 | 2325159 | 2325259 |
| Marker98660 | LG8 | 2336450 | 2336400 | 2336500 |
| Marker98658 | LG8 | 2336702 | 2336652 | 2336752 |
| Marker98653 | LG8 | 2348357 | 2348307 | 2348407 |
| Marker98587 | LG8 | 2395564 | 2395514 | 2395614 |
| Marker99236 | LG8 | 2424809 | 2424759 | 2424859 |
| Marker99211 | LG8 | 2428690 | 2428640 | 2428740 |
| Marker99166 | LG8 | 2458638 | 2458588 | 2458688 |
| Marker99141 | LG8 | 2487276 | 2487226 | 2487326 |
| Marker99125 | LG8 | 2499385 | 2499335 | 2499435 |
| Marker99121 | LG8 | 2514561 | 2514511 | 2514611 |
| Marker99111 | LG8 | 2521510 | 2521460 | 2521560 |
| Marker99007 | LG8 | 2651569 | 2651519 | 2651619 |
| Marker99016 | LG8 | 2666745 | 2666695 | 2666795 |
| Marker99025 | LG8 | 2675865 | 2675815 | 2675915 |
| Marker65526 | LG8 | 2780599 | 2780549 | 2780649 |
| Marker99077 | LG8 | 2904104 | 2904054 | 2904154 |
| Marker85731 | LG8 | 3007423 | 3007373 | 3007473 |
| Marker99388 | LG8 | 3045172 | 3045122 | 3045222 |
| Marker111129 | LG8 | 3105659 | 3105609 | 3105709 |
| Marker111132 | LG8 | 3105956 | 3105906 | 3106006 |
| Marker111148 | LG8 | 3187201 | 3187151 | 3187251 |
| Marker111166 | LG8 | 3237795 | 3237745 | 3237845 |
| Marker111175 | LG8 | 3245749 | 3245699 | 3245799 |
| Marker111258 | LG8 | 3312512 | 3312462 | 3312562 |
| Marker111298 | LG8 | 3355531 | 3355481 | 3355581 |
| Marker111329 | LG8 | 3408807 | 3408757 | 3408857 |
| Marker111413 | LG8 | 3463148 | 3463098 | 3463198 |
| Marker111425 | LG8 | 3495668 | 3495618 | 3495718 |
| Marker111453 | LG8 | 3521987 | 3521937 | 3522037 |
| Marker111502 | LG8 | 3562910 | 3562860 | 3562960 |
| Marker111506 | LG8 | 3567311 | 3567261 | 3567361 |
| Marker111540 | LG8 | 3574098 | 3574048 | 3574148 |
| Marker111541 | LG8 | 3574278 | 3574228 | 3574328 |
| Marker111544 | LG8 | 3575247 | 3575197 | 3575297 |
| Marker111548 | LG8 | 3575764 | 3575714 | 3575814 |
| Marker111551 | LG8 | 3578149 | 3578099 | 3578199 |
| Marker111553 | LG8 | 3584268 | 3584218 | 3584318 |
| Marker111574 | LG8 | 3603148 | 3603098 | 3603198 |
| Marker111581 | LG8 | 3621854 | 3621804 | 3621904 |
| Marker111608 | LG8 | 3696707 | 3696657 | 3696757 |
| Marker111635 | LG8 | 3709596 | 3709546 | 3709646 |
| Marker111639 | LG8 | 3709789 | 3709739 | 3709839 |
| Marker111668 | LG8 | 3759601 | 3759551 | 3759651 |
| Marker111719 | LG8 | 3798567 | 3798517 | 3798617 |
| Marker111734 | LG8 | 3798824 | 3798774 | 3798874 |
| Marker6616 | LG8 | 3808364 | 3808314 | 3808414 |
| Marker99637 | LG8 | 3888861 | 3888811 | 3888911 |
| Marker99632 | LG8 | 3896943 | 3896893 | 3896993 |
| Marker99629 | LG8 | 3897984 | 3897934 | 3898034 |
| Marker99618 | LG8 | 3906988 | 3906938 | 3907038 |
| Marker99608 | LG8 | 3926603 | 3926553 | 3926653 |
| Marker99604 | LG8 | 3928115 | 3928065 | 3928165 |
| Marker99698 | LG8 | 3962459 | 3962409 | 3962509 |
| Marker99697 | LG8 | 3965968 | 3965918 | 3966018 |
| Marker99696 | LG8 | 3966126 | 3966076 | 3966176 |
| Marker100239 | LG8 | 4110546 | 4110496 | 4110596 |
| Marker100201 | LG8 | 4188432 | 4188382 | 4188482 |
| Marker99732 | LG8 | 4227545 | 4227495 | 4227595 |
| Marker99776 | LG8 | 4287690 | 4287640 | 4287740 |
| Marker99777 | LG8 | 4287698 | 4287648 | 4287748 |
| Marker99787 | LG8 | 4314023 | 4313973 | 4314073 |
| Marker99792 | LG8 | 4317480 | 4317430 | 4317530 |
| Marker99878 | LG8 | 4398274 | 4398224 | 4398324 |
| Marker99979 | LG8 | 4490620 | 4490570 | 4490670 |
| Marker99990 | LG8 | 4496193 | 4496143 | 4496243 |
| Marker99995 | LG8 | 4496408 | 4496358 | 4496458 |
| Marker100033 | LG8 | 4605363 | 4605313 | 4605413 |
| Marker99278 | LG8 | 4654901 | 4654851 | 4654951 |
| Marker99303 | LG8 | 4674553 | 4674503 | 4674603 |
| Marker99307 | LG8 | 4765560 | 4765510 | 4765610 |
| Marker99312 | LG8 | 4775258 | 4775208 | 4775308 |
| Marker99314 | LG8 | 4779113 | 4779063 | 4779163 |
| Marker99315 | LG8 | 4779312 | 4779262 | 4779362 |
| Marker119295 | LG8 | 4868261 | 4868211 | 4868311 |
| Marker99522 | LG8 | 4886770 | 4886720 | 4886820 |
| Marker99527 | LG8 | 4899436 | 4899386 | 4899486 |
| Marker99551 | LG8 | 4913228 | 4913178 | 4913278 |
| Marker99577 | LG8 | 5046364 | 5046314 | 5046414 |
| Marker99584 | LG8 | 5056552 | 5056502 | 5056602 |
| Marker99587 | LG8 | 5058496 | 5058446 | 5058546 |
| Marker28182 | LG8 | 5102370 | 5102320 | 5102420 |
| Marker112174 | LG8 | 5167355 | 5167305 | 5167405 |
| Marker112199 | LG8 | 5204990 | 5204940 | 5205040 |
| Marker112204 | LG8 | 5222783 | 5222733 | 5222833 |
| Marker112263 | LG8 | 5312787 | 5312737 | 5312837 |
| Marker112276 | LG8 | 5337563 | 5337513 | 5337613 |
| Marker112279 | LG8 | 5340477 | 5340427 | 5340527 |
| Marker112281 | LG8 | 5340645 | 5340595 | 5340695 |
| Marker101540 | LG8 | 5376736 | 5376686 | 5376786 |
| Marker101516 | LG8 | 5444686 | 5444636 | 5444736 |
| Marker101511 | LG8 | 5444948 | 5444898 | 5444998 |
| Marker101419 | LG8 | 5582490 | 5582440 | 5582540 |
| Marker101418 | LG8 | 5596321 | 5596271 | 5596371 |
| Marker101394 | LG8 | 5621066 | 5621016 | 5621116 |
| Marker101386 | LG8 | 5640008 | 5639958 | 5640058 |
| Marker101361 | LG8 | 5818426 | 5818376 | 5818476 |
| Marker101351 | LG8 | 5821904 | 5821854 | 5821954 |
| Marker101347 | LG8 | 5942452 | 5942402 | 5942502 |
| Marker101300 | LG8 | 5975908 | 5975858 | 5975958 |
| Marker101297 | LG8 | 5976160 | 5976110 | 5976210 |
| Marker101276 | LG8 | 6032287 | 6032237 | 6032337 |
| Marker101231 | LG8 | 6081741 | 6081691 | 6081791 |
| Marker101224 | LG8 | 6083963 | 6083913 | 6084013 |
| Marker101196 | LG8 | 6139511 | 6139461 | 6139561 |
| Marker101192 | LG8 | 6143449 | 6143399 | 6143499 |
| Marker101189 | LG8 | 6148394 | 6148344 | 6148444 |
| Marker101180 | LG8 | 6155470 | 6155420 | 6155520 |
| Marker101167 | LG8 | 6182242 | 6182192 | 6182292 |
| Marker101133 | LG8 | 6196417 | 6196367 | 6196467 |
| Marker101130 | LG8 | 6210494 | 6210444 | 6210544 |
| Marker101124 | LG8 | 6217315 | 6217265 | 6217365 |
| Marker101096 | LG8 | 6243212 | 6243162 | 6243262 |
| Marker120147 | LG8 | 6394604 | 6394554 | 6394654 |
| Marker120140 | LG8 | 6396442 | 6396392 | 6396492 |
| Marker120136 | LG8 | 6400381 | 6400331 | 6400431 |
| Marker114670 | LG8 | 6467765 | 6467715 | 6467815 |
| Marker114669 | LG8 | 6472774 | 6472724 | 6472824 |
| Marker114649 | LG8 | 6516969 | 6516919 | 6517019 |
| Marker114640 | LG8 | 6523714 | 6523664 | 6523764 |
| Marker112800 | LG8 | 6527948 | 6527898 | 6527998 |
| Marker46687 | LG8 | 6574983 | 6574933 | 6575033 |
| Marker46703 | LG8 | 6590656 | 6590606 | 6590706 |
| Marker46732 | LG8 | 6614603 | 6614553 | 6614653 |
| Marker46757 | LG8 | 6620659 | 6620609 | 6620709 |
| Marker46782 | LG8 | 6642432 | 6642382 | 6642482 |
| Marker46788 | LG8 | 6663659 | 6663609 | 6663709 |
| Marker100458 | LG8 | 6841210 | 6841160 | 6841260 |
| Marker100490 | LG8 | 6846847 | 6846797 | 6846897 |
| Marker100528 | LG8 | 6915041 | 6914991 | 6915091 |
| Marker100605 | LG8 | 6957230 | 6957180 | 6957280 |
| Marker100611 | LG8 | 6957529 | 6957479 | 6957579 |
| Marker100367 | LG8 | 7045451 | 7045401 | 7045501 |
| Marker100340 | LG8 | 7068601 | 7068551 | 7068651 |
| Marker100320 | LG8 | 7184625 | 7184575 | 7184675 |
| Marker100309 | LG8 | 7220792 | 7220742 | 7220842 |
| Marker100305 | LG8 | 7224638 | 7224588 | 7224688 |
| Marker100294 | LG8 | 7244765 | 7244715 | 7244815 |
| Marker100279 | LG8 | 7285815 | 7285765 | 7285865 |
| Marker100658 | LG8 | 7325636 | 7325586 | 7325686 |
| Marker100671 | LG8 | 7336717 | 7336667 | 7336767 |
| Marker100674 | LG8 | 7337432 | 7337382 | 7337482 |
| Marker100747 | LG8 | 7471985 | 7471935 | 7472035 |
| Marker100820 | LG8 | 7569554 | 7569504 | 7569604 |
| Marker100823 | LG8 | 7569614 | 7569564 | 7569664 |
| Marker100936 | LG8 | 7752106 | 7752056 | 7752156 |
| Marker100937 | LG8 | 7752285 | 7752235 | 7752335 |
| Marker100957 | LG8 | 7803209 | 7803159 | 7803259 |
| Marker100967 | LG8 | 7811855 | 7811805 | 7811905 |
| Marker100988 | LG8 | 7842201 | 7842151 | 7842251 |
| Marker8848 | LG8 | 7917043 | 7916993 | 7917093 |
| Marker116792 | LG8 | 7943054 | 7943004 | 7943104 |
| Marker108066 | LG8 | 7973815 | 7973765 | 7973865 |
| Marker108072 | LG8 | 8019902 | 8019852 | 8019952 |
| Marker108076 | LG8 | 8032182 | 8032132 | 8032232 |
| Marker108080 | LG8 | 8035946 | 8035896 | 8035996 |
| Marker108091 | LG8 | 8044068 | 8044018 | 8044118 |
| Marker108093 | LG8 | 8044991 | 8044941 | 8045041 |
| Marker108121 | LG8 | 8085581 | 8085531 | 8085631 |
| Marker108125 | LG8 | 8099228 | 8099178 | 8099278 |
| Marker108126 | LG8 | 8122365 | 8122315 | 8122415 |
| Marker115019 | LG8 | 8297705 | 8297655 | 8297755 |
| Marker114954 | LG8 | 8388480 | 8388430 | 8388530 |
| Marker114950 | LG8 | 8393048 | 8392998 | 8393098 |
| Marker101772 | LG8 | 8536993 | 8536943 | 8537043 |
| Marker101770 | LG8 | 8537502 | 8537452 | 8537552 |
| Marker101740 | LG8 | 8606888 | 8606838 | 8606938 |
| Marker101675 | LG8 | 8662011 | 8661961 | 8662061 |
| Marker101674 | LG8 | 8663380 | 8663330 | 8663430 |
| Marker101673 | LG8 | 8673820 | 8673770 | 8673870 |
| Marker101662 | LG8 | 8675818 | 8675768 | 8675868 |
| Marker101636 | LG8 | 8710255 | 8710205 | 8710305 |
| Marker101008 | LG8 | 8841901 | 8841851 | 8841951 |
| Marker101033 | LG8 | 8895084 | 8895034 | 8895134 |
| Marker101564 | LG8 | 8928314 | 8928264 | 8928364 |
| Marker101013 | LG8 | 8934984 | 8934934 | 8935034 |
| Marker101837 | LG8 | 9082062 | 9082012 | 9082112 |
| Marker101842 | LG8 | 9082345 | 9082295 | 9082395 |
| Marker101850 | LG8 | 9086973 | 9086923 | 9087023 |
| Marker101867 | LG8 | 9111727 | 9111677 | 9111777 |
| Marker101869 | LG8 | 9111940 | 9111890 | 9111990 |
| Marker101871 | LG8 | 9118975 | 9118925 | 9119025 |
| Marker101896 | LG8 | 9135379 | 9135329 | 9135429 |
| Marker101902 | LG8 | 9137560 | 9137510 | 9137610 |
| Marker101912 | LG8 | 9140814 | 9140764 | 9140864 |
| Marker101945 | LG8 | 9225306 | 9225256 | 9225356 |
| Marker101954 | LG8 | 9240143 | 9240093 | 9240193 |
| Marker101963 | LG8 | 9250598 | 9250548 | 9250648 |
| Marker101964 | LG8 | 9251397 | 9251347 | 9251447 |
| Marker102008 | LG8 | 9299658 | 9299608 | 9299708 |
| Marker102021 | LG8 | 9331508 | 9331458 | 9331558 |
| Marker102022 | LG8 | 9331769 | 9331719 | 9331819 |
| Marker102023 | LG8 | 9332604 | 9332554 | 9332654 |
| Marker102028 | LG8 | 9336221 | 9336171 | 9336271 |
| Marker102060 | LG8 | 9406830 | 9406780 | 9406880 |
| Marker102119 | LG8 | 9454966 | 9454916 | 9455016 |
| Marker102125 | LG8 | 9462523 | 9462473 | 9462573 |
| Marker102678 | LG8 | 9557468 | 9557418 | 9557518 |
| Marker102700 | LG8 | 9614541 | 9614491 | 9614591 |
| Marker102710 | LG8 | 9628293 | 9628243 | 9628343 |
| Marker102736 | LG8 | 9644340 | 9644290 | 9644390 |
| Marker102639 | LG8 | 9801803 | 9801753 | 9801853 |
| Marker102605 | LG8 | 9833510 | 9833460 | 9833560 |
| Marker102596 | LG8 | 9836221 | 9836171 | 9836271 |
| Marker102529 | LG8 | 9992210 | 9992160 | 9992260 |
| Marker102419 | LG8 | 10174693 | 10174643 | 10174743 |
| Marker102345 | LG8 | 10263559 | 10263509 | 10263609 |
| Marker102341 | LG8 | 10275222 | 10275172 | 10275272 |
| Marker103317 | LG8 | 10328290 | 10328240 | 10328340 |
| Marker103304 | LG8 | 10364028 | 10363978 | 10364078 |
| Marker103287 | LG8 | 10388872 | 10388822 | 10388922 |
| Marker103285 | LG8 | 10389169 | 10389119 | 10389219 |
| Marker24627 | LG8 | 10562324 | 10562274 | 10562374 |
| Marker103162 | LG8 | 10611991 | 10611941 | 10612041 |
| Marker103158 | LG8 | 10614417 | 10614367 | 10614467 |
| Marker103115 | LG8 | 10679626 | 10679576 | 10679676 |
| Marker103063 | LG8 | 10816905 | 10816855 | 10816955 |
| Marker103056 | LG8 | 10843337 | 10843287 | 10843387 |
| Marker103034 | LG8 | 10877467 | 10877417 | 10877517 |
| Marker103020 | LG8 | 10925338 | 10925288 | 10925388 |
| Marker103007 | LG8 | 10943519 | 10943469 | 10943569 |
| Marker102964 | LG8 | 10965067 | 10965017 | 10965117 |
| Marker102943 | LG8 | 11011707 | 11011657 | 11011757 |
| Marker102942 | LG8 | 11014105 | 11014055 | 11014155 |
| Marker102940 | LG8 | 11014744 | 11014694 | 11014794 |
| Marker102933 | LG8 | 11020750 | 11020700 | 11020800 |
| Marker102932 | LG8 | 11020825 | 11020775 | 11020875 |
| Marker102914 | LG8 | 11027795 | 11027745 | 11027845 |
| Marker102905 | LG8 | 11030724 | 11030674 | 11030774 |
| Marker102836 | LG8 | 11151767 | 11151717 | 11151817 |
| Marker102832 | LG8 | 11161388 | 11161338 | 11161438 |
| Marker102816 | LG8 | 11179432 | 11179382 | 11179482 |
| Marker102797 | LG8 | 11195571 | 11195521 | 11195621 |
| Marker102782 | LG8 | 11215305 | 11215255 | 11215355 |
| Marker102775 | LG8 | 11225202 | 11225152 | 11225252 |
| Marker102773 | LG8 | 11229032 | 11228982 | 11229082 |
| Marker102766 | LG8 | 11235945 | 11235895 | 11235995 |
| Marker102765 | LG8 | 11238599 | 11238549 | 11238649 |
| Marker108829 | LG8 | 11362581 | 11362531 | 11362631 |
| Marker108836 | LG8 | 11392851 | 11392801 | 11392901 |
| Marker108859 | LG8 | 11415847 | 11415797 | 11415897 |
| Marker108863 | LG8 | 11437054 | 11437004 | 11437104 |
| Marker108864 | LG8 | 11438138 | 11438088 | 11438188 |
| Marker108868 | LG8 | 11443165 | 11443115 | 11443215 |
| Marker108872 | LG8 | 11455994 | 11455944 | 11456044 |
| Marker108892 | LG8 | 11484444 | 11484394 | 11484494 |
| Marker108894 | LG8 | 11489559 | 11489509 | 11489609 |
| Marker108908 | LG8 | 11497511 | 11497461 | 11497561 |
| Marker108939 | LG8 | 11508050 | 11508000 | 11508100 |
| Marker108945 | LG8 | 11527561 | 11527511 | 11527611 |
| Marker108961 | LG8 | 11548912 | 11548862 | 11548962 |
| Marker108963 | LG8 | 11550120 | 11550070 | 11550170 |
| Marker108970 | LG8 | 11556123 | 11556073 | 11556173 |
| Marker108998 | LG8 | 11590879 | 11590829 | 11590929 |
| Marker109011 | LG8 | 11606878 | 11606828 | 11606928 |
| Marker109013 | LG8 | 11607103 | 11607053 | 11607153 |
| Marker109053 | LG8 | 11666842 | 11666792 | 11666892 |
| Marker109056 | LG8 | 11698183 | 11698133 | 11698233 |
| Marker109062 | LG8 | 11703240 | 11703190 | 11703290 |
| Marker109063 | LG8 | 11705340 | 11705290 | 11705390 |
| Marker103575 | LG8 | 11746352 | 11746302 | 11746402 |
| Marker103574 | LG8 | 11753383 | 11753333 | 11753433 |
| Marker103573 | LG8 | 11760315 | 11760265 | 11760365 |
| Marker103555 | LG8 | 11786474 | 11786424 | 11786524 |
| Marker103533 | LG8 | 11800073 | 11800023 | 11800123 |
| Marker103530 | LG8 | 11800295 | 11800245 | 11800345 |
| Marker103514 | LG8 | 11829568 | 11829518 | 11829618 |
| Marker103500 | LG8 | 11870015 | 11869965 | 11870065 |
| Marker103498 | LG8 | 11874176 | 11874126 | 11874226 |
| Marker103497 | LG8 | 11876751 | 11876701 | 11876801 |
| Marker103491 | LG8 | 11893121 | 11893071 | 11893171 |
| Marker103480 | LG8 | 11925343 | 11925293 | 11925393 |
| Marker103454 | LG8 | 11944190 | 11944140 | 11944240 |
| Marker103421 | LG8 | 11982715 | 11982665 | 11982765 |
| Marker103418 | LG8 | 11987277 | 11987227 | 11987327 |
| Marker103406 | LG8 | 12008417 | 12008367 | 12008467 |
| Marker103629 | LG8 | 12066692 | 12066642 | 12066742 |
| Marker48204 | LG8 | 12220235 | 12220185 | 12220285 |
| Marker48538 | LG8 | 12238450 | 12238400 | 12238500 |
| Marker48542 | LG8 | 12238714 | 12238664 | 12238764 |
| Marker48545 | LG8 | 12239171 | 12239121 | 12239221 |
| Marker48594 | LG8 | 12427510 | 12427460 | 12427560 |
| Marker48596 | LG8 | 12427754 | 12427704 | 12427804 |
| Marker48602 | LG8 | 12449054 | 12449004 | 12449104 |
| Marker103692 | LG8 | 12546387 | 12546337 | 12546437 |
| Marker103694 | LG8 | 12560024 | 12559974 | 12560074 |
| Marker103713 | LG8 | 12585446 | 12585396 | 12585496 |
| Marker103716 | LG8 | 12590525 | 12590475 | 12590575 |
| Marker103737 | LG8 | 12618377 | 12618327 | 12618427 |
| Marker103739 | LG8 | 12618603 | 12618553 | 12618653 |
| Marker103743 | LG8 | 12624800 | 12624750 | 12624850 |
| Marker103766 | LG8 | 12656098 | 12656048 | 12656148 |
| Marker103793 | LG8 | 12714083 | 12714033 | 12714133 |
| Marker103801 | LG8 | 12738710 | 12738660 | 12738760 |
| Marker103814 | LG8 | 12755274 | 12755224 | 12755324 |
| Marker103835 | LG8 | 12796359 | 12796309 | 12796409 |
| Marker103843 | LG8 | 12803385 | 12803335 | 12803435 |
| Marker103859 | LG8 | 12810264 | 12810214 | 12810314 |
| Marker103862 | LG8 | 12811921 | 12811871 | 12811971 |
| Marker103889 | LG8 | 12873489 | 12873439 | 12873539 |
| Marker103921 | LG8 | 12948792 | 12948742 | 12948842 |
| Marker103946 | LG8 | 12955131 | 12955081 | 12955181 |
| Marker103951 | LG8 | 12968613 | 12968563 | 12968663 |
| Marker103980 | LG8 | 13028439 | 13028389 | 13028489 |
| Marker103981 | LG8 | 13028695 | 13028645 | 13028745 |
| Marker103990 | LG8 | 13048337 | 13048287 | 13048387 |
| Marker103991 | LG8 | 13050339 | 13050289 | 13050389 |
| Marker103994 | LG8 | 13060059 | 13060009 | 13060109 |
| Marker104017 | LG8 | 13082034 | 13081984 | 13082084 |
| Marker104027 | LG8 | 13084583 | 13084533 | 13084633 |
| Marker104053 | LG8 | 13138010 | 13137960 | 13138060 |
| Marker104056 | LG8 | 13147012 | 13146962 | 13147062 |
| Marker104058 | LG8 | 13156020 | 13155970 | 13156070 |
| Marker104076 | LG8 | 13156859 | 13156809 | 13156909 |
| Marker104080 | LG8 | 13158534 | 13158484 | 13158584 |
| Marker104086 | LG8 | 13162575 | 13162525 | 13162625 |
| Marker104092 | LG8 | 13172610 | 13172560 | 13172660 |
| Marker104227 | LG8 | 13415530 | 13415480 | 13415580 |
| Marker104212 | LG8 | 13431176 | 13431126 | 13431226 |
| Marker104199 | LG8 | 13463125 | 13463075 | 13463175 |
| Marker85343 | LG8 | 13767760 | 13767710 | 13767810 |
| Marker85311 | LG8 | 13815128 | 13815078 | 13815178 |
| Marker84589 | LG8 | 13832970 | 13832920 | 13833020 |
| Marker84583 | LG8 | 13854986 | 13854936 | 13855036 |
| Marker84574 | LG8 | 13890878 | 13890828 | 13890928 |
| Marker84563 | LG8 | 13928390 | 13928340 | 13928440 |
| Marker84551 | LG8 | 13936956 | 13936906 | 13937006 |
| Marker84518 | LG8 | 13986008 | 13985958 | 13986058 |
| Marker85651 | LG8 | 14079134 | 14079084 | 14079184 |
| Marker84477 | LG8 | 14313140 | 14313090 | 14313190 |
| Marker84476 | LG8 | 14317103 | 14317053 | 14317153 |
| Marker84472 | LG8 | 14324424 | 14324374 | 14324474 |
| Marker84467 | LG8 | 14336328 | 14336278 | 14336378 |
| Marker84451 | LG8 | 14347709 | 14347659 | 14347759 |
| Marker84446 | LG8 | 14350181 | 14350131 | 14350231 |
| Marker104640 | LG8 | 14577615 | 14577565 | 14577665 |
| Marker104645 | LG8 | 14584064 | 14584014 | 14584114 |
| Marker104646 | LG8 | 14586428 | 14586378 | 14586478 |
| Marker104665 | LG8 | 14605205 | 14605155 | 14605255 |
| Marker104347 | LG8 | 14687201 | 14687151 | 14687251 |
| Marker104363 | LG8 | 14732982 | 14732932 | 14733032 |
| Marker104436 | LG8 | 14855256 | 14855206 | 14855306 |
| Marker104448 | LG8 | 14884533 | 14884483 | 14884583 |
| Marker104449 | LG8 | 14884748 | 14884698 | 14884798 |
| Marker80355 | LG8 | 14886993 | 14886943 | 14887043 |
| Marker104482 | LG8 | 14925547 | 14925497 | 14925597 |
| Marker104705 | LG8 | 15081150 | 15081100 | 15081200 |
| Marker104715 | LG8 | 15095680 | 15095630 | 15095730 |
| Marker104716 | LG8 | 15104371 | 15104321 | 15104421 |
| Marker104727 | LG8 | 15119370 | 15119320 | 15119420 |
| Marker104733 | LG8 | 15124425 | 15124375 | 15124475 |
| Marker104734 | LG8 | 15125456 | 15125406 | 15125506 |
| Marker104745 | LG8 | 15140440 | 15140390 | 15140490 |
| Marker104746 | LG8 | 15140676 | 15140626 | 15140726 |
| Marker104749 | LG8 | 15148652 | 15148602 | 15148702 |
| Marker104774 | LG8 | 15181036 | 15180986 | 15181086 |
| Marker104793 | LG8 | 15199053 | 15199003 | 15199103 |
| Marker104825 | LG8 | 15240178 | 15240128 | 15240228 |
| Marker104885 | LG8 | 15308546 | 15308496 | 15308596 |
| Marker104953 | LG8 | 15416404 | 15416354 | 15416454 |
| Marker104955 | LG8 | 15416630 | 15416580 | 15416680 |
| Marker104970 | LG8 | 15443198 | 15443148 | 15443248 |
| Marker104971 | LG8 | 15447060 | 15447010 | 15447110 |
| Marker105021 | LG8 | 15465133 | 15465083 | 15465183 |
| Marker105055 | LG8 | 15525862 | 15525812 | 15525912 |
| Marker105074 | LG8 | 15552061 | 15552011 | 15552111 |
| Marker105088 | LG8 | 15576437 | 15576387 | 15576487 |
| Marker105097 | LG8 | 15600103 | 15600053 | 15600153 |
| Marker105120 | LG8 | 15629762 | 15629712 | 15629812 |
| Marker105135 | LG8 | 15676953 | 15676903 | 15677003 |
| Marker105137 | LG8 | 15678858 | 15678808 | 15678908 |
| Marker105156 | LG8 | 15699925 | 15699875 | 15699975 |
| Marker105167 | LG8 | 15720425 | 15720375 | 15720475 |
| Marker105169 | LG8 | 15726683 | 15726633 | 15726733 |
| Marker105195 | LG8 | 15781579 | 15781529 | 15781629 |
| Marker105197 | LG8 | 15786103 | 15786053 | 15786153 |
| Marker105215 | LG8 | 15819428 | 15819378 | 15819478 |
| Marker105232 | LG8 | 15828103 | 15828053 | 15828153 |
| Marker105247 | LG8 | 15865272 | 15865222 | 15865322 |
| Marker105253 | LG8 | 15877756 | 15877706 | 15877806 |
| Marker105278 | LG8 | 15926208 | 15926158 | 15926258 |
| Marker105288 | LG8 | 15938467 | 15938417 | 15938517 |
| Marker105305 | LG8 | 15979548 | 15979498 | 15979598 |
| Marker105307 | LG8 | 15979806 | 15979756 | 15979856 |
| Marker105325 | LG8 | 16002824 | 16002774 | 16002874 |
| Marker105338 | LG8 | 16022249 | 16022199 | 16022299 |
| Marker105386 | LG8 | 16109776 | 16109726 | 16109826 |
| Marker105420 | LG8 | 16161815 | 16161765 | 16161865 |
| Marker105427 | LG8 | 16201689 | 16201639 | 16201739 |
| Marker105428 | LG8 | 16206408 | 16206358 | 16206458 |
| Marker105458 | LG8 | 16259695 | 16259645 | 16259745 |
| Marker105471 | LG8 | 16300104 | 16300054 | 16300154 |
| Marker105497 | LG8 | 16336581 | 16336531 | 16336631 |
| Marker105498 | LG8 | 16336765 | 16336715 | 16336815 |
| Marker105508 | LG8 | 16348009 | 16347959 | 16348059 |
| Marker105510 | LG8 | 16349656 | 16349606 | 16349706 |
| Marker105516 | LG8 | 16351609 | 16351559 | 16351659 |
| Marker105519 | LG8 | 16351888 | 16351838 | 16351938 |
| Marker105520 | LG8 | 16354662 | 16354612 | 16354712 |
| Marker105883 | LG8 | 16378363 | 16378313 | 16378413 |
| Marker105857 | LG8 | 16404955 | 16404905 | 16405005 |
| Marker105854 | LG8 | 16412346 | 16412296 | 16412396 |
| Marker105837 | LG8 | 16477157 | 16477107 | 16477207 |
| Marker105832 | LG8 | 16493335 | 16493285 | 16493385 |
| Marker105812 | LG8 | 16533953 | 16533903 | 16534003 |
| Marker105785 | LG8 | 16583757 | 16583707 | 16583807 |
| Marker105763 | LG8 | 16616137 | 16616087 | 16616187 |
| Marker105757 | LG8 | 16642539 | 16642489 | 16642589 |
| Marker105745 | LG8 | 16674556 | 16674506 | 16674606 |
| Marker105715 | LG8 | 16689519 | 16689469 | 16689569 |
| Marker105707 | LG8 | 16695251 | 16695201 | 16695301 |
| Marker105632 | LG8 | 16884417 | 16884367 | 16884467 |
| Marker105588 | LG8 | 17012315 | 17012265 | 17012365 |
| Marker105559 | LG8 | 17042267 | 17042217 | 17042317 |
| Marker105907 | LG8 | 17160613 | 17160563 | 17160663 |
| Marker105914 | LG8 | 17171068 | 17171018 | 17171118 |
| Marker105928 | LG8 | 17206675 | 17206625 | 17206725 |
| Marker105937 | LG8 | 17223743 | 17223693 | 17223793 |
| Marker105942 | LG8 | 17229681 | 17229631 | 17229731 |
| Marker46856 | LG8 | 17254601 | 17254551 | 17254651 |
| Marker105966 | LG8 | 17277769 | 17277719 | 17277819 |
| Marker106006 | LG8 | 17363091 | 17363041 | 17363141 |
| Marker106007 | LG8 | 17367856 | 17367806 | 17367906 |
| Marker106025 | LG8 | 17429049 | 17428999 | 17429099 |
| Marker106041 | LG8 | 17455451 | 17455401 | 17455501 |
| Marker106322 | LG8 | 17657871 | 17657821 | 17657921 |
| Marker106321 | LG8 | 17665876 | 17665826 | 17665926 |
| Marker106320 | LG8 | 17680702 | 17680652 | 17680752 |
| Marker106313 | LG8 | 17696539 | 17696489 | 17696589 |
| Marker106299 | LG8 | 17721895 | 17721845 | 17721945 |
| Marker106570 | LG8 | 18046036 | 18045986 | 18046086 |
| Marker106072 | LG8 | 18063887 | 18063837 | 18063937 |
| Marker106088 | LG8 | 18097579 | 18097529 | 18097629 |
| Marker106650 | LG8 | 18510956 | 18510906 | 18511006 |
| Marker106648 | LG8 | 18518106 | 18518056 | 18518156 |
| Marker106638 | LG8 | 18564511 | 18564461 | 18564561 |
| Marker106630 | LG8 | 18568025 | 18567975 | 18568075 |
| Marker106627 | LG8 | 18572866 | 18572816 | 18572916 |
| Marker106621 | LG8 | 18589391 | 18589341 | 18589441 |
| Marker106611 | LG8 | 18632995 | 18632945 | 18633045 |
| Marker106609 | LG8 | 18634201 | 18634151 | 18634251 |
| Marker106607 | LG8 | 18635219 | 18635169 | 18635269 |
| Marker106606 | LG8 | 18638173 | 18638123 | 18638223 |
| Marker106604 | LG8 | 18643456 | 18643406 | 18643506 |
| Marker106409 | LG8 | 18777913 | 18777863 | 18777963 |
| Marker106422 | LG8 | 18834154 | 18834104 | 18834204 |
| Marker106683 | LG8 | 19013963 | 19013913 | 19014013 |
| Marker106689 | LG8 | 19016273 | 19016223 | 19016323 |
| Marker106692 | LG8 | 19017622 | 19017572 | 19017672 |
| Marker107001 | LG8 | 19826682 | 19826632 | 19826732 |
| Marker107023 | LG8 | 19880730 | 19880680 | 19880780 |
| Marker107040 | LG8 | 19934359 | 19934309 | 19934409 |
| Marker107051 | LG8 | 19956393 | 19956343 | 19956443 |
| Marker107053 | LG8 | 19963774 | 19963724 | 19963824 |
| Marker107074 | LG8 | 20039074 | 20039024 | 20039124 |
| Marker107085 | LG8 | 20086719 | 20086669 | 20086769 |
| Marker107092 | LG8 | 20110984 | 20110934 | 20111034 |
| Marker107111 | LG8 | 20160446 | 20160396 | 20160496 |
| Marker107116 | LG8 | 20176267 | 20176217 | 20176317 |
| Marker107187 | LG8 | 20263594 | 20263544 | 20263644 |
| Marker107189 | LG8 | 20271963 | 20271913 | 20272013 |
| Marker107226 | LG8 | 20325906 | 20325856 | 20325956 |
| Marker107228 | LG8 | 20329631 | 20329581 | 20329681 |
| Marker107233 | LG8 | 20336610 | 20336560 | 20336660 |
| Marker107264 | LG8 | 20439217 | 20439167 | 20439267 |
| Marker107273 | LG8 | 20445348 | 20445298 | 20445398 |
| Marker107301 | LG8 | 20521227 | 20521177 | 20521277 |
| Marker107317 | LG8 | 20540490 | 20540440 | 20540540 |
| Marker107342 | LG8 | 20562237 | 20562187 | 20562287 |
| Marker107354 | LG8 | 20600520 | 20600470 | 20600570 |
| Marker107359 | LG8 | 20613528 | 20613478 | 20613578 |
| Marker107361 | LG8 | 20621487 | 20621437 | 20621537 |
| Marker107368 | LG8 | 20638454 | 20638404 | 20638504 |
| Marker107388 | LG8 | 20678105 | 20678055 | 20678155 |
| Marker107393 | LG8 | 20681869 | 20681819 | 20681919 |
| Marker107400 | LG8 | 20690950 | 20690900 | 20691000 |
| Marker107419 | LG8 | 20711266 | 20711216 | 20711316 |
| Marker107428 | LG8 | 20732093 | 20732043 | 20732143 |
| Marker107435 | LG8 | 20746521 | 20746471 | 20746571 |
| Marker107475 | LG8 | 20790006 | 20789956 | 20790056 |
| Marker107476 | LG8 | 20791438 | 20791388 | 20791488 |
| Marker107541 | LG8 | 21009381 | 21009331 | 21009431 |
| Marker107554 | LG8 | 21017768 | 21017718 | 21017818 |
| Marker107565 | LG8 | 21052989 | 21052939 | 21053039 |
| Marker107573 | LG8 | 21068252 | 21068202 | 21068302 |
| Marker107584 | LG8 | 21084788 | 21084738 | 21084838 |
| Marker107585 | LG8 | 21087115 | 21087065 | 21087165 |
| Marker107591 | LG8 | 21110475 | 21110425 | 21110525 |
| Marker107598 | LG8 | 21116259 | 21116209 | 21116309 |
| Marker107617 | LG8 | 21151265 | 21151215 | 21151315 |
| Marker107621 | LG8 | 21157091 | 21157041 | 21157141 |
| Marker107626 | LG8 | 21161691 | 21161641 | 21161741 |
| Marker107998 | LG8 | 21201247 | 21201197 | 21201297 |
| Marker108013 | LG8 | 21235812 | 21235762 | 21235862 |
| Marker108054 | LG8 | 21355867 | 21355817 | 21355917 |
| Marker119972 | LG8 | 21371388 | 21371338 | 21371438 |
| Marker108006 | LG8 | 21449517 | 21449467 | 21449567 |
| Marker108001 | LG8 | 21464559 | 21464509 | 21464609 |
| Marker107995 | LG8 | 21468517 | 21468467 | 21468567 |
| Marker107985 | LG8 | 21478501 | 21478451 | 21478551 |
| Marker107983 | LG8 | 21494265 | 21494215 | 21494315 |
| Marker107981 | LG8 | 21496613 | 21496563 | 21496663 |
| Marker107967 | LG8 | 21556035 | 21555985 | 21556085 |
| Marker107957 | LG8 | 21561386 | 21561336 | 21561436 |
| Marker107951 | LG8 | 21566693 | 21566643 | 21566743 |
| Marker107945 | LG8 | 21572304 | 21572254 | 21572354 |
| Marker107929 | LG8 | 21599719 | 21599669 | 21599769 |
| Marker107900 | LG8 | 21656451 | 21656401 | 21656501 |
| Marker107899 | LG8 | 21656499 | 21656449 | 21656549 |
| Marker107890 | LG8 | 21671953 | 21671903 | 21672003 |
| Marker107877 | LG8 | 21709393 | 21709343 | 21709443 |
| Marker107874 | LG8 | 21731602 | 21731552 | 21731652 |
| Marker107868 | LG8 | 21755039 | 21754989 | 21755089 |
| Marker107865 | LG8 | 21758650 | 21758600 | 21758700 |
| Marker107856 | LG8 | 21768340 | 21768290 | 21768390 |
| Marker107759 | LG8 | 21880479 | 21880429 | 21880529 |
| Marker107755 | LG8 | 21880683 | 21880633 | 21880733 |
| Marker107738 | LG8 | 21929765 | 21929715 | 21929815 |
| Marker107732 | LG8 | 21932738 | 21932688 | 21932788 |
| Marker107731 | LG8 | 21932951 | 21932901 | 21933001 |
| Marker107719 | LG8 | 21953611 | 21953561 | 21953661 |
| Marker107699 | LG8 | 22002070 | 22002020 | 22002120 |
| Marker107697 | LG8 | 22002975 | 22002925 | 22003025 |
| Marker107688 | LG8 | 22022612 | 22022562 | 22022662 |
| Marker107662 | LG8 | 22050239 | 22050189 | 22050289 |
| Marker107660 | LG8 | 22053525 | 22053475 | 22053575 |
| Marker107656 | LG8 | 22071677 | 22071627 | 22071727 |
| Marker107654 | LG8 | 22071701 | 22071651 | 22071751 |
| Marker107649 | LG8 | 22083250 | 22083200 | 22083300 |
