# Supplementary material for: Construction of a High-Density Genetic Map and Identification of Quantitative Trait Loci Linked to Fruit Quality Traits in Apricots Using Specific-Locus Amplified Fragment Sequencing
Source: Front Plant Sci. 2022 Feb 14;13:798700. doi: 10.3389/fpls.2022.798700 (PMC8882730; doi:10.3389/fpls.2022.798700)
Supplement: Supplementary file 6 [file Table_6.docx]

**Supplementary Table 6.** **Summary of the markers linked to QTLs of fruit quality traits（LOD>3.0,and LOD>2.5 in SSC and FF19）**

| Trait | Group | Position | Marker | LOD | Expl. % | | Trait | Group | Position | Marker | LOD | Expl. % |
| --- | --- | --- | --- | --- | --- | --- | --- | --- | --- | --- | --- | --- |
| FW18 | Hg2 | 19.3 | Marker68245 | 3.27 | 8.60 |  | FW18 | Sg2 | 32.5 | Marker121490 | 3.14 | 8.20 |
| FW18 | Hg3 | 34.3 | Marker58893 | 3.41 | 8.90 |  |  |  |  |  |  |  |
| FW18 | Hg4 | 36.4 | Marker118377 | 3.28 | 8.60 |  | FW18 | Sg4 | 42.2-44.2 | Marker40475, 40397, 119270 | 3.14 | 8.20 |
| FW18 | Hg4 | 39.9-40.4 | Marker42385, 42775 | 3.35 | 8.80 |  | FW18 | Sg4 | 58.5-59.4 | Marker39211, 39549 | 3.21 | 8.40 |
| FW18 | Hg4 | 40.4-42.5 | Marker42494, 42596 | 4.05 | 10.50 |  | FW18 | Sg4 | 63.5-66.4 | Marker39116, 38610, 39002, 39076 | 3.40 | 8.90 |
| FW18 | Hg4 | 44.2 | Marker42419 | 3.35 | 8.80 |  | FW18 | Sg4 | 68.6-73.7 | Marker38005, 37945, 38706, 38856, 38847 | 3.71 | 9.70 |
| FW18 | Hg4 | 70.1 | Marker39007 | 3.21 | 8.40 |  |  |  |  |  |  |  |
| FW18 | Hg4 | 75.4 | Marker38162 | 3.59 | 9.40 |  |  |  |  |  |  |  |
| FW18 | Hg7 | 83.8 | Marker99527 | 3.12 | 8.20 |  | FW18 | Sg7 | 66.4 | Marker102797 | 3.28 | 8.60 |
|  |  |  |  |  |  |  |  |  |  |  |  |  |
| FW19 | Hg1 | 3.2 | Marker61119 | 3.27 | 9.40 |  | FW19 | Sg3 | 29 | Marker62327 | 3.01 | 8.60 |
| FW19 | Hg1 | 12.1 | Marker35668 | 3.28 | 9.40 |  | FW19 | Sg3 | 57.5 | Marker58388 | 3.04 | 8.70 |
| FW19 | Hg1 | 13.8 | Marker35655 | 3.23 | 9.30 |  | FW19 | Sg3 | 59.1-61.7 | Marker56644, 57739, 72717, 116994 | 3.24 | 9.30 |
| FW19 | Hg1 | 117.3-117.8 | Marker33669, 34918 | 3.16 | 9.10 |  | FW19 | Sg4 | 41.4 | Marker40181 | 3.26 | 9.40 |
| FW19 | Hg1 | 62.9 | Marker25196 | 3.89 | 11.10 |  |  |  |  |  |  |  |
| FW19 | Hg5 | 66.9 | Marker93339 | 3.28 | 9.40 |  |  |  |  |  |  |  |
| FH18 | Hg1 | 45.8-50.5 | Marker21929 | 3.84 | 10.00 |  | FH18 | Sg1 | 160.9 | Marker19556 | 3.03 | 8.00 |
| FH18 | Hg3 | 34.3 | Marker58893 | 3.16 | 8.30 |  | FH18 | Sg1 | 140.2-143.4 | Marker21702 | 3.81 | 9.90 |
| FH18 | Hg4 | 35.6-42.5 | Marker42782 | 3.76 | 9.80 |  | FH18 | Sg4 | 42.2-49.7 | Marker119270 | 4.30 | 11.10 |
| FH18 | Hg4 | 54.2-57.2 | Marker40697 | 4.20 | 10.90 |  | FH18 | Sg4 | 54.8-76.8 | Marker39268 | 5.47 | 13.90 |
| FH18 | Hg4 | 60.4-67.9 | Marker39275 | 4.36 | 11.30 |  | FH18 | Sg5 | 41.1-47.1 | Marker93013 | 3.07 | 8.10 |
| FH18 | Hg4 | 70.1-76.7 | Marker38162 | 5.36 | 13.70 |  | FH18 | Sg7 | 66.4 | Marker102797 | 3.96 | 10.30 |
| FH19 | Hg1 | 19.4 | Marker33712 | 3.21 | 9.20 |  | FH19 | Sg5 | 88.3 | Marker57966, 92443 | 3.89 | 11.00 |
| FH19 | Hg1 | 115.8-117.8 | Marker34918, 34090, 33669, 34145, 33411 | 3.68 | 10.50 |  |  |  |  |  |  |  |
| FH19 | Hg1 | 62.9 | Marker25196 | 3.09 | 8.90 |  |  |  |  |  |  |  |
| FH19 | Hg2 | 19.3 | Marker68245 | 3.08 | 8.90 |  |  |  |  |  |  |  |
| FH19 | Hg5 | 71.1 | Marker92341 | 3.40 | 9.70 |  |  |  |  |  |  |  |
| FH19 | Hg5 | 74.2 | Marker113111 | 3.15 | 9.00 |  |  |  |  |  |  |  |
| FV18 | Hg3 | 6.9-7.9 | Marker62787, 62528, 63202 | 4.13 | 10.70 |  | FV18 | Sg3 | 16 | Marker63149 | 3.50 | 9.20 |
| FV18 | Hg3 | 9.9 | Marker61339 | 3.53 | 9.20 |  | FV18 | Sg3 | 21.2 | Marker62942 | 3.88 | 10.10 |
| FV18 | Hg3 | 21.2 | Marker14305 | 3.74 | 9.70 |  | FV18 | Sg3 | 39.7 | Marker62366 | 4.17 | 10.80 |
| FV18 | Hg3 | 25.7-27.3 | Marker60180,60115, 60082, 59874 | 3.59 | 9.40 |  |  |  |  |  |  |  |
| FV18 | Hg3 | 30.5-31.4 | Marker59544, 59427, 59314 | 3.52 | 9.20 |  |  |  |  |  |  |  |
| FV18 | Hg3 | 34.3 | Marker58893 | 4.13 | 10.70 |  |  |  |  |  |  |  |
| FV19 | Hg1 | 62.9 | Marker25196 | 3.81 | 10.80 |  | FV19 | Sg3 | 55.8-63.8 | Marker57207 | 4.92 | 13.80 |
| FV19 | Hg3 | 7.5 | Marker62528 | 3.83 | 10.90 |  | FV19 | Sg3 | 54.2 | Marker57693 | 4.12 | 11.70 |
|  |  |  |  |  |  |  | FV19 | Sg3 | 39-39.5 | Marker62327, 62606 | 3.98 | 11.30 |
| FL18 | Hg1 | 63.6 | Marker28434 | 3.23 | 8.50 |  | FL18 | Sg4 | 29.2 | Marker37945 | 3.33 | 8.70 |
| FL18 | Hg4 | 42.5 | Marker42494 | 3.82 | 9.90 |  | FL18 | Sg6 | 59.5 | Marker3824 | 3.52 | 9.20 |
| FL18 | Hg4 | 75.4 | Marker38162 | 3.54 | 9.30 |  |  |  |  |  |  |  |
| FL18 | Hg6 | 30.9 | Marker3924 | 3.03 | 8.00 |  |  |  |  |  |  |  |
| FL19 | Hg1 | 129.0-130.6 | Marker35310, 35730, 35261, 35668 | 3.18 | 9.10 |  | FL19 | Sg3 | 57.5-58.1 | Marker58388, 57739 | 3.73 | 10.60 |
| FL19 | Hg1 | 62.9 | Marker25196 | 3.54 | 10.10 |  | FL19 | Sg3 | 61.1-62.7 | Marker113721, 56644, 72717 | 3.21 | 9.20 |
| FL19 | Hg4 | 30.4 | Marker43420 | 3.02 | 8.70 |  | FL19 | Sg4 | 43.5 | Marker40475 | 3.22 | 9.20 |
| FL19 | Hg5 | 33.0-33.2 | Marker92814,92341 | 3.33 | 9.50 |  | FL19 | Sg5 | 38.3 | Marker57966 | 3.06 | 8.80 |
| SSC18 | Hg2 | 10.2-10.6 | Marker121293, 64728 | 3.31 | 8.60 |  | SSC18 | Sg2 | 27.1 | Marker65691 | 2.65 | 7.00 |
| SSC18 | Hg2 | 13.8 | Marker66701 | 2.63 | 6.90 |  | SSC18 | Sg2 | 50.6 | Marker69023 | 2.50 | 6.60 |
| SSC18 | Hg2 | 32.7 | Marker70573 | 2.69 | 7.10 |  | SSC18 | Sg3 | 55 | Marker60915 | 2.55 | 6.70 |
|  |  |  |  |  |  |  | SSC18 | Sg3 | 87.8 | Marker53093 | 2.60 | 6.80 |
| SSC19 | Hg1 | 82.9 | Marker22446 | 2.51 | 7.30 |  | SSC19 | Sg2 | 35.4 | Marker89057 | 2.55 | 7.40 |
| SSC19 | Hg2 | 10.2 | Marker121293 | 2.55 | 7.40 |  | SSC19 | Sg3 | 11.6 | Marker62951 | 2.64 | 7.60 |
| SSC19 | Hg4 | 39.1-39.4 | Marker42337, 42196 | 2.91 | 8.40 |  | SSC19 | Sg4 | 10.6 | Marker44036 | 2.76 | 8.00 |
| SSC19 | Hg4 | 65.2-65.6 | Marker39997, 40087, 40108 | 2.68 | 7.80 |  | SSC19 | Sg4 | 32.9-33.7 | Marker41859, 42337 | 3.35 | 9.60 |
|  |  |  |  |  |  |  | SSC19 | Sg6 | 17.1 | Marker686 | 2.70 | 7.80 |
| FF18 | Hg1 | 35.2-35.8 | Marker19896, 18459, 18515 | 3.36 | 8.70 |  | FF18 | Sg1 | 155.3 | Marker20278 | 3.10 | 8.10 |
| FF18 | Hg2 | 0-1.7 | Marker8120, 64041 | 4.33 | 11.10 |  | FF18 | Sg3 | 60.4-64.8 | Marker57055， 115632， 57207， 113721， 56644， 72717， 116994 | 3.67 | 9.50 |
| FF18 | Hg2 | 91.2-92.5 | Marker76722, 76436 | 3.51 | 9.10 |  | FF18 | Sg3 | 68.8 | Marker55253 | 3.07 | 8.00 |
| FF18 | Hg7 | 42.4-43.5 | Marker104096,104885 | 3.10 | 8.10 |  | FF18 | Sg4 | 81.9 | Marker37259 | 3.36 | 8.80 |
| FF18 | Hg7 | 37.5-38.1 | Marker103591,103946, 103498 | 3.36 | 8.70 |  |  |  |  |  |  |  |
| FF18 | Hg7 | 62 | Marker109053 | 3.44 | 9.00 |  |  |  |  |  |  |  |
| FF19 | Hg1 | 98.2-98.8 | Marker31254 | 2.94 | 8.50 |  | FF19 | Sg1 | 62.1 | Marker30222 | 2.57 | 7.40 |
| FF19 | Hg1 | 53.7 | Marker28573 | 2.57 | 7.40 |  | FF19 | Sg5 | 49.2-51.3 | Marker93209, 93551, 93437 | 2.92 | 8.40 |
| FF19 | Hg1 | 12.2-13.9 | Marker15306, 15435, 15130, 14962, 15102 | 4.13 | 11.70 |  | FF19 | Sg5 | 43.7 | Marker93623 | 2.92 | 8.40 |
| FF19 | Hg5 | 63.6 | Marker93443 | 3.25 | 9.30 |  |  |  |  |  |  |  |
| FF19 | Hg5 | 66.9 | Marker93339 | 2.80 | 8.10 |  |  |  |  |  |  |  |
