# Supplementary material for: Construction of a High-Density Genetic Map and Identification of Quantitative Trait Loci Linked to Fruit Quality Traits in Apricots Using Specific-Locus Amplified Fragment Sequencing
Source: Front Plant Sci. 2022 Feb 14;13:798700. doi: 10.3389/fpls.2022.798700 (PMC8882730; doi:10.3389/fpls.2022.798700)
Supplement: Supplementary file 7 [file Table_7.docx]

**Supplementary Table 7.** **Candidate genes related to fruit quality annotated in apricot reference genome**

| Traits | QTL Loci | Linkage groups | Markers | Chr. | Start (bp) | End （bp） | Gene ID | GO_annotation | KEGG_annotation |
| --- | --- | --- | --- | --- | --- | --- | --- | --- | --- |
| FW | **H_FW19** | **Hg1** | **Marker25196** | LG2 | 21,354,956 | 21,355,056 | PARG06010 | poly(A)+ mRNA export from nucleus | nucleoporin GLE1 |
| FW | **H_FH19** | **Hg1** | **Marker25196** |  |  |  | PARG06013 | oxidoreductase activity, acting on paired donors, with incorporation or reduction of molecular oxygen | #N/A |
| FW | **H_FV19** | **Hg1** | **Marker25196** |  |  |  | PARG06015 | oxidoreductase activity, acting on paired donors, with incorporation or reduction of molecular oxygen | #N/A |
| FW |  |  |  |  |  |  | PARG06016 | protein kinase activity | dual-specificity kinase [EC:2.7.12.1] |
| FW |  |  |  |  |  |  | PARG06017 | protein binding | #N/A |
| FW |  |  |  |  |  |  | PARG06018 | protein tyrosine phosphatase activity | #N/A |
| FW |  |  |  |  |  |  | PARG06020 | pyridoxal phosphate binding | glutamate--glyoxylate aminotransferase [EC:2.6.1.4 2.6.1.2 2.6.1.44] |
| FW |  |  |  |  |  |  | PARG06021 | regulation of DNA endoreduplication | #N/A |
| FW |  |  |  |  |  |  | PARG06022 | protein binding | #N/A |
| FW |  |  |  |  |  |  | PARG06023 | zinc ion binding | #N/A |
| FW |  |  |  |  |  |  | PARG06025 | catechol oxidase activity | #N/A |
| FW |  |  |  |  |  |  | PARG06026 | catechol oxidase activity | #N/A |
| FH | **H_FH19** | **Hg1** | **Marker33669** | LG2 | 4,039,325 | 4,039,425 | PARG03876 | protein binding | #N/A |
| FH | H_FW19 | Hg1 | Marker33669 |  |  |  | PARG03878 | protein binding | #N/A |
| FH |  |  |  |  |  |  | PARG03881 | DNA-binding transcription factor activity | #N/A |
| FH |  |  |  |  |  |  | PARG03884 | cell redox homeostasis | transcription factor TCP21 (protein CCA1 HIKING EXPEDITION) |
| FH |  |  |  |  |  |  | PARG03885 | transmembrane transporter activity | homeobox-leucine zipper protein |
| FH |  |  |  |  |  |  | PARG03889 | sequence-specific DNA binding | #N/A |
| FH |  |  |  |  |  |  | PARG03892 | protein disulfide oxidoreductase activity | #N/A |
| FH |  |  |  |  |  |  | PARG03893 | catalytic activity | ethanolamine-phosphate cytidylyltransferase [EC:2.7.7.14] |
| FH |  |  |  |  |  |  | PARG03895 | hydrolase activity, hydrolyzing O-glycosyl compounds | isoamylase [EC:3.2.1.68] |
| FH | **H_FH19** | **Hg1** | **Marker34090** | LG2 | 5,219,574 | 5,974,024 | PARG04016 | structural constituent of ribosome | large subunit ribosomal protein L4e |
| FH | **H_FH19** | **Hg1** | **Marker34145** |  |  |  | PARG04023 | protein binding | leucine-rich PPR motif-containing protein, mitochondrial |
| FH | **H_FH19** | **Hg1** | **Marker33411** |  |  |  | PARG04026 | serine-type endopeptidase activity | #N/A |
| FH |  |  |  |  |  |  | PARG04028 | Elongator holoenzyme complex | elongator complex protein 4 |
| FH |  |  |  |  |  |  | PARG04030 | chitin binding | #N/A |
| FH |  |  |  |  |  |  | PARG04031 | peptidyl-diphthamide biosynthetic process from peptidyl-histidine | diphthamide biosynthesis protein 2 |
| FH |  |  |  |  |  |  | PARG04033 | transferase activity, transferring acyl groups other than amino-acyl groups | shikimate O-hydroxycinnamoyltransferase [EC:2.3.1.133] |
| FH |  |  |  |  |  |  | PARG04034 | methyltransferase activity | 2-methoxy-6-polyprenyl-1,4-benzoquinol methylase [EC:2.1.1.201] |
| FH |  |  |  |  |  |  | PARG04035 | structural constituent of ribosome | large subunit ribosomal protein LP1 |
| FH |  |  |  |  |  |  | PARG04036 | RNA binding | #N/A |
| FH |  |  |  |  |  |  | PARG04038 | cellular response to nitrate | #N/A |
| FH |  |  |  |  |  |  | PARG04040 | cellular response to nitrate | #N/A |
| FH |  |  |  |  |  |  | PARG04041 | protein binding | #N/A |
| FH |  |  |  |  |  |  | PARG04042 | carboxy-lyase activity | #N/A |
| FH |  |  |  |  |  |  | PARG04043 | ATPase-coupled cation transmembrane transporter activity | Zn2+/Cd2+-exporting ATPase [EC:7.2.2.12 7.2.2.21] |
| FH |  |  |  |  |  |  | PARG04047 | protein binding | #N/A |
| FH |  |  |  |  |  |  | PARG04049 | protein binding | #N/A |
| FH |  |  |  |  |  |  | PARG04053 | regulation of transcription, DNA-templated | #N/A |
| FH |  |  |  |  |  |  | PARG04059 | protein binding | #N/A |
| FH |  |  |  |  |  |  | PARG04060 | unfolded protein binding | heat shock 70kDa protein 1/2/6/8 |
| FH |  |  |  |  |  |  | PARG04061 | acetylglucosaminyltransferase activity | #N/A |
| FH |  |  |  |  |  |  | PARG04062 | aspartic-type endopeptidase activity | #N/A |
| FH |  |  |  |  |  |  | PARG04063 | protein binding | splicing factor 3A subunit 1 |
| FH |  |  |  |  |  |  | PARG04066 | protein binding | #N/A |
| FH |  |  |  |  |  |  | PARG04067 | zinc ion binding | #N/A |
| FH |  |  |  |  |  |  | PARG04068 | cysteine-type peptidase activity | #N/A |
| FH |  |  |  |  |  |  | PARG04069 | peroxidase activity | peroxidase [EC:1.11.1.7] |
| FH |  |  |  |  |  |  | PARG04071 | iron-sulfur cluster assembly | #N/A |
| FH |  |  |  |  |  |  | PARG04075 | proton-exporting ATPase activity, phosphorylative mechanism | H+-transporting ATPase [EC:7.1.2.1] |
| FH |  |  |  |  |  |  | PARG04076 | helicase activity | ATP-dependent RNA helicase DHX8/PRP22 [EC:3.6.4.13] |
| FH |  |  |  |  |  |  | PARG04077 | lysine-tRNA ligase activity | lysyl-tRNA synthetase, class II [EC:6.1.1.6] |
| FH |  |  |  |  |  |  | PARG04078 | protein binding | clock-associated PAS protein ZTL |
| FH |  |  |  |  |  |  | PARG04081 | ATP binding | #N/A |
| FH |  |  |  |  |  |  | PARG04082 | protein kinase activity | interleukin-1 receptor-associated kinase 4 [EC:2.7.11.1] |
| FH |  |  |  |  |  |  | PARG04083 | motor activity | #N/A |
| FH |  |  |  |  |  |  | PARG04086 | aminoacyl-tRNA ligase activity | lysyl-tRNA synthetase, class II [EC:6.1.1.6] |
| FH |  |  |  |  |  |  | PARG04088 | protein binding | #N/A |
| FH |  |  |  |  |  |  | PARG04089 | aminoacyl-tRNA ligase activity | lysyl-tRNA synthetase, class II [EC:6.1.1.6] |
| FH |  |  |  |  |  |  | PARG04091 | protein binding | #N/A |
| FH |  |  |  |  |  |  | PARG04092 | protein binding | #N/A |
| FH |  |  |  |  |  |  | PARG04093 | protein binding | #N/A |
| FH |  |  |  |  |  |  | PARG04094 | cysteine-type peptidase activity | #N/A |
| FH |  |  |  |  |  |  | PARG04098 | protein kinase activity | #N/A |
| FH |  |  |  |  |  |  | PARG04099 | RNA splicing | U4/U6.U5 tri-snRNP-associated protein 3 |
| FH |  |  |  |  |  |  | PARG04101 | protein binding | #N/A |
| FH |  |  |  |  |  |  | PARG04102 | protein binding | #N/A |
| FH | **H_FH18** | **Hg4** | **Marker40697** | LG3 | 17,018,832 | 18,371,259 | PARG11341 | DNA-3-methyladenine glycosylase activity | DNA-3-methyladenine glycosylase I [EC:3.2.2.20] |
| FH | **S_FH18** | **Sg2** | **Marker119270** |  |  |  | PARG11342 | structural constituent of ribosome | small subunit ribosomal protein S4e |
| FH | S_FW18 | Sg4 | Marker40475 |  |  |  | PARG11344 | photosystem I assembly | #N/A |
| FH | S_FW18 | Sg4 | Marker40397 |  |  |  | PARG11345 | purine nucleoside transmembrane transporter activity | #N/A |
| FH | S_FW19 | Sg4 | Marker40181 |  |  |  | PARG11346 | DNA helicase activity | DNA replication licensing factor MCM6 [EC:3.6.4.12] |
| FH |  |  |  |  |  |  | PARG11347 | anchored component of plasma membrane | #N/A |
| FH |  |  |  |  |  |  | PARG11348 | regulation of transcription, DNA-templated | #N/A |
| FH |  |  |  |  |  |  | PARG11349 | methyltransferase activity | release factor glutamine methyltransferase [EC:2.1.1.297] |
| FH |  |  |  |  |  |  | PARG11350 | helicase activity | pre-mRNA-splicing factor ATP-dependent RNA helicase DHX38/PRP16 [EC:3.6.4.13] |
| FH |  |  |  |  |  |  | PARG11351 | protein kinase activity | #N/A |
| FH |  |  |  |  |  |  | PARG11352 | protein heterodimerization activity | transcription initiation factor TFIID subunit 11 |
| FH |  |  |  |  |  |  | PARG11353 | protein binding | #N/A |
| FH |  |  |  |  |  |  | PARG11354 | protein binding | #N/A |
| FH |  |  |  |  |  |  | PARG11355 | protein binding | #N/A |
| FH |  |  |  |  |  |  | PARG11356 | protein binding | #N/A |
| FH |  |  |  |  |  |  | PARG11358 | protein binding | #N/A |
| FH |  |  |  |  |  |  | PARG11410 | DNA binding | #N/A |
| FH |  |  |  |  |  |  | PARG11411 | oxidoreductase activity | succinate-semialdehyde dehydrogenase, mitochondrial [EC:1.2.1.24] |
| FH |  |  |  |  |  |  | PARG11412 | membrane | #N/A |
| FH |  |  |  |  |  |  | PARG11413 | methyltransferase activity | #N/A |
| FH |  |  |  |  |  |  | PARG11414 | terpene synthase activity | ent-kaurene synthase [EC:4.2.3.19] |
| FH |  |  |  |  |  |  | PARG11415 | terpene synthase activity | #N/A |
| FH |  |  |  |  |  |  | PARG11416 | protein kinase activity | #N/A |
| FH |  |  |  |  |  |  | PARG11417 | protein kinase activity | #N/A |
| FH |  |  |  |  |  |  | PARG11422 | protein kinase activity | #N/A |
| FH |  |  |  |  |  |  | PARG11423 | IMP dehydrogenase activity | IMP dehydrogenase [EC:1.1.1.205] |
| FH |  |  |  |  |  |  | PARG11424 | 3-deoxy-8-phosphooctulonate synthase activity | 2-dehydro-3-deoxyphosphooctonate aldolase (KDO 8-P synthase) [EC:2.5.1.55] |
| FH |  |  |  |  |  |  | PARG11425 | protein binding | #N/A |
| FH |  |  |  |  |  |  | PARG11426 | structural constituent of ribosome | small subunit ribosomal protein S9 |
| FH |  |  |  |  |  |  | PARG11427 | metal ion transmembrane transporter activity | #N/A |
| FH |  |  |  |  |  |  | PARG11428 | metal ion transmembrane transporter activity | #N/A |
| FH |  |  |  |  |  |  | PARG11429 | GTPase activity | ribosome assembly protein 1 [EC:3.6.5.-] |
| FH |  |  |  |  |  |  | PARG11430 | protein binding | #N/A |
| FH |  |  |  |  |  |  | PARG11432 | ubiquitin-protein transferase activity | #N/A |
| FH |  |  |  |  |  |  | PARG11439 | cysteine-type peptidase activity | #N/A |
| FH |  |  |  |  |  |  | PARG11441 | RNA binding | #N/A |
| FH |  |  |  |  |  |  | PARG11442 | polygalacturonase activity | #N/A |
| FH |  |  |  |  |  |  | PARG11443 | hydrolase activity | allantoate deiminase [EC:3.5.3.9] |
| FH |  |  |  |  |  |  | PARG11444 | protein binding | #N/A |
| FH |  |  |  |  |  |  | PARG11445 | protein binding | #N/A |
| FH |  |  |  |  |  |  | PARG11446 | protein binding | #N/A |
| FH |  |  |  |  |  |  | PARG11447 | zinc ion transmembrane transporter activity | #N/A |
| FH |  |  |  |  |  |  | PARG11448 | zinc ion transmembrane transporter activity | #N/A |
| FH |  |  |  |  |  |  | PARG11449 | GTP binding | GTPase |
| FH |  |  |  |  |  |  | PARG11450 | protein binding | #N/A |
| FH |  |  |  |  |  |  | PARG11451 | protein binding | #N/A |
| FH |  |  |  |  |  |  | PARG11452 | damaged DNA binding | DNA polymerase eta [EC:2.7.7.7] |
| FH |  |  |  |  |  |  | PARG11453 | threonine-type endopeptidase activity | 20S proteasome subunit alpha 3 [EC:3.4.25.1] |
| FH |  |  |  |  |  |  | PARG11454 | hydrolase activity | #N/A |
| FH |  |  |  |  |  |  | PARG11456 | phosphatidate cytidylyltransferase activity | mitochondrial translocator assembly and maintenance protein 41 |
| FH |  |  |  |  |  |  | PARG11457 | protein binding | #N/A |
| FH |  |  |  |  |  |  | PARG11458 | transmembrane transporter activity | #N/A |
| FH |  |  |  |  |  |  | PARG11460 | zinc ion binding | #N/A |
| FH |  |  |  |  |  |  | PARG11464 | response to auxin | #N/A |
| FH |  |  |  |  |  |  | PARG11465 | response to auxin | #N/A |
| FH |  |  |  |  |  |  | PARG11466 | ATPase-coupled intramembrane lipid transporter activity | #N/A |
| FH |  |  |  |  |  |  | PARG11470 | protein binding | #N/A |
| FH |  |  |  |  |  |  | PARG11471 | protein binding | #N/A |
| FH |  |  |  |  |  |  | PARG11472 | protein binding | #N/A |
| FH |  |  |  |  |  |  | PARG11473 | hydrolase activity | allantoate deiminase [EC:3.5.3.9] |
| FH |  |  |  |  |  |  | PARG11474 | polygalacturonase activity | #N/A |
| FH |  |  |  |  |  |  | PARG11476 | polygalacturonase activity | #N/A |
| FH |  |  |  |  |  |  | PARG11478 | catalytic activity | choline-phosphate cytidylyltransferase [EC:2.7.7.15] |
| FH |  |  |  |  |  |  | PARG11481 | copper ion binding | P-type Cu+ transporter [EC:7.2.2.8] |
| FH |  |  |  |  |  |  | PARG11482 | cell redox homeostasis | #N/A |
| FH |  |  |  |  |  |  | PARG11487 | zinc ion binding | #N/A |
| FH |  |  |  |  |  |  | PARG11488 | aminoacyl-tRNA ligase activity | phenylalanyl-tRNA synthetase alpha chain [EC:6.1.1.20] |
| FH |  |  |  |  |  |  | PARG11489 | uridylyltransferase activity | UDP-N-acetylglucosamine/UDP-N-acetylgalactosamine diphosphorylase [EC:2.7.7.23 2.7.7.83] |
| FH |  |  |  |  |  |  | PARG11490 | nickel cation binding | #N/A |
| FH |  |  |  |  |  |  | PARG11491 | structural constituent of ribosome | large subunit ribosomal protein L7/L12 |
| FH |  |  |  |  |  |  | PARG11492 | ATP binding | #N/A |
| FH |  |  |  |  |  |  | PARG11493 | protein kinase activity | #N/A |
| FH |  |  |  |  |  |  | PARG11494 | RNA binding | #N/A |
| FH |  |  |  |  |  |  | PARG11495 | potassium ion transmembrane transporter activity | #N/A |
| FH |  |  |  |  |  |  | PARG11496 | potassium ion transmembrane transporter activity | #N/A |
| FH |  |  |  |  |  |  | PARG11501 | potassium ion transmembrane transporter activity | #N/A |
| FH |  |  |  |  |  |  | PARG11513 | GTP binding | protein SEY1 [EC:3.6.5.-] |
| FH |  |  |  |  |  |  | PARG11514 | DNA-binding transcription factor activity | #N/A |
| FH |  |  |  |  |  |  | PARG11515 | transferase activity, transferring glycosyl groups | #N/A |
| FH |  |  |  |  |  |  | PARG11516 | catalytic activity | #N/A |
| FH |  |  |  |  |  |  | PARG11517 | sulfiredoxin activity | sulfiredoxin [EC:1.8.98.2] |
| FH |  |  |  |  |  |  | PARG11519 | ATP binding | mitochondrial chaperone BCS1 |
| FH |  |  |  |  |  |  | PARG11521 | ATP binding | #N/A |
| FH |  |  |  |  |  |  | PARG11523 | protein binding | #N/A |
| FH |  |  |  |  |  |  | PARG11527 | protein binding | #N/A |
| FH |  |  |  |  |  |  | PARG11528 | glycine cleavage complex | #N/A |
| FH |  |  |  |  |  |  | PARG11529 | protein binding | #N/A |
| FH |  |  |  |  |  |  | PARG11530 | proteasome core complex | 20S proteasome subunit alpha 2 [EC:3.4.25.1] |
| FH |  |  |  |  |  |  | PARG11531 | proteasome core complex | 20S proteasome subunit alpha 5 [EC:3.4.25.1] |
| FH |  |  |  |  |  |  | PARG11532 | peroxisome | peroxin-1 |
| FH |  |  |  |  |  |  | PARG11534 | zinc ion binding | #N/A |
| FH |  |  |  |  |  |  | PARG11535 | transcription factor TFIIH core complex | #N/A |
| FH |  |  |  |  |  |  | PARG11536 | protein binding | #N/A |
| FH | **H_FH18** | **Hg4** | **Marker38162** | LG3 | 22,253,028 | 22,818,330 | PARG12128 | transferase activity, transferring acyl groups other than amino-acyl groups | #N/A |
| FH | S_FW18 | Sg4 | Marker38005 |  |  |  | PARG12129 | magnesium chelatase activity | magnesium chelatase subunit I [EC:6.6.1.1] |
| FH | S_FW18 | Sg4 | Marker37945 |  |  |  | PARG12130 | hydrolase activity | peroxisomal coenzyme A diphosphatase NUDT7 [EC:3.6.1.-] |
| FH |  |  |  |  |  |  | PARG12131 | hydrolase activity | peroxisomal coenzyme A diphosphatase NUDT7 [EC:3.6.1.-] |
| FH |  |  |  |  |  |  | PARG12132 | DNA-binding transcription factor activity | #N/A |
| FH |  |  |  |  |  |  | PARG12134 | protein binding | #N/A |
| FH |  |  |  |  |  |  | PARG12135 | calcium ion binding | #N/A |
| FH |  |  |  |  |  |  | PARG12136 | helicase activity | ATP-dependent RNA helicase DDX35 [EC:3.6.4.13] |
| FH |  |  |  |  |  |  | PARG12137 | ligand-gated ion channel activity | glutamate receptor, ionotropic, plant |
| FH |  |  |  |  |  |  | PARG12139 | protein-containing complex assembly | #N/A |
| FH |  |  |  |  |  |  | PARG12140 | ligand-gated ion channel activity | glutamate receptor, ionotropic, plant |
| FH |  |  |  |  |  |  | PARG12141 | peroxidase activity | peroxidase [EC:1.11.1.7] |
| FH |  |  |  |  |  |  | PARG12142 | serine-tRNA ligase activity | #N/A |
| FH |  |  |  |  |  |  | PARG12143 | aminoacyl-tRNA editing activity | D-aminoacyl-tRNA deacylase [EC:3.1.1.96] |
| FH |  |  |  |  |  |  | PARG12144 | negative regulation of transcription, DNA-templated | #N/A |
| FH |  |  |  |  |  |  | PARG12145 | hydrolase activity, acting on ester bonds | #N/A |
| FH |  |  |  |  |  |  | PARG12146 | response to hormone | #N/A |
| FH |  |  |  |  |  |  | PARG12147 | DNA-binding transcription factor activity | pathogenesis-related genes transcriptional activator PTI5 |
| FH |  |  |  |  |  |  | PARG12148 | DNA binding | #N/A |
| FH |  |  |  |  |  |  | PARG12149 | calcium ion binding | #N/A |
| FH |  |  |  |  |  |  | PARG12151 | DNA binding | #N/A |
| FH |  |  |  |  |  |  | PARG12152 | GTPase activity | Rab family, other |
| FH |  |  |  |  |  |  | PARG12154 | transferase activity, transferring hexosyl groups | flavonol-3-O-glucoside/galactoside glucosyltransferase [EC:2.4.1.239 2.4.1.-] |
| FH |  |  |  |  |  |  | PARG12157 | regulation of transcription, DNA-templated | #N/A |
| FH |  |  |  |  |  |  | PARG12159 | protein heterodimerization activity | histone H3 |
| FH |  |  |  |  |  |  | PARG12160 | O-methyltransferase activity | acetylserotonin O-methyltransferase, plant [EC:2.1.1.4] |
| FH |  |  |  |  |  |  | PARG12163 | response to hormone | #N/A |
| FH |  |  |  |  |  |  | PARG12164 | integral component of membrane | mlo protein |
| FH |  |  |  |  |  |  | PARG12165 | protein binding | #N/A |
| FH |  |  |  |  |  |  | PARG12166 | protein disulfide oxidoreductase activity | #N/A |
| FH |  |  |  |  |  |  | PARG12167 | GTPase activity | #N/A |
| FH |  |  |  |  |  |  | PARG12168 | regulation of transcription, DNA-templated | #N/A |
| FH |  |  |  |  |  |  | PARG12169 | protein kinase activity | #N/A |
| FH |  |  |  |  |  |  | PARG12170 | protein kinase activity | #N/A |
| FH |  |  |  |  |  |  | PARG12173 | protein binding | #N/A |
| FH |  |  |  |  |  |  | PARG12174 | transmembrane transporter activity | #N/A |
| FH |  |  |  |  |  |  | PARG12175 | DNA binding | #N/A |
| FH |  |  |  |  |  |  | PARG12176 | unfolded protein binding | #N/A |
| FH |  |  |  |  |  |  | PARG12177 | glycogen phosphorylase activity | glycogen phosphorylase [EC:2.4.1.1] |
| FH |  |  |  |  |  |  | PARG12182 | zinc ion binding | #N/A |
| FH |  |  |  |  |  |  | PARG12184 | DNA-binding transcription factor activity | EREBP-like factor |
| FH |  |  |  |  |  |  | PARG12186 | protein binding | #N/A |
| FH |  |  |  |  |  |  | PARG12187 | metal ion binding | #N/A |
| FH |  |  |  |  |  |  | PARG12189 | serine-type endopeptidase activity | peroxisomal leader peptide-processing protease [EC:3.4.21.-] |
| FH |  |  |  |  |  |  | PARG12191 | regulation of transcription, DNA-templated | #N/A |
| FH |  |  |  |  |  |  | PARG12192 | regulation of transcription, DNA-templated | #N/A |
| FH |  |  |  |  |  |  | PARG12199 | DNA binding | #N/A |
| FH |  |  |  |  |  |  | PARG12200 | structural constituent of ribosome | small subunit ribosomal protein S5 |
| FH |  |  |  |  |  |  | PARG12201 | protein binding | #N/A |
| FH |  |  |  |  |  |  | PARG12202 | protein binding | #N/A |
| FH |  |  |  |  |  |  | PARG12206 | translation initiation factor activity | translation initiation factor 3 subunit D |
| FH |  |  |  |  |  |  | PARG12207 | protein dimerization activity | #N/A |
| FH |  |  |  |  |  |  | PARG12208 | protein dimerization activity | #N/A |
| FH |  |  |  |  |  |  | PARG12211 | protein dimerization activity | #N/A |
| FH |  |  |  |  |  |  | PARG12214 | oxidoreductase activity | lipoxygenase [EC:1.13.11.12] |
| FH |  |  |  |  |  |  | PARG12215 | oxidoreductase activity | vesicle-fusing ATPase [EC:3.6.4.6] |
| FH |  |  |  |  |  |  | PARG12216 | structural constituent of ribosome | large subunit ribosomal protein L21 |
| FH |  |  |  |  |  |  | PARG12218 | 1,3-beta-D-glucan synthase activity | callose synthase [EC:2.4.1.-] |
| FH |  |  |  |  |  |  | PARG12220 | zinc ion binding | #N/A |
| FH |  |  |  |  |  |  | PARG12221 | 3-deoxy-manno-octulosonate cytidylyltransferase activity | 3-deoxy-manno-octulosonate cytidylyltransferase (CMP-KDO synthetase) [EC:2.7.7.38] |
| FH |  |  |  |  |  |  | PARG12224 | DNA-directed 5'-3' RNA polymerase activity | DNA-directed RNA polymerase II subunit RPB2 [EC:2.7.7.6] |
| FH |  |  |  |  |  |  | PARG12228 | metal ion binding | E3 ubiquitin-protein ligase RNF5 [EC:2.3.2.27] |
| FH |  |  |  |  |  |  | PARG12229 | sequence-specific DNA binding | heat shock transcription factor, other eukaryote |
| FH |  |  |  |  |  |  | PARG12230 | protein binding | #N/A |
| FH |  |  |  |  |  |  | PARG12231 | protein disulfide oxidoreductase activity | #N/A |
| FH |  |  |  |  |  |  | PARG12232 | protein disulfide oxidoreductase activity | #N/A |
| FH |  |  |  |  |  |  | PARG12236 | mismatched DNA binding | #N/A |
| FH |  |  |  |  |  |  | PARG12237 | allantoinase activity | allantoinase [EC:3.5.2.5] |
| FH |  |  |  |  |  |  | PARG12239 | transcription factor TFIIH core complex | transcription initiation factor TFIIH subunit 4 |
| FH |  |  |  |  |  |  | PARG12240 | integral component of membrane | #N/A |
| FH |  |  |  |  |  |  | PARG12241 | metal ion binding | #N/A |
| FH |  |  |  |  |  |  | PARG12242 | protein binding | #N/A |
| FH |  |  |  |  |  |  | PARG12244 | nucleic acid binding | splicing factor U2AF 65 kDa subunit |
| FH |  |  |  |  |  |  | PARG12248 | protein disulfide oxidoreductase activity | #N/A |
| FH |  |  |  |  |  |  | PARG12250 | regulation of transcription, DNA-templated | #N/A |
| FH |  |  |  |  |  |  | PARG12251 | protein kinase activity | #N/A |
| FH | **S_FH19** | **Sg5** | **Marker92443** | LG7 | 6,800,739 | 7,773,006 | PARG23408 | mismatched DNA binding | #N/A |
| FH | **S_FH19** | **Sg5** | **Marker57966** |  |  |  | PARG23409 | oxidoreductase activity | #N/A |
| FH | H_FH19 | Hg5 | Marker113111 |  |  |  | PARG23410 | protein kinase activity | #N/A |
| FH | H_FL19 | Hg5 | Marker92341 |  |  |  | PARG23412 | protein glycosylation\|\|transferase activity, transferring glycosyl groups | #N/A |
| FH |  |  |  |  |  |  | PARG23413 | DNA binding | transcription factor MYB, plant |
| FH |  |  |  |  |  |  | PARG23414 | DNA-binding transcription factor activity | #N/A |
| FH |  |  |  |  |  |  | PARG23415 | structural constituent of ribosome | #N/A |
| FH |  |  |  |  |  |  | PARG23416 | acyl-CoA oxidase activity | acyl-CoA oxidase [EC:1.3.3.6] |
| FH |  |  |  |  |  |  | PARG23418 | DNA binding | #N/A |
| FH |  |  |  |  |  |  | PARG23420 | oxidoreductase activity | #N/A |
| FH |  |  |  |  |  |  | PARG23424 | DNA-binding transcription factor activity | homeobox-leucine zipper protein |
| FH |  |  |  |  |  |  | PARG23427 | protein binding | #N/A |
| FH |  |  |  |  |  |  | PARG23429 | protein tyrosine/serine/threonine phosphatase activity | #N/A |
| FH |  |  |  |  |  |  | PARG23430 | palmitoyl hydrolase activity | palmitoyl-protein thioesterase [EC:3.1.2.22] |
| FH |  |  |  |  |  |  | PARG23431 | ubiquitin protein ligase binding | cullin 1 |
| FH |  |  |  |  |  |  | PARG23432 | ubiquitin protein ligase binding | #N/A |
| FH |  |  |  |  |  |  | PARG23433 | ubiquitin protein ligase binding | #N/A |
| FH |  |  |  |  |  |  | PARG23434 | ubiquitin protein ligase binding | #N/A |
| FH |  |  |  |  |  |  | PARG23435 | ubiquitin protein ligase binding | cullin 1 |
| FH |  |  |  |  |  |  | PARG23436 | ubiquitin protein ligase binding | #N/A |
| FH |  |  |  |  |  |  | PARG23437 | ubiquitin protein ligase binding | #N/A |
| FH |  |  |  |  |  |  | PARG23439 | electron transfer activity | ferredoxin-2, mitochondrial |
| FH |  |  |  |  |  |  | PARG23443 | phospholipase A1 activity | #N/A |
| FH |  |  |  |  |  |  | PARG23444 | phospholipase A1 activity | #N/A |
| FH |  |  |  |  |  |  | PARG23445 | phospholipase A1 activity | #N/A |
| FH |  |  |  |  |  |  | PARG23447 | hydrolase activity | #N/A |
| FH |  |  |  |  |  |  | PARG23449 | proton-exporting ATPase activity, phosphorylative mechanism | H+-transporting ATPase [EC:7.1.2.1] |
| FH |  |  |  |  |  |  | PARG23452 | DNA-binding transcription factor activity | EREBP-like factor |
| FH |  |  |  |  |  |  | PARG23453 | DNA-binding transcription factor activity | EREBP-like factor |
| FH |  |  |  |  |  |  | PARG23455 | U4/U6 x U5 tri-snRNP complex | U4/U6 small nuclear ribonucleoprotein PRP31 |
| FH |  |  |  |  |  |  | PARG23456 | thiol-dependent ubiquitin-specific protease activity | ubiquitin carboxyl-terminal hydrolase L3 [EC:3.4.19.12] |
| FH |  |  |  |  |  |  | PARG23457 | DNA-binding transcription factor activity | RAV-like factor |
| FH |  |  |  |  |  |  | PARG23458 | catalytic activity | methylglutaconyl-CoA hydratase [EC:4.2.1.18] |
| FH |  |  |  |  |  |  | PARG23462 | oxidation-reduction process\|\|oxidoreductase activity | #N/A |
| FH |  |  |  |  |  |  | PARG23463 | lipid metabolic process | plasminogen activator inhibitor 1 RNA-binding protein |
| FH |  |  |  |  |  |  | PARG23464 | RNA binding | #N/A |
| FH |  |  |  |  |  |  | PARG23465 | zinc ion binding | #N/A |
| FH |  |  |  |  |  |  | PARG23466 | GTPase activity | Ras-related protein Rab-1A |
| FH |  |  |  |  |  |  | PARG23467 | negative regulation of transcription, DNA-templated\|\|transcription corepressor activity | #N/A |
| FH |  |  |  |  |  |  | PARG23470 | FAD binding | #N/A |
| FH |  |  |  |  |  |  | PARG23472 | oxidoreductase activity | #N/A |
| FH |  |  |  |  |  |  | PARG23474 | oxidoreductase activity | #N/A |
| FH |  |  |  |  |  |  | PARG23476 | transferase activity, transferring acyl groups other than amino-acyl groups | #N/A |
| FH |  |  |  |  |  |  | PARG23480 | transmembrane transporter activity | #N/A |
| FH |  |  |  |  |  |  | PARG23481 | Set1C/COMPASS complex | #N/A |
| FH |  |  |  |  |  |  | PARG23484 | structural constituent of ribosome | #N/A |
| FH |  |  |  |  |  |  | PARG23485 | protein kinase activity | cell division control protein 7 [EC:2.7.11.1] |
| FH |  |  |  |  |  |  | PARG23486 | integral component of membrane | #N/A |
| FH |  |  |  |  |  |  | PARG23488 | regulation of transcription, DNA-templated | #N/A |
| FH |  |  |  |  |  |  | PARG23489 | unfolded protein binding | T-complex protein 1 subunit alpha |
| FH |  |  |  |  |  |  | PARG23490 | CCR4-NOT complex | #N/A |
| FH |  |  |  |  |  |  | PARG23491 | serine-type endopeptidase activity | ATP-dependent Clp protease, protease subunit [EC:3.4.21.92] |
| FH |  |  |  |  |  |  | PARG23495 | protein binding | #N/A |
| FH |  |  |  |  |  |  | PARG23499 | protein binding | #N/A |
| FH |  |  |  |  |  |  | PARG23500 | integral component of membrane | #N/A |
| FH |  |  |  |  |  |  | PARG23501 | histone-lysine N-methyltransferase activity | [histone H3]-lysine9 N-trimethyltransferase EHMT [EC:2.1.1.355] |
| FH |  |  |  |  |  |  | PARG23502 | protein binding | #N/A |
| FH |  |  |  |  |  |  | PARG23503 | protein binding | #N/A |
| FH |  |  |  |  |  |  | PARG23506 | oxidoreductase activity, acting on the CH-NH2 group of donors, NAD or NADP as acceptor | glutamate dehydrogenase (NAD(P)+) [EC:1.4.1.3] |
| FH |  |  |  |  |  |  | PARG23513 | zinc ion binding | #N/A |
| FH |  |  |  |  |  |  | PARG23515 | phosphorelay signal transduction system | #N/A |
| FH |  |  |  |  |  |  | PARG23516 | methyltransferase activity | #N/A |
| FH |  |  |  |  |  |  | PARG23517 | zinc ion binding | #N/A |
| FH |  |  |  |  |  |  | PARG23518 | methyltransferase activity | 1,4-dihydroxy-2-naphthoyl-CoA hydrolase [EC:3.1.2.28] |
| FH |  |  |  |  |  |  | PARG23519 | protein binding | #N/A |
| FH |  |  |  |  |  |  | PARG23520 | GTP binding | #N/A |
| FH |  |  |  |  |  |  | PARG23524 | phosphoacetylglucosamine mutase activity | phosphoacetylglucosamine mutase [EC:5.4.2.3] |
| FH |  |  |  |  |  |  | PARG23525 | structural constituent of ribosome | small subunit ribosomal protein S21 |
| FH |  |  |  |  |  |  | PARG23526 | structural molecule activity | iron-sulfur cluster assembly 2 |
| FH |  |  |  |  |  |  | PARG23527 | oxidoreductase activity, acting on paired donors, with incorporation or reduction of molecular oxygen | PHYB activation tagged suppressor 1 [EC:1.14.-.-] |
| FH |  |  |  |  |  |  | PARG23529 | transcription factor TFIIH core complex | #N/A |
| FH |  |  |  |  |  |  | PARG23531 | transcription factor TFIIH core complex | #N/A |
| FH |  |  |  |  |  |  | PARG23532 | translation initiation factor binding | #N/A |
| FH |  |  |  |  |  |  | PARG23534 | DNA binding | #N/A |
| FH |  |  |  |  |  |  | PARG23535 | protein binding | #N/A |
| FH |  |  |  |  |  |  | PARG23536 | extracellular region | #N/A |
| FH |  |  |  |  |  |  | PARG23538 | hydrolase activity | #N/A |
| FH |  |  |  |  |  |  | PARG23539 | hydrolase activity | #N/A |
| FH |  |  |  |  |  |  | PARG23540 | tRNA processing | adenylate dimethylallyltransferase (cytokinin synthase) [EC:2.5.1.27 2.5.1.112] |
| FH |  |  |  |  |  |  | PARG23541 | GTPase activity | #N/A |
| FH |  |  |  |  |  |  | PARG23542 | transcription corepressor activity | #N/A |
| FH |  |  |  |  |  |  | PARG23543 | purine nucleoside transmembrane transporter activity | #N/A |
| FH |  |  |  |  |  |  | PARG23544 | oxidoreductase activity | #N/A |
| FH |  |  |  |  |  |  | PARG23545 | aspartic-type endopeptidase activity | presenilin 1 [EC:3.4.23.-] |
| FH |  |  |  |  |  |  | PARG23546 | GTPase activity | Ras-related protein Rab-6A |
| FH |  |  |  |  |  |  | PARG23548 | enzyme inhibitor activity | #N/A |
| FH |  |  |  |  |  |  | PARG23549 | nucleic acid binding | #N/A |
| FH |  |  |  |  |  |  | PARG23551 | protein binding | mRNA export factor |
| FH |  |  |  |  |  |  | PARG23552 | ATP binding | DNA repair and recombination protein RAD54 and RAD54-like protein [EC:3.6.4.-] |
| FH |  |  |  |  |  |  | PARG23553 | hydrolase activity | regulator of nonsense transcripts 1 [EC:3.6.4.-] |
| FH |  |  |  |  |  |  | PARG23556 | proton-transporting ATP synthase activity, rotational mechanism | #N/A |
| FH |  |  |  |  |  |  | PARG23557 | CTP synthase activity | #N/A |
| FH |  |  |  |  |  |  | PARG23558 | ATP-dependent peptidase activity | ATP-dependent Lon protease [EC:3.4.21.53] |
| FH |  |  |  |  |  |  | PARG23562 | cation transport\|\|tsolute:proton antiporter activity | #N/A |
| FH |  |  |  |  |  |  | PARG23564 | endoribonuclease activity | poly(U)-specific endoribonuclease [EC:3.1.-.-] |
| FH |  |  |  |  |  |  | PARG23565 | beta-amylase activity | beta-amylase [EC:3.2.1.2] |
| FH |  |  |  |  |  |  | PARG23568 | protein kinase activity | #N/A |
| FH |  |  |  |  |  |  | PARG23569 | protein kinase activity | #N/A |
| FH |  |  |  |  |  |  | PARG23570 | protein serine/threonine kinase activity | #N/A |
| FH |  |  |  |  |  |  | PARG23571 | protein serine/threonine kinase activity | #N/A |
| FH |  |  |  |  |  |  | PARG23572 | protein serine/threonine kinase activity | #N/A |
| FH |  |  |  |  |  |  | PARG23574 | protein binding | #N/A |
| FH |  |  |  |  |  |  | PARG23575 | protein binding | #N/A |
| FH |  |  |  |  |  |  | PARG23576 | protein kinase activity | #N/A |
| FH |  |  |  |  |  |  | PARG23577 | SNAP receptor activity | novel plant SNARE |
| FH |  |  |  |  |  |  | PARG23578 | ATP binding | #N/A |
| FV | **H_FV18** | **Hg3** | **Marker63202** | LG4 | 35,066 | 1,212,672 | PARG12670 | hydrolase activity, acting on ester bonds | TatD DNase family protein [EC:3.1.21.-] |
| FV | **S_FV18** | **Sg3** | **Marker62942** |  |  |  | PARG12674 | ATP binding | #N/A |
| FV | S_FV18 | Hg5 | Marker63149 |  |  |  | PARG12675 | translation release factor activity | peptide chain release factor subunit 1 |
| FV | H_FV18 | Hg6 | Marker61339 |  |  |  | PARG12677 | catalytic activity | acetyl-CoA synthetase [EC:6.2.1.1] |
| FV |  |  |  |  |  |  | PARG12678 | protein binding | #N/A |
| FV |  |  |  |  |  |  | PARG12679 | oxidoreductase activity, acting on the aldehyde or oxo group of donors, NAD or NADP as acceptor | glyceraldehyde-3-phosphate dehydrogenase (NADP+) (phosphorylating) [EC:1.2.1.13] |
| FV |  |  |  |  |  |  | PARG12681 | ATP binding | ATP-dependent RNA helicase DDX59 [EC:3.6.4.13] |
| FV |  |  |  |  |  |  | PARG12684 | magnesium ion transmembrane transporter activity | syntaxin 7 |
| FV |  |  |  |  |  |  | PARG12685 | nucleic acid binding | #N/A |
| FV |  |  |  |  |  |  | PARG12686 | potassium ion transmembrane transporter activity | diphosphoinositol-polyphosphate diphosphatase [EC:3.6.1.52] |
| FV |  |  |  |  |  |  | PARG12687 | zinc ion binding | #N/A |
| FV |  |  |  |  |  |  | PARG12688 | protein kinase activity | interleukin-1 receptor-associated kinase 4 [EC:2.7.11.1] |
| FV |  |  |  |  |  |  | PARG12690 | Ino80 complex | #N/A |
| FV |  |  |  |  |  |  | PARG12691 | N-acetyltransferase activity | N-alpha-acetyltransferase 30 [EC:2.3.1.256] |
| FV |  |  |  |  |  |  | PARG12692 | ubiquinol-cytochrome-c reductase activity | ubiquinol-cytochrome c reductase iron-sulfur subunit [EC:7.1.1.8] |
| FV |  |  |  |  |  |  | PARG12694 | histone-lysine N-methyltransferase activity | [histone H3]-lysine9 N-trimethyltransferase EHMT [EC:2.1.1.355] |
| FV |  |  |  |  |  |  | PARG12696 | alpha-mannosidase activity | alpha-mannosidase [EC:3.2.1.24] |
| FV |  |  |  |  |  |  | PARG12697 | alpha-mannosidase activity | alpha-mannosidase [EC:3.2.1.24] |
| FV |  |  |  |  |  |  | PARG12698 | metal ion binding | #N/A |
| FV |  |  |  |  |  |  | PARG12699 | sialyltransferase activity | #N/A |
| FV |  |  |  |  |  |  | PARG12700 | aspartate kinase activity | aspartate kinase [EC:2.7.2.4] |
| FV |  |  |  |  |  |  | PARG12701 | protein dimerization activity | #N/A |
| FV |  |  |  |  |  |  | PARG12704 | zinc ion binding | #N/A |
| FV |  |  |  |  |  |  | PARG12706 | proton transmembrane transporter activity | #N/A |
| FV |  |  |  |  |  |  | PARG12715 | transferase activity, transferring acyl groups other than amino-acyl groups | #N/A |
| FV |  |  |  |  |  |  | PARG12716 | nucleic acid binding | #N/A |
| FV |  |  |  |  |  |  | PARG12719 | regulation of transcription, DNA-templated | #N/A |
| FV |  |  |  |  |  |  | PARG12720 | phosphoric diester hydrolase activity | glycerophosphodiester phosphodiesterase [EC:3.1.4.46] |
| FV |  |  |  |  |  |  | PARG12722 | palmitoyltransferase activity | palmitoyltransferase ZDHHC9/14/18 [EC:2.3.1.225] |
| FV |  |  |  |  |  |  | PARG12724 | exocytosis | exocyst complex component 7 |
| FV |  |  |  |  |  |  | PARG12727 | protein binding | #N/A |
| FV |  |  |  |  |  |  | PARG12728 | protein disulfide oxidoreductase activity | #N/A |
| FV |  |  |  |  |  |  | PARG12730 | transferase activity, transferring acyl groups other than amino-acyl groups | omega-hydroxypalmitate O-feruloyl transferase [EC:2.3.1.188] |
| FV |  |  |  |  |  |  | PARG12731 | ribonucleoprotein complex | large subunit ribosomal protein L7Ae |
| FV |  |  |  |  |  |  | PARG12732 | protein binding | PERQ amino acid-rich with GYF domain-containing protein |
| FV |  |  |  |  |  |  | PARG12733 | solute:proton antiporter activity | #N/A |
| FV |  |  |  |  |  |  | PARG12734 | protein binding | #N/A |
| FV |  |  |  |  |  |  | PARG12735 | DNA-binding transcription factor activity | #N/A |
| FV |  |  |  |  |  |  | PARG12736 | transmembrane transport | #N/A |
| FV |  |  |  |  |  |  | PARG12737 | ATPase activity | #N/A |
| FV |  |  |  |  |  |  | PARG12738 | regulation of auxin polar transport | #N/A |
| FV |  |  |  |  |  |  | PARG12740 | DNA-binding transcription factor activity | #N/A |
| FV |  |  |  |  |  |  | PARG12771 | voltage-gated chloride channel activity | chloride channel 7 |
| FV |  |  |  |  |  |  | PARG12772 | RNA methyltransferase activity | #N/A |
| FV |  |  |  |  |  |  | PARG12773 | ATP binding | uridine kinase [EC:2.7.1.48] |
| FV |  |  |  |  |  |  | PARG12774 | hydrolase activity, acting on ester bonds | #N/A |
| FV |  |  |  |  |  |  | PARG12777 | racemase and epimerase activity, acting on carbohydrates and derivatives | ribulose-phosphate 3-epimerase [EC:5.1.3.1] |
| FV |  |  |  |  |  |  | PARG12778 | protein kinase activity | #N/A |
| FV |  |  |  |  |  |  | PARG12780 | calcium ion binding | #N/A |
| FV |  |  |  |  |  |  | PARG12781 | nucleobase-containing compound kinase activity | adenylate kinase [EC:2.7.4.3] |
| FV |  |  |  |  |  |  | PARG12783 | protein kinase activity | #N/A |
| FV |  |  |  |  |  |  | PARG12784 | oxygen-dependent protoporphyrinogen oxidase activity | protoporphyrinogen/coproporphyrinogen III oxidase [EC:1.3.3.4 1.3.3.15] |
| FV |  |  |  |  |  |  | PARG12785 | protein binding | #N/A |
| FV |  |  |  |  |  |  | PARG12786 | translation | #N/A |
| FV |  |  |  |  |  |  | PARG12787 | regulation of transcription, DNA-templated | mTERF domain-containing protein, mitochondrial |
| FV |  |  |  |  |  |  | PARG12788 | protein binding | #N/A |
| FV |  |  |  |  |  |  | PARG12790 | COP9 signalosome | #N/A |
| FV |  |  |  |  |  |  | PARG12792 | protein binding | #N/A |
| FV |  |  |  |  |  |  | PARG12793 | protein binding | #N/A |
| FV |  |  |  |  |  |  | PARG12795 | histone acetyltransferase activity | #N/A |
| FV |  |  |  |  |  |  | PARG12798 | electron transfer activity | #N/A |
| FV |  |  |  |  |  |  | PARG12800 | thymidylate kinase activity | dTMP kinase [EC:2.7.4.9] |
| FV |  |  |  |  |  |  | PARG12801 | protein binding | #N/A |
| FV |  |  |  |  |  |  | PARG12802 | integral component of membrane | #N/A |
| FV |  |  |  |  |  |  | PARG12805 | protein binding | #N/A |
| FV |  |  |  |  |  |  | PARG12806 | membrane | prohibitin 1 |
| FV |  |  |  |  |  |  | PARG12807 | oxidoreductase activity, acting on the aldehyde or oxo group of donors, NAD or NADP as acceptor | glyceraldehyde 3-phosphate dehydrogenase [EC:1.2.1.12] |
| FV |  |  |  |  |  |  | PARG12809 | protein binding | #N/A |
| FV |  |  |  |  |  |  | PARG12810 | glucose-6-phosphate dehydrogenase activity | glucose-6-phosphate 1-dehydrogenase [EC:1.1.1.49 1.1.1.363] |
| FV |  |  |  |  |  |  | PARG12811 | nucleic acid binding | DNA repair protein RAD5 [EC:3.6.4.-] |
| FV |  |  |  |  |  |  | PARG12812 | membrane | #N/A |
| FV |  |  |  |  |  |  | PARG12813 | ATP binding | #N/A |
| FV |  |  |  |  |  |  | PARG12814 | membrane | #N/A |
| FV |  |  |  |  |  |  | PARG12815 | membrane | #N/A |
| FV |  |  |  |  |  |  | PARG12816 | protein phosphatase regulator activity | serine/threonine-protein phosphatase 2A regulatory subunit B' |
| FV |  |  |  |  |  |  | PARG12817 | hydrolase activity | #N/A |
| FV |  |  |  |  |  |  | PARG12818 | hydrolase activity, acting on ester bonds | GPI inositol-deacylase [EC:3.-.-.-] |
| FV |  |  |  |  |  |  | PARG12819 | metal ion binding | #N/A |
| FV |  |  |  |  |  |  | PARG12821 | DNA binding | transcription factor MYB, plant |
| FV |  |  |  |  |  |  | PARG12822 | nucleic acid binding | #N/A |
| FV |  |  |  |  |  |  | PARG12828 | hydrolase activity, hydrolyzing O-glycosyl compounds | #N/A |
| FV |  |  |  |  |  |  | PARG12829 | proton-transporting ATP synthase complex assembly | ATP synthase mitochondrial F1 complex assembly factor 2 |
| FV |  |  |  |  |  |  | PARG12830 | tricarboxylic acid cycle, 2 iron, 2 sulfur cluster binding | succinate dehydrogenase (ubiquinone) iron-sulfur subunit [EC:1.3.5.1] |
| FV |  |  |  |  |  |  | PARG12831 | electron transfer activity | #N/A |
| FV |  |  |  |  |  |  | PARG12835 | ubiquitin-ubiquitin ligase activity | #N/A |
| FV |  |  |  |  |  |  | PARG12836 | electron transfer activity | #N/A |
| FV |  |  |  |  |  |  | PARG12837 | ubiquitin-protein transferase activity | #N/A |
| FV |  |  |  |  |  |  | PARG12838 | protein binding | #N/A |
| FV |  |  |  |  |  |  | PARG12839 | protein binding | #N/A |
| FV |  |  |  |  |  |  | PARG12842 | metalloendopeptidase activity | mitochondrial inner membrane protease ATP23 [EC:3.4.24.-] |
| FV |  |  |  |  |  |  | PARG12844 | glycerol-3-phosphate dehydrogenase [NAD+] activity | glycerol-3-phosphate dehydrogenase (NAD+) [EC:1.1.1.8] |
| FV |  |  |  |  |  |  | PARG12845 | hydrolase activity | abhydrolase domain-containing protein 17 [EC:3.1.2.22] |
| FV |  |  |  |  |  |  | PARG12847 | kinase activity | uridine kinase [EC:2.7.1.48] |
| FV |  |  |  |  |  |  | PARG12849 | nucleic acid binding | #N/A |
| FV |  |  |  |  |  |  | PARG12850 | methyltransferase activity | #N/A |
| FV |  |  |  |  |  |  | PARG12851 | threonine-type endopeptidase activity | cytochrome P450 family 724 subfamily B polypeptide 1 [EC:1.14.13.-] |
| FV |  |  |  |  |  |  | PARG12852 | GTP binding | Ras-related protein Rab-2A |
| FV |  |  |  |  |  |  | PARG12853 | protein binding | #N/A |
| FV |  |  |  |  |  |  | PARG12856 | methyltransferase activity | #N/A |
| FV |  |  |  |  |  |  | PARG12858 | hydrolase activity, acting on ester bonds | #N/A |
| FV |  |  |  |  |  |  | PARG12859 | hydrolase activity, acting on ester bonds | #N/A |
| FV |  |  |  |  |  |  | PARG12860 | translation elongation factor activity | elongation factor Tu |
| FV |  |  |  |  |  |  | PARG12862 | SAGA complex | SAGA-associated factor 29 |
| FV |  |  |  |  |  |  | PARG12864 | pseudouridine synthase activity | tRNA pseudouridine55 synthase [EC:5.4.99.25] |
| FV |  |  |  |  |  |  | PARG12866 | ATP binding | glucuronokinase [EC:2.7.1.43] |
| FV |  |  |  |  |  |  | PARG12867 | vesicle fusion with Golgi apparatus | #N/A |
| FV |  |  |  |  |  |  | PARG12871 | beta-1,4-mannosylglycoprotein 4-beta-N-acetylglucosaminyltransferase activity | beta-1,4-mannosyl-glycoprotein beta-1,4-N-acetylglucosaminyltransferase [EC:2.4.1.144] |
| FV |  |  |  |  |  |  | PARG12872 | methyltransferase activity | ribosomal RNA-processing protein 8 [EC:2.1.1.287] |
| FV |  |  |  |  |  |  | PARG12873 | metalloendopeptidase activity | #N/A |
| FV |  |  |  |  |  |  | PARG12874 | intramolecular lyase activity | #N/A |
| FV |  |  |  |  |  |  | PARG12875 | isomerase activity | glucose-6-phosphate 1-epimerase [EC:5.1.3.15] |
| FV |  |  |  |  |  |  | PARG12876 | protein binding | #N/A |
| FV |  |  |  |  |  |  | PARG12878 | protein binding | #N/A |
| FV |  |  |  |  |  |  | PARG12880 | protein kinase activity | heterogeneous nuclear ribonucleoprotein A1/A3 |
| FV |  |  |  |  |  |  | PARG12882 | ATPase-coupled transmembrane transporter activity | #N/A |
| FV |  |  |  |  |  |  | PARG12883 | structural constituent of ribosome | large subunit ribosomal protein L27Ae |
| FV |  |  |  |  |  |  | PARG12884 | GPI-anchor transamidase complex | #N/A |
| FV |  |  |  |  |  |  | PARG12885 | protein binding | F-box and leucine-rich repeat protein 2/20 |
| FV |  |  |  |  |  |  | PARG12886 | regulation of DNA endoreduplication | #N/A |
| FV |  |  |  |  |  |  | PARG12892 | protein binding | #N/A |
| FV | **S_FV19** | **Sg3** | **Marker62327** | LG4 | 2,627,228 | 3,408,526 | PARG13137 | phosphatidic acid binding | #N/A |
| FV | **S_FV18** | **Sg3** | **Marker62366** |  |  |  | PARG13138 | phosphatidic acid binding | #N/A |
| FV | **H_FV18** | **Hg3** | **Marker62528** |  |  |  | PARG13139 | pectinesterase activity | pectinesterase [EC:3.1.1.11] |
| FV | **S_FV19** | **Sg3** | **Marker62606** |  |  |  | PARG13141 | zinc ion binding | #N/A |
| FV | **H_FV18** | **Hg3** | **Marker62787** |  |  |  | PARG13142 | SNAP receptor activity | vesicle transport through interaction with t-SNAREs 1 |
| FV |  |  |  |  |  |  | PARG13144 | membrane | #N/A |
| FV |  |  |  |  |  |  | PARG13145 | membrane | #N/A |
| FV |  |  |  |  |  |  | PARG13146 | DNA-binding transcription factor activity | EREBP-like factor |
| FV |  |  |  |  |  |  | PARG13147 | protein serine/threonine kinase activity | 5'-AMP-activated protein kinase, catalytic alpha subunit [EC:2.7.11.11] |
| FV |  |  |  |  |  |  | PARG13148 | electron transfer activity | #N/A |
| FV |  |  |  |  |  |  | PARG13151 | protein kinase activity | #N/A |
| FV |  |  |  |  |  |  | PARG13152 | sequence-specific DNA binding | #N/A |
| FV |  |  |  |  |  |  | PARG13153 | regulation of division septum assembly | #N/A |
| FV |  |  |  |  |  |  | PARG13154 | regulation of division septum assembly | #N/A |
| FV |  |  |  |  |  |  | PARG13155 | electron transfer activity | #N/A |
| FV |  |  |  |  |  |  | PARG13156 | chorismate mutase activity | chorismate mutase [EC:5.4.99.5] |
| FV |  |  |  |  |  |  | PARG13157 | protein binding | #N/A |
| FV |  |  |  |  |  |  | PARG13166 | cellular response to nitrate | #N/A |
| FV |  |  |  |  |  |  | PARG13177 | protein dimerization activity | #N/A |
| FV |  |  |  |  |  |  | PARG13181 | calcium ion binding | #N/A |
| FV |  |  |  |  |  |  | PARG13185 | protein binding | #N/A |
| FV |  |  |  |  |  |  | PARG13186 | transferase activity, transferring hexosyl groups | 2-hydroxyflavanone C-glucosyltransferase [EC:2.4.1.360] |
| FV |  |  |  |  |  |  | PARG13187 | transferase activity, transferring hexosyl groups | 2-hydroxyflavanone C-glucosyltransferase [EC:2.4.1.360] |
| FV |  |  |  |  |  |  | PARG13188 | RNA methyltransferase activity | #N/A |
| FV |  |  |  |  |  |  | PARG13193 | electron transfer activity | #N/A |
| FV |  |  |  |  |  |  | PARG13194 | protein kinase activity | #N/A |
| FV |  |  |  |  |  |  | PARG13195 | electron transfer activity | #N/A |
| FV |  |  |  |  |  |  | PARG13196 | ubiquitin-protein transferase activity | ubiquitin conjugation factor E4 B [EC:2.3.2.27] |
| FV |  |  |  |  |  |  | PARG13198 | protein tyrosine/serine/threonine phosphatase activity | phosphatidylinositol-3,4,5-trisphosphate 3-phosphatase and dual-specificity protein phosphatase PTEN [EC:3.1.3.16 3.1.3.48 3.1.3.67] |
| FV |  |  |  |  |  |  | PARG13199 | ion channel activity | cyclic nucleotide gated channel, plant |
| FV |  |  |  |  |  |  | PARG13202 | calmodulin binding | #N/A |
| FV |  |  |  |  |  |  | PARG13203 | protein binding | #N/A |
| FV |  |  |  |  |  |  | PARG13205 | ATP binding | #N/A |
| FV |  |  |  |  |  |  | PARG13207 | protein binding | #N/A |
| FV |  |  |  |  |  |  | PARG13208 | polygalacturonate 4-alpha-galacturonosyltransferase activity | galacturonosyltransferase 12/13/14/15 [EC:2.4.1.-] |
| FV |  |  |  |  |  |  | PARG13209 | protein binding | #N/A |
| FV |  |  |  |  |  |  | PARG13211 | glycogen phosphorylase activity | glycogen phosphorylase [EC:2.4.1.1] |
| FV |  |  |  |  |  |  | PARG13212 | nucleic acid binding | #N/A |
| FV |  |  |  |  |  |  | PARG13214 | UDP-glucose 6-dehydrogenase activity | UDPglucose 6-dehydrogenase [EC:1.1.1.22] |
| FV |  |  |  |  |  |  | PARG13215 | protein binding | #N/A |
| FV |  |  |  |  |  |  | PARG13217 | nucleic acid binding | #N/A |
| FV |  |  |  |  |  |  | PARG13219 | activation of protein kinase activity | #N/A |
| FV |  |  |  |  |  |  | PARG13220 | structural constituent of ribosome | small subunit ribosomal protein S19e |
| FV |  |  |  |  |  |  | PARG13221 | metalloendopeptidase activity | mitochondrial-processing peptidase subunit beta [EC:3.4.24.64] |
| FV |  |  |  |  |  |  | PARG13222 | acetyl-CoA carboxylase activity | #N/A |
| FV |  |  |  |  |  |  | PARG13223 | extracellular region | #N/A |
| FV |  |  |  |  |  |  | PARG13224 | protein binding | #N/A |
| FV |  |  |  |  |  |  | PARG13225 | transferase activity, transferring hexosyl groups | #N/A |
| FV |  |  |  |  |  |  | PARG13226 | transferase activity, transferring hexosyl groups | #N/A |
| FV |  |  |  |  |  |  | PARG13227 | transferase activity, transferring hexosyl groups | #N/A |
| FV |  |  |  |  |  |  | PARG13228 | transferase activity, transferring hexosyl groups | #N/A |
| FV |  |  |  |  |  |  | PARG13231 | regulation of transcription, DNA-templated | #N/A |
| FV |  |  |  |  |  |  | PARG13232 | regulation of transcription, DNA-templated | transcription initiation factor TFIIB |
| FV |  |  |  |  |  |  | PARG13233 | alpha-amylase activity | alpha-amylase [EC:3.2.1.1] |
| FV |  |  |  |  |  |  | PARG13235 | RNA binding | #N/A |
| FV |  |  |  |  |  |  | PARG13236 | exocytosis | exocyst complex component 7 |
| FV |  |  |  |  |  |  | PARG13237 | serine-type carboxypeptidase activity | serine carboxypeptidase-like clade II [EC:3.4.16.-] |
| FV |  |  |  |  |  |  | PARG13242 | protein kinase activity | #N/A |
| FV |  |  |  |  |  |  | PARG13243 | signal transduction | #N/A |
| FV |  |  |  |  |  |  | PARG13244 | transferase activity, transferring acyl groups other than amino-acyl groups | #N/A |
| FV |  |  |  |  |  |  | PARG13245 | transferase activity, transferring acyl groups other than amino-acyl groups | #N/A |
| FV |  |  |  |  |  |  | PARG13246 | transferase activity, transferring acyl groups other than amino-acyl groups | #N/A |
| FV |  |  |  |  |  |  | PARG13247 | transferase activity, transferring acyl groups other than amino-acyl groups | #N/A |
| FV |  |  |  |  |  |  | PARG13248 | transferase activity, transferring acyl groups other than amino-acyl groups | #N/A |
| FV |  |  |  |  |  |  | PARG13249 | transferase activity, transferring acyl groups other than amino-acyl groups | #N/A |
| FV |  |  |  |  |  |  | PARG13250 | transferase activity, transferring acyl groups other than amino-acyl groups | #N/A |
| FV |  |  |  |  |  |  | PARG13251 | transferase activity, transferring acyl groups other than amino-acyl groups | #N/A |
| FV |  |  |  |  |  |  | PARG13252 | transferase activity, transferring acyl groups other than amino-acyl groups | #N/A |
| FV |  |  |  |  |  |  | PARG13254 | DNA-binding transcription factor activity | #N/A |
| FV |  |  |  |  |  |  | PARG13255 | protein heterodimerization activity | transcription initiation factor TFIID subunit 8 |
| FV |  |  |  |  |  |  | PARG13256 | regulation of monopolar cell growth | #N/A |
| FV |  |  |  |  |  |  | PARG13260 | translation initiation factor activity | translation initiation factor 3 subunit M |
| FV |  |  |  |  |  |  | PARG13261 | protein kinase activity | #N/A |
| FV |  |  |  |  |  |  | PARG13263 | structural constituent of ribosome | #N/A |
| FV |  |  |  |  |  |  | PARG13264 | protein kinase activity | #N/A |
| FV |  |  |  |  |  |  | PARG13266 | protein kinase activity | #N/A |
| FV |  |  |  |  |  |  | PARG13267 | ATP binding | #N/A |
| FV |  |  |  |  |  |  | PARG13271 | protein kinase activity | #N/A |
| FV |  |  |  |  |  |  | PARG13272 | protein kinase activity | #N/A |
| FV |  |  |  |  |  |  | PARG13273 | protein kinase activity | #N/A |
| FV |  |  |  |  |  |  | PARG13280 | protein kinase activity | #N/A |
| FV |  |  |  |  |  |  | PARG13281 | protein kinase activity | #N/A |
| FV |  |  |  |  |  |  | PARG13283 | protein kinase activity | #N/A |
| FV |  |  |  |  |  |  | PARG13284 | protein kinase activity | #N/A |
| FV |  |  |  |  |  |  | PARG13286 | methyltransferase activity | #N/A |
| FV |  |  |  |  |  |  | PARG13287 | transmembrane transporter activity | #N/A |
| FV |  |  |  |  |  |  | PARG13291 | sulfate transmembrane transporter activity | #N/A |
| FV |  |  |  |  |  |  | PARG13292 | hydrolase activity, hydrolyzing O-glycosyl compounds | #N/A |
| FV | **H_FV18** | **Hg3** | **Marker58893** | LG4 | 7,297,721 | 7,720,239 | PARG13880 | structural constituent of nuclear pore | nuclear pore complex protein Nup133 |
| FV | H_FW18 | Hg3 | Marker58893 |  |  |  | PARG13881 | protein binding | #N/A |
| FV | H_FV18 | Hg3 | Marker59314 |  |  |  | PARG13882 | intracellular protein transport | vacuolar protein sorting-associated protein 11 |
| FV | H_FV18 | Hg3 | Marker59427 |  |  |  | PARG13883 | metal ion binding | #N/A |
| FV |  |  |  |  |  |  | PARG13886 | protein binding | #N/A |
| FV |  |  |  |  |  |  | PARG13887 | protein binding | #N/A |
| FV |  |  |  |  |  |  | PARG13888 | mRNA processing | #N/A |
| FV |  |  |  |  |  |  | PARG13890 | mRNA processing | #N/A |
| FV |  |  |  |  |  |  | PARG13892 | protein binding | #N/A |
| FV |  |  |  |  |  |  | PARG13893 | protein binding | #N/A |
| FV |  |  |  |  |  |  | PARG13896 | protein binding | #N/A |
| FV |  |  |  |  |  |  | PARG13897 | protein binding | #N/A |
| FV |  |  |  |  |  |  | PARG13899 | transcription coactivator activity | transcriptional adapter 2-alpha |
| FV |  |  |  |  |  |  | PARG13900 | cysteine-type peptidase activity | #N/A |
| FV |  |  |  |  |  |  | PARG13901 | protein binding | #N/A |
| FV |  |  |  |  |  |  | PARG13902 | glucose-6-phosphate dehydrogenase activity | glucose-6-phosphate 1-dehydrogenase [EC:1.1.1.49 1.1.1.363] |
| FV |  |  |  |  |  |  | PARG13904 | zinc ion binding | #N/A |
| FV |  |  |  |  |  |  | PARG13905 | protein homodimerization activity | #N/A |
| FV |  |  |  |  |  |  | PARG13907 | alpha-1,6-mannosylglycoprotein 2-beta-N-acetylglucosaminyltransferase activity | alpha-1,6-mannosyl-glycoprotein beta-1,2-N-acetylglucosaminyltransferase [EC:2.4.1.143] |
| FV |  |  |  |  |  |  | PARG13909 | protein binding | #N/A |
| FV |  |  |  |  |  |  | PARG13911 | regulation of transcription, DNA-templated | nuclear transcription Y subunit beta |
| FV |  |  |  |  |  |  | PARG13913 | hydrolase activity | #N/A |
| FV |  |  |  |  |  |  | PARG13914 | transferase activity, transferring acyl groups other than amino-acyl groups | acetyl-CoA C-acetyltransferase [EC:2.3.1.9] |
| FV |  |  |  |  |  |  | PARG13916 | chromatin binding | #N/A |
| FV |  |  |  |  |  |  | PARG13917 | chromatin binding | #N/A |
| FV |  |  |  |  |  |  | PARG13918 | indole-3-glycerol-phosphate synthase activity | indole-3-glycerol phosphate synthase [EC:4.1.1.48] |
| FV |  |  |  |  |  |  | PARG13919 | protein binding | #N/A |
| FV |  |  |  |  |  |  | PARG13920 | protein binding | #N/A |
| FV |  |  |  |  |  |  | PARG13921 | protein binding | #N/A |
| FV |  |  |  |  |  |  | PARG13922 | indole-3-glycerol-phosphate synthase activity | #N/A |
| FV |  |  |  |  |  |  | PARG13923 | protein binding | #N/A |
| FV |  |  |  |  |  |  | PARG13925 | catalytic activity | long-chain acyl-CoA synthetase [EC:6.2.1.3] |
| FV |  |  |  |  |  |  | PARG13926 | nucleic acid binding | heterogeneous nuclear ribonucleoprotein A1/A3 |
| FV |  |  |  |  |  |  | PARG13927 | protein binding | #N/A |
| FV |  |  |  |  |  |  | PARG13929 | protein binding | vacuolar protein 8 |
| FV |  |  |  |  |  |  | PARG13930 | ribonuclease activity | #N/A |
| FV |  |  |  |  |  |  | PARG13933 | rRNA processing | #N/A |
| FV |  |  |  |  |  |  | PARG13934 | zinc ion binding | #N/A |
| FV |  |  |  |  |  |  | PARG13935 | catalytic activity | #N/A |
| FV |  |  |  |  |  |  | PARG13936 | protein binding | #N/A |
| FV |  |  |  |  |  |  | PARG13938 | zinc ion binding | #N/A |
| FV |  |  |  |  |  |  | PARG13939 | iron ion binding | cytochrome P450 family 724 subfamily B polypeptide 1 [EC:1.14.13.-] |
| FV |  |  |  |  |  |  | PARG13940 | serine-type endopeptidase activity | #N/A |
| FV |  |  |  |  |  |  | PARG13941 | polygalacturonase activity | #N/A |
| FV |  |  |  |  |  |  | PARG13943 | transferase activity, transferring glycosyl groups | #N/A |
| FV |  |  |  |  |  |  | PARG13946 | protein binding | 25S rRNA (uracil2634-N3)-methyltransferase [EC:2.1.1.313] |
| FV |  |  |  |  |  |  | PARG13949 | glucose-1-phosphate adenylyltransferase activity | glucose-1-phosphate adenylyltransferase [EC:2.7.7.27] |
| FV |  |  |  |  |  |  | PARG13950 | DNA binding | #N/A |
| FV |  |  |  |  |  |  | PARG13951 | cation transmembrane transporter activity | #N/A |
| FV |  |  |  |  |  |  | PARG13953 | DNA binding | #N/A |
| FV |  |  |  |  |  |  | PARG13954 | integral component of membrane | transmembrane 9 superfamily member 2/4 |
| FV |  |  |  |  |  |  | PARG13956 | nucleic acid binding | #N/A |
| FV |  |  |  |  |  |  | PARG13957 | GARP complex | vacuolar protein sorting-associated protein 53 |
| FV |  |  |  |  |  |  | PARG13958 | zinc ion binding | #N/A |
| FV | **S_FV19** | **Sg3** | **Marker57693** | LG4 | 9,435,929 | 9,436,029 | PARG14209 | DNA helicase activity | ATP-dependent DNA helicase PIF1 [EC:3.6.4.12] |
| FV |  |  |  |  |  |  | PARG14212 | protein dimerization activity | #N/A |
| FV |  |  |  |  |  |  | PARG14213 | DNA-binding transcription factor activity | MADS-box transcription factor, plant |
| FV |  |  |  |  |  |  | PARG14214 | transferase activity, transferring glycosyl groups | galacturonosyltransferase 12/13/14/15 [EC:2.4.1.-] |
| FV |  |  |  |  |  |  | PARG14215 | RNA polymerase III transcription regulator recruiting activity | #N/A |
| FV |  |  |  |  |  |  | PARG14216 | transcription by RNA polymerase III | general transcription factor 3C polypeptide 3 (transcription factor C subunit 4) |
| FV |  |  |  |  |  |  | PARG14217 | dTDP-glucose 4,6-dehydratase activity | UDP-glucose 4,6-dehydratase [EC:4.2.1.76] |
| FV |  |  |  |  |  |  | PARG14220 | iron ion binding | #N/A |
| FV |  |  |  |  |  |  | PARG14221 | protein kinase activity | #N/A |
| FV |  |  |  |  |  |  | PARG14222 | protein kinase activity | #N/A |
| FL | **S_FL19** | **Sg3** | **Marker58388** | LG4 | 8,919,795 | 9,206,314 | PARG14129 | photosystem II oxygen evolving complex | photosystem II oxygen-evolving enhancer protein 2 |
| FL | **S_FL19** | **Sg3** | **Marker57739** |  |  |  | PARG14130 | protein binding | WD repeat-containing protein 26 |
| FL |  |  |  |  |  |  | PARG14131 | helicase activity | ATP-dependent RNA helicase DHX36 [EC:3.6.4.13] |
| FL |  |  |  |  |  |  | PARG14133 | integral component of membrane | regulatory protein NPR1 |
| FL |  |  |  |  |  |  | PARG14134 | DNA binding | #N/A |
| FL |  |  |  |  |  |  | PARG14135 | male meiosis II | #N/A |
| FL |  |  |  |  |  |  | PARG14136 | protein binding | glutathione S-transferase [EC:2.5.1.18] |
| FL |  |  |  |  |  |  | PARG14137 | protein binding | glutathione S-transferase [EC:2.5.1.18] |
| FL |  |  |  |  |  |  | PARG14139 | regulation of transcription, DNA-templated | #N/A |
| FL |  |  |  |  |  |  | PARG14141 | anaphase-promoting complex | anaphase-promoting complex subunit 5 |
| FL |  |  |  |  |  |  | PARG14142 | 4-hydroxyphenylpyruvate dioxygenase activity | 4-hydroxyphenylpyruvate dioxygenase [EC:1.13.11.27] |
| FL |  |  |  |  |  |  | PARG14143 | hydrolase activity, acting on ester bonds | #N/A |
| FL |  |  |  |  |  |  | PARG14144 | response to auxin | #N/A |
| FL |  |  |  |  |  |  | PARG14145 | methyltransferase activity | methyltransferase NSUN6 [EC:2.1.1.-] |
| FL |  |  |  |  |  |  | PARG14147 | aspartic-type endopeptidase activity | aspartyl protease family protein [EC:3.4.23.-] |
| FL |  |  |  |  |  |  | PARG14148 | 3-hydroxyisobutyryl-CoA hydrolase activity | 3-hydroxyisobutyryl-CoA hydrolase [EC:3.1.2.4] |
| FL |  |  |  |  |  |  | PARG14149 | protein binding | vacuolar protein sorting-associated protein 16 |
| FL |  |  |  |  |  |  | PARG14153 | diacylglycerol kinase activity | #N/A |
| FL |  |  |  |  |  |  | PARG14159 | Golgi transport complex | conserved oligomeric Golgi complex subunit 5 |
| FL |  |  |  |  |  |  | PARG14161 | 1,3-beta-D-glucan synthase activity | 1,3-beta-glucan synthase [EC:2.4.1.34] |
| FL |  |  |  |  |  |  | PARG14162 | photosynthesis, light harvesting | #N/A |
| FL |  |  |  |  |  |  | PARG14163 | photosynthesis, light harvesting | light-harvesting complex II chlorophyll a/b binding protein 1 |
| FL |  |  |  |  |  |  | PARG14167 | protein kinase activity | #N/A |
| FL |  |  |  |  |  |  | PARG14169 | sequence-specific DNA binding | #N/A |
| FL |  |  |  |  |  |  | PARG14171 | potassium channel activity | potassium channel subfamily K, other eukaryote |
| FL |  |  |  |  |  |  | PARG14172 | potassium channel activity | #N/A |
| FL |  |  |  |  |  |  | PARG14173 | metal ion transmembrane transporter activity | #N/A |
| FL |  |  |  |  |  |  | PARG14174 | protein binding | #N/A |
| FL |  |  |  |  |  |  | PARG14175 | AT DNA binding | #N/A |
| FL |  |  |  |  |  |  | PARG14177 | cysteine-type peptidase activity | #N/A |
| FL |  |  |  |  |  |  | PARG14178 | xyloglucosyl transferase activity | xyloglucan:xyloglucosyl transferase [EC:2.4.1.207] |
| FL |  |  |  |  |  |  | PARG14179 | xyloglucosyl transferase activity | xyloglucan:xyloglucosyl transferase TCH4 [EC:2.4.1.207] |
| FL |  |  |  |  |  |  | PARG14184 | protein kinase activity | #N/A |
| FL |  |  |  |  |  |  | PARG14185 | nucleic acid binding | #N/A |
| SSC | **H_SSC18** | **Hg2** | **Marker121293** | LG5 | 4,846,409 | 6,655,777 | PARG17510 | pyruvate kinase activity | pyruvate kinase [EC:2.7.1.40] |
| SSC | H_SSC18 | Hg2 | Marker66701 |  |  |  | PARG17511 | DNA binding | #N/A |
| SSC | S_SSC18 | Sg2 | Marker65691 |  |  |  | PARG17512 | regulation of transcription, DNA-templated | #N/A |
| SSC | H_SSC19 | Hg2 | Marker121293 |  |  |  | PARG17513 | malate dehydrogenase (NADP+) activity | malate dehydrogenase (NADP+) [EC:1.1.1.82] |
| SSC |  |  |  |  |  |  | PARG17514 | catalytic activity | OPC-8:0 CoA ligase 1 [EC:6.2.1.-] |
| SSC |  |  |  |  |  |  | PARG17517 | protein binding | #N/A |
| SSC |  |  |  |  |  |  | PARG17519 | oxidoreductase activity, acting on the aldehyde or oxo group of donors, disulfide as acceptor | #N/A |
| SSC |  |  |  |  |  |  | PARG17520 | hydrolase activity, hydrolyzing O-glycosyl compounds | beta-glucosidase [EC:3.2.1.21] |
| SSC |  |  |  |  |  |  | PARG17523 | protein kinase activity | #N/A |
| SSC |  |  |  |  |  |  | PARG17524 | proteolysis | #N/A |
| SSC |  |  |  |  |  |  | PARG17525 | cysteine-type peptidase activity | #N/A |
| SSC |  |  |  |  |  |  | PARG17528 | proteolysis | #N/A |
| SSC |  |  |  |  |  |  | PARG17530 | ATP binding | #N/A |
| SSC |  |  |  |  |  |  | PARG17531 | protein phosphorylation | #N/A |
| SSC |  |  |  |  |  |  | PARG17533 | RNA binding | RNA-binding protein Nova |
| SSC |  |  |  |  |  |  | PARG17536 | electron transfer activity | #N/A |
| SSC |  |  |  |  |  |  | PARG17540 | oxidoreductase activity | #N/A |
| SSC |  |  |  |  |  |  | PARG17542 | protein binding | #N/A |
| SSC |  |  |  |  |  |  | PARG17544 | protein binding | #N/A |
| SSC |  |  |  |  |  |  | PARG17545 | protein binding | #N/A |
| SSC |  |  |  |  |  |  | PARG17546 | protein binding | #N/A |
| SSC |  |  |  |  |  |  | PARG17548 | carbohydrate phosphatase activity | #N/A |
| SSC |  |  |  |  |  |  | PARG17557 | protein binding | prolactin regulatory element-binding protein |
| SSC |  |  |  |  |  |  | PARG17558 | protein binding | #N/A |
| SSC |  |  |  |  |  |  | PARG17561 | transferase activity, transferring glycosyl groups | #N/A |
| SSC |  |  |  |  |  |  | PARG17562 | protein binding | #N/A |
| SSC |  |  |  |  |  |  | PARG17563 | protein dimerization activity | #N/A |
| SSC |  |  |  |  |  |  | PARG17568 | protein dimerization activity | #N/A |
| SSC |  |  |  |  |  |  | PARG17574 | protein binding | #N/A |
| SSC |  |  |  |  |  |  | PARG17576 | hydrolase activity | 5-oxoprolinase (ATP-hydrolysing) [EC:3.5.2.9] |
| SSC |  |  |  |  |  |  | PARG17577 | catalytic activity | acyl-CoA synthetase [EC:6.2.1.-] |
| SSC |  |  |  |  |  |  | PARG17578 | protein binding | #N/A |
| SSC |  |  |  |  |  |  | PARG17580 | protein binding | #N/A |
| SSC |  |  |  |  |  |  | PARG17583 | nucleic acid binding | #N/A |
| SSC |  |  |  |  |  |  | PARG17587 | protein binding | #N/A |
| SSC |  |  |  |  |  |  | PARG17592 | catalytic activity | phospholipase C [EC:3.1.4.3] |
| SSC |  |  |  |  |  |  | PARG17593 | catalytic activity | phospholipase C [EC:3.1.4.3] |
| SSC |  |  |  |  |  |  | PARG17597 | structural constituent of nuclear pore | nuclear pore complex protein Nup98-Nup96 |
| SSC |  |  |  |  |  |  | PARG17599 | transcription corepressor activity | #N/A |
| SSC |  |  |  |  |  |  | PARG17600 | mRNA splicing, via spliceosome | #N/A |
| SSC |  |  |  |  |  |  | PARG17601 | ubiquitin-dependent protein catabolic process | S-phase kinase-associated protein 1 |
| SSC |  |  |  |  |  |  | PARG17603 | pectinesterase activity | #N/A |
| SSC |  |  |  |  |  |  | PARG17605 | ubiquitin-dependent protein catabolic process | S-phase kinase-associated protein 1 |
| SSC |  |  |  |  |  |  | PARG17606 | ubiquitin-dependent protein catabolic process | #N/A |
| SSC |  |  |  |  |  |  | PARG17608 | ubiquitin-dependent protein catabolic process | S-phase kinase-associated protein 1 |
| SSC |  |  |  |  |  |  | PARG17609 | pectinesterase activity | #N/A |
| SSC |  |  |  |  |  |  | PARG17611 | ubiquitin-dependent protein catabolic process | S-phase kinase-associated protein 1 |
| SSC |  |  |  |  |  |  | PARG17612 | ATP binding | #N/A |
| SSC |  |  |  |  |  |  | PARG17614 | pectinesterase activity | #N/A |
| SSC |  |  |  |  |  |  | PARG17615 | DNA binding | #N/A |
| SSC |  |  |  |  |  |  | PARG17616 | protein kinase activity | #N/A |
| SSC |  |  |  |  |  |  | PARG17617 | protein kinase activity | #N/A |
| SSC |  |  |  |  |  |  | PARG17620 | pectinesterase activity | #N/A |
| SSC |  |  |  |  |  |  | PARG17621 | ubiquitin-dependent protein catabolic process | S-phase kinase-associated protein 1 |
| SSC |  |  |  |  |  |  | PARG17623 | transmembrane transport | #N/A |
| SSC |  |  |  |  |  |  | PARG17624 | GTPase activity | #N/A |
| SSC |  |  |  |  |  |  | PARG17626 | protein binding | glutathione S-transferase [EC:2.5.1.18] |
| SSC |  |  |  |  |  |  | PARG17629 | transferase activity, transferring hexosyl groups | #N/A |
| SSC |  |  |  |  |  |  | PARG17630 | signal transduction | #N/A |
| SSC |  |  |  |  |  |  | PARG17632 | protein binding | #N/A |
| SSC |  |  |  |  |  |  | PARG17634 | protein binding | #N/A |
| SSC |  |  |  |  |  |  | PARG17637 | protein binding | #N/A |
| SSC |  |  |  |  |  |  | PARG17638 | protein binding | #N/A |
| SSC |  |  |  |  |  |  | PARG17639 | aspartic-type endopeptidase activity | #N/A |
| SSC |  |  |  |  |  |  | PARG17640 | aspartic-type endopeptidase activity | #N/A |
| SSC |  |  |  |  |  |  | PARG17642 | DNA-directed 5'-3' RNA polymerase activity | DNA-directed RNA polymerase III subunit RPC8 |
| SSC |  |  |  |  |  |  | PARG17643 | protein kinase activity | interleukin-1 receptor-associated kinase 4 [EC:2.7.11.1] |
| SSC |  |  |  |  |  |  | PARG17644 | protein kinase activity | #N/A |
| SSC |  |  |  |  |  |  | PARG17645 | oxidoreductase activity | #N/A |
| SSC |  |  |  |  |  |  | PARG17647 | polysaccharide binding | interleukin-1 receptor-associated kinase 4 [EC:2.7.11.1] |
| SSC |  |  |  |  |  |  | PARG17648 | protein kinase activity | #N/A |
| SSC |  |  |  |  |  |  | PARG17649 | polysaccharide binding | #N/A |
| SSC |  |  |  |  |  |  | PARG17651 | protein kinase activity | #N/A |
| SSC |  |  |  |  |  |  | PARG17652 | protein kinase activity | #N/A |
| SSC |  |  |  |  |  |  | PARG17653 | microtubule minus-end binding | #N/A |
| SSC |  |  |  |  |  |  | PARG17654 | transmembrane transporter activity | #N/A |
| SSC |  |  |  |  |  |  | PARG17655 | DNA binding | #N/A |
| SSC |  |  |  |  |  |  | PARG17657 | protein binding | #N/A |
| SSC |  |  |  |  |  |  | PARG17661 | damaged DNA binding | #N/A |
| SSC |  |  |  |  |  |  | PARG17662 | ligand-gated ion channel activity | glutamate receptor, ionotropic, plant |
| SSC |  |  |  |  |  |  | PARG17663 | unfolded protein binding | #N/A |
| SSC |  |  |  |  |  |  | PARG17664 | ligand-gated ion channel activity | glutamate receptor, ionotropic, plant |
| SSC |  |  |  |  |  |  | PARG17665 | ligand-gated ion channel activity | glutamate receptor, ionotropic, plant |
| SSC |  |  |  |  |  |  | PARG17668 | ligand-gated ion channel activity | #N/A |
| SSC |  |  |  |  |  |  | PARG17671 | ligand-gated ion channel activity | glutamate receptor, ionotropic, plant |
| SSC |  |  |  |  |  |  | PARG17677 | tRNA (guanine-N7-)-methyltransferase activity | #N/A |
| SSC |  |  |  |  |  |  | PARG17682 | O-methyltransferase activity | caffeoyl-CoA O-methyltransferase [EC:2.1.1.104] |
| SSC |  |  |  |  |  |  | PARG17683 | structural constituent of ribosome | large subunit ribosomal protein L3 |
| SSC |  |  |  |  |  |  | PARG17684 | uridylyltransferase activity | UTP--glucose-1-phosphate uridylyltransferase [EC:2.7.7.9] |
| SSC |  |  |  |  |  |  | PARG17685 | defense response | #N/A |
| SSC |  |  |  |  |  |  | PARG17688 | zinc ion binding | protein transport protein SEC23 |
| SSC |  |  |  |  |  |  | PARG17689 | DNA binding | replication factor C subunit 3/5 |
| SSC |  |  |  |  |  |  | PARG17691 | AT DNA binding | #N/A |
| SSC |  |  |  |  |  |  | PARG17692 | metal ion transport | #N/A |
| SSC |  |  |  |  |  |  | PARG17693 | oxidation-reduction process | ent-kaurenoic acid monooxygenase [EC:1.14.14.107] |
| SSC |  |  |  |  |  |  | PARG17694 | GTP binding | mitochondrial GTPase 1 |
| SSC |  |  |  |  |  |  | PARG17695 | DNA binding | #N/A |
| SSC |  |  |  |  |  |  | PARG17696 | ubiquitin-protein transferase activity | #N/A |
| SSC |  |  |  |  |  |  | PARG17697 | 3-hydroxyisobutyryl-CoA hydrolase activity | 3-hydroxyisobutyryl-CoA hydrolase [EC:3.1.2.4] |
| SSC |  |  |  |  |  |  | PARG17699 | ubiquitin-dependent protein catabolic process | #N/A |
| SSC |  |  |  |  |  |  | PARG17700 | DNA binding | #N/A |
| SSC |  |  |  |  |  |  | PARG17701 | protein kinase activity | #N/A |
| SSC |  |  |  |  |  |  | PARG17702 | iron ion binding | ent-kaurenoic acid monooxygenase [EC:1.14.14.107] |
| SSC |  |  |  |  |  |  | PARG17703 | iron ion binding | ent-kaurenoic acid monooxygenase [EC:1.14.14.107] |
| SSC |  |  |  |  |  |  | PARG17704 | protein kinase activity | #N/A |
| SSC |  |  |  |  |  |  | PARG17705 | oxidation-reduction process | #N/A |
| SSC |  |  |  |  |  |  | PARG17706 | iron ion binding | ent-kaurenoic acid monooxygenase [EC:1.14.14.107] |
| SSC |  |  |  |  |  |  | PARG17708 | protein kinase activity | BR-signaling kinase [EC:2.7.11.1] |
| SSC |  |  |  |  |  |  | PARG17710 | transmembrane transporter activity | #N/A |
| SSC |  |  |  |  |  |  | PARG17712 | ribose phosphate diphosphokinase activity | ribose-phosphate pyrophosphokinase [EC:2.7.6.1] |
| SSC |  |  |  |  |  |  | PARG17713 | nutrient reservoir activity | #N/A |
| SSC |  |  |  |  |  |  | PARG17714 | protein dimerization activity | #N/A |
| SSC |  |  |  |  |  |  | PARG17715 | protein binding | #N/A |
| SSC |  |  |  |  |  |  | PARG17716 | protein binding | #N/A |
| SSC |  |  |  |  |  |  | PARG17717 | protein binding | #N/A |
| SSC |  |  |  |  |  |  | PARG17719 | ATP binding | #N/A |
| SSC |  |  |  |  |  |  | PARG17720 | ATPase-coupled transmembrane transporter activity | ATP-binding cassette, subfamily C (CFTR/MRP), member 1 [EC:7.6.2.3] |
| SSC |  |  |  |  |  |  | PARG17721 | ADP binding | #N/A |
| SSC |  |  |  |  |  |  | PARG17723 | hydrolase activity | #N/A |
| SSC |  |  |  |  |  |  | PARG17724 | carbohydrate metabolic process | endoglucanase [EC:3.2.1.4] |
| SSC |  |  |  |  |  |  | PARG17725 | catalytic activity | diaminohydroxyphosphoribosylaminopyrimidine deaminase / 5-amino-6-(5-phosphoribosylamino)uracil reductase [EC:3.5.4.26 1.1.1.193] |
| SSC |  |  |  |  |  |  | PARG17726 | single-stranded DNA helicase activity | DNA replication ATP-dependent helicase Dna2 [EC:3.6.4.12] |
| SSC |  |  |  |  |  |  | PARG17727 | catalytic activity | #N/A |
| SSC |  |  |  |  |  |  | PARG17728 | oxidoreductase activity | aldehyde decarbonylase [EC:4.1.99.5] |
| SSC |  |  |  |  |  |  | PARG17729 | oxidoreductase activity | aldehyde decarbonylase [EC:4.1.99.5] |
| SSC |  |  |  |  |  |  | PARG17730 | oxidoreductase activity | aldehyde decarbonylase [EC:4.1.99.5] |
| SSC |  |  |  |  |  |  | PARG17733 | structural constituent of ribosome | large subunit ribosomal protein L18 |
| SSC |  |  |  |  |  |  | PARG17734 | uridylate kinase activity | UMP-CMP kinase [EC:2.7.4.14] |
| SSC |  |  |  |  |  |  | PARG17735 | malate dehydrogenase (decarboxylating) (NAD+) activity | malate dehydrogenase (decarboxylating) [EC:1.1.1.39] |
| SSC |  |  |  |  |  |  | PARG17736 | GTPase activity | #N/A |
| SSC |  |  |  |  |  |  | PARG17738 | hydrolase activity | #N/A |
| SSC |  |  |  |  |  |  | PARG17739 | helicase activity | #N/A |
| SSC |  |  |  |  |  |  | PARG17740 | GTP binding | #N/A |
| SSC |  |  |  |  |  |  | PARG17741 | phosphatidylinositol N-acetylglucosaminyltransferase activity | #N/A |
| SSC |  |  |  |  |  |  | PARG17742 | DNA binding | #N/A |
| SSC |  |  |  |  |  |  | PARG17743 | purine nucleoside transmembrane transporter activity | #N/A |
| SSC |  |  |  |  |  |  | PARG17744 | GTPase activity | Ras-related protein Rab-6A |
| SSC |  |  |  |  |  |  | PARG17748 | enzyme inhibitor activity | #N/A |
| SSC |  |  |  |  |  |  | PARG17750 | ATP binding | #N/A |
| SSC |  |  |  |  |  |  | PARG17753 | protein kinase activity | #N/A |
| SSC |  |  |  |  |  |  | PARG17754 | protein kinase activity | #N/A |
| SSC |  |  |  |  |  |  | PARG17755 | protein kinase activity | #N/A |
| SSC |  |  |  |  |  |  | PARG17756 | protein folding | chaperonin GroES |
| SSC |  |  |  |  |  |  | PARG17761 | ADP binding | #N/A |
| SSC |  |  |  |  |  |  | PARG17764 | ADP binding | #N/A |
| SSC |  |  |  |  |  |  | PARG17768 | dolichyl pyrophosphate Glc1Man9GlcNAc2 alpha-1,3-glucosyltransferase activity | alpha-1,3-glucosyltransferase [EC:2.4.1.265] |
| SSC |  |  |  |  |  |  | PARG17770 | protein kinase activity | #N/A |
| SSC |  |  |  |  |  |  | PARG17771 | integral component of membrane | #N/A |
| SSC |  |  |  |  |  |  | PARG17774 | RNA binding | translation initiation factor 4G |
| SSC |  |  |  |  |  |  | PARG17777 | nucleic acid binding | #N/A |
| SSC |  |  |  |  |  |  | PARG17778 | structural constituent of ribosome | large subunit ribosomal protein L37Ae |
| SSC |  |  |  |  |  |  | PARG17780 | protein kinase regulator activity | #N/A |
| SSC |  |  |  |  |  |  | PARG17781 | iron ion binding | #N/A |
| SSC |  |  |  |  |  |  | PARG17782 | iron ion binding | #N/A |
| SSC |  |  |  |  |  |  | PARG17783 | iron ion binding | #N/A |
| SSC |  |  |  |  |  |  | PARG17784 | monolayer-surrounded lipid storage body | #N/A |
| SSC |  |  |  |  |  |  | PARG17787 | monooxygenase activity | #N/A |
| SSC |  |  |  |  |  |  | PARG17788 | iron ion binding | #N/A |
| SSC |  |  |  |  |  |  | PARG17789 | iron ion binding | #N/A |
| SSC |  |  |  |  |  |  | PARG17791 | protein dimerization activity | #N/A |
| SSC |  |  |  |  |  |  | PARG17792 | small GTPase mediated signal transduction | Ras-related C3 botulinum toxin substrate 1 |
| SSC |  |  |  |  |  |  | PARG17795 | nucleic acid binding | #N/A |
| SSC |  |  |  |  |  |  | PARG17796 | protein binding | #N/A |
| SSC |  |  |  |  |  |  | PARG17797 | metal ion binding | #N/A |
| SSC |  |  |  |  |  |  | PARG17799 | signal transduction | #N/A |
| SSC |  |  |  |  |  |  | PARG17802 | signal transduction | #N/A |
| SSC |  |  |  |  |  |  | PARG17803 | protein binding | #N/A |
| SSC |  |  |  |  |  |  | PARG17804 | ADP binding | #N/A |
| SSC |  |  |  |  |  |  | PARG17805 | protein binding | #N/A |
| SSC |  |  |  |  |  |  | PARG17806 | peptidyl-prolyl cis-trans isomerase activity | #N/A |
| SSC |  |  |  |  |  |  | PARG17809 | DNA-directed 5'-3' RNA polymerase activity | DNA-directed RNA polymerases I, II, and III subunit RPABC1 |
| SSC |  |  |  |  |  |  | PARG17810 | DNA-directed 5'-3' RNA polymerase activity | DNA-directed RNA polymerases I, II, and III subunit RPABC1 |
| SSC |  |  |  |  |  |  | PARG17812 | threonine kinase activity | #N/A |
| SSC |  |  |  |  |  |  | PARG17814 | plant-type cell wall organization | endoglucanase [EC:3.2.1.4] |
| SSC |  |  |  |  |  |  | PARG17815 | protein binding | #N/A |
| SSC |  |  |  |  |  |  | PARG17817 | alpha-1,6-mannosyltransferase activity | alpha-1,6-mannosyltransferase [EC:2.4.1.260] |
| SSC |  |  |  |  |  |  | PARG17818 | protein binding | #N/A |
| SSC |  |  |  |  |  |  | PARG17819 | protein binding | #N/A |
| SSC |  |  |  |  |  |  | PARG17820 | ATP binding | chromodomain-helicase-DNA-binding protein 1-like [EC:3.6.4.12] |
| SSC |  |  |  |  |  |  | PARG17821 | catalytic activity | #N/A |
| SSC |  |  |  |  |  |  | PARG17824 | ubiquitin-protein transferase activity | E3 ubiquitin-protein ligase BRE1 [EC:2.3.2.27] |
| SSC |  |  |  |  |  |  | PARG17826 | aminoacyl-tRNA ligase activity | #N/A |
| SSC |  |  |  |  |  |  | PARG17827 | aminoacyl-tRNA ligase activity | #N/A |
| SSC |  |  |  |  |  |  | PARG17828 | aminoacyl-tRNA ligase activity | #N/A |
| SSC |  |  |  |  |  |  | PARG17829 | DNA-binding transcription factor activity | #N/A |
| SSC |  |  |  |  |  |  | PARG17832 | structural constituent of ribosome | small subunit ribosomal protein S19 |
| SSC |  |  |  |  |  |  | PARG17834 | ATP binding | DnaJ homolog subfamily A member 2 |
| SSC |  |  |  |  |  |  | PARG17836 | protein binding | #N/A |
| SSC |  |  |  |  |  |  | PARG17837 | protein binding | #N/A |
| SSC |  |  |  |  |  |  | PARG17838 | protein binding | #N/A |
| SSC |  |  |  |  |  |  | PARG17841 | DNA binding | transcription factor MYB, plant |
| SSC |  |  |  |  |  |  | PARG17842 | zinc ion binding | #N/A |
| SSC |  |  |  |  |  |  | PARG17848 | RNA binding | #N/A |
| SSC |  |  |  |  |  |  | PARG17859 | regulation of transcription, DNA-templated | #N/A |
| SSC |  |  |  |  |  |  | PARG17860 | RNA binding | poly(rC)-binding protein 3/4 |
| SSC |  |  |  |  |  |  | PARG17863 | signal transduction | #N/A |
| SSC |  |  |  |  |  |  | PARG17866 | ADP binding | #N/A |
| SSC |  |  |  |  |  |  | PARG17871 | ADP binding | #N/A |
| SSC |  |  |  |  |  |  | PARG17876 | ADP binding | #N/A |
| SSC |  |  |  |  |  |  | PARG17877 | ADP binding | #N/A |
| SSC |  |  |  |  |  |  | PARG17878 | protein binding | #N/A |
| SSC |  |  |  |  |  |  | PARG17879 | protein binding | #N/A |
| SSC |  |  |  |  |  |  | PARG17881 | ADP binding | #N/A |
| SSC |  |  |  |  |  |  | PARG17882 | ADP binding | #N/A |
| SSC |  |  |  |  |  |  | PARG17883 | signal transduction | #N/A |
| SSC |  |  |  |  |  |  | PARG17885 | nucleus | #N/A |
| SSC |  |  |  |  |  |  | PARG17886 | Arp2/3 complex-mediated actin nucleation | actin related protein 2/3 complex, subunit 5 |
| SSC |  |  |  |  |  |  | PARG17887 | peptidyl-prolyl cis-trans isomerase activity | #N/A |
| SSC |  |  |  |  |  |  | PARG17888 | transmembrane transporter activity | #N/A |
| SSC |  |  |  |  |  |  | PARG17889 | transmembrane transporter activity | #N/A |
| SSC |  |  |  |  |  |  | PARG17890 | palmitoyl hydrolase activity | palmitoyl-protein thioesterase [EC:3.1.2.22] |
| SSC |  |  |  |  |  |  | PARG17892 | methyltransferase activity | tRNA wybutosine-synthesizing protein 4 [EC:2.1.1.290 2.3.1.231] |
| SSC |  |  |  |  |  |  | PARG17894 | galactosyltransferase activity | #N/A |
| SSC |  |  |  |  |  |  | PARG17895 | protein binding | #N/A |
| SSC |  |  |  |  |  |  | PARG17896 | protein binding | #N/A |
| SSC |  |  |  |  |  |  | PARG17897 | protein binding | #N/A |
| SSC |  |  |  |  |  |  | PARG17898 | protein binding | #N/A |
| SSC |  |  |  |  |  |  | PARG17899 | proton-exporting ATPase activity, phosphorylative mechanism | H+-transporting ATPase [EC:7.1.2.1] |
| SSC |  |  |  |  |  |  | PARG17902 | positive regulation of ubiquitin protein ligase activity | #N/A |
| SSC |  |  |  |  |  |  | PARG17903 | DNA-binding transcription factor activity | EREBP-like factor |
| SSC |  |  |  |  |  |  | PARG17904 | DNA-binding transcription factor activity | EREBP-like factor |
| SSC |  |  |  |  |  |  | PARG17905 | DNA-binding transcription factor activity | EREBP-like factor |
| SSC |  |  |  |  |  |  | PARG17906 | DNA-binding transcription factor activity | EREBP-like factor |
| SSC |  |  |  |  |  |  | PARG17907 | DNA-binding transcription factor activity | EREBP-like factor |
| SSC |  |  |  |  |  |  | PARG17909 | aminoacyl-tRNA ligase activity | methionyl-tRNA synthetase [EC:6.1.1.10] |
| SSC | **H_SSC18** | **Hg2** | **Marker64728** | LG4 | 19,712,829 | 20,787,883 | PARG15443 | catalytic activity | #N/A |
| SSC | S_SSC18 | Sg3 | Marker53093 |  |  |  | PARG15444 | ADP antiporter activity | #N/A |
| SSC |  |  |  |  |  |  | PARG15446 | zinc ion binding | #N/A |
| SSC |  |  |  |  |  |  | PARG15448 | molybdate ion transmembrane transporter activity | #N/A |
| SSC |  |  |  |  |  |  | PARG15450 | ATP binding | molecular chaperone DnaK |
| SSC |  |  |  |  |  |  | PARG15452 | ATPase activity | #N/A |
| SSC |  |  |  |  |  |  | PARG15454 | oxidoreductase activity | #N/A |
| SSC |  |  |  |  |  |  | PARG15455 | oxidoreductase activity | #N/A |
| SSC |  |  |  |  |  |  | PARG15457 | oxidoreductase activity | #N/A |
| SSC |  |  |  |  |  |  | PARG15458 | oxidoreductase activity | gibberellin 3beta-dioxygenase [EC:1.14.11.15] |
| SSC |  |  |  |  |  |  | PARG15460 | microtubule-severing ATPase activity | katanin p60 ATPase-containing subunit A1 [EC:5.6.1.1] |
| SSC |  |  |  |  |  |  | PARG15461 | catalytic activity | aromatic aminotransferase [EC:2.6.1.-] |
| SSC |  |  |  |  |  |  | PARG15462 | Cellular Component nucleus | D-glycerate 3-kinase [EC:2.7.1.31] |
| SSC |  |  |  |  |  |  | PARG15463 | regulation of transcription, DNA-templated | #N/A |
| SSC |  |  |  |  |  |  | PARG15464 | regulation of transcription, DNA-templated | #N/A |
| SSC |  |  |  |  |  |  | PARG15465 | thiolester hydrolase activity | #N/A |
| SSC |  |  |  |  |  |  | PARG15467 | thiolester hydrolase activity | #N/A |
| SSC |  |  |  |  |  |  | PARG15469 | protein binding | #N/A |
| SSC |  |  |  |  |  |  | PARG15471 | zinc ion binding | #N/A |
| SSC |  |  |  |  |  |  | PARG15477 | centromeric DNA binding | #N/A |
| SSC |  |  |  |  |  |  | PARG15478 | protein binding | #N/A |
| SSC |  |  |  |  |  |  | PARG15487 | zinc ion binding | #N/A |
| SSC |  |  |  |  |  |  | PARG15488 | protein binding | #N/A |
| SSC |  |  |  |  |  |  | PARG15489 | intracellular transport | #N/A |
| SSC |  |  |  |  |  |  | PARG15491 | vesicle-mediated transport | coatomer subunit beta' |
| SSC |  |  |  |  |  |  | PARG15493 | thymidylate kinase activity | dTMP kinase [EC:2.7.4.9] |
| SSC |  |  |  |  |  |  | PARG15497 | protein binding | #N/A |
| SSC |  |  |  |  |  |  | PARG15502 | helicase activity | regulator of telomere elongation helicase 1 [EC:3.6.4.12] |
| SSC |  |  |  |  |  |  | PARG15503 | negative regulation of transcription, DNA-templated | #N/A |
| SSC |  |  |  |  |  |  | PARG15505 | GTPase activity | Ras-related protein Rab-5C |
| SSC |  |  |  |  |  |  | PARG15506 | cellular response to nitrate | #N/A |
| SSC |  |  |  |  |  |  | PARG15508 | protein methyltransferase activity | methyltransferase-like protein 22 [EC:2.1.1.-] |
| SSC |  |  |  |  |  |  | PARG15509 | protein transport | #N/A |
| SSC |  |  |  |  |  |  | PARG15510 | threonine kinase activity | #N/A |
| SSC |  |  |  |  |  |  | PARG15512 | transmembrane transporter activity | #N/A |
| SSC |  |  |  |  |  |  | PARG15513 | transmembrane transporter activity | #N/A |
| SSC |  |  |  |  |  |  | PARG15514 | pyridoxal phosphate biosynthetic process | pyridoxal 5'-phosphate synthase pdxS subunit [EC:4.3.3.6] |
| SSC |  |  |  |  |  |  | PARG15517 | DNA helicase activity | chromosome transmission fidelity protein 1 [EC:3.6.4.13] |
| SSC |  |  |  |  |  |  | PARG15518 | structural constituent of ribosome | large subunit ribosomal protein L37e |
| SSC |  |  |  |  |  |  | PARG15520 | phosphatidylinositol binding | #N/A |
| SSC |  |  |  |  |  |  | PARG15521 | protein phosphorylation | interleukin-1 receptor-associated kinase 4 [EC:2.7.11.1] |
| SSC |  |  |  |  |  |  | PARG15522 | GTPase activity | Ras-related protein Rab-7A |
| SSC |  |  |  |  |  |  | PARG15524 | integral component of membrane | #N/A |
| SSC |  |  |  |  |  |  | PARG15525 | integral component of membrane | #N/A |
| SSC |  |  |  |  |  |  | PARG15526 | integral component of membrane | #N/A |
| SSC |  |  |  |  |  |  | PARG15527 | microtubule-based process | #N/A |
| SSC |  |  |  |  |  |  | PARG15531 | cell redox homeostasis | protein disulfide-isomerase A1 [EC:5.3.4.1] |
| SSC |  |  |  |  |  |  | PARG15532 | oxidation-reduction process | glyoxylate/hydroxypyruvate reductase [EC:1.1.1.79 1.1.1.81] |
| SSC |  |  |  |  |  |  | PARG15533 | Rho guanyl-nucleotide exchange factor activity | #N/A |
| SSC |  |  |  |  |  |  | PARG15534 | photosystem I reaction center | photosystem I subunit VI |
| SSC |  |  |  |  |  |  | PARG15536 | regulation of transcription, DNA-templated | homeobox-leucine zipper protein |
| SSC |  |  |  |  |  |  | PARG15539 | transmembrane transporter activity | TATA element modulatory factor |
| SSC |  |  |  |  |  |  | PARG15540 | integral component of membrane | #N/A |
| SSC |  |  |  |  |  |  | PARG15544 | catalytic activity | malonyl-CoA/methylmalonyl-CoA synthetase [EC:6.2.1.-] |
| SSC |  |  |  |  |  |  | PARG15545 | regulation of transcription, DNA-templated | #N/A |
| SSC |  |  |  |  |  |  | PARG15547 | catalytic activity | #N/A |
| SSC |  |  |  |  |  |  | PARG15548 | iron ion binding | #N/A |
| SSC |  |  |  |  |  |  | PARG15549 | protein dimerization activity | #N/A |
| SSC |  |  |  |  |  |  | PARG15551 | regulation of transcription, DNA-templated | #N/A |
| SSC |  |  |  |  |  |  | PARG15553 | protein binding | #N/A |
| SSC |  |  |  |  |  |  | PARG15554 | RNA binding | #N/A |
| SSC |  |  |  |  |  |  | PARG15556 | tRNA 3'-trailer cleavage | ribonuclease Z [EC:3.1.26.11] |
| SSC |  |  |  |  |  |  | PARG15557 | electron transfer activity | #N/A |
| SSC |  |  |  |  |  |  | PARG15558 | channel activity | #N/A |
| SSC |  |  |  |  |  |  | PARG15564 | transferase activity, transferring acyl groups other than amino-acyl groups | #N/A |
| SSC |  |  |  |  |  |  | PARG15565 | protein binding | #N/A |
| SSC |  |  |  |  |  |  | PARG15566 | ntracellular protein transport | #N/A |
| SSC |  |  |  |  |  |  | PARG15569 | voltage-gated proton channel activity | voltage-gated hydrogen channel 1 |
| SSC |  |  |  |  |  |  | PARG15575 | DNA binding | transcription factor MYB, plant |
| SSC |  |  |  |  |  |  | PARG15576 | protein kinase activity | #N/A |
| SSC | **S_SSC19** | **Sg4** | **Marker42337** | LG3 | 12,611,144 | 14,225,202 | PARG10729 | GTP binding | #N/A |
| SSC | H_SSC19 | Hg4 | Marker42196 |  |  |  | PARG10731 | L-methionine salvage from methylthioadenosine | methylthioribulose-1-phosphate dehydratase [EC:4.2.1.109] |
| SSC | H_FW18 | Hg4 | Marker42385 |  |  |  | PARG10733 | electron transfer activity | TBC1 domain family member 2 |
| SSC | H_FW18 | Hg4 | Marker118377 |  |  |  | PARG10736 | transmembrane transport | #N/A |
| SSC | H_FW18 | Hg4 | Marker42419 |  |  |  | PARG10738 | 3'-5' exonuclease activity | #N/A |
| SSC | H_FH18 | Hg4 | Marker42782 |  |  |  | PARG10739 | purine nucleoside transmembrane transporter activity | #N/A |
| SSC |  |  |  |  |  |  | PARG10740 | xenobiotic transmembrane transporter activity | #N/A |
| SSC |  |  |  |  |  |  | PARG10741 | xenobiotic transmembrane transporter activity | #N/A |
| SSC |  |  |  |  |  |  | PARG10742 | xenobiotic transmembrane transporter activity | #N/A |
| SSC |  |  |  |  |  |  | PARG10743 | DNA binding | #N/A |
| SSC |  |  |  |  |  |  | PARG10744 | DNA binding | #N/A |
| SSC |  |  |  |  |  |  | PARG10745 | DNA binding | #N/A |
| SSC |  |  |  |  |  |  | PARG10746 | DNA binding | #N/A |
| SSC |  |  |  |  |  |  | PARG10747 | DNA binding | #N/A |
| SSC |  |  |  |  |  |  | PARG10748 | xenobiotic transmembrane transporter activity | #N/A |
| SSC |  |  |  |  |  |  | PARG10749 | xenobiotic transmembrane transporter activity | #N/A |
| SSC |  |  |  |  |  |  | PARG10750 | 3-hydroxyacyl-CoA dehydrogenase activity | 3-hydroxybutyryl-CoA dehydrogenase [EC:1.1.1.157] |
| SSC |  |  |  |  |  |  | PARG10751 | protein kinase activity | #N/A |
| SSC |  |  |  |  |  |  | PARG10753 | DNA-binding transcription factor activity | #N/A |
| SSC |  |  |  |  |  |  | PARG10754 | protein serine/threonine phosphatase activity | protein phosphatase 1L [EC:3.1.3.16] |
| SSC |  |  |  |  |  |  | PARG10873 | calcium ion binding | #N/A |
| SSC |  |  |  |  |  |  | PARG10874 | DNA-binding transcription factor activity | #N/A |
| SSC |  |  |  |  |  |  | PARG10875 | DNA-binding transcription factor activity | #N/A |
| SSC |  |  |  |  |  |  | PARG10876 | ATP binding | #N/A |
| SSC |  |  |  |  |  |  | PARG10877 | cell-cell signaling involved in cell fate commitment | #N/A |
| SSC |  |  |  |  |  |  | PARG10879 | protein binding | #N/A |
| SSC |  |  |  |  |  |  | PARG10880 | malate dehydrogenase activity | tRNA dimethylallyltransferase [EC:2.5.1.75] |
| SSC |  |  |  |  |  |  | PARG10883 | nucleic acid binding | heterogeneous nuclear ribonucleoprotein A1/A3 |
| SSC |  |  |  |  |  |  | PARG10884 | DNA binding | #N/A |
| SSC |  |  |  |  |  |  | PARG10885 | protein binding | #N/A |
| SSC |  |  |  |  |  |  | PARG10886 | protein binding | #N/A |
| SSC |  |  |  |  |  |  | PARG10887 | protein binding | #N/A |
| SSC |  |  |  |  |  |  | PARG10889 | protein binding | #N/A |
| SSC |  |  |  |  |  |  | PARG10890 | protein binding | #N/A |
| SSC |  |  |  |  |  |  | PARG10892 | protein binding | #N/A |
| SSC |  |  |  |  |  |  | PARG10894 | protein binding | #N/A |
| SSC |  |  |  |  |  |  | PARG10895 | protein binding | #N/A |
| SSC |  |  |  |  |  |  | PARG10910 | protein binding | #N/A |
| SSC |  |  |  |  |  |  | PARG10911 | protein binding | #N/A |
| SSC |  |  |  |  |  |  | PARG10912 | zinc ion binding | #N/A |
| SSC |  |  |  |  |  |  | PARG10914 | protein binding | #N/A |
| SSC |  |  |  |  |  |  | PARG10917 | protein binding | #N/A |
| SSC |  |  |  |  |  |  | PARG10918 | cytidine to uridine editing | #N/A |
| SSC |  |  |  |  |  |  | PARG10919 | protein binding | #N/A |
| SSC |  |  |  |  |  |  | PARG10921 | protein binding | #N/A |
| SSC |  |  |  |  |  |  | PARG10923 | protein binding | #N/A |
| SSC |  |  |  |  |  |  | PARG10924 | protein binding | #N/A |
| SSC |  |  |  |  |  |  | PARG10925 | ATP binding | #N/A |
| SSC |  |  |  |  |  |  | PARG10927 | ATPase activity | #N/A |
| SSC |  |  |  |  |  |  | PARG10928 | protein kinase activity | protein deglycase [EC:3.5.1.124] |
| SSC |  |  |  |  |  |  | PARG10929 | N-acetyltransferase activity | #N/A |
| SSC |  |  |  |  |  |  | PARG10930 | galactosyltransferase activity | #N/A |
| SSC |  |  |  |  |  |  | PARG10932 | protein binding | #N/A |
| SSC |  |  |  |  |  |  | PARG10934 | extracellular space | #N/A |
| SSC |  |  |  |  |  |  | PARG10935 | UDP-N-acetylglucosamine 1-carboxyvinyltransferase activity | #N/A |
| SSC |  |  |  |  |  |  | PARG10936 | catalytic activity | protein phosphatase PTC7 [EC:3.1.3.16] |
| SSC |  |  |  |  |  |  | PARG10937 | catalytic activity | protein phosphatase PTC7 [EC:3.1.3.16] |
| SSC |  |  |  |  |  |  | PARG10938 | ATP binding | mitochondrial chaperone BCS1 |
| SSC |  |  |  |  |  |  | PARG10939 | ATP binding | #N/A |
| SSC |  |  |  |  |  |  | PARG10940 | catalytic activity | protein phosphatase PTC7 [EC:3.1.3.16] |
| SSC |  |  |  |  |  |  | PARG10941 | hydrolase activity, hydrolyzing O-glycosyl compounds | #N/A |
| SSC |  |  |  |  |  |  | PARG10942 | sequence-specific DNA binding | #N/A |
| SSC |  |  |  |  |  |  | PARG10944 | protein binding | #N/A |
| SSC |  |  |  |  |  |  | PARG10946 | zinc ion binding | bifunctional polynucleotide phosphatase/kinase [EC:3.1.3.32 2.7.1.78] |
| SSC |  |  |  |  |  |  | PARG10948 | regulation of GTPase activity | Rab3 GTPase-activating protein non-catalytic subunit |
| SSC |  |  |  |  |  |  | PARG10950 | polygalacturonase activity | #N/A |
| SSC |  |  |  |  |  |  | PARG10951 | uroporphyrinogen decarboxylase activity | uroporphyrinogen decarboxylase [EC:4.1.1.37] |
| SSC |  |  |  |  |  |  | PARG10955 | hydrolase activity, hydrolyzing O-glycosyl compounds | #N/A |
| SSC |  |  |  |  |  |  | PARG10960 | phosphoenolpyruvate carboxylase activity | phosphoenolpyruvate carboxylase [EC:4.1.1.31] |
| SSC |  |  |  |  |  |  | PARG10961 | mannosyltransferase activity | alpha-1,2-mannosyltransferase [EC:2.4.1.259 2.4.1.261] |
| SSC |  |  |  |  |  |  | PARG10962 | catalytic activity | protein phosphatase PTC7 [EC:3.1.3.16] |
| SSC |  |  |  |  |  |  | PARG10963 | N-acetyltransferase activity | #N/A |
| SSC |  |  |  |  |  |  | PARG10964 | N-acetyltransferase activity | #N/A |
| SSC |  |  |  |  |  |  | PARG10965 | N-acetyltransferase activity | #N/A |
| SSC |  |  |  |  |  |  | PARG10966 | N-acetyltransferase activity | parafibromin |
| SSC |  |  |  |  |  |  | PARG10967 | lipid binding | #N/A |
| SSC |  |  |  |  |  |  | PARG10971 | threonine-type endopeptidase activity | 20S proteasome subunit beta 4 [EC:3.4.25.1] |
| FF | **H_FF19** | **Hg1** | **Marker15306** | LG2 | 41,540,385 | 42,678,117 | PARG09078 | protein binding | #N/A |
| FF | **H_FF19** | **Hg1** | **Marker15130** |  |  |  | PARG09079 | catalytic activity | 3-ketoacyl-CoA synthase [EC:2.3.1.199] |
| FF | **H_FF19** | **Hg1** | **Marker15102** |  |  |  | PARG09080 | catalytic activity | 3-ketoacyl-CoA synthase [EC:2.3.1.199] |
| FF | **H_FF19** | **Hg1** | **Marker14962** |  |  |  | PARG09081 | catalytic activity | #N/A |
| FF |  |  |  |  |  |  | PARG09083 | protein binding | #N/A |
| FF |  |  |  |  |  |  | PARG09084 | signal recognition particle | signal recognition particle subunit SRP14 |
| FF |  |  |  |  |  |  | PARG09087 | NAD binding | 3-hydroxyisobutyrate dehydrogenase [EC:1.1.1.31] |
| FF |  |  |  |  |  |  | PARG09088 | nutrient reservoir activity | #N/A |
| FF |  |  |  |  |  |  | PARG09089 | GTP binding | ADP-ribosylation factor-like protein 2 |
| FF |  |  |  |  |  |  | PARG09091 | hydrolase activity | #N/A |
| FF |  |  |  |  |  |  | PARG09092 | Ran GTPase binding | #N/A |
| FF |  |  |  |  |  |  | PARG09109 | pseudouridine synthase activity | H/ACA ribonucleoprotein complex subunit 4 [EC:5.4.99.-] |
| FF |  |  |  |  |  |  | PARG09110 | Rab-protein geranylgeranyltransferase complex | #N/A |
| FF |  |  |  |  |  |  | PARG09117 | aminoacyl-tRNA ligase activity | tyrosyl-tRNA synthetase [EC:6.1.1.1] |
| FF |  |  |  |  |  |  | PARG09118 | ammonium transmembrane transporter activity | #N/A |
| FF |  |  |  |  |  |  | PARG09119 | protein binding | #N/A |
| FF |  |  |  |  |  |  | PARG09120 | phosphomethylpyrimidine kinase activity | hydroxymethylpyrimidine/phosphomethylpyrimidine kinase [EC:2.7.1.49 2.7.4.7] |
| FF |  |  |  |  |  |  | PARG09121 | protein kinase activity | serine/threonine-protein kinase SRK2 [EC:2.7.11.1] |
| FF |  |  |  |  |  |  | PARG09122 | integral component of membrane | transmembrane 9 superfamily member 3 |
| FF |  |  |  |  |  |  | PARG09123 | electron transfer activity | #N/A |
| FF |  |  |  |  |  |  | PARG09124 | protein binding | DDB1- and CUL4-associated factor 13 |
| FF |  |  |  |  |  |  | PARG09126 | pyridoxal phosphate binding | (R)-3-amino-2-methylpropionate-pyruvate transaminase [EC:2.6.1.44 2.6.1.40] |
| FF |  |  |  |  |  |  | PARG09127 | integral component of membrane | mlo protein |
| FF |  |  |  |  |  |  | PARG09128 | cytidine to uridine editing | #N/A |
| FF |  |  |  |  |  |  | PARG09130 | DNA binding | #N/A |
| FF |  |  |  |  |  |  | PARG09132 | DNA binding | #N/A |
| FF |  |  |  |  |  |  | PARG09133 | DNA binding | #N/A |
| FF |  |  |  |  |  |  | PARG09135 | DNA binding | #N/A |
| FF |  |  |  |  |  |  | PARG09138 | DNA-directed 5'-3' RNA polymerase activity | DNA-directed RNA polymerase I subunit RPA2 [EC:2.7.7.6] |
| FF |  |  |  |  |  |  | PARG09139 | phospholipase D activity | phospholipase D1/2 [EC:3.1.4.4] |
| FF |  |  |  |  |  |  | PARG09141 | intra-Golgi vesicle-mediated transport | conserved oligomeric Golgi complex subunit 1 |
| FF |  |  |  |  |  |  | PARG09143 | protein binding | #N/A |
| FF |  |  |  |  |  |  | PARG09144 | protein binding | #N/A |
| FF |  |  |  |  |  |  | PARG09145 | protein binding | #N/A |
| FF |  |  |  |  |  |  | PARG09146 | protein binding | #N/A |
| FF |  |  |  |  |  |  | PARG09147 | protein binding | #N/A |
| FF |  |  |  |  |  |  | PARG09148 | protein binding | #N/A |
| FF |  |  |  |  |  |  | PARG09149 | protein binding | #N/A |
| FF |  |  |  |  |  |  | PARG09150 | protein binding | #N/A |
| FF |  |  |  |  |  |  | PARG09151 | metal ion transmembrane transporter activity | #N/A |
| FF |  |  |  |  |  |  | PARG09152 | cellulose microfibril organization | #N/A |
| FF |  |  |  |  |  |  | PARG09153 | phosphoprotein phosphatase activity | ubiquitin-like domain-containing CTD phosphatase 1 [EC:3.1.3.16] |
| FF |  |  |  |  |  |  | PARG09154 | nucleic acid binding | DNA-directed RNA polymerase III subunit RPC6 |
| FF |  |  |  |  |  |  | PARG09155 | protein kinase activity | #N/A |
| FF |  |  |  |  |  |  | PARG09156 | transcription by RNA polymerase III | DNA-directed RNA polymerase III subunit RPC6 |
| FF |  |  |  |  |  |  | PARG09157 | DNA-binding transcription factor activity | #N/A |
| FF |  |  |  |  |  |  | PARG09160 | magnesium ion transmembrane transporter activity | #N/A |
| FF |  |  |  |  |  |  | PARG09161 | valine-tRNA ligase activity | valyl-tRNA synthetase [EC:6.1.1.9] |
| FF |  |  |  |  |  |  | PARG09162 | iron ion binding | #N/A |
| FF |  |  |  |  |  |  | PARG09163 | structural constituent of ribosome | large subunit ribosomal protein L24 |
| FF |  |  |  |  |  |  | PARG09165 | helicase activity | ATP-dependent RNA helicase DHX29 [EC:3.6.4.13] |
| FF |  |  |  |  |  |  | PARG09172 | protein binding | #N/A |
| FF |  |  |  |  |  |  | PARG09174 | membrane | #N/A |
| FF |  |  |  |  |  |  | PARG09178 | protein binding | #N/A |
| FF |  |  |  |  |  |  | PARG09179 | pseudouridine synthase activity | tRNA pseudouridine38-40 synthase [EC:5.4.99.12] |
| FF |  |  |  |  |  |  | PARG09180 | RNA binding | YTH domain-containing family protein |
| FF |  |  |  |  |  |  | PARG09181 | protein kinase activity | #N/A |
| FF |  |  |  |  |  |  | PARG09182 | structural constituent of ribosome | large subunit ribosomal protein L3e |
| FF |  |  |  |  |  |  | PARG09183 | DNA binding | #N/A |
| FF |  |  |  |  |  |  | PARG09187 | cytoplasm | autophagy-related protein 5 |
| FF |  |  |  |  |  |  | PARG09188 | protein binding | #N/A |
| FF |  |  |  |  |  |  | PARG09190 | heme binding | 5-O-(4-coumaroyl)-D-quinate 3'-monooxygenase [EC:1.14.14.96] |
| FF |  |  |  |  |  |  | PARG09191 | heme binding | 5-O-(4-coumaroyl)-D-quinate 3'-monooxygenase [EC:1.14.14.96] |
| FF |  |  |  |  |  |  | PARG09192 | oxidoreductase activity, acting on paired donors, with incorporation or reduction of molecular oxygen | 5-O-(4-coumaroyl)-D-quinate 3'-monooxygenase [EC:1.14.14.96] |
| FF |  |  |  |  |  |  | PARG09193 | endoplasmic reticulum to Golgi vesicle-mediated transport | trafficking protein particle complex subunit 2 |
| FF |  |  |  |  |  |  | PARG09194 | nucleic acid binding | #N/A |
| FF |  |  |  |  |  |  | PARG09195 | phosphoribosylamine-glycine ligase activity | phosphoribosylamine---glycine ligase [EC:6.3.4.13] |
| FF |  |  |  |  |  |  | PARG09196 | protein kinase activity | glycogen synthase kinase 3 beta [EC:2.7.11.26] |
| FF |  |  |  |  |  |  | PARG09197 | oxidoreductase activity | regulator of nonsense transcripts 3 |
| FF |  |  |  |  |  |  | PARG09198 | RNA binding | protein quaking |
| FF |  |  |  |  |  |  | PARG09200 | translation elongation factor activity | elongation factor 1-gamma |
| FF |  |  |  |  |  |  | PARG09201 | protein glycosylation | xyloglucan galactosyltransferase MUR3 [EC:2.4.1.-] |
| FF |  |  |  |  |  |  | PARG09202 | leucyl-tRNA aminoacylation | Ras-related protein Rab-1A |
| FF |  |  |  |  |  |  | PARG09203 | leucyl-tRNA aminoacylation | leucyl-tRNA synthetase [EC:6.1.1.4] |
| FF |  |  |  |  |  |  | PARG09205 | DNA binding | #N/A |
| FF |  |  |  |  |  |  | PARG09206 | aminoacyl-tRNA ligase activity | leucyl-tRNA synthetase [EC:6.1.1.4] |
| FF |  |  |  |  |  |  | PARG09207 | DNA binding | #N/A |
| FF |  |  |  |  |  |  | PARG09208 | ATP binding | #N/A |
| FF |  |  |  |  |  |  | PARG09209 | DNA-directed 5'-3' RNA polymerase activity | #N/A |
| FF |  |  |  |  |  |  | PARG09211 | glucosylceramidase activity | non-lysosomal glucosylceramidase [EC:3.2.1.45] |
| FF |  |  |  |  |  |  | PARG09212 | protein kinase activity | #N/A |
| FF |  |  |  |  |  |  | PARG09213 | zinc ion binding | #N/A |
| FF |  |  |  |  |  |  | PARG09215 | DNA binding | #N/A |
| FF |  |  |  |  |  |  | PARG09216 | zinc ion binding | #N/A |
| FF |  |  |  |  |  |  | PARG09218 | protein binding | #N/A |
| FF |  |  |  |  |  |  | PARG09219 | protein binding | #N/A |
| FF |  |  |  |  |  |  | PARG09221 | nucleotide-excision repair | transcription initiation factor TFIIH subunit 1 |
| FF |  |  |  |  |  |  | PARG09223 | acetylglucosaminyltransferase activity | #N/A |
| FF |  |  |  |  |  |  | PARG09228 | hydrolase activity | beta-glucosidase [EC:3.2.1.21] |
| FF |  |  |  |  |  |  | PARG09229 | mRNA splicing, via spliceosome | small nuclear ribonucleoprotein F |
| FF |  |  |  |  |  |  | PARG09230 | hydrolase activity, hydrolyzing O-glycosyl compounds | beta-glucosidase [EC:3.2.1.21] |
| FF |  |  |  |  |  |  | PARG09232 | mitochondrial pyruvate transmembrane transport | mitochondrial pyruvate carrier 1 |
| FF |  |  |  |  |  |  | PARG09234 | protein binding | #N/A |
| FF |  |  |  |  |  |  | PARG09235 | transmembrane transporter activity | #N/A |
| FF |  |  |  |  |  |  | PARG09237 | NAD+ kinase activity | #N/A |
| FF |  |  |  |  |  |  | PARG09238 | oxidoreductase activity | #N/A |
| FF |  |  |  |  |  |  | PARG09239 | mRNA splicing, via spliceosome | #N/A |
| FF |  |  |  |  |  |  | PARG09241 | response to hormone | #N/A |
| FF |  |  |  |  |  |  | PARG09242 | mitotic spindle assembly checkpoint | #N/A |
| FF |  |  |  |  |  |  | PARG09243 | transmembrane transporter activity | #N/A |
| FF |  |  |  |  |  |  | PARG09244 | protein binding | leucine-rich PPR motif-containing protein, mitochondrial |
| FF |  |  |  |  |  |  | PARG09245 | RNA binding | survival of motor neuron-related-splicing factor 30 |
| FF |  |  |  |  |  |  | PARG09246 | unfolded protein binding | #N/A |
| FF |  |  |  |  |  |  | PARG09108 | GTP binding | #N/A |
| FF | **H_FF18** | **Hg2** | **Marker64041** | LG5 | 586,137 | 762,265 | PARG16301 | ubiquitin protein ligase activity | E3 ubiquitin-protein ligase RNF115/126 [EC:2.3.2.27] |
| FF | **H_FF18** | **Hg2** | **Marker8120** |  |  |  | PARG16303 | aspartic-type endopeptidase activity | #N/A |
| FF |  |  |  |  |  |  | PARG16306 | aspartic-type endopeptidase activity | #N/A |
| FF |  |  |  |  |  |  | PARG16307 | calcium ion binding | #N/A |
| FF |  |  |  |  |  |  | PARG16308 | ATP binding | citrate synthase [EC:2.3.3.1] |
| FF |  |  |  |  |  |  | PARG16309 | protein metabolic process | #N/A |
| FF |  |  |  |  |  |  | PARG16312 | lipid binding | #N/A |
| FF |  |  |  |  |  |  | PARG16314 | symporter activity | #N/A |
| FF |  |  |  |  |  |  | PARG16321 | beta-N-acetylhexosaminidase activity | hexosaminidase [EC:3.2.1.52] |
| FF |  |  |  |  |  |  | PARG16322 | ribosome | small subunit ribosomal protein S13e |
| FF |  |  |  |  |  |  | PARG16323 | motor activity | myosin V |
| FF |  |  |  |  |  |  | PARG16329 | zinc ion binding | #N/A |
| FF |  |  |  |  |  |  | PARG16334 | protein binding | #N/A |
