# Supplementary figures and images for: Construction of a High-Density Genetic Map and Identification of Quantitative Trait Loci Linked to Fruit Quality Traits in Apricots Using Specific-Locus Amplified Fragment Sequencing
Source: Front Plant Sci. 2022 Feb 14;13:798700. doi: 10.3389/fpls.2022.798700 (PMC8882730; doi:10.3389/fpls.2022.798700)

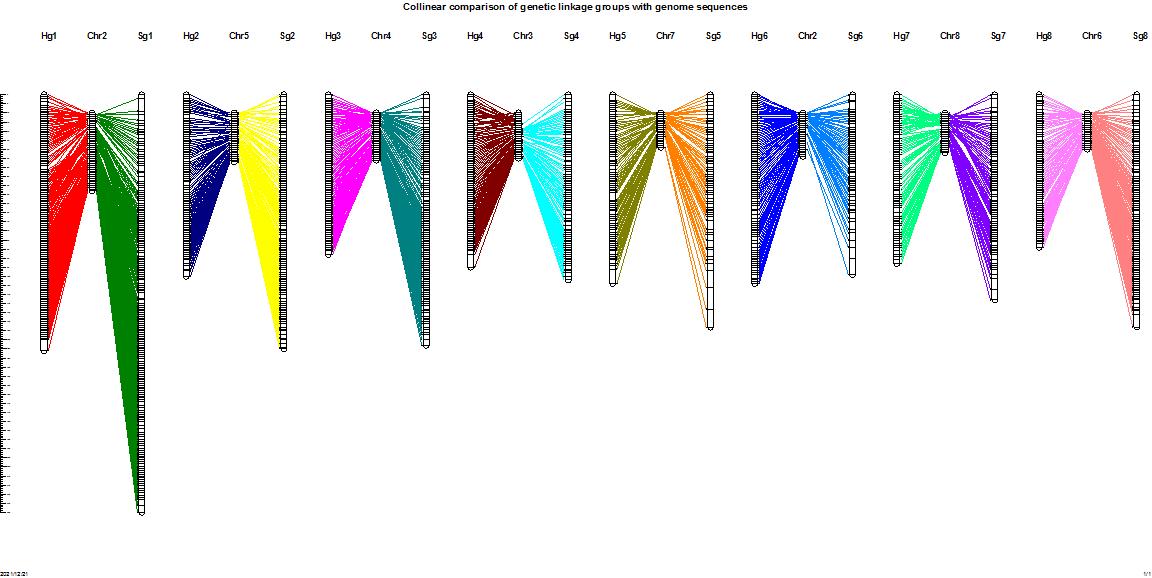

Supplement: Supplementary file 8 [file Image_1.jpg]
